# Omics Derived Biomarkers and Novel Drug Targets for Improved Intervention in Advanced Prostate Cancer

**DOI:** 10.3390/diagnostics10090658

**Published:** 2020-08-31

**Authors:** Maria Frantzi, Marie C. Hupe, Axel S. Merseburger, Joost P. Schanstra, Harald Mischak, Agnieszka Latosinska

**Affiliations:** 1 Mosaiques Diagnostics GmbH, 30659 Hannover, Germany; mischak@mosaiques-diagnostics.com (H.M.); latosinska@mosaiques-diagnostics.com (A.L.); 2 Department of Urology, University Hospital Schleswig-Holstein, Campus Lübeck, 23538 Lübeck, Germany; MarieChristine.Hupe@uksh.de (M.C.H.); Axel.Merseburger@uksh.de (A.S.M.); 3 Institut National de la Santé et de la Recherche Médicale (INSERM) U1048, Institute of Cardiovascular and Metabolic Diseases, 31432 Toulouse, France; joost-peter.schanstra@inserm.fr; 4 Université Toulouse III Paul-Sabatier, 31400 Toulouse, France; 5 Institute of Cardiovascular and Medical Sciences, University of Glasgow, Glasgow G12 8TA, UK

**Keywords:** biomarkers, drug targets, omics, prostate cancer

## Abstract

Prostate cancer (PCa) is one of the most frequently diagnosed malignancies, and the fifth leading cause of cancer related mortality in men. For advanced PCa, radical prostatectomy, radiotherapy, and/or long-term androgen deprivation therapy are the recommended treatment options. However, subsequent progression to metastatic disease after initial therapy results in low 5-year survival rates (29%). Omics technologies enable the acquisition of high-resolution large datasets that can provide insights into molecular mechanisms underlying PCa pathology. For the purpose of this article, a systematic literature search was conducted through the Web of Science Database to critically evaluate recent omics-driven studies that were performed towards: (a) Biomarker development and (b) characterization of novel molecular-based therapeutic targets. The results indicate that multiple omics-based biomarkers with prognostic and predictive value have been validated in the context of PCa, with several of those being also available for commercial use. At the same time, omics-driven potential drug targets have been investigated in pre-clinical settings and even in clinical trials, holding the promise for improved clinical management of advanced PCa, as part of personalized medicine pipelines.

## 1. Introduction

Prostate cancer (PCa) is placed second among the most frequently diagnosed cancers in males worldwide [1], affecting approximately one out of nine men during their lifetime [2]. In 2018, over 1.2 million men were diagnosed with PCa worldwide [3], ranking this malignancy as the one with the highest incidence among men in 114 countries and the first cause of cancer related deaths in 56 countries [4]. Unfortunately, there is a treatment paradox regarding PCa. On the one hand, more than 40% of the PCa patients experience slow-growing cancer forms (with local and regional cancer spread), for whom the 5-year survival rate is almost 100% [5]. For such patients, curative options do exist, but patients with indolent clinically insignificant PCa are frequently overtreated. On the other hand, the same malignancy is regarded as not curable at an advanced stage. For advanced PCa, radical prostatectomy (RP), radiotherapy, and/or long-term androgen deprivation therapy (ADT) are the recommended treatment options as part of multi-modal therapy [6]. Yet, after resistance to ADT, PCa progresses to metastatic castration resistant PCa (mCRPC), and treatment options are limited. As a result, the 5-year survival prognosis is reduced to 29% [5].

Clinical practice for monitoring PCa progression and metastasis is currently based on imaging techniques, such as bone scans, computed tomography (CT), magnetic resonance imaging (MRI) scans, and the recently introduced prostate-specific membrane antigen imaging positron emission tomography (PSMA PET-CT), which are typically performed after a raise in PSA levels. Nevertheless, although PSMA PET-CT has demonstrated superior sensitivity for detecting lymph node cancer spread, its potential impact on clinical management is still under evaluation [7]. Considering these clinical challenges, prognostic biomarkers for monitoring PCa recurrence and/or metastasis are necessary. Upon PCa recurrence and progression, and as the prognosis worsens, several second line treatment options are available, including taxane-based chemotherapy (like cabazitaxel or docetaxel), agents targeting the androgen receptor (AR), such the AR antagonists enzalutamide, apalutamide, darolutamide, and the cytochrome P450 inhibitor abiraterone acetate, immunotherapy (sipuleucel-T and immune checkpoint inhibitors), radiotherapy (including radium-223 and Lu-177-PSMA-617) [2], and the recently approved targeted therapies to inhibit poly (adenosine diphosphate-ribose) polymerase (PARP) such as olaparib, niraparib, and rucaparib. Yet, the response rates to the above therapies are low and so is the overall survival for these patients. Considering the heavy burden of advanced PCa, novel, more specific treatments based on interpretation of omics findings are expected to be of high value, along with effective tools to predict and monitor treatment response and guide individualized intervention for mCRPC.

**Figure 1:**
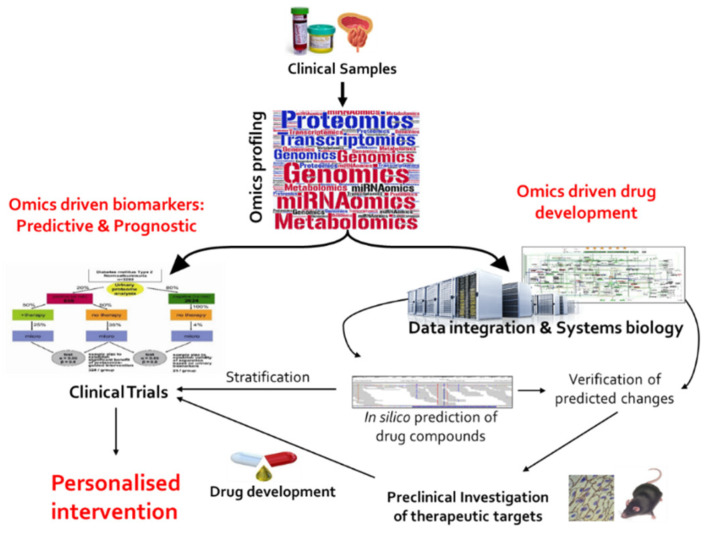
Directions of omics-driven studies towards personalized intervention for advanced prostate cancer (PCa). This figure has been adapted from [10] with permission.

Omics technologies, such as genomics, epigenomics, transcriptomics, proteomics, and metabolomics/lipidomics have facilitated simultaneous analysis of thousands of molecular features, resulting in a better definition of molecular pathophysiology in chronic heterogeneous diseases, like cancer [8]. As a result, various clinically relevant actionable mutated genes, disease-altered pathways, and molecular patterns with prognostic and predictive significance were introduced, giving rise to personalized medicine approaches [9]. For advanced PCa in particular, molecularly driven drug targets are expected to improve intervention, as part of tailored treatment strategies based on novel more specific therapies, guided by omics-based biomarkers. The two pipelines for omics-driven personalized intervention in advanced PCa are schematically presented in Figure 1. Considering these two directions, for the purpose of this review, a systematic literature search was conducted using the Web of Science database to critically evaluate recent omics studies focusing on: a) Biomarker development and b) introduction and verification of potential drug targets. Overall, this review aims to critically present the omics-driven studies and assess their clinical implementation potential in advanced PCa.

## 2. Literature Search and Review Strategy

**Figure 2:**
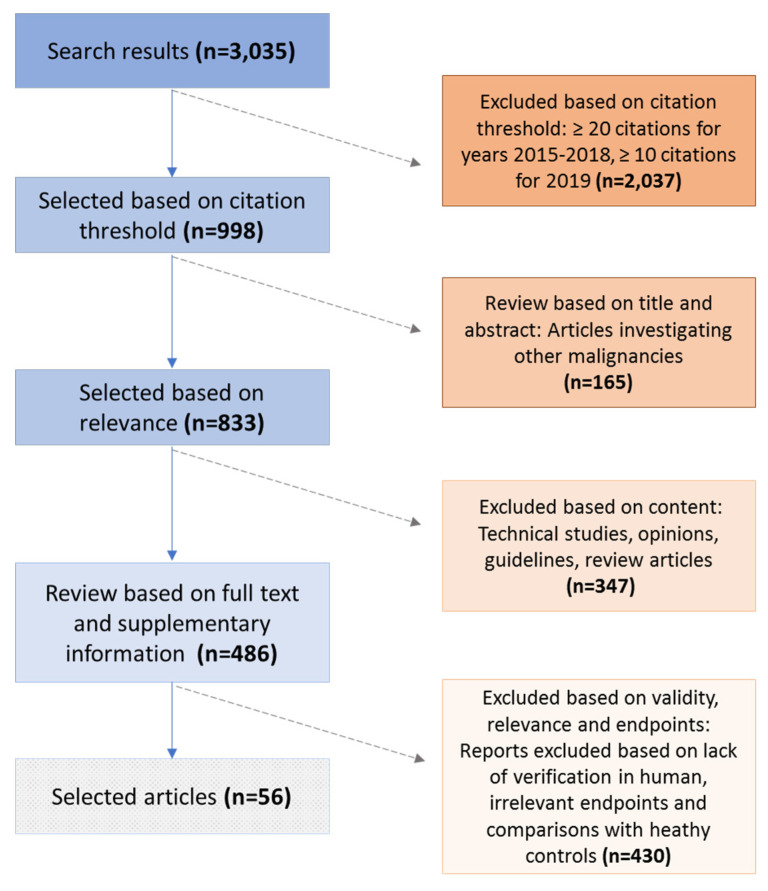
Schematic representation of the performed literature search and the review strategy.

A systematic literature search was performed through the Web of Science search platform on 15 July 2020. Published reports were retrieved from Web of Science Core Collection based on the following search terms: (1) TOPIC: (“omic*” or “proteom*” or “transcriptom*” or “genom*” or “metabolom*”) AND TOPIC: (“prostate ca*” or “prostate adeno*”) AND TOPIC: (“drug” or “therap*” or “biomarker*” or “marker*”) and (2) Timespan: 2015–2020. The above search resulted in the retrieval of 3035 manuscripts. The manuscripts were further shortlisted based on the number of citations, as follows: A threshold of ≥20 was applied for the years 2015, 2016, 2017, and 2018. A citation threshold of ≥10 was applied for manuscripts published in year 2019, and no citation threshold was applied for articles published in year 2020. Shortlisting based on the number of citations resulted in 998 selected manuscripts (Table S1). These 998 manuscripts were subsequently screened for their relevance in the field of omics-derived biomarkers and/or potential drug targets in prostate cancer. Methodological papers, papers presenting databases and web-based tools, reviews, opinion articles, commentaries, conference and case reports were excluded, as well as manuscripts referring to different malignancies (*n* = 575 articles). As a result, 423 shortlisted original manuscripts were further screened based on the following criteria: (a) Investigation of advanced PCa pathology; (b) reporting on relevant clinical endpoints (i.e., prediction of metastasis, biochemical recurrence, overall survival, prediction of treatment of advanced PCa); (c) application of at least one omics platform; and (d) investigation in human specimens (excluding studies performed only in cell lines and/or animal models). After thorough screening, 56 studies were selected and presented in this review (Table S2), based on agreement between two independent evaluators (co-authors). A graphical representation of the search and review strategy is presented in Figure 2.

## 3. Molecular Landscape of Localized and Advanced Prostate Cancer

### 3.1. Molecular Subclassification of PCa Tumours

Several large omics datasets, including genomes [11,12,13], mitochondrial genomes [12], transcriptomes [12], methylation datasets [12,13], and proteogenomic datasets [14,15] that were acquired through omics analysis of PCa tumors, are available via large scientific networks and research consortia. Comprehensive analysis of the above datasets revealed a plethora of molecular alterations that have been reported in PCa, all indicating a significant inter-patient heterogeneity within the PCa tumors. Among the most prominent molecular alterations, oncogene amplifications in AR gene, MYC proto-oncogene, and phosphatidylinositol-4,5-bisphosphate 3-kinase catalytic subunit alpha (PIK3CA) gene were reported. Additionally, frequent mutations were evident in tumor suppressor genes like phosphatase and tensin homolog (PTEN), the gene encoding tumor protein p53 (TP53), and prostatic tumor suppressor Homeobox protein Nkx-3.1 (NKX3-1). In parallel, oncogenic gene fusions were characterized, including the fusion of the androgen regulated erythroblast transformation-specific (ETS)-related gene (ERG) with the gene encoding for transmembrane protease serine 2 (TMPRSS2). Furthermore, additional point mutations were identified in PCa tumors involving speckle type BTB/POZ protein coding gene (SPOP) and forkhead Box A1 (FOXA1), as well as multiple AR splice variants (like AR-V7 and AR-V9). 

In one of the most comprehensive molecular analyses reported by the Cancer Genome Atlas project (TCGA) [13], 333 primary PCa tumors were profiled for somatic copy number variations, methylation, transcript levels, and microRNAs, and were further complemented with protein data (through reverse phase protein arrays) [13]. As a result, a molecular taxonomy of PCa was proposed, presenting seven main subtypes, into which 74% of the PCa primary tumors could be classified. Moreover, molecular changes in several interlinked pathways, like signaling and DNA damage repair pathways, were reported [13]. The majority of tumors (59%) were enriched in gene fusions of the ETS family and were classified into the first four classes: Gene fusions of ERG (46%), ETS variant transcription factor (ETV) 1 (8%), ETV4 (4%), and follicular lymphoma susceptibility to 1 (FL1; 1%). The ERG gene fusions were observed together with androgen regulated partner genes, most frequently with TMPRSS2, but also with solute carrier family 45 member 3 (SLC45A3) and NDRG1 gene. Furthermore, ETS positive tumors were identified harboring PTEN deletions [13]. The second most frequent observed subclass of tumors was characterized by the presence of mutations in the SPOP gene (11%), followed by classes carrying mutations in FOXA1 (3%), and isocitrate dehydrogenase 1 (IDH1; 1%) genes. The SPOP-mutant class was additionally characterized by deletions of chromodomain helicase DNA binding protein 1 (CHD1), 6q, and 2q. SPOP mutations were reported to deregulate AR and AR coactivators, leading to increased AR activity. Additional molecular analysis revealed that, regardless of the above seven subclasses, 25% of the prostate cancers harbored mutations in genes involved in kinase signaling pathways, such as PI3K or mitogen-activated protein kinase (MAPK) pathways, and 19% of the PCa tumors harbored inactivating mutations in DNA repair genes [13]. 

Complementary analysis by comprehensive integration of proteomics and genomics datasets indicated that tumors characterized by ETS fusions presented an extensive dysregulation of downstream metabolic pathways [14]. Similar to this observation, in a proteomics study including high-resolution proteomics on tissue specimens from 22 PCa patients, Latosinska et al., reported on 1433 proteins, out of which, 145 were significantly altered in advanced PCa. Downstream functional enrichment analysis revealed alterations in several metabolic pathways, metabolism, and signaling, including among others protein interactions between MYC targets [16].

### 3.2. Molecular Evolution of Metastatic Disease

In an integrative analysis aiming to characterize the molecular landscape of metastatic PCa in comparison to primary tumors [17], whole exome sequencing of 150 mCRPC tumors was performed. The analysis revealed alterations in AR, TP53, PTEN, and ETS genes at high frequency (40–60%). When comparing the mCRPC exome profiles with those from primary tumors (*n* = 440; TCGA dataset), TP53 and AR mutations were enriched, together with alterations in DNA damage repair related genes [like breast cancer type 1 and 2 susceptibility genes (BRCA1, BRCA2) and ataxia telangiectasia-mutated gene (ATM)]. In parallel, there was a complete absence of IDH1 mutations in metastatic tumors. Compared to primary PCa, mCRPC tumors showed higher overall burden in copy number variations and point mutations in the PI3K pathway, but also more frequent amplification and/or mutation of AR signaling in metastatic tumors [17]. Towards that end, a longitudinal genomics study including 176 primary and metastatic PCa tumors derived from the same individuals (*n* = 63) was conducted. Whole exome sequencing, microarray hybridization, and copy number variation analyses in the above cohort confirmed previous findings on recurrent alterations in AR, ERG, TP53, and SPOP genes. The analysis additionally supported high inter-patient, but relatively low intra-patient, heterogeneity in relation to the genomic events [18], further suggesting a clonal evolution of metastatic disease. This phenomenon is even more prominent in untreated patients with advanced PCa, where the large majority of driver gene mutations were common between primary and metastatic PCa tumors within individual patients, suggesting low intra-patient heterogeneity [19]. In the above study, whole exome and whole genome sequencing profiles were analyzed from 20 treatment-naïve patients and 76 untreated metastases. The detected alterations were further mapped into phylogenetic trees to determine timing and evolution of mutational landscape. Based on this analysis, evidence from untreated patients demonstrates that driver mutations promote metastasis seeding, with increased heterogeneity in slower growing primary tumors. This is in contrast to driver mutations that result in fast metastasis and are correlated with lower intra-patient heterogeneity, as metastasis occurs before different driver subclones are expanded [19]. Genomic evolution of metastasis was more precisely investigated by employing phylogenetic analysis, in a cohort of 51 tumors derived from 10 patients that were analyzed via whole genome sequencing. The results of this study supported that metastatic evolution related to castration resistance is based on clonal seeding of the cells from the primary tumor, with whom there is a common genetic imprint [20]. The clonal seeding is a result of the tumor′s requirement to overcome androgen deprivation, which consequently results in multiple resistant subclones [20]. Additionally, mutations in TP53 and DNA damage repair genes have been identified as driver mutations in PCa metastasis. This scientific evidence supports previous reports [21], which have suggested a higher overall burden of mutations in DNA repair and other signaling pathways in metastatic PCa tumors. 

### 3.3. Molecular Classification of Rare Prostate Tumours

Characterization of PCa at the molecular level has additionally enabled a better understanding of rare aggressive PCa clinical phenotypes, such as small cell prostate carcinoma (SCPC) with neuroendocrine differentiation. Neuroendocrine prostate cancers (NEPC) frequently present an aggressive phenotype [22] and typically do not respond to ADT [22,23]. In a prospective study including 202 PCa patients who progressed after abiraterone or enzalutamide treatment, 17% were characterized with SCPC phenotype (exhibiting neuroendocrine features) [23]. This group of patients experienced decreased overall survival (OS) after ADT [hazard ratio (HR) of 2.02; *p* = 0.027]. Genomics analysis in the above cohort revealed that in the SCPC tumors, mutations in genes related to DNA repair pathway, such as in BRCA1, BRCA2, ATM, cyclin-dependent kinase 12 (CDK12), partner and localizer of BRCA2 (PALB2), fanconi anaemia, complementation group A (FANCA), checkpoint kinase 2 (CHEK2), mutL homolog 1 (MLH1), mutS homolog 2 (MSH2), MSH3, MLH3, and MSH6 were less frequent compared to the rest cohort (*p* = 0.035). Instead, prominent alterations in SCPC tumors included amplification of AR gene supported by strong nuclear staining (assessed by immunohistochemistry), frequent loss of TP53 and retinoblastoma protein 1 (RB1), likely as a result of treatment selective pressure related to androgen antagonists. Additional alterations included upregulation of E2F1 transcription factor, overexpression of DEK proto-oncogene (E2F target), and upregulation of pancreatic and duodenal homeobox 1 (PDX1), a Hox-type transcription factor, which promotes neuroendocrine differentiation in the pancreas [23].

In a similar study, whole exome sequencing in 32 PCa patients revealed serine/arginine repetitive matrix 4 (SRRM4) splicing factor as a master regulator of >66% of downstream events that drive neurogenesis, through targeting RE1 silencing transcription factor (REST) and enhancing loss of function of TP53 [22]. Similarly, in a study employing copy number variation analysis in tumors from 59 PCa patients, SCPC tumors revealed RB1 loss, TP53 mutations, amplification of aurora kinase A gene (AURKA) and N-Myc proto-oncogene (MYCN), as well as expression of luminal epithelial markers and proneural transcription factors [24]. MYCN is an oncogenic transcription factor that has been reported to promote neural lineage gene expression in PCa [25]. MYCN is stabilized by AURKA, which prevents MYCN degradation in SCPC (and other malignancies with neuronal differentiation). Among others, MYCN promotes tumor aggressiveness by driving AR independence. Based on this important molecular finding, a clinical trial has been initiated, using the AURKA inhibitor alisertib to disrupt the stabilized complex with MYCN. In this phase 2 clinical trial, patients with AR-independent neuroendocrine PCa were treated with alisertib. Genomic analysis confirmed mutations in RB1 (55%), TP53 (46%), PTEN (29%), and AR (27%). However, while patients with AURKA mutations showed increased OS after treatment with alisertib, the difference was not significant (*p* = 0.05).

Along these lines, following the observations that advanced prostate cancer shares multiple characteristics with stem cells, such as self-renewal and proliferative capacity, tumor-associated calcium signal transducer 2 (TROP2) was identified as a master regulator of prostate stem cell self-renewal [26] also contributing to the neuroendocrine phenotype in CRPC. Based on immunohistochemical analysis in 58 recurrent and 176 non recurrent PCa patients, TROP2 was identified as highly expressed in recurrent PCa and significantly correlated with biochemical recurrence over a period of nine years (BCR; *p* < 0.05) [27]. Thorough investigation at the pre-clinical setting also revealed that TROP2 mediated neuroendocrine differentiation, was additionally accompanied with significant downregulation of AR pathway and concomitant upregulation of PARP1. Based on these findings, a clinical trial testing sacituzumab govitecan, an antibody-drug conjugate targeting TROP2 positive cells, has been recently initiated for patients with mCRPC, who have progressed after abiraterone or enzalutamide treatment (NCT03725761). Although TROP2′s association with neuroendocrine phenotype in CRPC, was not derived from a pure omics investigation, the above trial was included as relevant to the context based on the reviewer′s recommendation. 

## 4. Omics-Derived Biomarkers Predictive of Clinical Outcome and Treatment Response

### 4.1. Commercially Available Omics-Driven Biomarker Tests with Diagnostic Potential

Comprehensive molecular analysis applying the various omics platforms improved our understanding of molecular mechanisms underlying PCa pathology and offered a plethora of potential diagnostic, prognostic, and predictive biomarkers. As a result, several molecular based tests are currently available, aiming at improving on PCa clinical management [28]. These omics-driven tests include diagnostic biomarkers for PCa early detection and guiding biopsies. As this review focuses on advanced PCa, these tests are outside the scope of this article, and thus are only briefly described. Prostate cancer antigen (PCA3) or DD3 is a long non-coding RNA, and the first molecular urine-based assay that was approved by the U.S. Food and Drug Administration (FDA) for reducing unnecessary biopsies in a repeated biopsy setting [29,30]. PCA3 urinary testing, resulted in 67% sensitivity and 83% specificity for detection of PCa [31], while in the repeated biopsy setting PCA3 demonstrated sensitivity estimates in the range of 52–58% and specificity in the range of 72–87%, respectively [29,30]. Additional available tests based on omics biomarkers are the SelectMDx test, the Mi-Prostate Score, and the ExoDx Prostate Intelliscore. The selectMDx assay is based on the combination of two mRNA biomarkers (transcripts), homeobox protein (DLX-1), and homeobox protein Hox-C6 (HOXC6). SelectMDx resulted in an area under the curve (AUC) of 0.73 for detecting high grade PCa [Gleason score (GS) ≥ 7] [32]. Upon integration of the two molecular biomarkers with clinical variables (DRE, PSA density, and number of previous biopsies) the AUC estimate was improved to 0.89 [32]. Mi-Prostate Score (Michigan Prostate score/MiPS) refers to the combination of PCA3 long non-coding RNA with the gene fusion TMPRSS2-ERG that is described above and has been extensively studied though genomics studies in PCa progression [33]. The combined omics-based test in the form of a nomogram (Mi-Prostate Score) resulted in AUC estimates of 0.76 and 0.78 for detecting any PCa and high grade (GS ≥ 7) PCa, respectively [33]. ExoDx Prostate Intelliscore is a three-gene biomarker test based on the exosomal transcript levels of PCA3, ERG, and SAM pointed domain-containing Ets transcription factor (SPDEF). Combination of the above transcripts (i.e., the ExoDx Prostate Intelliscore) has been introduced to guide first biopsy [34] and resulted in an AUC of 0.71 [34]. An additional tissue-based assay called ConfirmMDx is also available, targeting epigenetic alterations (methylation) of promoter regions of Ras association domain-containing protein 1 (RASSF1), glutathione S-transferase pi gene (GSTP1), and adenomatous polyposis coli protein (APC) [35]. ConfirmMDx was developed with the intended application to reduce unnecessary repeated biopsies, demonstrating sensitivity and specificity estimates of 62% and 64%, respectively [35]. Following the same principle, DNA methylation particularly at targeted genes like GSTP1, cyclin-dependent kinase inhibitor 2A (CDKN2A), DNA (cytosine-5-)-methyltransferase 3 beta (DNMT3B), secretoglobin family 3A member 1 (SCGB3A1), and hypoxia-inducible factor 3 alpha (HIF3A), has been previously investigated in the context of PCa [36,37]. Most recently, published data including a genome-wide analysis in 79,194 prostate cancer patients revealed 759 CpG sites (corresponding to 82 genomic loci significantly associated with PCa risk; showing false discovery rate adjusted by Bonferroni correction; *p* ≤ 6.47 × 10^−7^). Out of those, 42 CpG sites were also correlated with altered expression of 28 target genes, 11 of which were subsequently validated in the TCGA cohort. Among the most promising genes altered, as a result of the CpG methylation, were nuclear ubiquitous casein and cyclin-dependent kinases substrate [NUCKS1; odds ratio (OR) of 1.35; *p* = 3.59 × 10^−5^], complement C4B gene (OR of 0.79; *p* = 2.18 × 10^−4^), cilia and flagella associated protein 44 (CFAP44; OR of 1.91; *p* = 9.11 × 10^−14^) [38]. Yet, validation of the above methylation targets in the appropriate clinical setting is required to demonstrate potential value as markers for PCa risk. 

### 4.2. Prognostic Biomarkers for Advanced PCa

#### 4.2.1. Genomics Based Biomarker Tests 

In the context of advanced PCa, molecularly based prognostic tools, predictive of PCa clinical outcome, are highly relevant. Among those, most studied commercially available gene panels for prediction of PCa outcome are: (a) Decipher, (b) Oncotype DX Genomic Prostate Score, and (c) Prolaris. All three gene-based prognostic biomarker tests are currently applied to predict disease outcomes in addition to clinical parameters or clinical nomograms [28]. Decipher is based on 22 coding and non-protein coding regions, which was developed based on post-operative tissue specimens from PCa patients who had undergone RP [39]. The intended context was to predict metastasis after RP [40,41] based on scientific evidence for significant prediction of metastasis after biochemical recurrence [hazard ratio (HR) of 1.37; *p* = 0.018] [42,43,44] and prediction of disease specific mortality (HR of 1.57; *p* = 0.037). Follow-up studies demonstrated that Decipher is an independent significant predictor of 5-year metastasis (OR of 1.48; *p* = 0.018) after adjusting for clinical risk factors, outperforming similar models (like Stephenson nomogram and CAPRA-S) [45]. Similarly, OncotypeDX is a 17-gene assay which is based on the detection of 12 prostate cancer related genes, encoding for zinc α 2-glycoprotein 1 (AZGP1), kallikrein-2 (KLK2), 3-oxo-5-alpha-steroid 4-dehydrogenase 2 (SRD5A2), protein FAM13C (FAM13C), filamin-C (FLNC), gelsolin (GSN), tropomyosin beta chain (TPM2), glutathione S-transferase Mu 2 (GSTM2), targeting protein for Xklp2 (TPX2), biglycan (BGN), collagen alpha-1(I) chain (COL1A1), secreted frizzled-related protein 4 (SFRP4), and five other reference genes. As part of investigations in advanced PCa, the above genomic score proved as a significant predictor of biochemical recurrence following RP (OR of 2.9; *p* < 0.001) [46,47] and predictor of metastasis (OR of 2.8; *p* < 0.001) [48]. Furthermore, the Prolaris test, a cell cycle progression test that is based on 46 genes, has been introduced as a tool to predict biochemical recurrence (BCR) based on RP tissue gene expression analysis [49,50]. The Prolaris test was able to predict BCR in PCa patients monitored for up to 72 months (HR of 1.44; *p* = 5.3 × 10^−4^) [50]. Unfortunately, a comparison of these 3 tests in the same cohort and consequently an assessment of potential additive value is not available yet. Moreover, prediction is ultimately only of value if it meets a consequence: Targeted therapeutic intervention. Unfortunately, to the best of our knowledge, a study investigating a benefit of intervention as a result of any of the predictive tests is still missing. 

#### 4.2.2. Biomarker Tests Targeting Non-Coding RNAs

Applying advanced genomic methods revealed that 66% of the genome is actively transcribed into noncoding RNAs (ncRNAs), while less than 2% of the sequences encode proteins [51,52]. ncRNAs are further categorized according to their length into: (a) Short and medium ncRNAs such as microRNAs (miRNAs), small nucleolar RNAs (snoRNAs), piwi interacting RNAs (defined based on their interaction with piwi subfamily Argonaute proteins [53]), and (b) long non-coding RNAs (lncRNAs). Both short and medium, as well as long non-coding RNAs, have been implicated in cancer initiation and progression [54]. 

In PCa in particular, apart from PCA3 or DDR, several other lncRNAs have been reported to be associated with advanced PCa, such as SWI/SNF complex antagonist associated with prostate cancer 1 (SChLAP1), nuclear enriched abundant transcript 1 (NEAT1), prostate-specific transcript 1 (PCGEM1), prostate cancer associated non-coding RNA 1 (PRNCR1), HOTAIR long noncoding RNA and prostate cancer associated transcript 1 (PCAT1) [55]. The most promising potential as a prognostic biomarker has been demonstrated for SChLAP1 (lncRNA), specifically implicated in advanced PCa and reported as significant predictor of PCa 10 year metastasis (OR of 2.45; *p* < 0.0001) [56]. A further lncRNA, prostate cancer associated transcript 14 (PCAT14), was investigated for its potential as a prognostic PCa biomarker. Following an integrative analysis of RNA sequencing data (*n* = 58 primary PCa tumors) with Affymetrix datasets from 131 primary and 19 metastatic PCa tumors, PCAT14 was identified as significantly downregulated in metastatic PCa [57]. Patients with high PCAT14 expression showed increased metastasis free survival (HR of 0.66; *p* = 0.023), overall survival (HR of 0.71; *p* = 0.0044), and disease specific survival (HR of 0.54; *p* = 0.023) [57]. 

Along these lines, miRNAs have also been reported as potential prognostic biomarkers predicting clinical outcome of advanced PCa. MiR-221 and miR-222 were observed as downregulated in PCa as part of a tumor suppressor cluster [58]. Evidence based on molecular biology suggested that re-establishment of expression levels of both miRNAs inhibits cell migration and invasion. MiR-222, additionally demonstrated potential as a prognostic marker and independent predictor of progression-free survival in CRPC patients (HR of 0.21; *p* = 0.006) [58]. Similarly, screening of 752 miRNAs for significant correlations with PCa led to the identification of a three microRNA-based prognostic biomarker including miR-185-5p, miR-221-3p, and miR-326. This microRNA biomarker panel demonstrated a prognostic potential for predicting BCR of PCa (HR of 1.36; *p* = 0.031) in three RP cohorts over a time span of 120 months [59].

#### 4.2.3. Biomarker Tests Targeting Proteomics Features

A study employing high resolution proteomics analysis by mass spectrometry was performed in 28 prostate tumors from formalin-fixed paraffin-embedded prostatectomy samples [15]. Increased expression of proteins involved in multiple metabolic processes was detected, like fatty acid and protein synthesis, ribosomal biogenesis, and protein secretion, among others including carnitine palmitoyltransferase 2 (CPT2, fatty acid transporter), coatomer protein complex, subunit alpha (COPA, vesicle secretion), and mitogen- and stress-activated protein kinase 1 and 2 (MSK1/2, protein kinase). Additionally, proneuropeptide-Y (pro-NPY) was found overexpressed in PCa and was further correlated with lower PCa specific survival (HR 2.13; *p* = 0.0087) [15]. Moreover, starting from a proteomics discovery study including 381 PCa patients and by employing quantitative multiplex proteomics imaging, 12 protein biomarkers including actinin Alpha 1 (ACTN1), cullin 2 (CUL2), derlin 1 (DERL1), fusion RNA-binding protein FUS/TLS (FUS), mitochondrial 70 kDa heat shock protein (HSPA9), decaprenyl-diphosphate synthase subunit 2 (PDSS2), zinc finger protein PLAG1 (PLAG1), phospho-S6 Ribosomal Protein (pS6), mothers against decapentaplegic homolog 2 (SMAD2), mothers against decapentaplegic homolog 4 (SMAD4), voltage-dependent anion-selective channel 1 (VDAC1), and Y box binding protein 1 (YBX1), were identified as significantly correlated with PCa outcome [60]. Subsequently, eight of the above biomarkers (DERL1, PDSS2, pS6, YBX1, HSPA9, FUS, SMAD4, CUL2) demonstrated significant prognostic values when correlated with disease pathology and were further considered as an 8-biomarker panel. The latter was further validated in an independent cohort of 276 patients, demonstrating significant correlation in predicting aggressive non-localized >T3a or N+ or M+ PCa [60]. Unfortunately, no HR or significance measures were reported. While not investigating advanced PCa, in a recent paper, multiple urinary peptide based biomarkers were described as a result of investigating samples from 823 patients with different grades of PCa [61]. Nineteen endogenous peptides derived from different collagen chains, from fractalkine, semaphorin-7A, and from Protein phosphatase 1 regulatory subunit 3A were combined into a classifier using support vector machines. In an independent test set, this classifier could distinguish patients GS ≥ 7 with an AUC of 0.81 (*p* < 0.0001). Especially considering the ease and non-invasive procedure of sampling (urine) compared to the tissue-based biomarkers described above, further investigation of this classifier for its ability to predict progression to CRPC and mCRPC appears warranted. 

### 4.3. Predictive Biomarkers for Advanced PCa

#### 4.3.1. Circulating Tumor Cells as Predictive Biomarkers to Taxane Chemotherapeutic Agents

There is an emergent need for biomarkers to predict response to available treatments in patients with advanced PCa, especially those with CRPC. Towards this goal, several omics-driven studies have been published mainly investigating circulating tumor cells (CTCs) and cell-free DNA (cfDNA) extracted from serum. cfDNA concentration was evaluated as predictive biomarker in patients receiving taxane-based chemotherapy, as part of two phase 2 clinical trials, including more than 2300 CRPC patients. In the above trials, the patients received chemotherapy as first line treatment (*n* = 777 treated with cabazitaxel and *n* = 391 with docetaxel) and those receiving chemotherapy as second-line treatment (*n* = 1200 treated with cabazitaxel). In the pooled analysis, including 2368 patients, the baseline cfDNA concentration correlated with shorter progression free survival (PFS) (HR of 1.54; *p* = 0.004), and shorter OS after taxane-based chemotherapy (HR of 1.53, *p* = 0.001) in both first- and second-line chemotherapy settings [62]. The above results demonstrated a good predictive potential of cfDNA levels for predicting response to taxane-based chemotherapy [62].

#### 4.3.2. Androgen Receptor Expression as Predictive Biomarker to AR Targeted Therapies 

Molecular characterization of progression CPRC has revealed evolution to an AR- independent phenotype and, as described above, neuroendocrine tumor characteristics. As these types of tumors do not respond to AR-targeted treatments, such patients could potentially benefit from alternative treatment like chemotherapy and/or PARP inhibitors. As such, non-invasive detection of neuroendocrine transition could be applied as a stratification tool to stratify patients for AR-targeted therapies [63]. In a proof-of-concept prospective study including 27 CRPC patients (12 with neuroendocrine phenotype and 15 with atypia), CTCs were extracted and characterized via an immunofluorescence platform (Epic) demonstrating unique morphological and cell surface markers. Lower AR expression and lower cytokeratin expression was detected in NEPC compared to CTCs from all other CRPC patients [63]. The unique CTC markers indicating NEPC phenotype (such as cytokeratin, CD45, CD56) were subsequently selected to train a random forest classifier to detect neuroendocrine phenotype. This panel was further validated in an independent prospective cohort of 159 CRPC patients. Based on the classification, 17 out of 159 (10.7%) CRPC patients were classified with neuroendocrine phenotype based on the CTC markers. In these 17 patients a small, yet significantly higher proportion of visceral metastases (*p* = 0.04) was detected [63]. These results indicate that characterization of neuroendocrine specific CTCs in the CRPC setting might be a useful tool to stratify patients for AR-targeted therapies. However, a clinical study investigating such an approach is missing. 

In a similar study, serum cfDNA [64] was investigated with the aim to evaluate circulating AR copy number as a predictive marker for CRPC patients treated with enzalutamide after chemotherapy (docetaxel) [64]. The serum circulating AR copy number was assessed in a prospective cohort of 59 CRPC patients at the baseline and correlated with progression free (PFS) and OS. AR copy number gain was detected in 21 (36%) patients, which was further correlated with worse outcome, significantly lower PFS (2.4 vs. 4.0 months, *p* = 0.0004), and OS (6.1 vs. 14.1 months, *p* = 0.0003). More recently, a prospective multi-omics study was conducted by integrating cf-DNA and cf-RNA datasets from 67 mCRPC patients that receive AR antagonists (*n* = 41) or taxane-based chemotherapy (*n* = 26), assessing molecular alterations related to AR gene [65]. Copy number variations in AR and other somatic mutations (assessed based on cfDNA profiles) were studied, along with AR-variants (AR-V7 and AR-V9 based on cfRNA profiles) providing significant evidence that AR gain and presence of any AR alteration predicted reduced progression-free survival (HR of 3.2; *p* = 0.01 and HR of 3.0; *p* = 0.04) and reduced overall survival (HR of 2.8; *p* = 0.04 and HR 2.9; *p* = 0.03). The results though refer only to the patients that have received AR antagonists, whereas AR alteration status had no impact on the outcome after taxane-based chemotherapy [65]. 

#### 4.3.3. Mutations in DNA Damage Repair Genes as Predictive Markers to PARP Inhibitors

Based on multiple comprehensive molecular characterization analyses of advanced PCa, it was evident that mutations in DNA damage repair genes are frequently detected in mCRPC (>10%) [66]. Following this observation, the DNA damage response (DDR) mutational status was investigated as a predictive biomarker for treatment response in advanced PCa, such as docetaxel taxane chemotherapy, abiraterone or enzalutamide, as well as PARP inhibition with olaparib [66]. As predicted based on the molecular mechanisms, intervention with olaparib (PARP inhibitor) [67] in patients stratified based for DDR mutational status (mutations in BRCA1, BRCA2, or ATM) resulted in longer progression-free survival compared to placebo (7.4 months vs. 3.6 months; HR for progression was 0.34; *p* < 0.001). Therefore, in mCPRC patients who displayed disease progression after enzalutamide or abiraterone treatment and those stratified for DDR mutations, the use of PAPR inhibitors might be beneficial.

The molecularly driven biomarker tests, along with their clinical context of use and the targeted omics features are summarized in Table 1. Table 1 includes a list of the commercially available tests after obtaining approval from the FDA or certified via Clinical Laboratory Improvement Amendments (CLIA), as a result of multiple prospective clinical studies. Moreover, as described above, this review is focused on omics-driven biomarkers. However, in order to give a comprehensive view of the currently commercially available tests, omics-based biomarkers that are currently measured by RT-PCR are also listed.

## 5. Omics Derived Therapeutic Targets for Advanced PCa

### 5.1. Integrative Omics for Personalized Drug Targeting

Cross-correlation of multiple omics datasets is expected to improve the coverage of associated molecular pathways and thus to improve drug targeting. In such an integrative study, Drake and colleagues [74] investigated tissue specimens from tumors derived from mCRPC patients to integrate genomic, transcriptomic, and phosphoproteomic data for downstream pathway analysis. In this analysis, phoshopeptides corresponding to 74 kinases were identified, 18 of which were differentially phosphorylated in mCPRC (based on 1.5-fold threshold; adjusted *p* value < 0.05). Following this, differentially expressed master transcriptional regulators, mutated genes, and differentially activated kinases were integrated to develop a signaling network of druggable kinase pathways, characteristic of mCRPC. Within this network, among others, signaling proteins such as DNA-dependent protein kinase (PRKDC), 5′-AMP-activated protein kinase catalytic subunit alpha-2 (PRKAA2), protein tyrosine kinase 2 (PTK2), ribosomal protein S6 kinase alpha-4 (RPS6KA4), and cyclin-dependent kinase (CDK) family members were defined as druggable targets in mCRPC. Using MSigDB hallmark gene sets, six major signaling pathways were significantly enriched in mCRPC based on the phosphorylation status of key proteins as derived by the phosphoproteomics datasets. Enriched cancer hallmarks included among others: (a) The cell cycle pathway, (b) the DNA repair pathway, (c) AKT/mTOR/MAPK pathway, and (d) the nuclear receptor pathway. Importantly, by building an hierarchical kinase network, input from each patient could be imported and based on the aim to reverse as many altered disease specific molecular features as possible, the hierarchy based network reveals the top kinase targets for every individual patient based on connectivity scores [74].

### 5.2. Omics-Driven Potential Drug Targets and Downstream Omics Profiling

Starting from evaluation of patients′ genomics datasets, miR-195 was identified as an oncogene regulator implicated in malignant tumors [75]. In advanced PCa, miR-195 downregulation was significantly correlated with future metastasis (*p* < 0.001), and biochemical recurrence (*p* < 0.001) and an independent predictor for recurrence free survival (RFS; *p* = 0.022). Further preclinical verification confirmed the tumor suppressive role of miR-195 in PCa cell invasion, migration, and apoptosis assays in vitro [75]. Subsequent downstream mass spectrometry proteomics analysis revealed ribosomal protein S6 kinase B1 (RPS6KB1) as a novel direct target of miR-195. Following this observation, knockdown of RPS6KB1 could rescue the effects induced by miR-195, with matrix metallopeptidase 9 (MMP-9), vascular endothelial growth factor (VEGF), BCL2 associated agonist of cell death (BAD), and E-cadherin (CDH1) being downstream effectors of the miR-195-RPS6KB1 axis [75]. These results suggest a potential of miR-195 as a target for intervention in advanced PCa. 

Following the observation that SPOP is frequently mutated in advanced PCa, further investigations were initiated to elucidate the functional implications of SPOP alterations [76]. The SPOP gene encodes the E3 ubiquitin ligase substrate-binding adaptor speckle-type POZ protein, which binds to bromodomain and extra-Terminal motif (BET) proteins [such as bromodomain-containing protein 2 (BRD2, BRD3, and BRD4) and as a result, leads to ubiquitination and proteasomal degradation of the above proteins. Following this observation, downstream transcriptomics and BRD4 cistrome analyses for identifying cis-acting targets at genome-wide scale were performed. Based on these analyses, it was demonstrated that SPOP mutation enhances BRD4-dependent expression of GTPase Ras-related C3 botulinum toxin substrate 1 (RAC1), cholesterol biosynthesis genes, and AKT-mTORC1 activation. Moreover, SPOP mutation promotes BET inhibitor resistance, and this effect can be reversed by AKT inhibitors, suggesting a role of SPOP (the presence of mutated gene) as a biomarker to stratify PCa patients towards treatment with BET inhibitors [76].

As a follow-up to genomics data analysis leading to the observation that AR is overexpressed and hyperactivated in CRPC (via not yet exactly defined mechanisms), further investigations were performed to reveal the molecular pathways that lead to AR amplification [77]. As a result, in a study targeting retinoid acid receptor-related orphan receptor γ (ROR-γ) was found overexpressed in tumors from mCRPC patients. Additional in vitro experiments and downstream gene expression analysis revealed that ROR-γ exerts an antagonist effect on AR function. Following this, ROR-γ-selective antagonists (SR2211, XY018, and XY011) were applied and subsequently blocked the expression of AR variants such as AR-V7 as well as full-length AR at the gene transcriptional level, proposing ROR-γ as a potential drug target to overcome resistance to ADT therapy [77].

## 6. Conclusions

PCa is a highly heterogeneous malignancy, with advanced PCa still being a lethal disease. The increasing number and quality of omics derived datasets available enable a better molecular characterization of the prostate cancer underlying pathology. Based on comprehensive molecular analysis, several gene fusions, mostly concerning AR regulated genes, along with point mutations in oncogenes and tumor suppressor genes were revealed at an enhanced level when comparing primary to metastatic disease. Interestingly altered pathways, involving mutations in DNA damage repair genes frequently present in metastatic tumors were also implicated. Following up on this observation, novel treatment schemes involving PAPR inhibitors were investigated as alternative treatments in patients who progressed after abiraterone or enzalutamide treatment. In this setting, DDR mutational status is investigated as a biomarker tool to stratify patients eligible for treatment with PARP inhibitors. 

**Figure 3:**
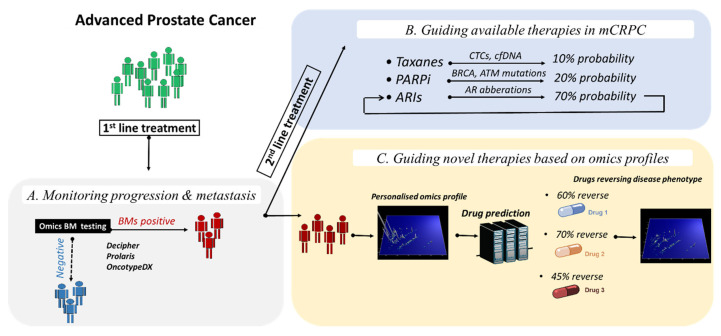
Applications of omics-driven biomarkers and novel drug targets in advanced PCa. After initial diagnosis of advanced PCa, first line treatment is initiated. (**A**) Omics-driven biomarkers with prognostic value can inform patients for risk of developing progression and/or metastasis. (**B**) After progression to metastatic disease, second line therapeutic options are available, including, among others, taxane-based chemotherapy, androgen receptor inhibition, and targeted therapy by recently approved Poly (ADP-ribose) polymerase inhibitors. In this setting, omics biomarkers to predict response to the different therapies are of value. (**C**) Nevertheless, as only part of mCRPC patients respond to these available therapies, novel drug targets based on omics derived molecular profiles are also expected to impact decision making in advanced PCa. Abbreviations: ARIs, Androgen receptor inhibitors; BM, biomarker; mCRPC, metastatic castration resistant prostate cancer; PARPi, Poly (ADP-ribose) polymerase inhibitors.

Complementary to this observation, molecularly driven prognostic biomarkers are of high importance in guiding patient management, particularly for monitoring recurrence/progression to CRPC and metastatic disease and guiding treatment in advanced PCa. Based on the retrieved studies from this systematic review, several prognostic biomarkers were identified as a follow-up to large omics data investigation and integration, including, among others, altered transcripts, non-coding genes (lncRNA, miRNAs) and proteins, with higher performance reported for combination of biomarkers into multi-parametric panels and/or by using algorithms or nomograms to additionally integrate clinical and other variables. Additionally, omics-derived predictive biomarkers, including, among others, results from large prospective clinical trials, demonstrate an additional potential for guiding first and second line treatment based on prediction of response to taxane-based chemotherapy, abiraterone and enzalutamide, and more recently, by stratifying mCRPC patients based on DDR mutational status for PARP inhibition. A summary of the most promising omics-driven biomarkers and their clinical potential in advanced PCa is depicted in Figure 3.

Based on this systematic search, it is evident that most omics-driven biomarkers and potential drug targets for advanced PCa, are based on genomics and/or proteomics rather than metabolomics. Following the selection criteria within this systematic literature search, although in the initial selection metabolomics studies were included (*n* = 21), this further prioritization procedure did not result in the final selection of publications based on metabolomics analysis. In particular, the above studies included results based on comparisons between PCa patients and healthy controls (or patients presenting with BPH; 14 studies), thus they were excluded. Additionally, seven studies reported experiments in cell lines without any verification phase for the biomarkers in human specimens and were also not selected for this review. As omics technologies are emerging and have already reached analytical maturity, the field is evolving towards multi-omics integration, on the one hand to develop multi-omics biomarkers, but also to suggest individualized intervention based on, e.g., druggable kinases. The multiple studies reported provide a very good background for the application of omics-driven biomarkers and novel therapeutic targets towards personalized medicine approaches in PCa management. This holds a substantial promise to support tailored treatment strategies, particularly for advanced PCa, where timing is crucial. It will now be important to achieve the transition from the multiple discovery studies, as reported here, to testing in appropriately powered prospective clinical trials, followed by implementation in patient management [78,79]. Unfortunately, funding for such efforts is scarce and frequently refused based on arguments like “lack of novelty”. Overall, the currently available data clearly indicate a potential benefit of the application of omics-based biomarkers in the management of PCa, especially in the context of personalized intervention. It is to be hoped that opportunities for thoroughly assessing this potential in properly powered clinical studies will be generated, despite the lack of interest of public funders, possibly via the inclusion of patient support groups.

## Figures and Tables

**Table 1:** Overview on omics-driven commercially available biomarker-based tests.

Biomarker Test	Omics Features	Assay Method	Biofluid/Biospecimen	Clinical Application	References
STHLM3 nomogram	PSA, free PSA, intact PSA, KLK2, MSMB, MIC1, 232 SNPs, age, family, history, DRE SNPs	PSA immunoassays + SNP genotyping	Serum	**Diagnostic** Guiding 1st biopsy	Grönberg et al., 2015 [68]
Select MDx	HOXC6 and DLX1 mRNAs	RT-PCR	Urine-based (post-DRE)	**Diagnostic** Guiding 1st biopsy	Van Neste et al., 2016 [32]
ExoDx Intelliscore	PCA3, ERG and SPDEF Exosomal mRNAs	RT-PCR	Urine	**Diagnostic** Guiding 1st biopsy	McKiernan et al., 2018 [34]
Progensa CA3	PCA3 (DD3) lnc RNA	RT-PCR	Urine (post-DRE)	**Diagnostic** Guiding 1st biopsy & repeated biopsies	Hessels et al., 2003 [31]; Marks et al., 2007 [29]; Ramos et al., 2013 [30]
MiProstate	TMPRSS2-ERG and PCA3 Gene fusion & lnc RNA	RT-PCR	Urine (post-DRE)	**Diagnostic** Guiding 1st & repeated biopsies	Leyten et al., 2014 [69] Tomlins et al., 2016 [33]
Confirm MDx	GSTP1, APC, RASSF Methylation	Multiplex PCR	Tissue (Biopsy)	**Diagnostic** Guiding repeated biopsies	Partin et al., 2014 [35]
OncotypeDx	12 cancer-related genes (AZGP1, KLK2, SRD5A2, FAM13C, FLNC, GSN, TPM2, GSTM2, TPX2, BGN, COL1A1, SFRP4) and 5 reference genes mRNAs	RT-PCR	Tissue (Biopsy)	**Diagnostic** Guiding repeated biopsies **Prognostic** Guiding active treatment & AS	Klein et al., 2014 [46] Cullen et al., 2015 [47]
SChLAP1	SChLAP1 lnc RNA	Microarray hybridization	Tissue (Radical prostatectomy)	**Prognostic** Guiding active treatment & AS	Prensner et al., 2015 [56]
Decipher	22 coding and non-protein coding regions mRNAs	Affymetrix microarrays	Tissue	**Prognostic** Monitoring metastasis	Glass et al., 2016 [42] Ross et al., 2014 [43]
Prolaris	31 cell cycle progression and 15 reference genes mRNAs	RT-PCR	Tissue (Radical prostatectomy)	**Prognostic** Monitoring biochemical recurrence	Leon et al., 2018 [50]
AR-V7	AR-V7 CTCs	RT-PCR, ddPCR	Serum	**Predictive** Response to abiraterone/enzalutamide	Seitz et al., 2017 [70,71,72,73]
DNA repair genes	BRCA1, BRCA2, or ATM mRNAs	Next-Generation Sequencing	Tissue	**Predictive** Response to opaparib	de Bono et al., 2020 [67]

Table Abbreviations: ATM—ataxia telangiectasia-mutated gene, APC—adenomatous polyposis coli, AR-V7—androgen-receptor splice variant 7 messenger RNA, AZGP1—zinc α 2-glycoprotein, BGN—biglycan, BRCA1—breast cancer type 1 susceptibility protein, BRCA2—breast cancer type 2 susceptibility protein, COL1A1—collagen alpha-1(I) chain, ddPCR—droplet digital PCR, DLX1—homeobox protein DLX-1, DRE—digital rectal examination, ERG—transcriptional regulator ERG, FAM13C—protein FAM13C, FLNC—filamin-C, GSN—gelsolin, GSTM2—glutathione S-transferase Mu 2, GSTP1—glutathione S-transferase pi gene, HOXC6—homeobox protein Hox-C6, KLK2—kallikrein-2, MIC1—macrophage inhibitory cytokine 1, MSMB—microseminoprotein beta, PCA3—prostate cancer gene 3, PSA—prostate specific antigen, RASSF—Ras association domain-containing protein 1, RT-PCR—reverse transcription polymerase chain reaction, SChLAP1—second chromosome locus associated with prostate-1, SFRP4—secreted frizzled-related protein 4, SNPs—single nucleotide polymorphisms, SPDEF—SAM pointed domain-containing Ets transcription factor, SRD5A2—steroid 5 Alpha-Reductase 2, TMPRSS2—transmembrane serine protease 2, TPM2—tropomyosin beta chain, TPX2—targeting protein for Xklp2.

## Supplementary Materials

The following are available online at https://www.mdpi.com/2075-4418/10/9/658/s1, Table S1: Complete list of the studies retrieved through literature search using Web of Science after applying the citation thresholds (*n* = 998), Table S2. List of selected studies that have been presented in the context of this review (*n* = 56).

## Abbreviations

ACTN1: actinin alpha 1, ADT: androgen deprivation therapy, APC: adenomatous polyposis coli, AR: androgen receptor, AR-V: androgen-receptor splice variant, ATM: ataxia telangiectasia-mutated gene, AUC: area under the curve, AURKA: aurora kinase A, AZGP1: zinc α 2-glycoprotein, BAD: BCL2 associated agonist of cell death, BCR: biochemical recurrence, BET: bromodomain and extra-Terminal motif proteins, BGN: biglycan, BRCA1: breast cancer type 1 susceptibility protein, BRCA2: breast cancer type 2 susceptibility protein, BRD2: bromodomain-containing protein 2, BRD3: bromodomain-containing protein 3, BRD4: bromodomain-containing protein 4, C4B: complement C4B, CDH1: E-cadherin, CDK: cyclin-dependent kinase, CDK12: cyclin-dependent kinase 12, CDKN2A: cyclin-dependent kinase inhibitor 2A, CFAP44: cilia and flagella associated protein 44, cfDNA: cell-free DNA, CHD1: chromodomain helicase DNA binding protein 1, CHEK2: checkpoint kinase 2, CLIA: clinical laboratory improvement amendments, COL1A1: collagen alpha-1(I) chain, CT: computed tomography, CTCs: circulating tumour cells, CUL2: cullin 2, ddPCR: droplet digital PCR, DERL1: derlin 1, DLX1: homeobox protein DLX-1, DNMT3B: DNA (cytosine-5-)-methyltransferase 3 beta, DRE: digital rectal examination, E2F1: E2F1 transcription factor, ETV: ETS variant transcription factor, ERG: ETS-related gene, ETS: erythroblast transformation-specific, FAM13C: protein FAM13C, FANCA: fanconi anaemia, complementation group A, FDA: U.S. food and drug administration, FL1: follicular lymphoma susceptibility to 1, FLNC: filamin-C, FOXA1: forkhead Box A1, FUS: fusion RNA-binding protein FUS/TLS, GS: gleason score, GSN: gelsolin, GSTM2: glutathione S-transferase Mu 2, GSTP1: glutathione S-transferase pi gene, HIF3A: hypoxia-inducible factor 3 alpha, HOXC6: homeobox protein Hox-C6, HSPA9: mitochondrial 70kDa heat shock protein, HR: hazard ratio, IDH1: isocitrate dehydrogenase 1, KLK2: kallikrein-2, lncRNA: long non-coding RNA, mCRPC: metastatic castration resistant prostate cancer, MIC1: macrophage inhibitory cytokine 1, miRNA: microRNA, MLH1: mutL homolog 1, MMP9: matrix metallopeptidase 9, MRI: magnetic resonance imaging, MSH2: mutS homolog, MSMB: microseminoprotein beta, MYCN: N-Myc proto-oncogene, ncRNA: noncoding RNA, NEAT: nuclear enriched abundant transcript 1, NEPC: neuroendocrine prostate cancer, NKX3-1: homeobox protein Nkx-3.1, NUCKS1: nuclear ubiquitous casein and cyclin-dependent kinases substrate, OR: odds ratio, OS: overall survival, PALB2: partner and localizer of BRCA2, PARP: poly(adenosine diphosphate-ribose) polymerase, PCa: prostate cancer; PCA3: prostate cancer gene 3, PCAT1: prostate cancer associated transcript 1, PCAT14: prostate cancer associated transcript 14, PCGEM1: prostate-specific transcript 1, PDSS2: decaprenyl-diphosphate synthase subunit 2, PDX1: pancreatic and duodenal homeobox 1, PI3KCA: phosphatidylinositol-4,5-bisphosphate 3-kinase catalytic subunit alpha, PLAG1: zinc finger protein PLAG1, PRKAA2: 5’-AMP-activated protein kinase catalytic subunit alpha-2, PRKDC: DNA-dependent protein kinase, PRNCR1: prostate cancer associated non-coding RNA 1, pS6: phospho-S6 ribosomal protein, PSA: prostate specific antigen, PSMA PET-CT: antigen prostate-specific membrane antigen imaging positron emission tomography, PTEN: phosphatase and tensin homolog, PTK2: protein tyrosine kinase 2, RAC1: Ras-related C3 botulinum toxin substrate 1, RASSF: Ras association domain-containing protein 1, RB1: retinoblastoma protein 1, REST: RE1 silencing transcription factor, ROR-γ: retinoid acid receptor-related orphan receptor γ, RP: radical prostatectomy, RPS6KA4: ribosomal protein S6 kinase alpha-4, RPBS6KB1: ribosomal protein S6 kinase B1, RT-PCR: reverse transcription polymerase chain reaction, SCGB3A1: secretoglobin family 3A member 1, SChLAP1: second chromosome locus associated with prostate-1, SCPC: small cell prostate carcinoma, SFRP4: secreted frizzled-related protein 4, SLC45A3: solute carrier family 45 member 3, SMAD2: mothers against decapentaplegic homolog 2, SMAD4: mothers against decapentaplegic homolog 4, snoRNAs: small nucleolar RNAs, SNPs: single nucleotide polymorphisms, SPDEF: SAM pointed domain-containing Ets transcription factor, SPOP: speckle type BTB/POZ protein coding gene, SRD5A2: steroid 5 Alpha-Reductase 2, SRRM4: serine/arginine repetitive matrix 4, TCGA: the cancer genome atlas, TMPRSS2: transmembrane serine protease 2, TP53: tumor protein p53, TPM2: tropomyosin beta chain, TPX2: targeting protein for Xklp2, TROP2: tumor-associated calcium signal transducer 2, VDAC1: voltage-dependent anion-selective channel 1, VEGF: vascular endothelial growth factor, YBX1: Y box binding protein 1.
