# Supplementary material for: Omics Derived Biomarkers and Novel Drug Targets for Improved Intervention in Advanced Prostate Cancer
Source: Diagnostics (Basel). 2020 Aug 31;10(9):658. doi: 10.3390/diagnostics10090658 (PMC7555799; doi:10.3390/diagnostics10090658)
Supplement: Supplementary file 1 [file diagnostics-10-00658-s001.zip › Table S1. List of selected manuscripts after applying citation threshold.pdf]

Table S1. List of selected manuscripts after applying citation threshold

Title	Authors	Journal	Publication Year	Volume	Issue	Total Citations	Average per Year
GEPIA: a web server for cancer and normal gene expression profiling and interactive analyses	Tang, Zefang; Li, Chenwei; Kang, Boxi; Gao, Ge; Li, Cheng; Zhang, Zemin	NUCLEIC ACIDS RESEARCH	2017	45	W1	1218	304,5
The landscape of long noncoding RNAs in the human transcriptome	Iyer, Matthew K.; Niknafs, Yashar S.; Malik, Rohit; Singhal, Udit; Sahu, Anirban; Hosono, Yasuyuki; Barrette, Terrence R.; Prensner, John R.; Evans, Joseph R.; Zhao, Shuang; Poliakov, Anton; Cao, Xuhong; Dhanasekaran, Saravana M.; Wu, Yi-Mi; Robinson, Dan R.; Beer, David G.; Feng, Felix Y.; Iyer, Hariharan K.; Chinnaiyan, Arul M.	NATURE GENETICS	2015	47	3	1100	183,33
Long Noncoding RNAs in Cancer Pathways	Schmitt, Adam M.; Chang, Howard Y.	CANCER CELL	2016	29	4	1076	215,2
DNA-Repair Defects and Olaparib in Metastatic Prostate Cancer	Mateo, J.; Carreira, S.; Sandhu, S.; Miranda, S.; Mossop, H.; Perez-Lopez, R.; Rodrigues, D. Nava; Robinson, D.; Omlin, A.; Tunariu, N.; Boysen, G.; Porta, N.; Flohr, P.; Gillman, A.; Figueiredo, I.; Paulding, C.; Seed, G.; Jain, S.; Ralph, C.; Protheroe, A.; Hussain, S.; Jones, R.; Elliott, T.; McGovern, U.; Bianchini, D.; Goodall, J.; Zafeiriou, Z.; Williamson, C. T.; Ferraldeschi, R.; Riisnaes, R.; Ebbs, B.; Fowler, G.; Roda, D.; Yuan, W.; Wu, Y. -M.; Cao, X.; Brough, R.; Pemberton, H.; A'Hern, R.; Swain, A.; Kunju, L. P.; Eeles, R.; Attard, G.; Lord, C. J.; Ashworth, A.; Rubin, M. A.; Knudsen, K. E.; Feng, F. Y.; Chinnaiyan, A. M.; Hall, E.; de Bono, J. S.	NEW ENGLAND JOURNAL OF MEDICINE	2015	373	18	935	155,83

The evolutionary history of lethal metastatic prostate cancer	Gundem, Gunes; Van Loo, Peter; Kremeyer, Barbara; Alexandrov, Ludmil B.; Tubio, Jose M. C.; Papaemmanuil, Elli; Brewer, Daniel S.; Kallio, Heini M. L.; Hoegnas, Gunilla; Annala, Matti; Kivinummi, Kati; Goody, Victoria; Latimer, Calli; O'Meara, Sarah; Dawson, Kevin J.; Isaacs, William; Emmert-Buck, Michael R.; Nykter, Matti; Foster, Christopher; Kote-Jarai, Zsofia; Easton, Douglas; Whitaker, Hayley C.; Neal, David E.; Cooper, Colin S.; Eeles, Rosalind A.; Visakorpi, Tapio; Campbell, Peter J.; McDermott, Ultan; Wedge, David C.; Bova, G. Steven	NATURE	2015	520	7547	604	100,67
Divergent clonal evolution of castration-resistant neuroendocrine prostate cancer	Beltran, Himisha; Prandi, Davide; Mosquera, Juan Miguel; Benelli, Matteo; Puca, Loredana; Cyrta, Joanna; Marotz, Clarisse; Giannopoulou, Eugenia; Chakravarthi, Balabhadrapatruni V. S. K.; Varambally, Sooryanarayana; Tomlins, Scott A.; Nanus, David M.; Tagawa, Scott T.; Van Allen, Eliezer M.; Elemento, Olivier; Sboner, Andrea; Garraway, Levi A.; Rubin, Mark A.; Demichelis, Francesca	NATURE MEDICINE	2016	22	3	485	97
Long Noncoding RNA and Cancer: A New Paradigm	Bhan, Arunoday; Soleimani, Milad; Mandal, Subhrangsu S.	CANCER RESEARCH	2017	77	15	457	114,25
Prostate cancer	Attard, Gerhardt; Parker, Chris; Eeles, Ros A.; Schroder, Fritz; Tomlins, Scott A.; Tannock, Ian; Drake, Charles G.; de Bono, Johann S.	LANCET	2016	387	10013	434	86,8
Targeting metastasis	Steeg, Patricia S.	NATURE REVIEWS CANCER	2016	16	4	413	82,6
Emerging mechanisms of resistance to androgen receptor inhibitors in prostate cancer	Watson, Philip A.; Arora, Vivek K.; Sawyers, Charles L.	NATURE REVIEWS CANCER	2015	15	12	407	67,83

Prostate Cancer, Version 1.2016 Featured Updates to the NCCN Guidelines	Mohler, James L.; Armstrong, Andrew J.; Bahnson, Robert R.; D'Amico, Anthony Victor; Davis, Brian J.; Eastham, James A.; Enke, Charles A.; Farrington, Thomas A.; Higano, Celestia S.; Horwitz, Eric M.; Hurwitz, Michael; Kane, Christopher J.; Kawachi, Mark H.; Kuettel, Michael; Lee, Richard J.; Meeks, Joshua J.; Penson, David F.; Plimack, Elizabeth R.; Pow-Sang, Julio M.; Raben, David; Richey, Sylvia; Roach, Mack, III; Rosenfeld, Stan; Schaeffer, Edward; Skolarus, Ted A.; Small, Eric J.; Sonpavde, Guru; Srinivas, Sandy; Strope, Seth A.; Tward, Jonathan; Shead, Dorothy A.; Freedman-Cass, Deborah A.	JOURNAL OF THE NATIONAL COMPREHENSIVE CANCER NETWORK	2016	14	1	393	78,6
Emerging applications of metabolomics in drug discovery and precision medicine	Wishart, David S.	NATURE REVIEWS DRUG DISCOVERY	2016	15	7	349	69,8
Mechanisms of long noncoding RNA function in development and disease	Schmitz, Sandra U.; Grote, Phillip; Herrmann, Bernhard G.	CELLULAR AND MOLECULAR LIFE SCIENCES	2016	73	13	286	57,2
Substantial interindividual and limited intraindividual genomic diversity among tumors from men with metastatic prostate cancer	Kumar, Akash; Coleman, Ilsa; Morrissey, Colm; Zhang, Xiaotun; True, Lawrence D.; Gulati, Roman; Etzioni, Ruth; Bolouri, Hamid; Montgomery, Bruce; White, Thomas; Lucas, Jared M.; Brown, Lisha G.; Dumpit, Ruth F.; DeSarkar, Navonil; Higano, Celestia; Yu, Evan Y.; Coleman, Roger; Schultz, Nikolaus; Fang, Min; Lange, Paul H.; Shendure, Jay; Vessella, Robert L.; Nelson, Peter S.	NATURE MEDICINE	2016	22	4	267	53,4

Androgen Receptor Gene Aberrations in Circulating Cell-Free DNA: Biomarkers of Therapeutic Resistance in Castration-Resistant Prostate Cancer	Azad, Arun A.; Volik, Stanislav V.; Wyatt, Alexander W.; Haegert, Anne; Le Bihan, Stephane; Bell, Robert H.; Anderson, Shawn A.; McConeghy, Brian; Shukin, Robert; Bazov, Jenny; Youngren, Jack; Paris, Pamela; Thomas, George; Small, Eric J.; Wang, Yuzhuo; Gleave, Martin E.; Collins, Colin C.; Chi, Kim N.	CLINICAL CANCER RESEARCH	2015	21	10	236	39,33
Plasma AR and abiraterone-resistant prostate cancer	Romanel, Alessandro; Tandefelt, Delila Gasi; Conteduca, Vincenza; Jayaram, Anuradha; Casiraghi, Nicola; Wetterskog, Daniel; Salvi, Samanta; Amadori, Dino; Zafeiriou, Zafeiris; Rescigno, Pasquale; Bianchini, Diletta; Gurioli, Giorgia; Casadio, Valentina; Carreira, Suzanne; Goodall, Jane; Wingate, Anna; Ferraldeschi, Roberta; Tunariu, Nina; Flohr, Penny; De Giorgi, Ugo; de Bono, Johann S.; Demichelis, Francesca; Attard, Gerhardt	SCIENCE TRANSLATIONAL MEDICINE	2015	7	312	225	37,5

Spatial genomic heterogeneity within localized, multifocal prostate cancer	Boutros, Paul C.; Fraser, Michael; Harding, Nicholas J.; de Borja, Richard; Trudel, Dominique; Lalonde, Emilie; Meng, Alice; Hennings-Yeomans, Pablo H.; McPherson, Andrew; Sabelnykova, Veronica Y.; Zia, Amin; Fox, Natalie S.; Livingstone, Julie; Shiah, Yu-Jia; Wang, Jianxin; Beck, Timothy A.; Have, Cherry L.; Chong, Taryne; Sam, Michelle; Johns, Jeremy; Timms, Lee; Buchner, Nicholas; Wong, Ada; Watson, John D.; Simmons, Trent T.; P'ng, Christine; Zafarana, Gaetano; Nguyen, Francis; Luo, Xuemei; Chu, Kenneth C.; Prokopec, Stephenie D.; Sykes, Jenna; Dal Pra, Alan; Berlin, Alejandro; Brown, Andrew; Chan-Seng-Yue, Michelle A.; Yousif, Fouad; Denroche, Robert E.; Chong, Lauren C.; Chen, Gregory M.; Jung, Esther; Fung, Clement; Starmans, Maud H. W.; Chen, Hanbo; Govind, Shaylan K.; Hawley, James; D'Costa, Alister; Pintilie, Melania; Waggott, Daryl; Hach, Faraz; Lambin, Philippe; Muthuswamy, Lakshmi B.; Cooper, Colin; Eeles, Rosalind; Neal, David; Tetu, Bernard; Sahinalp, Cenk; Stein, Lincoln D.; Fleshner, Neil; Shah, Sohrab P.; Collins, Colin C.; Hudson, Thomas J.; McPherson, John D.; van der Kwast, Theodorus; Bristow, Robert G.	NATURE GENETICS	2015	47	7	219	36,5
Sphingolipids and their metabolism in physiology and disease	Hannun, Yusuf A.; Obeid, Lina M.	NATURE REVIEWS MOLECULAR CELL BIOLOGY	2018	19	3	216	72

Analysis of the genetic phylogeny of multifocal prostate cancer identifies multiple independent clonal expansions in neoplastic and morphologically normal prostate tissue	Cooper, Colin S.; Eeles, Rosalind; Wedge, David C.; Van Loo, Peter; Gundem, Gunes; Alexandrov, Ludmil B.; Kremeyer, Barbara; Butler, Adam; Lynch, Andrew G.; Camacho, Niedzica; Massie, Charlie E.; Kay, Jonathan; Luxton, Hayley J.; Edwards, Sandra; Kote-Jarai, Zsofia; Dennis, Nening; Merson, Sue; Leongamornlert, Daniel; Zamora, Jorge; Corbishley, Cathy; Thomas, Sarah; Nik-Zainal, Serena; O'Meara, Sarah; Matthews, Lucy; Clark, Jeremy; Hurst, Rachel; Mithen, Richard; Bristow, Robert G.; Boutros, Paul C.; Fraser, Michael; Cooke, Susanna; Raine, Keiran; Jones, David; Menzies, Andrew; Stebbings, Lucy; Hinton, Jon; Teague, Jon; McLaren, Stuart; Mudie, Laura; Hardy, Claire; Anderson, Elizabeth; Joseph, Olivia; Goody, Victoria; Robinson, Ben; Maddison, Mark; Gamble, Stephen; Greenman, Christopher; Berney, Dan; Hazell, Steven; Livni, Naomi; Fisher, Cyril; Ogden, Christopher; Kumar, Pardeep; Thompson, Alan; Woodhouse, Christopher; Nicol, David; Mayer, Erik; Dudderidge, Tim; Shah, Nimish C.; Gnanapragasam, Vincent; Voet, Thierry; Campbell, Peter; Futreal, Andrew; Easton, Douglas; Warren, Anne Y.; Foster, Christopher S.; Stratton, Michael R.; Whitaker, Hayley C.; McDermott, Ultan; Brewer, Daniel S.; Neal, David E.	NATURE GENETICS	2015	47	4	215	35,83
Differentiation of mammary tumors and reduction in metastasis upon Malat1 lncRNA loss	Arun, Gayatri; Diermeier, Sarah; Akerman, Martin; Chang, Kung-Chi; Wilkinson, J. Erby; Hearn, Stephen; Kim, Youngsoo; MacLeod, A. Robert; Krainer, Adrian R.; Norton, Larry; Brogi, Edi; Egeblad, Mikala; Spector, David L.	GENES & DEVELOPMENT	2016	30	1	212	42,4

Long noncoding RNAs in cancer: mechanisms of action and technological advancements	Bartonicek, Nenad; Maag, Jesper L. V.; Dinger, Marcel E.	MOLECULAR CANCER	2016	15		206	41,2
Integrative clinical genomics of metastatic cancer	Robinson, Dan R.; Wu, Yi-Mi; Lonigro, Robert J.; Vats, Pankaj; Cobain, Erin; Everett, Jessica; Cao, Xuhong; Rabban, Erica; Kumar-Sinha, Chandan; Raymond, Victoria; Schuetze, Scott; Alva, Ajjai; Siddiqui, Javed; Chugh, Rashmi; Worden, Francis; Zalupski, Mark M.; Innis, Jeffrey; Mody, Rajen J.; Tomlins, Scott A. .; Lucas, David; Baker, Laurence H.; Ramnath, Nithya; Schott, Ann F.; Hayes, Daniel F.; Vijai, Joseph; Offit, Kenneth; Stoffel, Elena M.; Roberts, J. Scott; Smith, David C.; Kunju, Lakshmi P.; Talpaz, Moshe; Cieslik, Marcin; Chinnaiyan, Arul M.	NATURE	2017	548	7667	202	50,5
Tracking the origins and drivers of subclonal metastatic expansion in prostate cancer	Hong, Matthew K. H.; Macintyre, Geoff; Wedge, David C.; Van Loo, Peter; Patel, Keval; Lunke, Sebastian; Alexandrov, Ludmil B.; Sloggett, Clare; Cmero, Marek; Marass, Francesco; Tsui, Dana; Mangiola, Stefano; Lonie, Andrew; Naeem, Haroon; Sapre, Nikhil; Phal, Pramit M.; Kurganovs, Natalie; Chin, Xiaowen; Kerger, Michael; Warren, Anne Y.; Neal, David; Gnanapragasam, Vincent; Rosenfeld, Nitzan; Pedersen, John S.; Ryan, Andrew; Haviv, Izhak; Costello, Anthony J.; Corcoran, Niall M.; Hovens, Christopher M.	NATURE COMMUNICATION S	2015	6		189	31,5
Abscopal effects of radiation therapy: A clinical review for the radiobiologist	Siva, Shankar; MacManus, Michael P.; Martin, Roger F.; Martin, Olga A.	CANCER LETTERS	2015	356	1	183	30,5

Genomic hallmarks of localized, non-indolent prostate cancer	Fraser, Michael; Sabelnykova, Veronica Y.; Yamaguchi, Takafumi N.; Heisler, Lawrence E.; Livingstone, Julie; Huang, Vincent; Shiah, Yu-Jia; Yousif, Fouad; Lin, Xihui; Masella, Andre P.; Fox, Natalie S.; Xie, Michael; Prokopec, Stephenie D.; Berlin, Alejandro; Lalonde, Emilie; Ahmed, Musaddeque; Trudel, Dominique; Luo, Xuemei; Beck, Timothy A.; Meng, Alice; Zhang, Junyan; D'Costa, Alister; Denroche, Robert E.; Kong, Haiying; Espiritu, Shadrielle Melijah G.; Chua, Melvin L. K.; Wong, Ada; Chong, Taryne; Sam, Michelle; Johns, Jeremy; Timms, Lee; Buchner, Nicholas B.; Orain, Michele; Picard, Valerie; Hovington, Helene; Murison, Alexander; Kron, Ken; Harding, Nicholas J.; P'ng, Christine; Houlahan, Kathleen E.; Chu, Kenneth C.; Lo, Bryan; Francis Nguyen; Li, Constance H.; Sun, Ren X.; de Borja, Richard; Cooper, Christopher I.; Hopkins, Julia F.; Govind, Shaylan K.; Fung, Clement; Waggott, Daryl; Green, Jeffrey; Haider, Syed; Chan-Seng-Yue, Michelle A.; Jung, Esther; Wang, Zhiyuan; Bergeron, Alain; Dal Pra, Alan; Lacombe, Louis; Collins, Colin C.; Sahinalp, Cenk; Lupien, Mathieu; Fleshner, Neil E.; He, Housheng H.; Fradet, Yves; Tetu, Bernard; van der Kwast, Theodorus; McPherson, John D.; Bristow, Robert G.; Boutros, Paul C.	NATURE	2017	541	7637	175	43,75

The long tail of oncogenic drivers in prostate cancer	Armenia, Joshua; Wankowicz, Stephanie A. M.; Liu, David; Gao, Jianjiong; Kundra, Ritika; Reznik, Ed; Chatila, Walid K.; Chakravarty, Debyani; Han, G. Celine; Coleman, Ilsa; Montgomery, Bruce; Pritchard, Colin; Morrissey, Colm; Barbieri, Christopher E.; Beltran, Himisha; Sboner, Andrea; Zafeiriou, Zafeiris; Miranda, Susana; Bielski, Craig M.; Penson, Alexander V.; Tolonen, Charlotte; Huang, Franklin W.; Robinson, Dan; Wu, Yi Mi; Lonigro, Robert; Garraway, Levi A.; Demichelis, Francesca; Kantoff, Philip W.; Taplin, Mary-Ellen; Abida, Wassim; Taylor, Barry S.; Scher, Howard I.; Nelson, Peter S.; de Bono, Johann S.; Rubin, Mark A.; Sawyers, Charles L.; Chinnaiyan, Arul M.; Schultz, Nikolaus; Van Allen, Eliezer M.	NATURE GENETICS	2018	50	5	161	53,67
Targeting YAP-Dependent MDSC Infiltration Impairs Tumor Progression	Wang, Guocan; Lu, Xin; Dey, Prasenjit; Deng, Pingna; Wu, Chia Chin; Jiang, Shan; Fang, Zhuangna; Zhao, Kuo; Konaparthi, Ramakrishna; Hua, Sujun; Zhang, Jianhua; Li-Ning-Tapia, Elsa M.; Kapoor, Avnish; Wu, Chang-Jiun; Patel, Neelay Bhaskar; Guo, Zhenglin; Ramamoorthy, Vandhana; Tieu, Trang N.; Heffernan, Tim; Zhao, Di; Shang, Xiaoying; Khadka, Sunada; Hou, Pingping; Hu, Baoli; Jin, Eun-Jung; Yao, Wantong; Pan, Xiaolu; Ding, Zhihu; Shi, Yanxia; Li, Liren; Chang, Qing; Troncoso, Patricia; Logethetis, Christopher J.; McArthur, Mark J.; Chin, Lynda; Wang, Y. Alan; DePinho, Ronaldo A.	CANCER DISCOVERY	2016	6	1	149	29,8

MethHC: a database of DNA methylation and gene expression in human cancer	Huang, Wei-Yun; Hsu, Sheng-Da; Huang, Hsi-Yuan; Sun, Yi-Ming; Chou, Chih-Hung; Weng, Shun-Long; Huang, Hsien-Da	NUCLEIC ACIDS RESEARCH	2015	43	D1	149	24,83
Long Noncoding RNA in Cancer: Wiring Signaling Circuitry	Lin, Chunru; Yang, Liuqing	TRENDS IN CELL BIOLOGY	2018	28	4	145	48,33
Implementing Genome-Driven Oncology	Hyman, David M.; Taylor, Barry S.; Baselga, Jose	CELL	2017	168	4	145	36,25
A Biopsy-based 17-gene Genomic Prostate Score Predicts Recurrence After Radical Prostatectomy and Adverse Surgical Pathology in a Racially Diverse Population of Men with Clinically Low- and Intermediate-risk Prostate Cancer	Cullen, Jennifer; Rosner, Inger L.; Brand, Timothy C.; Zhang, Nan; Tsiatis, Athanasios C.; Moncur, Joel; Ali, Amina; Chen, Yongmei; Knezevic, Dejan; Maddala, Tara; Lawrence, H. Jeffrey; Febbo, Phillip G.; Srivastava, Shiv; Sesterhenn, Isabell A.; McLeod, David G.	EUROPEAN UROLOGY	2015	68	1	141	23,5
Active Surveillance for the Management of Localized Prostate Cancer (Cancer Care Ontario Guideline): American Society of Clinical Oncology Clinical Practice Guideline Endorsement	Chen, Ronald C.; Rumble, R. Bryan; Loblaw, D. Andrew; Finelli, Antonio; Ehdaie, Behfar; Cooperberg, Matthew R.; Morgan, Scott C.; Tyldesley, Scott; Haluschak, John J.; Tan, Winston; Justman, Stewart; Jain, Suneil	JOURNAL OF CLINICAL ONCOLOGY	2016	34	18	140	28
The Landscape of Circular RNA in Cancer	Vo, Josh N.; Cieslik, Marcin; Zhang, Yajia; Shukla, Sudhanshu; Xiao, Lanbo; Zhang, Yuping; Wu, Yi-Mi; Dhanasekaran, Saravana M.; Engelke, Carl G.; Cao, Xuhong; Robinson, Dan R.; Nesvizhskii, Alexey I.; Chinnaiyan, Arul M.	CELL	2019	176	4	137	68,5
Current and future perspectives of liquid biopsies in genomics-driven oncology	Heitzer, Ellen; Haque, Imran S.; Roberts, Charles E. S.; Speicher, Michael R.	NATURE REVIEWS GENETICS	2019	20	2	136	68

Exosomes secreted under hypoxia enhance invasiveness and stemness of prostate cancer cells by targeting adherens junction molecules	Ramteke, Anand; Ting, Harold; Agarwal, Chapla; Mateen, Samiha; Somasagara, Ranganathan; Hussain, Anowar; Graner, Michael; Frederick, Barbara; Agarwal, Rajesh; Deep, Gagan	MOLECULAR CARCINOGENESIS	2015	54	7	135	22,5
Genomic Alterations in Cell-Free DNA and Enzalutamide Resistance in Castration-Resistant Prostate Cancer	Wyatt, Alexander W.; Azad, Arun A.; Volik, Stanislav V.; Annala, Matti; Beja, Kevin; McConeghy, Brian; Haegert, Anne; Warner, Evan W.; Mo, Fan; Brahmbhatt, Sonal; Shukin, Robert; Le Bihan, Stephane; Gleave, Martin E.; Nykter, Matti; Collins, Colin C.; Chi, Kim N.	JAMA ONCOLOGY	2016	2	12	132	26,4
Retinoic acid receptors: From molecular mechanisms to cancer therapy	di Masi, Alessandra; Leboffe, Loris; De Marinis, Elisabetta; Pagano, Francesca; Cicconi, Laura; Rochette-Egly, Cecile; Lo-Coco, Francesco; Ascenzi, Paolo; Nervi, Clara	MOLECULAR ASPECTS OF MEDICINE	2015	41		130	21,67
Understanding the Mechanisms of Androgen Deprivation Resistance in Prostate Cancer at the Molecular Level	Karantanos, Theodoros; Evans, Christopher P.; Tombal, Bertrand; Thompson, Timothy C.; Montironi, Rodolfo; Isaacs, William B.	EUROPEAN UROLOGY	2015	67	3	129	21,5
Kinase-targeted cancer therapies: progress, challenges and future directions	Bhullar, Khushwant S.; Orrego Lagaron, Naiara; McGowan, Eileen M.; Parmar, Indu; Jha, Amitabh; Hubbard, Basil P.; Rupasinghe, H. P. Vasantha	MOLECULAR CANCER	2018	17		128	42,67
Genomic Classifier Identifies Men With Adverse Pathology After Radical Prostatectomy Who Benefit From Adjuvant Radiation Therapy	Den, Robert B.; Yousefi, Kasra; Trabulsi, Edouard J.; Abdollah, Firas; Choeurng, Voleak; Feng, Felix Y.; Dicker, Adam P.; Lallas, Costas D.; Gomella, Leonard G.; Davicioni, Elai; Karnes, R. Jeffrey	JOURNAL OF CLINICAL ONCOLOGY	2015	33	8	127	21,17
The Role of Cholesterol in Cancer	Kuzu, Omer F.; Noory, Mohammad A.; Robertson, Gavin P.	CANCER RESEARCH	2016	76	8	126	25,2

Large oncosomes contain distinct protein cargo and represent a separate functional class of tumor-derived extracellular vesicles	Minciacchi, Valentina R.; You, Sungyong; Spinelli, Cristiana; Morley, Samantha; Zandian, Mandana; Aspuria, Paul-Joseph; Cavallini, Lorenzo; Ciardiello, Chiara; Sobreiro, Mariana Reis; Morello, Matteo; Kharmate, Geetanjali; Jang, Su Chul; Kim, Dae-Kyum; Hosseini-Beheshti, Elham; Guns, Emma Tomlinson; Gleave, Martin; Gho, Yong Song; Mathivanan, Suresh; Yang, Wei; Freeman, Michael R.; Di Vizio, Dolores	ONCOTARGET	2015	6	13	120	20
Galectin expression in cancer diagnosis and prognosis: A systematic review	Thijssen, Victor L.; Heusschen, Roy; Caers, Jo; Griffioen, Arjan W.	BIOCHIMICA ET BIOPHYSICA ACTA-REVIEWS ON CANCER	2015	1855	2	116	19,33
Anticancer Molecular Mechanisms of Resveratrol	Varoni, Elena M.; Lo Faro, Alfredo Fabrizio; Sharifi-Rad, Javad; Iriti, Marcello	FRONTIERS IN NUTRITION	2016	3		115	23
Development and Validation of a Scalable Next-Generation Sequencing System for Assessing Relevant Somatic Variants in Solid Tumors	Hovelson, Daniel H.; McDaniel, Andrew S.; Cani, Andi K.; Johnson, Bryan; Rhodes, Kate; Williams, Paul D.; Bandla, Santhoshi; Bien, Geoffrey; Choppa, Paul; Hyland, Fiona; Gottimukkala, Rajesh; Liu, Guoying; Manivannan, Manimozhi; Schageman, Jeoffrey; Ballesteros-Villagrana, Efren; Grasso, Catherine S.; Quist, Michael J.; Yadati, Venkata; Amin, Anmol; Siddiqui, Javed; Betz, Bryan L.; Knudsen, Karen E.; Cooney, Kathleen A.; Feng, Felix Y.; Roh, Michael H.; Nelson, Peter S.; Liu, Chia-Jen; Beer, David G.; Wyngaard, Peter; Chinnaiyan, Arul M.; Sadis, Seth; Rhodes, Daniel R.; Tomlins, Scott A.	NEOPLASIA	2015	17	4	115	19,17
Long Noncoding RNAs in Cancer: From Function to Translation	Sahu, Anirban; Singhal, Udit; Chinnaiyan, Arul M.	TRENDS IN CANCER	2015	1	2	113	18,83

Cell-free DNA (cfDNA): Clinical Significance and Utility in Cancer Shaped By Emerging Technologies	Volik, Stanislav; Alcaide, Miguel; Morin, Ryan D.; Collins, Colin	MOLECULAR CANCER RESEARCH	2016	14	10	110	22
Circulating Tumor DNA Genomics Correlate with Resistance to Abiraterone and Enzalutamide in Prostate Cancer	Annala, Matti; Vandekerkhove, Gillian; Khalaf, Daniel; Taavitsainen, Sinja; Beja, Kevin; Warner, Evan W.; Sunderland, Katherine; Kollmannsberger, Christian; Eigl, Bernhard J.; Finch, Daygen; Oja, Conrad D.; Vergidis, Joanna; Zulfiqar, Muhammad; Azad, Arun A.; Nykter, Matti; Gleave, Martin E.; Wyatt, Alexander W.; Chi, Kim N.	CANCER DISCOVERY	2018	8	4	108	36
Oligometastatic prostate cancer: definitions, clinical outcomes, and treatment considerations	Tosoian, Jeffrey J.; Gorin, Michael A.; Ross, Ashley E.; Pienta, Kenneth J.; Tran, Phuoc T.; Schaeffer, Edward M.	NATURE REVIEWS UROLOGY	2017	14	1	108	27
Precision diagnostics: moving towards protein biomarker signatures of clinical utility in cancer	Borrebaeck, Carl A. K.	NATURE REVIEWS CANCER	2017	17	3	103	25,75
Genomic Predictors of Outcome in Prostate Cancer	Bostrom, Peter J.; Bjartell, Anders S.; Catto, James W. F.; Eggener, Scott E.; Lilja, Hans; Loeb, Stacy; Schalken, Jack; Schlomm, Thorsten; Cooperberg, Matthew R.	EUROPEAN UROLOGY	2015	68	6	103	17,17
Tissue-based Genomics Augments Post-prostatectomy Risk Stratification in a Natural History Cohort of Intermediate- and High-Risk Men	Ross, Ashley E.; Johnson, Michael H.; Yousefi, Kasra; Davicioni, Elai; Netto, George J.; Marchionni, Luigi; Fedor, Helen L.; Glavaris, Stephanie; Choeurng, Voleak; Buerki, Christine; Erho, Nicholas; Lam, Lucia L.; Humphreys, Elizabeth B.; Faraj, Sheila; Bezerra, Stephania M.; Han, Misop; Partin, Alan W.; Trock, Bruce J.; Schaeffer, Edward M.	EUROPEAN UROLOGY	2016	69	1	102	20,4

Combined Value of Validated Clinical and Genomic Risk Stratification Tools for Predicting Prostate Cancer Mortality in a High-risk Prostatectomy Cohort	Cooperberg, Matthew R.; Davicioni, Elai; Crisan, Anamaria; Jenkins, Robert B.; Ghadessi, Mercedeh; Karnes, R. Jeffrey	EUROPEAN UROLOGY	2015	67	2	102	17
Using data-independent, high-resolution mass spectrometry in protein biomarker research: Perspectives and clinical applications	Sajic, Tatjana; Liu, Yansheng; Aebersold, Ruedi	PROTEOMICS CLINICAL APPLICATIONS	2015	9	3-4	101	16,83
Cholesterol uptake disruption, in association with chemotherapy, is a promising combined metabolic therapy for pancreatic adenocarcinoma	Guillaumond, Fabienne; Bidaut, Ghislain; Ouaissi, Mehdi; Servais, Stephane; Gouirand, Victoire; Olivares, Orianne; Lac, Sophie; Borge, Laurence; Roques, Julie; Gayet, Odile; Pinault, Michelle; Guimaraes, Cyrille; Nigri, Jeremy; Loncle, Celine; Lavaut, Marie-Noelle; Garcia, Stephane; Tailleux, Anne; Staels, Bart; Calvo, Ezequiel; Tomasini, Richard; Iovanna, Juan Lucio; Vasseur, Sophie	PROCEEDINGS OF THE NATIONAL ACADEMY OF SCIENCES OF THE UNITED STATES OF AMERICA	2015	112	8	101	16,83
Long noncoding RNAs and tumorigenesis: genetic associations, molecular mechanisms, and therapeutic strategies	Zhang, Fan; Zhang, Liang; Zhang, Caiguo	TUMOR BIOLOGY	2016	37	1	100	20
Mindfulness Meditation for Younger Breast Cancer Survivors: A Randomized Controlled Trial	Bower, Julienne E.; Crosswell, Alexandra D.; Stanton, Annette L.; Crespi, Catherine M.; Winston, Diana; Arevalo, Jesusa; Ma, Jeffrey; Cole, Steve W.; Ganz, Patricia A.	CANCER	2015	121	8	100	16,67
Targeted protein degradation by PROTACs	Neklesa, Taavi K.; Winkler, James D.; Crews, Craig M.	PHARMACOLOGY & THERAPEUTICS	2017	174		99	24,75

Breast Cancer Risk From Modifiable and Nonmodifiable Risk Factors Among White Women in the United States	Maas, Paige; Barrdahl, Myrto; Joshi, Amit D.; Auer, Paul L.; Gaudet, Mia M.; Milne, Roger L.; Schumacher, Fredrick R.; Anderson, William F.; Check, David; Chattopadhyay, Subham; Baglietto, Laura; Berg, Christine D.; Chanock, Stephen J.; Cox, David G.; Figueroa, Jonine D.; Gail, Mitchell H.; Graubard, Barry I.; Haiman, Christopher A.; Hankinson, Susan E.; Hoover, Robert N.; Isaacs, Claudine; Kolonel, Laurence N.; Le Marchand, Loic; Lee, I-Min; Lindstrom, Sara; Overvad, Kim; Romieu, Isabelle; Sanchez, Maria-Jose; Southey, Melissa C.; Stram, Daniel O.; Tumino, Rosario; VanderWeele, Tyler J.; Willett, Walter C.; Zhang, Shumin; Buring, Julie E.; Canzian, Federico; Gapstur, Susan M.; Henderson, Brian E.; Hunter, David J.; Giles, Graham G.; Prentice, Ross L.; Ziegler, Regina G.; Kraft, Peter; Garcia-Closas, Montse; Chatterjee, Nilanjan	JAMA ONCOLOGY	2016	2	10	98	19,6

The OncoArray Consortium: A Network for Understanding the Genetic Architecture of Common Cancers	Amos, Christopher I.; Dennis, Joe; Wang, Zhaoming; Byun, Jinyoung; Schumacher, Fredrick R.; Gayther, Simon A.; Casey, Graham; Hunter, David J.; Sellers, Thomas A.; Gruber, Stephen B.; Dunning, Alison M.; Michailidou, Kyriaki; Fachal, Laura; Doheny, Kimberly; Spurdle, Amanda B.; Li, Yafang; Xiao, Xiangjun; Romm, Jane; Pugh, Elizabeth; Coetzee, Gerhard A.; Hazelett, Dennis J.; Bojesen, Stig E.; Caga-Anan, Charlisse; Haiman, Christopher A.; Kamal, Ahsan; Luccarini, Craig; Tessier, Daniel; Vincent, Daniel; Bacot, Francois; Van den Berg, David J.; Nelson, Stefanie; Demetriades, Stephen; Goldgar, David E.; Couch, Fergus J.; Forman, Judith L.; Giles, Graham G.; Conti, David V.; Bickeboeller, Heike; Risch, Angela; Waldenberger, Melanie; Brueske-Hohlfeld, Irene; Hicks, Belynda D.; Ling, Hua; McGuffog, Lesley; Lee, Andrew; Kuchenbaecker, Karoline; Soucy, Penny; Manz, Judith; Cunningham, Julie M.; Butterbach, Katja; Kote-Jarai, Zsofia; Kraft, Peter; FitzGerald, Liesel; Lindstrom, Sara; Adams, Marcia; McKay, James D.; Phelan, Catherine M.; Benlloch, Sara; Kelemen, Linda E.; Brennan, Paul; Riggan, Marjorie; O'Mara, Tracy A.; Shen, Hongbing; Shi, Yongyong; Thompson, Deborah J.; Goodman, Marc T.; Nielsen, Sune F.; Berchuck, Andrew; Laboissiere, Sylvie; Schmit, Stephanie L.; Shelford,	CANCER EPIDEMIOLOGY BIOMARKERS & PREVENTION	2017	26	1	96	24

A Genomic Classifier Improves Prediction of Metastatic Disease Within 5 Years After Surgery in Node-negative High-risk Prostate Cancer Patients Managed by Radical Prostatectomy Without Adjuvant Therapy	Klein, Eric A.; Yousefi, Kasra; Haddad, Zaid; Choeurng, Voleak; Buerki, Christine; Stephenson, Andrew J.; Li, Jianbo; Kattan, Michael W.; Magi-Galluzzi, Cristina; Davicioni, Elai	EUROPEAN UROLOGY	2015	67	4	96	16
A common classification framework for neuroendocrine neoplasms: an International Agency for Research on Cancer (IARC) and World Health Organization (WHO) expert consensus proposal	Rindi, Guido; Klimstra, David S.; Abedi-Ardekani, Behnoush; Asa, Sylvia L.; Bosman, Frederik T.; Brambilla, Elisabeth; Busam, Klaus J.; de Krijger, Ronald R.; Dietel, Manfred; El-Naggar, Adel K.; Fernandez-Cuesta, Lynnette; Kloeppel, Guenter; McCluggage, W. Glenn; Moch, Holger; Ohgaki, Hiroko; Rakha, Emad A.; Reed, Nicholas S.; Rous, Brian A.; Sasano, Hironobu; Scarpa, Aldo; Scoazec, Jean-Yves; Travis, William D.; Tallini, Giovanni; Trouillas, Jacqueline; van Krieken, J. Han; Cree, Ian A.	MODERN PATHOLOGY	2018	31	12	95	31,67
Subclonal Genomic Architectures of Primary and Metastatic Colorectal Cancer Based on Intratumoral Genetic Heterogeneity	Kim, Tae-Min; Jung, Seung-Hyun; An, Chang Hyeok; Lee, Sung Hak; Baek, In-Pyo; Kim, Min Sung; Park, Sung-Won; Rhee, Je-Keun; Lee, Sug-Hyung; Chung, Yeun-Jun	CLINICAL CANCER RESEARCH	2015	21	19	94	15,67
Augmenting antitumor immune responses with epigenetic modifying agents	Heninger, Erika; Krueger, Timothy E. G.; Lang, Joshua M.	FRONTIERS IN IMMUNOLOGY	2015	6		94	15,67

Clinical and Genomic Characterization of Treatment-Emergent Small-Cell Neuroendocrine Prostate Cancer: A Multi-institutional Prospective Study	Aggarwal, Rahul; Huang, Jiaoti; Alumkal, Joshi J.; Zhang, Li; Feng, Felix Y.; Thomas, George V.; Weinstein, Alana S.; Friedl, Verena; Zhang, Can; Witte, Owen N.; Lloyd, Paul; Gleave, Martin; Evans, Christopher P.; Youngren, Jack; Beer, Tomasz M.; Rettig, Matthew; Wong, Christopher K.; True, Lawrence; Foye, Adam; Playdle, Denise; Ryan, Charles J.; Lara, Primo; Chi, Kim N.; Uzunangelov, Vlado; Sokolov, Artem; Newton, Yulia; Beltran, Himisha; Demichelis, Francesca; Rubin, Mark A.; Stuart, Joshua M.; Small, Eric J.	JOURNAL OF CLINICAL ONCOLOGY	2018	36	24	93	31
Genomic instability in human cancer: Molecular insights and opportunities for therapeutic attack and prevention through diet and nutrition	Ferguson, Lynnette R.; Chen, Helen; Collins, Andrew R.; Connell, Marisa; Damia, Giovanna; Dasgupta, Santanu; Malhotra, Meenakshi; Meeker, Alan K.; Amedei, Amedeo; Amin, Amr; Ashraf, S. Salman; Aquilano, Katia; Azmi, Asfar S.; Bhakta, Dipita; Bilsland, Alan; Boosani, Chandra S.; Chen, Sophie; Ciriolo, Maria Rosa; Fujii, Hiromasa; Guha, Gunjan; Halicka, Dorota; Helferich, William G.; Keith, W. Nicol; Mohammed, Sulma I.; Niccolai, Elena; Yang, Xujuan; Honoki, Kanya; Parslow, Virginia R.; Prakash, Satya; Rezazadeh, Sarallah; Shackelford, Rodney E.; Sidransky, David; Tran, Phuoc T.; Yang, Eddy S.; Maxwell, Christopher A.	SEMINARS IN CANCER BIOLOGY	2015	35		93	15,5
SUMOylation-Mediated Regulation of Cell Cycle Progression anc Cancer	Eifler, Karolin; Vertegaal, Alfred C. O.	TRENDS IN BIOCHEMICAL SCIENCES	2015	40	12	93	15,5
Therapeutic Targeting of Long Non-Coding RNAs in Cancer	Arun, Gayatri; Diermeier, Sarah D.; Spector, David L.	TRENDS IN MOLECULAR MEDICINE	2018	24	3	91	30,33

Clinical implications of PTEN loss in prostate cancer	Jamaspishvili, Tamara; Berman, David M.; Ross, Ashley E.; Scher, Howard I.; De Marzo, Angelo M.; Squire, Jeremy A.; Lotan, Tamara L.	NATURE REVIEWS UROLOGY	2018	15	4	90	30
Landscape of Phosphatidylinositol-3-Kinase Pathway Alterations Across 19 784 Diverse Solid Tumors	Millis, Sherri Z.; Ikeda, Sadakatsu; Reddy, Sandeep; Gatalica, Zoran; Kurzrock, Razelle	JAMA ONCOLOGY	2016	2	12	89	17,8
The role of exosomes and miRNAs in drug-resistance of cancer cells	Bach, Duc-Hiep; Hong, Ji-Young; Park, Hyen Joo; Lee, Sang Kook	INTERNATIONAL JOURNAL OF CANCER	2017	141	2	86	21,5
Population-based multicase-control study in common tumors in Spain (MCC-Spain): rationale and study design	Castano-Vinyals, Gemma; Aragones, Nuria; Perez-Gomez, Beatriz; Martin, Vicente; Llorca, Javier; Moreno, Victor; Altzibar, Jone M.; Ardanaz, Eva; de Sanjose, Silvia; Juan Jimenez-Moleon, Jose; Tardon, Adonina; Alguacil, Juan; Peiro, Rosana; Marcos-Gragera, Rafael; Navarro, Carmen; Pollan, Marina; Kogevinas, Manolis	GACETA SANITARIA	2015	29	4	86	14,33
The long noncoding RNA Malat1: Its physiological and pathophysiological functions	Zhang, Xuejing; Hamblin, Milton H.; Yin, Ke-Jie	RNA BIOLOGY	2017	14	12	85	21,25
Integration of copy number and transcriptomics provides risk stratification in prostate cancer: A discovery and validation cohort study	Ross-Adams, H.; Lamb, A. D.; Dunning, M. J.; Halim, S.; Lindberg, J.; Massie, C. M.; Egevad, L. A.; Russell, R.; Ramos-Montoya, A.; Vowler, S. L.; Sharma, N. L.; Kay, J.; Whitaker, H.; Clark, J.; Hurst, R.; Gnanapragasam, V. J.; Shah, N. C.; Warren, A. Y.; Cooper, C. S.; Lynch, A. G.; Stark, R.; Mills, I. G.; Groenberg, H.; Neal, D. E.	EBIOMEDICINE	2015	2	9	85	14,17

Development and validation of a 24-gene predictor of response to postoperative radiotherapy in prostate cancer: a matched, retrospective analysis	Zhao, Shuang G.; Chang, S. Laura; Spratt, Daniel E.; Erho, Nicholas; Yu, Menggang; Ashab, Hussam Al-Deen; Alshalalfa, Mohammed; Speers, Corey; Tomlins, Scott A.; Davicioni, Elai; Dicker, Adam P.; Carroll, Peter R.; Cooperberg, Matthew R.; Freedland, Stephen J.; Karnes, R. Jeffrey; Ross, Ashley E.; Schaeffer, Edward M.; Den, Robert B.; Nguyen, Paul L.; Feng, Felix Y.	LANCET ONCOLOGY	2016	17	11	84	16,8
Identification of prostate cancer biomarkers in urinary exosomes	Overbye, Anders; Skotland, Tore; Koehler, Christian J.; Thiede, Bernd; Seierstad, Therese; Berge, Viktor; Sandvig, Kirsten; Llorente, Alicia	ONCOTARGET	2015	6	30	84	14
Technical challenges of working with extracellular vesicles	Ramirez, Marcel I.; Amorim, Maria G.; Gadelha, Catarina; Milic, Ivana; Welsh, Joshua A.; Freitas, Vanessa M.; Nawaz, Muhammad; Akbar, Naveed; Couch, Yvonne; Makin, Laura; Cooke, Fiona; Vettore, Andre L.; Batista, Patricia X.; Freezor, Roberta; Pezuk, Julia A.; Rosa-Fernandes, Livia; Carreira, Ana Claudia O.; Devitt, Andrew; Jacobs, Laura; Silva, Israel T.; Coakley, Gillian; Nunes, Diana N.; Carter, Dave; Palmisano, Giuseppe; Dias-Neto, Emmanuel	NANOSCALE	2018	10	3	83	27,67
MYC Deregulation in Primary Human Cancers	Kalkat, Manpreet; De Melo, Jason; Hickman, Katherine Ashley; Lourenco, Corey; Redel, Cornelia; Resetca, Diana; Tamachi, Aaliya; Tu, William B.; Penn, Linda Z.	GENES	2017	8	6	82	20,5
Genome-wide association studies of cancer: current insights and future perspectives	Sud, Amit; Kinnersley, Ben; Houlston, Richard S.	NATURE REVIEWS CANCER	2017	17	11	80	20
Tumour-suppressive microRNA-144-5p directly targets CCNE1/2 as potential prognostic markers in bladder cancer	Matsushita, R.; Seki, N.; Chiyomaru, T.; Inoguchi, S.; Ishihara, T.; Goto, Y.; Nishikawa, R.; Mataki, H.; Tatarano, S.; Itesako, T.; Nakagawa, M.; Enokida, H.	BRITISH JOURNAL OF CANCER	2015	113	2	80	13,33

PTEN loss is associated with upgrading of prostate cancer from biopsy to radical prostatectomy	Lotan, Tamara L.; Carvalho, Filipe L. F.; Peskoe, Sarah B.; Hicks, Jessica L.; Good, Jennifer; Fedor, Helen L.; Humphreys, Elizabeth; Han, Misop; Platz, Elizabeth A.; Squire, Jeremy A.; De Marzo, Angelo M.; Berman, David M.	MODERN PATHOLOGY	2015	28	1	80	13,33
Clinical utility of circulating non-coding RNAs - an update	Anfossi, Simone; Babayan, Anna; Pantel, Klaus; Calin, George A.	NATURE REVIEWS CLINICAL ONCOLOGY	2018	15	9	79	26,33
Androgen receptor variant-driven prostate cancer: clinical implications and therapeutic targeting	Antonarakis, E. S.; Armstrong, A. J.; Dehm, S. M.; Luo, J.	PROSTATE CANCER AND PROSTATIC DISEASES	2016	19	3	79	15,8
Decipher Genomic Classifier Measured on Prostate Biopsy Predicts Metastasis Risk	Klein, Eric A.; Haddad, Zaid; Yousefi, Kasra; Lam, Lucia L. C.; Wang, Qiqi; Choeurng, Voleak; Palmer-Aronsten, Beatrix; Buerki, Christine; Davicioni, Elai; Li, Jianbo; Kattan, Michael W.; Stephenson, Andrew J.; Magi-Galluzzi, Cristina	UROLOGY	2016	90		79	15,8
Update on Systemic Prostate Cancer Therapies: Management of Metastatic Castration-resistant Prostate Cancer in the Era of Precision Oncology	Nuhn, Philipp; De Bono, Johann S.; Fizazi, Karim; Freedland, Stephen J.; Grilli, Maurizio; Kantoff, Philip W.; Sonpavde, Guru; Sternberg, Cora N.; Yegnasubramanian, Srinivasan; Antonarakis, Emmanuel S.	EUROPEAN UROLOGY	2019	75	1	78	39
Inferring expressed genes by whole-genome sequencing of plasma DNA	Ulz, Peter; Thallinger, Gerhard G.; Auer, Martina; Graf, Ricarda; Kashofer, Karl; Jahn, Stephan W.; Abete, Luca; Pristauz, Ganda; Petru, Edgar; Geigl, Jochen B.; Heitzer, Ellen; Speicher, Michael R.	NATURE GENETICS	2016	48	10	78	15,6

Combined Tumor Suppressor Defects Characterize Clinically Defined Aggressive Variant Prostate Cancers	Aparicio, Ana M.; Shen, Li; Tapia, Elsa Li Ning; Lu, Jing-Fang; Chen, Hsiang-Chun; Zhang, Jiexin; Wu, Guanglin; Wang, Xuemei; Troncoso, Patricia; Corn, Paul; Thompson, Timothy C.; Broom, Bradley; Baggerly, Keith; Maity, Sankar N.; Logothetis, Christopher J.	CLINICAL CANCER RESEARCH	2016	22	6	78	15,6
Automated Multiplexed ECL Immunoarrays for Cancer Biomarker Proteins	Kadimisetty, Karteek; Malla, Spundana; Sardesai, Naimish P.; Joshi, Amit A.; Faria, Ronaldo C.; Lee, Norman H.; Rusling, James F.	ANALYTICAL CHEMISTRY	2015	87	8	78	13
DNA Repair in Prostate Cancer: Biology and Clinical Implications	Mateo, Joaquin; Boysen, Gunther; Barbieri, Christopher E.; Bryant, Helen E.; Castro, Elena; Nelson, Pete S.; Olmos, David; Pritchard, Colin C.; Rubin, Mark A.; de Bono, Johann S.	EUROPEAN UROLOGY	2017	71	3	77	19,25
Tumor-suppressive microRNA-223 inhibits cancer cell migration and invasion by targeting ITGA3/ITGB1 signaling in prostate cancer	Kurozumi, Akira; Goto, Yusuke; Matsushita, Ryosuke; Fukumoto, Ichiro; Kato, Mayuko; Nishikawa, Rika; Sakamoto, Shinichi; Enokida, Hideki; Nakagawa, Masayuki; Ichikawa, Tomohiko; Seki, Naohiko	CANCER SCIENCE	2016	107	1	77	15,4
MiRNA-based therapeutic intervention of cancer	Naidu, Srivatsava; Magee, Peter; Garofalo, Michela	JOURNAL OF HEMATOLOGY & ONCOLOGY	2015	8		77	12,83
Long Noncoding RNAs as New Architects in Cancer Epigenetics, Prognostic Biomarkers, and Potential Therapeutic Targets	Meseure, Didier; Alsibai, Kinan Drak; Nicolas, Andre; Bieche, Ivan; Morillon, Antonin	BIOMED RESEARCH INTERNATIONAL	2015	2015		77	12,83
Intratumoral and Intertumoral Genomic Heterogeneity of Multifocal Localized Prostate Cancer Impacts Molecular Classifications and Genomic Prognosticators	Wei, Lei; Wang, Jianmin; Lampert, Erika; Schlanger, Simon; DePriest, Adam D.; Hu, Qiang; Gomez, Eduardo Cortes; Murakam, Mitsuko; Glenn, Sean T.; Conroy, Jeffrey; Morrison, Carl; Azabdaftari, Gissou; Mohler, James L.; Liu, Song; Heemers, Hannelore V.	EUROPEAN UROLOGY	2017	71	2	76	19

miR-195 Inhibits Tumor Progression by Targeting RPS6KB1 in Human Prostate Cancer	Cai, Chao; Chen, Qing-Biao; Han, Zhao-Dong; Zhang, Yan-Qiong; He, Hui-Chan; Chen, Jia-Hong; Chen, Yan-Ru; Yang, Sheng-Bang; Wu, Yong-Ding; Zeng, Yan-Ru; Qin, Guo-Qiang; Liang, Yu-Xiang; Dai, Qi-Shan; Jiang, Fu-Neng; Wu, Shu-lin; Zeng, Guo-Hua; Zhong, Wei-De; Wu, Chin-Lee	CLINICAL CANCER RESEARCH	2015	21	21		76	12,67
Extracellular vesicles: potential applications in cancer diagnosis, prognosis, and epidemiology	Verma, Mukesh; Lam, Tram Kim; Hebert, Elizabeth; Divi, Rao L.	BMC CLINICAL PATHOLOGY	2015	15			75	12,5
Circulating tumor DNA and liquid biopsy: opportunities, challenges, and recent advances in detection technologies	Gorgannezhad, Lena; Umer, Muhammad; Islam, Md. Nazmul; Nam-Trung Nguyen; Shiddiky, Muhammad J. A.	LAB ON A CHIP	2018	18	8		74	24,67
Long Noncoding RNAs in Cancer and Therapeutic Potential	Renganathan, Arun; Felley-Bosco, Emanuela	LONG NON CODING RNA BIOLOGY	2017	1008			74	18,5
High expression of AFAP1-AS1 is associated with poor survival and short-term recurrence in pancreatic ductal adenocarcinoma	Ye, Yibiao; Chen, Jie; Zhou, Yu; Fu, Zhiqiang; Zhou, Quanbo; Wang, YingXue; Gao, Wenchao; Zheng, ShangYou; Zhao, Xiaohui; Chen, Tao; Chen, Rufu	JOURNAL OF TRANSLATIONAL MEDICINE	2015	13			74	12,33
Molecular Pathways: Novel Approaches for Improved Therapeutic Targeting of Hedgehog Signaling in Cancer Stem Cells	Justilien, Verline; Fields, Alan P.	CLINICAL CANCER RESEARCH	2015	21	3		74	12,33
Intrinsic BET inhibitor resistance in SPOP-mutated prostate cancer is mediated by BET protein stabilization and AKT-mTORC1 activation	Zhang, Pingzhao; Wang, Dejie; Zhao, Yu; Ren, Shancheng; Gao, Kun; Ye, Zhenqing; Wang, Shangqian; Pan, Chun-Wu; Zhu, Yasheng; Yan, Yuqian; Yang, Yinhui; Wu, Di; He, Yundong; Zhang, Jun; Lu, Daru; Liu, Xiuping; Yu, Long; Zhao, Shimin; Li, Yao; Lin, Dong; Wang, Yuzhuo; Wang, Liguo; Chen, Yu; Sun, Yinghao; Wang, Chenji; Huang, Haojie	NATURE MEDICINE	2017	23	9		72	18

Phosphoproteome Integration Reveals Patient-Specific Networks in Prostate Cancer	Drake, Justin M.; Paull, Evan O.; Graham, Nicholas A.; Lee, John K.; Smith, Bryan A.; Titz, Bjoern; Stoyanova, Tanya; Faltermeier, Claire M.; Uzunangelov, Vladislav; Carlin, Daniel E.; Fleming, Daniel Teo; Wong, Christopher K.; Newton, Yulia; Sudha, Sud; Vashisht, Ajay A.; Huang, Jiaoti; Wohlschlegel, James A.; Graeber, Thomas G.; Witte, Owen N.; Stuart, Joshua M.	CELL	2016	166	4	72	14,4
PSA and beyond: alternative prostate cancer biomarkers	Saini, Sharanjot	CELLULAR ONCOLOGY	2016	39	2	72	14,4
Metabolomic analysis of prostate cancer risk in a prospective cohort: The alpha-tocolpherol, beta-carotene cancer prevention (ATBC) study	Mondul, Alison M.; Moore, Steven C.; Weinstein, Stephanie J.; Karoly, Edward D.; Sampson, Joshua N.; Albanes, Demetrius	INTERNATIONAL JOURNAL OF CANCER	2015	137	9	72	12
Exosomes Released from Breast Cancer Carcinomas Stimulate Cell Movement	Harris, Dinari A.; Patel, Sajni H.; Gucek, Marjan; Hendrix, An; Westbroek, Wendy; Taraska, Justin W.	PLOS ONE	2015	10	3	72	12
The Interaction between Epigenetics, Nutrition and the Development of Cancer	Bishop, Karen S.; Ferguson, Lynnette R.	NUTRIENTS	2015	7	2	72	12
Inhibition of fatty acid desaturation is detrimental to cancer cell survival in metabolically compromised environments	Peck, Barrie; Schug, Zachary T.; Zhang, Qifeng; Dankworth, Beatrice; Jones, Dylan T.; Smethurst, Elizabeth; Patel, Rachana; Mason, Susan; Jiang, Ming; Saunders, Rebecca; Howell, Michael; Mitter, Richard; Spencer-Dene, Bradley; Stamp, Gordon; McGarry, Lynn; James, Daniel; Shanks, Emma; Aboagye, Eric O.; Critchlow, Susan E.; Leung, Hing Y.; Harris, Adrian L.; Wakelam, Michael J. O.; Gottlieb, Eyal; Schulze, Almut	CANCER & METABOLISM	2016	4		71	14,2

Genome-wide CRISPR screen identifies HNRNPL as a prostate cancer dependency regulating RNA splicing	Fei, Teng; Chen, Yiwen; Xiao, Tengfei; Li, Wei; Cato, Laura; Zhang, Peng; Cotter, Maura B.; Bowden, Michaela; Lis, Rosina T.; Zhao, Shuang G.; Wu, Qiu; Feng, Felix Y.; Loda, Massimo; He, Housheng Hansen; Liu, X. Shirley; Brown, Myles	PROCEEDINGS OF THE NATIONAL ACADEMY OF SCIENCES OF THE UNITED STATES OF AMERICA	2017	114	26	70	17,5
A Prospective Investigation of PTEN Loss and ERG Expression in Lethal Prostate Cancer	Ahearn, Thomas U.; Pettersson, Andreas; Ebot, Ericka M.; Gerke, Travis; Graff, Rebecca E.; Morais, Carlos L.; Hicks, Jessica L.; Wilson, Kathryn M.; Rider, Jennifer R.; Sesso, Howard D.; Fiorentino, Michelangelo; Flavin, Richard; Finn, Stephen; Giovannucci, Edward L.; Loda, Massimo; Stampfer, Meir J.; De Marzo, Angelo M.; Mucci, Lorelei A.; Lotan, Tamara L.	JNCI-JOURNAL OF THE NATIONAL CANCER INSTITUTE	2016	108	2	70	14
Novel Biomarker Signature That May Predict Aggressive Disease in African American Men With Prostate Cancer	Yamoah, Kosj; Johnson, Michael H.; Choeurng, Voleak; Faisal, Farzana A.; Yousefi, Kasra; Haddad, Zaid; Ross, Ashley E.; Alshalafa, Mohammed; Den, Robert; Lal, Priti; Feldman, Michael; Dicker, Adam P.; Klein, Eric A.; Davicioni, Elai; Rebbeck, Timothy R.; Schaeffer, Edward M.	JOURNAL OF CLINICAL ONCOLOGY	2015	33	25	70	11,67
Targeting the adaptive molecular landscape of castration-resistant prostate cancer	Wyatt, Alexander W.; Gleave, Martin E.	EMBO MOLECULAR MEDICINE	2015	7	7	70	11,67
Role of exosomal proteins in cancer diagnosis	Li, Weihua; Li, Chuanyun; Zhou, Tong; Liu, Xiuhong; Liu, Xiaoni; Li, Xiuhui; Chen, Dexi	MOLECULAR CANCER	2017	16		69	17,25
SRRM4 Drives Neuroendocrine Transdifferentiation of Prostate Adenocarcinoma Under Androgen Receptor Pathway Inhibition	Li, Yinan; Donmez, Nilgun; Sahinalp, Cenk; Xie, Ning; Wang, Yuwei; Xue, Hui; Mo, Fan; Beltran, Himisha; Gleave, Martin; Wang, Yuzhuo; Collins, Colin; Dong, Xuesen	EUROPEAN UROLOGY	2017	71	1	69	17,25

Development and Clinical Validation of an In Situ Biopsy-Based Multimarker Assay for Risk Stratification in Prostate Cancer	Blume-Jensen, Peter; Berman, David M.; Rimm, David L.; Shipitsin, Michail; Putzi, Mathew; Nifong, Thomas P.; Small, Clayton; Choudhury, Sibgat; Capela, Teresa; Coupal, Louis; Ernst, Christina; Hurley, Aeron; Kaprelyants, Alex; Chang, Hua; Giladi, Eldar; Nardone, Julie; Dunyak, James; Loda, Massimo; Klein, Eric A.; Magi-Galluzzi, Cristina; Latour, Mathieu; Epstein, Jonathan I.; Kantoff, Philip; Saad, Fred	CLINICAL CANCER RESEARCH	2015	21	11	69	11,5
The association between germline BRCA2 variants and sensitivity to platinum-based chemotherapy among men with metastatic prostate cancer	Pomerantz, Mark M.; Spisak, Sandor; Jia, Li; Cronin, Angel M.; Csabai, Istvan; Ledet, Elisa; Sartor, A. Oliver; Rainville, Irene; O'Connor, Edward P.; Herbert, Zachary T.; Szallasi, Zoltan; Oh, William K.; Kantoff, Philip W.; Garber, Judy E.; Schrag, Deborah; Kibel, Adam S.; Freedman, Matthew L.	CANCER	2017	123	18	68	17
Truncation and constitutive activation of the androgen receptor by diverse genomic rearrangements in prostate cancer	Henzler, Christine; Li, Yingming; Yang, Rendong; McBride, Terri; Ho, Yeung; Sprenger, Cynthia; Liu, Gang; Coleman, Ilsa; Lakely, Bryce; Li, Rui; Ma, Shihong; Landman, Sean R.; Kumar, Vipin; Hwang, Tae Hyun; Raj, Ganesh V.; Higano, Celestia S.; Morrissey, Colm; Nelson, Peter S.; Plymate, Stephen R.; Dehm, Scott M.	NATURE COMMUNICATIONS	2016	7		68	13,6
MicroRNAs as Biomarkers for Diagnosis, Prognosis and Theranostics in Prostate Cancer	Bertoli, Gloria; Cava, Claudia; Castiglioni, Isabella	INTERNATIONAL JOURNAL OF MOLECULAR SCIENCES	2016	17	3	68	13,6
Landscape of gene fusions in epithelial cancers: seq and ye shall find	Kumar-Sinha, Chandan; Kalyana-Sundaram, Shanker; Chinnaiyan, Arul M.	GENOME MEDICINE	2015	7		68	11,33
Therapeutic Implications for Overcoming Radiation Resistance in Cancer Therapy	Kim, Byeong Mo; Hong, Yunkyung; Lee, Seunghoon; Liu, Pengda; Lim, Ji Hong; Lee, Yong Heon; Lee, Tae Ho; Chang, Kyu Tae; Hong, Yonggeun	INTERNATIONAL JOURNAL OF MOLECULAR SCIENCES	2015	16	11	68	11,33

Assessing the Clinical Impact of Risk Prediction Models With Decision Curves: Guidance for Correct Interpretation and Appropriate Use	Kerr, Kathleen F.; Brown, Marshall D.; Zhu, Kehao; Janes, Holly	JOURNAL OF CLINICAL ONCOLOGY	2016	34	21	67	13,4
A novel multi-tissue RNA diagnostic of healthy ageing relates to cognitive health status	Sood, Sanjana; Gallagher, Iain J.; Lunnon, Katie; Rullman, Eric; Keohane, Aoife; Crossland, Hannah; Phillips, Bethan E.; Cederholm, Tommy; Jensen, Thomas; van Loon, Luc J. C.; Lannfelt, Lars; Kraus, William E.; Atherton, Philip J.; Howard, Robert; Gustafsson, Thomas; Hodges, Angela; Timmons, James A.	GENOME BIOLOGY	2015	16		67	11,17
Synthetic essentiality of chromatin remodelling factor CHD1 in PTEN-deficient cancer	Zhao, Di; Lu, Xin; Wang, Guocan; Lan, Zhengdao; Liao, Wenting; Li, Jun; Liang, Xin; Chen, Jasper Robin; Shah, Sagar; Shang, Xiaoying; Tang, Ming; Deng, Pingna; Dey, Prasenjit; Chakravarti, Deepavali; Chen, Peiwen; Spring, Denise J.; Navone, Nora M.; Troncoso, Patricia; Zhang, Jianhua; Wang, Y. Alan; DePinho, Ronald A.	NATURE	2017	542	7642	65	16,25
SOX9 drives WNT pathway activation in prostate cancer	Ma, Fen; Ye, Huihui; He, Housheng Hansen; Gerrin, Sean J.; Chen, Sen; Tanenbaum, Benjamin A.; Cai, Changmeng; Sowalsky, Adam G.; He, Lingfeng; Wang, Hongyun; Balk, Steven P.; Yuan, Xin	JOURNAL OF CLINICAL INVESTIGATION	2016	126	5	65	13
ROR-gamma drives androgen receptor expression and represents a therapeutic target in castration-resistant prostate cancer	Wang, Junjian; Zou, June X.; Xue, Xiaoqian; Cai, Demin; Zhang, Yan; Duan, Zhijian; Xiang, Qiuping; Yang, Joy C.; Louie, Maggie C.; Borowsky, Alexander D.; Gao, Allen C.; Evans, Christopher P.; Lam, Kit S.; Xu, Jianzhen; Kung, Hsing-Jien; Evans, Ronald M.; Xu, Yong; Chen, Hong-Wu	NATURE MEDICINE	2016	22	5	65	13

Stem cell and neurogenic gene-expression profiles link prostate basal cells to aggressive prostate cancer	Zhang, Dingxiao; Park, Daechan; Zhong, Yi; Lu, Yue; Rycaj, Kiera; Gong, Shuai; Chen, Xin; Liu, Xin; Chao, Hsueh-Ping; Whitney, Pamela; Calhoun-Davis, Tammy; Takata, Yoko; Shen, Jianjun; Iyer, Vishwanath R.; Tang, Dean G.	NATURE COMMUNICATION S	2016	7		65	13
MicroRNA expression signature of castration-resistant prostate cancer: the microRNA-221/222 cluster functions as a tumour suppressor and disease progression marker	Goto, Yusuke; Kojima, Satoko; Nishikawa, Rika; Kurozumi, Akira; Kato, Mayuko; Enokida, Hideki; Matsushita, Ryosuke; Yamazaki, Kazuto; Ishida, Yasuo; Nakagawa, Masayuki; Naya, Yukio; Ichikawa, Tomohiko; Seki, Naohiko	BRITISH JOURNAL OF CANCER	2015	113	7	65	10,83
SPOP mutation leads to genomic instability in prostate cancer	Boysen, Gunther; Barbieri, Christopher E.; Prandi, Davide; Blattner, Mirjam; Chae, Sung-Suk; Dahija, Arun; Nataraj, Srilakshmi; Huang, Dennis; Marotz, Clarisse; Xu, Limei; Huang, Julie; Lecca, Paola; Chhangawala, Sagar; Liu, Deli; Zhou, Pengbo; Sboner, Andrea; de Bono, Johann S.; Demichelis, Francesca; Houvras, Yariv; Rubin, Mark A.	ELIFE	2015	4		65	10,83
CRISPR screens identify genomic ribonucleotides as a source of PARP-trapping lesions	Zimmermann, Michal; Murina, Olga; Reijns, Martin A. M.; Agathanggelou, Angelo; Challis, Rachel; Tarnauskaite, Zygimante; Muir, Morwenna; Fluteau, Adeline; Aregger, Michael; McEwan, Andrea; Yuan, Wei; Clarke, Matthew; Lambros, Maryou B.; Paneesha, Shankara; Moss, Paul; Chandrashekhar, Megha; Angers, Stephane; Moffat, Jason; Brunton, Valerie G.; Hart, Traver; de Bono, Johann; Stankovic, Tatjana; Jackson, Andrew P.; Durocher, Daniel	NATURE	2018	559	7713	64	21,33
The role of long noncoding RNAs in cancer: the dark matter matters	Hu, Xiaowen; Sood, Anil K.; Dang, Chi V.; Zhang, Lin	CURRENT OPINION IN GENETICS & DEVELOPMENT	2018	48		64	21,33

The NOTCH1-MYC highway toward T-cell acute lymphoblastic leukemia	Sanchez-Martin, Marta; Ferrando, Adolfo	BLOOD	2017	129	9	64	16
Higher plasma levels of lysophosphatidylcholine 18:0 are related to a lower risk of common cancers in a prospective metabolomics study	Kuehn, Tilman; Floegel, Anna; Sookthai, Disorn; Johnson, Theron; Rolle-Kampczyk, Ulrike; Otto, Wolfgang; von Bergen, Martin; Boeing, Heiner; Kaaks, Rudolf	BMC MEDICINE	2016	14		64	12,8
Integrated Proteomic and Glycoproteomic Analyses of Prostate Cancer Cells Reveal Glycoprotein Alteration in Protein Abundance and Glycosylation	Shah, Punit; Wang, Xiangchun; Yang, Weiming; Eshghi, Shadi Toghi; Sun, Shisheng; Hoti, Naseruddin; Chen, Lijun; Yang, Shuang; Pasay, Jered; Rubin, Abby; Zhang, Hui	MOLECULAR & CELLULAR PROTEOMICS	2015	14	10	64	10,67

Sequencing of prostate cancers identifies new cancer genes, routes of progression and drug targets	Wedge, David C.; Gundem, Gunes; Mitchell, Thomas; Woodcock, Dan J.; Martincorena, Inigo; Ghori, Mohammed; Zamora, Jorge; Butler, Adam; Whitaker, Hayley; Kote-Jarai, Zsofia; Alexandrov, Ludmil B.; Van Loo, Peter; Massie, Charlie E.; Dentro, Stefan; Warren, Anne Y.; Verrill, Clare; Berney, Dan M.; Dennis, Nening; Merson, Sue; Hawkins, Steve; Howat, William; Lu, Yong-Jie; Lambert, Adam; Kay, Jonathan; Kremeyer, Barbara; Karaszi, Katalin; Luxton, Hayley; Camacho, Niedzica; Marsden, Luke; Edwards, Sandra; Matthews, Lucy; Bo, Valeria; Leongamornlert, Daniel; McLaren, Stuart; Ng, Anthony; Yu, Yongwei; Zhang, Hongwei; Dadaev, Tokhir; Thomas, Sarah; Easton, Douglas F.; Ahmed, Mahbubl; Bancroft, Elizabeth; Fisher, Cyril; Livni, Naomi; Nicol, David; Tavare, Simon; Gill, Pelvender; Greenman, Christopher; Khoo, Vincent; Van As, Nicholas; Kumar, Pardeep; Ogden, Christopher; Cahill, Declan; Thompson, Alan; Mayer, Erik; Rowe, Edward; Dudderidge, Tim; Gnanapragasam, Vincent; Shah, Nimish C.; Raine, Keiran; Jones, David; Menzies, Andrew; Stebbings, Lucy; Teague, Jon; Hazell, Steven; Corbishley, Cathy; de Bono, Johann; Attard, Gerhardt; Isaacs, William; Visakorpi, Tapio; Fraser, Michael; Boutros, Paul C.; Bristow, Robert G.; Workman, Paul; Sander, Chris; Hamdy,	NATURE GENETICS	2018	50	5	62	20,67
WNT signalling in prostate cancer	Murillo-Garzon, Virginia; Kypta, Robert	NATURE REVIEWS UROLOGY	2017	14	11	62	15,5

Accession of Tumor Heterogeneity by Multiplex Transcriptome Profiling of Single Circulating Tumor Cells	Gorges, Tobias M.; Kuske, Andra; Roeck, Katharina; Mauermann, Oliver; Mueller, Volkmar; Peine, Sven; Verpoort, Karl; Novosadova, Vendula; Kubista, Mikael; Riethdorf, Sabine; Pantel, Klaus	CLINICAL CHEMISTRY	2016	62	11	62	12,4
The role of epigenetics in genetic and environmental epidemiology	Ladd-Acosta, Christine; Fallin, M. Daniele	EPIGENOMICS	2016	8	2	62	12,4
The use of exome capture RNA-seq for highly degraded RNA with application to clinical cancer sequencing	Cieslik, Marcin; Chugh, Rashmi; Wu, Yi-Mi; Wu, Ming; Brennan, Christine; Lonigro, Robert; Su, Fengyun; Wang, Rui; Siddiqui, Javed; Mehra, Rohit; Cao, Xuhong; Lucas, David; Chinnaiyan, Arul M.; Robinson, Dan	GENOME RESEARCH	2015	25	9	62	10,33
MEXPRESS: visualizing expression, DNA methylation and clinical TCGA data	Koch, Alexander; De Meyer, Tim; Jeschke, Jana; Van Criekinge, Wim	BMC GENOMICS	2015	16		62	10,33
The elements of life and medicines	Chellan, Prinessa; Sadler, Peter J.	PHILOSOPHICAL TRANSACTIONS OF THE ROYAL SOCIETY A-MATHEMATICAL PHYSICAL AND ENGINEERING SCIENCES	2015	373	2037	62	10,33
Functional significance of aberrantly expressed microRNAs in prostate cancer	Goto, Yusuke; Kurozumi, Akira; Enokida, Hideki; Ichikawa, Tomohiko; Seki, Naohiko	INTERNATIONAL JOURNAL OF UROLOGY	2015	22	3	62	10,33
Serum-Based miRNAs in the Prediction and Detection of Recurrence in Melanoma Patients	Fleming, Nathaniel H.; Zhong, Judy; da Silva, Ines Pires; de Miera, Eleazar Vega-Saenz; Brady, Bobbi; Han, Sung Won; Hanniford, Doug; Wang, Jinhua; Shapiro, Richard L.; Hernando, Eva; Osman, Iman	CANCER	2015	121	1	62	10,33

Concordance of Circulating Tumor DNA and Matched Metastatic Tissue Biopsy in Prostate Cancer	Wyatt, Alexander W.; Annala, Matti; Aggarwal, Rahul; Beja, Kevin; Feng, Felix; Youngren, Jack; Foye, Adam; Lloyd, Paul; Nykter, Matti; Beer, Tomasz M.; Alumkal, Joshi J.; Thomas, George V.; Reiter, Robert E.; Rettig, Matthew B.; Evans, Christopher P.; Gao, Allen C.; Chi, Kim N.; Small, Eric J.; Gleave, Martin E.	JNCI-JOURNAL OF THE NATIONAL CANCER INSTITUTE	2018	110	1	61	20,33
Understanding the Effectiveness of Natural Compound Mixtures in Cancer through Their Molecular Mode of Action	Aung, Thazin Nwe; Qu, Zhipeng; Kortschak, R. Daniel; Adelson, David L.	INTERNATIONAL JOURNAL OF MOLECULAR SCIENCES	2017	18	3	61	15,25
Biology and evolution of poorly differentiated neuroendocrine tumors	Rickman, David S.; Beltran, Himisha; Demichelis, Francesca; Rubin, Mark A.	NATURE MEDICINE	2017	23	6	60	15
JAK-STAT signaling in cancer: From cytokines to non-coding genome	Pencik, Jan; Ha Thi Thanh Pham; Schmoellerl, Johannes; Javaheri, Tahereh; Schlederer, Michaela; Culig, Zoran; Merkel, Olaf; Moriggl, Richard; Grebien, Florian; Kenner, Lukas	CYTOKINE	2016	87		60	12
Regulation of prostate cancer progression by the tumor microenvironment	Shiao, Stephen L.; Chu, Gina Chia-Yi; Chung, Leland W. K.	CANCER LETTERS	2016	380	1	60	12
Analysis of DNA methylation in cancer: location revisited	Koch, Alexander; Joosten, Sophie C.; Feng, Zheng; de Ruijter, Tim C.; Draht, Muriel X.; Melotte, Veerle; Smits, Kim M.; Veeck, Jurgen; Herman, James G.; Van Neste, Leander; Van Criekinge, Wim; De Meyer, Tim; van Engeland, Manon	NATURE REVIEWS CLINICAL ONCOLOGY	2018	15	7	59	19,67
Targeting EZH2 in cancer therapy	Yamagishi, Makoto; Uchimaru, Kaoru	CURRENT OPINION IN ONCOLOGY	2017	29	5	59	14,75
Tumor evolution: Linear, branching, neutral or punctuated?	Davis, Alexander; Gao, Ruli; Navin, Nicholas	BIOCHIMICA ET BIOPHYSICA ACTA-REVIEWS ON CANCER	2017	1867	2	59	14,75

The Epithelial-to-Mesenchymal Transition-Like Process in Glioblastoma: An Updated Systematic Review and In Silico Investigation	Iser, Isabele C.; Pereira, Mariana B.; Lenz, Guido; Wink, Marcia R.	MEDICINAL RESEARCH REVIEWS	2017	37	2	59	14,75
Genomic, pathological, and clinical heterogeneity as drivers of personalized medicine in prostate cancer	Fraser, Michael; Berlin, Alejandro; Bristow, Robert G.; van der Kwast, Theodorus	UROLOGIC ONCOLOGY-SEMINARS AND ORIGINAL INVESTIGATIONS	2015	33	2	59	9,83
Genomic correlates of clinical outcome in advanced prostate cancer	Abida, Wassim; Cyrta, Joanna; Heller, Glenn; Prandi, Davide; Armenia, Joshua; Coleman, Ilsa; Cieslik, Marcin; Benelli, Matteo; Robinson, Dan; Van Allen, Eliezer M.; Sboner, Andrea; Fedrizzi, Tarcisio; Mosquera, Juan Miguel; Robinson, Brian D.; De Sarkar, Navonil; Kunju, Lakshmi P.; Tomlins, Scott; Wu, Yi Mi; Rodrigues, Daniel Nava; Loda, Massimo; Gopalan, Anuradha; Reuter, Victor E.; Pritchard, Colin C.; Mateo, Joaquin; Bianchini, Diletta; Miranda, Susana; Carreira, Suzanne; Rescigno, Pasquale; Filipenko, Julie; Vinson, Jacob; Montgomery, Robert B.; Beltran, Himisha; Heath, Elisabeth I.; Scher, Howard I.; Kantoff, Philip W.; Taplin, Mary-Ellen; Schultz, Nikolaus; deBono, Johann S.; Demichelis, Francesca; Nelson, Peter S.; Rubin, Mark A.; Chinnaiyan, Arul M.; Sawyers, Charles L.	PROCEEDINGS OF THE NATIONAL ACADEMY OF SCIENCES OF THE UNITED STATES OF AMERICA	2019	116	23	58	29

Widespread and Functional RNA Circularization in Localized Prostate Cancer	Chen, Sujun; Huang, Vincent; Xu, Xin; Livingstone, Julie; Soares, Fraser; Jeon, Jouhyun; Zeng, Yong; Hua, Junjie Tony; Petricca, Jessica; Guo, Haiyang; Wang, Miranda; Yousif, Fouad; Zhang, Yuzhe; Donmez, Nilgun; Ahmed, Musaddeque; Volik, Stas; Lapuk, Anna; Chua, Melvin L. K.; Heisler, Lawrence E.; Foucal, Adrien; Fox, Natalie S.; Fraser, Michael; Bhandari, Vinayak; Shiah, Yu-Jia; Guan, Jiansheng; Li, Jixi; Orain, Michele; Picard, Valerie; Hovington, Helene; Bergeron, Alain; Lacombe, Louis; Fradet, Yves; Tetu, Bernard; Liu, Stanley; Feng, Felix; Wu, Xue; Shao, Yang W.; Komor, Malgorzata A.; Sahinalp, Cenk; Collins, Colin; Hoogstrate, Youri; de Jong, Mark; Fijneman, Remond J. A.; Fei, Teng; Jenster, Guido; van der Kwast, Theodorus; Bristow, Robert G.; Boutros, Paul C.; He, Housheng Hansen	CELL	2019	176	4	58	29
Minimal functional driver gene heterogeneity among untreated metastases	Reiter, Johannes G.; Makohon-Moore, Alvin P.; Gerold, Jeffrey M.; Heyde, Alexander; Attiyeh, Marc A.; Kohutek, Zachary A.; Tokheim, Collin J.; Brown, Alexia; DeBlasio, Rayne M.; Niyazov, Juliana; Zucker, Amanda; Karchin, Rachel; Kinzler, Kenneth W.; Iacobuzio-Donahue, Christine A.; Vogelstein, Bert; Nowak, Martin A.	SCIENCE	2018	361	6406	58	19,33
A Pan-cancer Analysis of the Expression and Clinical Relevance of Small Nucleolar RNAs in Human Cancer	Gong, Jing; Li, Yajuan; Liu, Chun-Jie; Xiang, Yu; Li, Chunlai; Ye, Youqiong; Zhang, Zhao; Hawke, David H.; Park, Peter K.; Diao, Lixia; Putkey, John A.; Yang, Liuqing; Guo, An-Yuan; Lin, Chunru; Han, Leng	CELL REPORTS	2017	21	7	58	14,5
Prostate cancer, PI3K, PTEN and prognosis	Wise, Helen M.; Hermida, Miguel A.; Leslie, Nicholas R.	CLINICAL SCIENCE	2017	131	3	58	14,5

Molecular profiling of prostate cancer derived exosomes may reveal a predictive signature for response to docetaxel	Kharaziha, Pedram; Chioureas, Dimitris; Rutishauser, Dorothea; Baltatzis, George; Lennartsson, Lena; Fonseca, Pedro; Azimi, Alireza; Hultenby, Kjell; Zubarev, Roman; Ullen, Anders; Yachnin, Jeffrey; Nilsson, Sten; Panaretakis, Theocharis	ONCOTARGET	2015	6	25	58	9,67
Utilization of a Genomic Classifier for Prediction of Metastasis Following Salvage Radiation Therapy after Radical Prostatectomy	Freedland, Stephen J.; Choeurng, Voleak; Howard, Lauren; De Hoedt, Amanda; du Plessis, Marguerite; Yousefi, Kasra; Lam, Lucia L.; Buerki, Christine; Ra, Seong; Robbins, Bruce; Trabulsi, Edouard J.; Shah, Nikhil L.; Abdollah, Firas; Feng, Felix Y.; Davicioni, Elai; Dicker, Adam P.; Karnes, Robert J.; Den, Robert B.	EUROPEAN UROLOGY	2016	70	4	57	11,4
Genetic polymorphisms and paclitaxel- or docetaxel-induced toxicities: A systematic review	Frederiks, C. N.; Lam, S. W.; Guchelaar, H. J.; Boven, E.	CANCER TREATMENT REVIEWS	2015	41	10	57	9,5
Associations of Luminal and Basal Subtyping of Prostate Cancer With Prognosis and Response to Androgen Deprivation Therapy	Zhao, Shuang G.; Chang, S. Laura; Erho, Nicholas; Yu, Menggang; Lehrer, Jonathan; Alshalalfa, Mohammed; Speers, Corey; Cooperberg, Matthew R.; Kim, Won; Ryan, Charles J.; Den, Robert B.; Freedland, Stephen J.; Posadas, Edwin; Sandler, Howard; Klein, Eric A.; Black, Peter; Seiler, Roland; Tomlins, Scott A.; Chinnaiyan, Arul M.; Jenkins, Robert B.; Davicioni, Elai; Ross, Ashley E.; Schaeffer, Edward M.; Nguyen, Paul L.; Carroll, Peter R.; Karnes, R. Jeffrey; Spratt, Daniel E.; Feng, Felix Y.	JAMA ONCOLOGY	2017	3	12	56	14
Pan-cancer analysis of bi-allelic alterations in homologous recombination DNA repair genes	Riaz, Nadeem; Blecua, Pedro; Lim, Raymond S.; Shen, Ronglai; Higginson, Daniel S.; Weinhold, Nils; Norton, Larry; Weigelt, Britta; Powell, Simon N.; Reis-Filho, Jorge S.	NATURE COMMUNICATIONS	2017	8		56	14

Proteomic profiling of NCI-60 extracellular vesicles uncovers common protein cargo and cancer type-specific biomarkers	Hurwitz, Stephanie N.; Rider, Mark A.; Bundy, Joseph L.; Liu, Xia; Singh, Rakesh K.; Meckes, David G., Jr.	ONCOTARGET	2016	7	52	56	11,2
Dual-strand tumor-suppressor microRNA-145 (miR-145-5p and miR-145-3p) coordinately targeted MTDH in lung squamous cell carcinoma	Mataki, Hiroko; Seki, Naohiko; Mizuno, Keiko; Nohata, Nijiro; Kamikawaji, Kazuto; Kumamoto, Tomohiro; Koshizuka, Keiichi; Goto, Yusuke; Inoue, Hiromasa	ONCOTARGET	2016	7	44	56	11,2
LncRNAs as new biomarkers to differentiate triple negative breast cancer from non-triple negative breast cancer	Lv, Mingming; Xu, Pengfei; Wu, Ying; Huang, Lei; Li, Wenqu; Lv, Shanshan; Wu, Xiaowei; Zeng, Xin; Shen, Rong; Jia, Xuemei; Yin, Yongmei; Gu, Yun; Yuan, Hongyan; Xie, Hui; Fu, Ziyi	ONCOTARGET	2016	7	11	54	10,8
Prostate cancer in East Asia: evolving trend over the last decade	Zhu, Yao; Wang, Hong-Kai; Qu, Yuan-Yuan; Ye, Ding-Wei	ASIAN JOURNAL OF ANDROLOGY	2015	17	1	54	9
Development and Validation of a Novel Integrated Clinical-Genomic Risk Group Classification for Localized Prostate Cancer	Spratt, Daniel E.; Zhang, Jingbin; Santiago-Jimenez, Maria; Dess, Robert T.; Davis, John W.; Den, Robert B.; Dicker, Adam P.; Kane, Christopher J.; Pollack, Alan; Stoyanova, Radka; Abdollah, Firas; Ross, Ashley E.; Cole, Adam; Uchio, Edward; Randall, Josh M.; Hao Nguyen; Zhao, Shuang G.; Mehra, Rohit; Glass, Andrew G.; Lam, Lucia L. C.; Chelliserry, Jijumon; du Plessis, Marguerite; Choeurng, Voleak; Aranes, Maria; Kolisnik, Tyler; Margrave, Jennifer; Alter, Jason; Jordan, Jennifer; Buerki, Christine; Yousefi, Kasra; Haddad, Zaid; Davicioni, Elai; Trabulsi, Edouard J.; Loeb, Stacy; Tewari, Ashutosh; Carroll, Peter R.; Weinmann, Sheila; Schaeffer, Edward M.; Klein, Eric A.; Karnes, R. Jeffrey; Feng, Felix Y.; Nguyen, Paul L.	JOURNAL OF CLINICAL ONCOLOGY	2018	36	6	53	17,67

MetaLnc9 Facilitates Lung Cancer Metastasis via a PGK1-Activated AKT/mTOR Pathway	Yu, Tao; Zhao, Yingjun; Hu, Zhixiang; Li, Jing; Chu, Dandan; Zhang, Jiwei; Li, Zhe; Chen, Bing; Zhang, Xiao; Pan, Hongyu; Li, Shengli; Lin, Hechun; Liu, Lei; Yan, Mingxia; He, Xianghuo; Yao, Ming	CANCER RESEARCH	2017	77	21	53	13,25
Integrated Classification of Prostate Cancer Reveals a Novel Luminal Subtype with Poor Outcome	You, Sungyong; Knudsen, Beatrice S.; Erho, Nicholas; Alshalalfa, Mohammed; Takhar, Mandeep; Ashab, Hussam Al-deen; Davicioni, Elai; Karnes, R. Jeffrey; Klein, Eric A.; Den, Robert B.; Ross, Ashley E.; Schaeffer, Edward M.; Garraway, Isla P.; Kim, Jayoung; Freeman, Michael R.	CANCER RESEARCH	2016	76	17	53	10,6
Genomic Analysis of Tumor Microenvironment Immune Types across 14 Solid Cancer Types: Immunotherapeutic Implications	Chen, Yu-Pei; Zhang, Yu; Lv, Jia-Wei; Li, Ying-Qin; Wang, Ya-Qin; He, Qing-Mei; Yang, Xiao-Jing; Sun, Ying; Mao, Yan-Ping; Yun, Jing-Ping; Liu, Na; Ma, Jun	THERANOSTICS	2017	7	14	52	13
Urine metabolic fingerprinting using LC-MS and GC-MS reveals metabolite changes in prostate cancer: A pilot study	Struck-Lewicka, Wiktoria; Kordalewska, Marta; Bujak, Renata; Mpanga, Arlette Yumba; Markuszewski, Marcin; Jacyna, Julia; Matuszewski, Marcin; Kaliszan, Roman; Markuszewski, Michal J.	JOURNAL OF PHARMACEUTICAL AND BIOMEDICAL ANALYSIS	2015	111		52	8,67
Whole Transcriptome Sequencing Reveals Extensive Unspliced mRNA in Metastatic Castration-Resistant Prostate Cancer	Sowalsky, Adam G.; Xia, Zheng; Wang, Liguo; Zhao, Hao; Chen, Shaoyong; Bubley, Glenn J.; Balk, Steven P.; Li, Wei	MOLECULAR CANCER RESEARCH	2015	13	1	52	8,67
Cholesterol lowering: role in cancer prevention and treatment	Murai, Toshiyuki	BIOLOGICAL CHEMISTRY	2015	396	1	52	8,67
Genomic Markers in Prostate Cancer Decision Making	Cucchiara, Vito; Cooperberg, Matthew R.; Dall'Era, Marc; Lin, Daniel W.; Montorsi, Francesco; Schalken, Jack A.; Evans, Christopher P.	EUROPEAN UROLOGY	2018	73	4	51	17
Clinical applications of the CellSearch platform in cancer patients	Riethdorf, Sabine; O'Flaherty, Linda; Hille, Claudia; Pantel, Klaus	ADVANCED DRUG DELIVERY REVIEWS	2018	125		51	17

LuCaP Prostate Cancer Patient-Derived Xenografts Reflect the Molecular Heterogeneity of Advanced Disease and Serve as Models for Evaluating Cancer Therapeutics	Nguyen, Holly M.; Vessella, Robert L.; Morrissey, Colm; Brown, Lisha G.; Coleman, Ilsa M.; Higano, Celestia S.; Mostaghel, Elahe A.; Zhang, Xiaotun; True, Lawrence D.; Lam, Hung-Ming; Roudier, Martine; Lange, Paul H.; Nelson, Peter S.; Corey, Eva	PROSTATE	2017	77	6	51	12,75
Yin Yang 1 is associated with cancer stem cell transcription factors (SOX2, OCT4, BMI1) and clinical implication	Kaufhold, Samantha; Garban, Hermes; Bonavida, Benjamin	JOURNAL OF EXPERIMENTAL & CLINICAL CANCER RESEARCH	2016	35		51	10,2
Large extracellular vesicles carry most of the tumour DNA circulating in prostate cancer patient plasma	Vagner, Tatyana; Spinelli, Cristiana; Minciacchi, Valentina R.; Balaj, Leonora; Zandian, Mandana; Conley, Andrew; Zijlstra, Andries; Freeman, Michael R.; Demichelis, Francesca; De, Subhajyoti; Posadas, Edwin M.; Tanaka, Hisashi; Di Vizio, Dolores	JOURNAL OF EXTRACELLULAR VESICLES	2018	7	1	50	16,67
Opposing effects of cancer-type-specific SPOP mutants on BET protein degradation and sensitivity to BET inhibitors	Janouskova, Hana; El Tekle, Geniver; Bellini, Elisa; Udeshi, Namrata D.; Rinaldi, Anna; Ulbricht, Anna; Bernasocchi, Tiziano; Civenni, Gianluca; Losa, Marco; Svinkina, Tanya; Bielski, Craig M.; Kryukov, Gregory V.; Cascione, Luciano; Napoli, Sara; Enchev, Radoslav I.; Mutch, David G.; Carney, Michael E.; Berchuck, Andrew; Winterhoff, Boris J. N.; Broaddus, Russell R.; Schraml, Peter; Moch, Holger; Bertoni, Francesco; Catapano, Carlo V.; Peter, Matthias; Carr, Steven A.; Garraway, Levi A.; Wild, Peter J.; Theurillat, Jean-Philippe P.	NATURE MEDICINE	2017	23	9	50	12,5
Meta-analysis of miRNA expression profiles for prostate cancer recurrence following radical prostatectomy	Pashaei, Elnaz; Pashaei, Elham; Ahmady, Maryam; Ozen, Mustafa; Aydin, Nizamettin	PLOS ONE	2017	12	6	50	12,5
Roles of long noncoding RNAs in colorectal cancer metastasis	Li, He; Ma, Si-Qing; Huang, Jin; Chen, Xiao-Ping; Zhou, Hong-Hao	ONCOTARGET	2017	8	24	50	12,5

Patient derived organoids to model rare prostate cancer phenotypes	Puca, Loredana; Bareja, Rohan; Prandi, Davide; Shaw, Reid; Benelli, Matteo; Karthaus, Wouter R.; Hess, Judy; Sigouros, Michael; Donoghue, Adam; Kossai, Myriam; Gao, Dong; Cyrta, Joanna; Sailer, Verena; Vosoughi, Aram; Pauli, Chantal; Churakova, Yelena; Cheung, Cynthia; Deonarine, Lesa Dayal; McNary, Terra J.; Rosati, Rachele; Tagawa, Scott T.; Nanus, David M.; Mosquera, Juan Miguel; Sawyers, Charles L.; Chen, Yu; Inghirami, Giorgio; Rao, Rema A.; Grandori, Carla; Elemento, Olivier; Sboner, Andrea; Demichelis, Francesca; Rubin, Mark A.; Beitrah, Himisha	NATURE COMMUNICATION S	2018	9		49	16,33
Role of miRNAs in human cancer metastasis: Implications for therapeutic intervention	Jafri, Mohammad Alam; Al-Qahtani, Mohammed Hussein; Shay, Jerry William	SEMINARS IN CANCER BIOLOGY	2017	44		49	12,25
The Proteome of Primary Prostate Cancer	Iglesias-Gato, Diego; Wikstrom, Pernilla; Tyanova, Stefka; Lavallee, Charlotte; Thysell, Elin; Carlsson, Jessica; Hagglof, Christina; Cox, Juergen; Andren, Ove; Stattin, Par; Egevad, Lars; Widmark, Anders; Bjartell, Anders; Collins, Colin C.; Bergh, Anders; Geiger, Tamar; Mann, Matthias; Flores-Morales, Amilcar	EUROPEAN UROLOGY	2016	69	5	49	9,8
Integration of lipidomics and transcriptomics unravels aberrant lipid metabolism and defines cholesteryl oleate as potential biomarker of prostate cancer	Li, Jia; Ren, Shancheng; Piao, Hai-long; Wang, Fubo; Yin, Peiyuan; Xu, Chuanliang; Lu, Xin; Ye, Guozhu; Shao, Yaping; Yan, Min; Zhao, Xinjie; Sun, Yinghao; Xu, Guowang	SCIENTIFIC REPORTS	2016	6		49	9,8
EpCAM-Independent Enrichment of Circulating Tumor Cells in Metastatic Breast Cancer	Schneck, Helen; Gierke, Berthold; Uppenkamp, Frauke; Behrens, Bianca; Niederacher, Dieter; Stoecklein, Nikolas H.; Templin, Markus F.; Pawlak, Michael; Fehm, Tanja; Neubauer, Hans	PLOS ONE	2015	10	12	49	8,17

Urinary Volatile Organic Compounds for the Detection of Prostate Cancer	Khalid, Tanzeela; Aggio, Raphael; White, Paul; Costello, Ben De Lacy; Persad, Raj; Al-Kateb, Huda; Jones, Peter; Probert, Chris S.; Ratcliffe, Norman	PLOS ONE	2015	10	11	49	8,17
Mouse hospital and co-clinical trial project-from bench to bedside	Clohessy, John G.; Pandolfi, Pier Paolo	NATURE REVIEWS CLINICAL ONCOLOGY	2015	12	8	49	8,17
IGFBP7, a novel tumor stroma marker, with growth-promoting effects in colon cancer through a paracrine tumor-stroma interaction	Rupp, C.; Scherzer, M.; Rudisch, A.; Unger, C.; Haslinger, C.; Schweifer, N.; Artaker, M.; Nivarthi, H.; Moriggl, R.; Hengstschlaeger, M.; Kerjaschki, D.; Sommergruber, W.; Dolznig, H.; Garin-Chesa, P.	ONCOGENE	2015	34	7	49	8,17
Identification of novel long non-coding RNAs in clear cell renal cell carcinoma	Blondeau, Jasmine J. C.; Deng, Mario; Syring, Isabella; Schroedter, Sarah; Schmidt, Doris; Perner, Sven; Mueller, Stefan C.; Ellinger, Joerg	CLINICAL EPIGENETICS	2015	7		49	8,17
Insights into Chemoresistance of Prostate Cancer	Zhang, Wei; Meng, Yan; Liu, Na; Wen, Xiao-Fei; Yang, Tao	INTERNATIONAL JOURNAL OF BIOLOGICAL SCIENCES	2015	11	10	49	8,17
The crucial role of multiomic approach in cancer research and clinically relevant outcomes	Lu, Miaolong; Zhan, Xianquan	EPMA JOURNAL	2018	9	1	48	16
Comprehensive Profiling of the Androgen Receptor in Liquid Biopsies from Castration-resistant Prostate Cancer Reveals Novel Intra-AR Structural Variation and Splice Variant Expression Patterns	De laere, Bram; van Dam, Pieter-Jan; Whitington, Tom; Mayrhofer, Markus; Diaz, Emanuela Henao; Van den Eynden, Gert; Vandebroek, Jean; Del-Favero, Jurgen; Van Laere, Steven; Dirix, Luc; Gronberg, Henrik; Lindberg, Johan	EUROPEAN UROLOGY	2017	72	2	48	12
Identification of androgen-responsive lncRNAs as diagnostic and prognostic markers for prostate cancer	Wan, Xuechao; Huang, Wenhua; Yang, Shu; Zhang, Yalong; Pu, Honglei; Fu, Fangqiu; Huang, Yan; Wu, Hai; Li, Tao; Li, Yao	ONCOTARGET	2016	7	37	48	9,6
Biomarker Discovery in Human Prostate Cancer: an Update in Metabolomics Studies	Lima, Ana Rita; Bastos, Maria de Lourdes; Carvalho, Marcia; de Pinho, Paula Guedes	TRANSLATIONAL ONCOLOGY	2016	9	4	48	9,6

Targeting the fibroblast growth factor receptor family in cancer	Hallinan, Niamh; Finn, Stephen; Cuffe, Sinead; Rafee, Shereen; O'Byrne, Kenneth; Gately, Kathy	CANCER TREATMENT REVIEWS	2016	46		48	9,6
Prostate cancer stem cells: the role of androgen and estrogen receptors	Di Zazzo, Erika; Galasso, Giovanni; Giovannelli, Pia; Di Donato, Marzia; Di Santi, Annalisa; Cernera, Gustavo; Rossi, Valentina; Abbondanza, Ciro; Moncharmont, Bruno; Sinisi, Antonio Agostino; Castoria, Gabriella; Migliaccio, Antimo	ONCOTARGET	2016	7	1	48	9,6
Supervised Multi-View Canonical Correlation Analysis (sMVCCA): Integrating Histologic and Proteomic Features for Predicting Recurrent Prostate Cancer	Lee, George; Singanamalli, Asha; Wang, Haibo; Feldman, Michael D.; Master, Stephen R.; Shih, Natalie N. C.; Spangler, Elaine; Rebbeck, Timothy; Tomaszewski, John E.; Madabhushi, Anant	IEEE TRANSACTIONS ON MEDICAL IMAGING	2015	34	1	48	8
Recent Advances in Prostate Cancer Treatment and Drug Discovery	Nevedomskaya, Ekaterina; Baumgart, Simon J.; Haendler, Bernard	INTERNATIONAL JOURNAL OF MOLECULAR SCIENCES	2018	19	5	47	15,67
Personalized Proteome Profiles of Healthy and Tumor Human Colon Organoids Reveal Both Individual Diversity and Basic Features of Colorectal Cancer	Cristobal, Alba; van den Toom, Henk W. P.; van de Wetering, Marc; Clevers, Hans; Heck, Albert J. R.; Mohammed, Shabaz	CELL REPORTS	2017	18	1	47	11,75
Inhibition of the hedgehog pathway in patients with basal-cell nevus syndrome: final results from the multicentre, randomised, double-blind, placebo-controlled, phase 2 trial	Tang, Jean Y.; Ally, Mina S.; Chanana, Anita M.; Mackay-Wiggan, Julian M.; Aszterbaum, Michelle; Lindgren, Joselyn A.; Ulerio, Grace; Rezaee, Melika R.; Gildengorin, Ginny; Marji, Jackleen; Clark, Charlotte; Bickers, David R.; Epstein, Ervin H., Jr.	LANCET ONCOLOGY	2016	17	12	47	9,4

FOXA1 overexpression mediates endocrine resistance by altering the ER transcriptome and IL-8 expression in ER-positive breast cancer	Fu, Xiaoyong; Jeselsohn, Rinath; Pereira, Resel; Hollingsworth, Emporia F.; Creighton, Chad J.; Li, Fugen; Shea, Martin; Nardone, Agostina; De Angelis, Carmine; Heiser, Laura M.; Anur, Pavana; Wang, Nicholas; Grasso, Catherine S.; Spellman, Paul T.; Griffith, Obi L.; Tsimelzon, Anna; Gutierrez, Carolina; Huang, Shixia; Edwards, Dean P.; Trivedi, Meghana V.; Rimawi, Mothaffar F.; Lopez-Terrada, Dolores; Hilsenbeck, Susan G.; Gray, Joe W.; Brown, Myles; Osborne, C. Kent; Schiff, Rachel	PROCEEDINGS OF THE NATIONAL ACADEMY OF SCIENCES OF THE UNITED STATES OF AMERICA	2016	113	43	47	9,4
A genetic basis for the variation in the vulnerability of cancer to DNA damage	Yard, Brian D.; Adams, Drew J.; Chie, Eui Kyu; Tamayo, Pablo; Battaglia, Jessica S.; Gopal, Priyanka; Rogacki, Kevin; Pearson, Bradley E.; Phillips, James; Raymond, Daniel P.; Pennell, Nathan A.; Almeida, Francisco; Cheah, Jaime H.; Clemons, Paul A.; Shamji, Alykhan; Peacock, Craig D.; Schreiber, Stuart L.; Hammerman, Peter S.; Abazeed, Mohamed E.	NATURE COMMUNICATIONS	2016	7		47	9,4
Altered Glycosylation in Prostate Cancer	Drake, Richard R.; Jones, E. Ellen; Powers, Thomas W.; Nyalwidhe, Julius O.	GLYCOSYLATION AND CANCER	2015	126		47	7,83

Analysis of the androgen receptor-regulated lncRNA landscape identifies a role for ARLNC1 in prostate cancer progression	Zhang, Yajia; Pitchiaya, Sethuramasundaram; Cieslik, Marcin; Niknafs, Yashar S.; Tien, Jean C. -Y.; Hosono, Yasuyuki; Iyer, Matthew K.; Yazdani, Sahr; Subramaniam, Shruthi; Shukla, Sudhanshu K.; Jiang, Xia; Wang, Lisha; Liu, Tzu-Ying; Uhl, Michael; Gawronski, Alexander R.; Qiao, Yuanyuan; Xiao, Lanbo; Dhanasekaran, Saravana M.; Juckette, Kristin M.; Kunju, Lakshmi P.; Cao, Xuhong; Patel, Utsav; Batish, Mona; Shukla, Girish C.; Paulsen, Michelle T.; Ljungman, Mats; Jiang, Hui; Mehra, Rohit; Backofen, Rolf; Sahinalp, Cenk S.; Freier, Susan M.; Watt, Andrew T.; Guo, Shuling; Wei, John T.; Feng, Felix Y.; Malik, Rohit; Chinnaiyan, Arul M.	NATURE GENETICS	2018	50	6	46	15,33
Metabolomic Biomarkers of Prostate Cancer: Prediction, Diagnosis, Progression, Prognosis, and Recurrence	Kelly, Rachel S.; Heiden, Matthew G. Vander; Giovannucci, Edward; Mucci, Lorelei A.	CANCER EPIDEMIOLOGY BIOMARKERS & PREVENTION	2016	25	6	46	9,2
Urinary Exosomes: The Potential for Biomarker Utility, Intercellular Signaling and Therapeutics in Urological Malignancy	Franzen, Carrie A.; Blackwell, Robert H.; Foreman, Kimberly E.; Kuo, Paul C.; Flanigan, Robert C.; Gupta, Gopal N.	JOURNAL OF UROLOGY	2016	195	5	46	9,2
Gene regulatory mechanisms underpinning prostate cancer susceptibility	Whitington, Thomas; Gao, Ping; Song, Wei; Ross-Adams, Helen; Lamb, Alastair D.; Yang, Yuehong; Svezia, Ilaria; Klevebring, Daniel; Mills, Ian G.; Karlsson, Robert; Halim, Silvia; Dunning, Mark J.; Egevad, Lars; Warren, Anne Y.; Neal, David E.; Gronberg, Henrik; Lindberg, Johan; Wei, Gong-Hong; Wiklund, Fredrik	NATURE GENETICS	2016	48	4	46	9,2

Techniques of using circulating tumor DNA as a liquid biopsy component in cancer management	Elazezy, Maha; Joosse, Simon A.	COMPUTATIONAL AND STRUCTURAL BIOTECHNOLOGY JOURNAL	2018	16		45	15
Impact of novel miR-145-3p regulatory networks on survival in patients with castration-resistant prostate cancer	Goto, Yusuke; Kurozumi, Akira; Arai, Takayuki; Nohata, Nijiro; Kojima, Satoko; Okato, Atsushi; Kato, Mayuko; Yamazaki, Kazuto; Ishida, Yasuo; Naya, Yukio; Ichikawa, Tomohiko; Seki, Naohiko	BRITISH JOURNAL OF CANCER	2017	117	3	45	11,25
Cooperative Dynamics of AR and ER Activity in Breast Cancer	D'Amato, Nicholas C.; Gordon, Michael A.; Babbs, Beatrice; Spoelstra, Nicole S.; Butterfield, Kiel T. Carson; Torkko, Kathleen C.; Phan, Vernon T.; Barton, Valerie N.; Rogers, Thomas J.; Sartorius, Carol A.; Elias, Anthony; Gertz, Jason; Jacobsen, Britta M.; Richer, Jennifer K.	MOLECULAR CANCER RESEARCH	2016	14	11	45	9
Targeting bet bromodomain proteins in solid tumors	Sahai, Vaibhav; Redig, Amanda J.; Collier, Katharine A.; Eckerdt, Frank D.; Munshi, Hidayatullah G.	ONCOTARGET	2016	7	33	45	9
Genomic and Epigenomic Alterations in Cancer	Chakravarthi, Balabhadrapatruni V. S. K.; Nepal, Saroj; Varambally, Sooryanarayana	AMERICAN JOURNAL OF PATHOLOGY	2016	186	7	45	9
Integrin beta 4 and vinculin contained in exosomes are potential markers for progression of prostate cancer associated with taxane-resistance	Kawakami, Kyojiro; Fujita, Yasunori; Kato, Taku; Mizutani, Kosuke; Kameyama, Koji; Tsumoto, Hiroki; Miura, Yuri; Deguchi, Takashi; Ito, Masafumi	INTERNATIONAL JOURNAL OF ONCOLOGY	2015	47	1	45	7,5
Selenium and Chronic Diseases: A Nutritional Genomics Perspective	Meplan, Catherine	NUTRIENTS	2015	7	5	45	7,5

Tumour heterogeneity poses a significant challenge to cancer biomarker research	Cyll, Karolina; Ersvaer, Elin; Vlatkovic, Ljiljana; Pradhan, Manohar; Kildal, Wanja; Kjaer, Marte Avranden; Kleppe, Andreas; Hveem, Tarjei S.; Carlsen, Birgitte; Gill, Silje; Loffeler, Sven; Haug, Erik Skaaheim; Waehre, Hakon; Sooriakumaran, Prasanna; Danielsen, Havard E.	BRITISH JOURNAL OF CANCER	2017	117	3	44	11
DNMT1 Inhibition Reprograms Pancreatic Cancer Stem Cells via Upregulation of the miR-17-92 Cluster	Zagorac, Sladjana; Alcala, Sonia; Fernandez Bayon, Gustavo; Kheir, Tony Bou; Schoenhals, Matthieu; Gonzalez-Neira, Anna; Fernandez Fraga, Mario; Aicher, Alexandra; Heeschen, Christopher; Sainz, Bruno, Jr.	CANCER RESEARCH	2016	76	15	44	8,8
Treatment of the Primary Tumor in Metastatic Prostate Cancer: Current Concepts and Future Perspectives	Bayne, Christopher E.; Williams, Stephen B.; Cooperberg, Matthew R.; Gleave, Martin E.; Graefen, Markus; Montorsi, Francesco; Novara, Giacomo; Smaldone, Marc C.; Sooriakumaran, Prasanna; Wiklund, Peter N.; Chapin, Brian F.	EUROPEAN UROLOGY	2016	69	5	44	8,8
DNA damage response and prostate cancer: defects, regulation and therapeutic implications	Karanika, S.; Karantanos, T.; Li, L.; Corn, P. G.; Thompson, T. C.	ONCOGENE	2015	34	22	44	7,33
Polycomb-mediated silencing in neuroendocrine prostate cancer	Clermont, Pier-Luc; Lin, Dong; Crea, Francesco; Wu, Rebecca; Xue, Hui; Wang, Yuwei; Thu, Kelsie L.; Lam, Wan L.; Collins, Colin C.; Wang, Yuzhuo; Helgason, Cheryl D.	CLINICAL EPIGENETICS	2015	7		44	7,33

Second-Generation HSP90 Inhibitor Onalespib Blocks mRNA Splicing of Androgen Receptor Variant 7 in Prostate Cancer Cells	Ferraldeschi, Roberta; Welti, Jonathan; Powers, Marissa V.; Yuan, Wei; Smyth, Tomoko; Seed, George; Riisnaes, Ruth; Hedayat, Somaieh; Wang, Hannah; Crespo, Mateus; Rodrigues, Daniel Nava; Figueiredo, Ines; Miranda, Susana; Carreira, Suzanne; Lyons, John F.; Sharp, Swee; Plymate, Stephen R.; Attard, Gerhardt; Wallis, Nicola; Workman, Paul; de Bono, Johann S.	CANCER RESEARCH	2016	76	9	43	8,6
Integrated gene and miRNA expression analysis of prostate cancer associated fibroblasts supports a prominent role for interleukin-6 in fibroblast activation	Doldi, Valentina; Callari, Maurizio; Giannoni, Elisa; D'Aiuto, Francesca; Maffezzini, Massimo; Valdagni, Riccardo; Chiarugi, Paola; Gandellini, Paolo; Zaffaroni, Nadia	ONCOTARGET	2015	6	31	43	7,17
Cooperative integration between HEDGEHOG-GLI signalling and other oncogenic pathways: implications for cancer therapy	Pandolfi, Silvia; Stecca, Barbara	EXPERT REVIEWS IN MOLECULAR MEDICINE	2015	17		43	7,17
Diverse genetic-driven immune landscapes dictate tumor progression through distinct mechanisms	Bezzi, Marco; Seitzer, Nina; Ishikawa, Tomoki; Reschke, Markus; Chen, Ming; Wang, Guocan; Mitchell, Caitlin; Ng, Christopher; Katon, Jesse; Lunardi, Andrea; Signoretti, Sabina; Clohessy, John G.; Zhang, Jiangwen; Pandolfi, Pier Paolo	NATURE MEDICINE	2018	24	2	42	14
Using circulating cell-free DNA to monitor personalized cancer therapy	Oellerich, Michael; Schuetz, Ekkehard; Beck, Julia; Kanzow, Philipp; Plowman, Piers N.; Weiss, Glen J.; Walson, Philip D.	CRITICAL REVIEWS IN CLINICAL LABORATORY SCIENCES	2017	54	3	42	10,5
Perspectives of long non-coding RNAs in cancer	Rao, Arunagiri Kuha Deva Magendhra; Rajkumar, Thangarajan; Mani, Samson	MOLECULAR BIOLOGY REPORTS	2017	44	2	42	10,5

Metabolomic Profiling of Extracellular Vesicles and Alternative Normalization Methods Reveal Enriched Metabolites and Strategies to Study Prostate Cancer-Related Changes	Puhka, Maija; Takatalo, Maarit; Nordberg, Maria-Elisa; Valkonen, Sami; Nandania, Jatin; Aatonen, Maria; Yliperttula, Marjo; Laitinen, Saara; Velagapudi, Vidya; Mirtti, Tuomas; Kallioniemi, Olli; Rannikko, Antti; Siljander, Pia R-M; Af Hallstrom, Taija Maria	THERANOSTICS	2017	7	16	42	10,5
Analytic validation of a clinical-grade PTEN immunohistochemistry assay in prostate cancer by comparison with PTEN FISH	Lotan, Tamara L.; Wei, Wei; Ludkovski, Olga; Morais, Carlos L.; Guedes, Liana B.; Jamaspishvili, Tamara; Lopez, Karen; Hawley, Sarah T.; Feng, Ziding; Fazli, Ladan; Hurtado-Coll, Antonio; McKenney, Jesse K.; Simko, Jeffrey; Carroll, Peter R.; Gleave, Martin; Lin, Daniel W.; Nelson, Peter S.; Thompson, Ian M.; True, Lawrence D.; Brooks, James D.; Lance, Raymond; Troyer, Dean; Squire, Jeremy A.	MODERN PATHOLOGY	2016	29	8	42	8,4
Advances in Therapeutic Cancer Vaccines	Wong, Karrie K.; Li, WeiWei Aileen; Mooney, David J.; Dranoff, Glenn	TUMOR IMMUNOLOGY	2016	130		42	8,4
Biodegradable nano-films for capture and non-invasive release of circulating tumor cells	Li, Wei; Reategui, Eduardo; Park, Myoung-Hwan; Castleberry, Steven; Deng, Jason Z.; Hsu, Bryan; Mayner, Sarah; Jensen, Anne E.; Sequist, Lecia V.; Maheswaran, Shyamala; Haber, Daniel A.; Toner, Mehmet; Stott, Shannon L.; Hammond, Paula T.	BIOMATERIALS	2015	65		42	7
Regulation of the glucocorticoid receptor via a BET-dependent enhancer drives antiandrogen resistance in prostate cancer	Shah, Neel; Wang, Ping; Wongvipat, John; Karthaus, Wouter R.; Abida, Wassim; Armenia, Joshua; Rockowitz, Shira; Drier, Yotam; Bernstein, Bradley E.; Long, Henry W.; Freedman, Matthew L.; Arora, Vivek K.; Zheng, Deyou; Sawyers, Charles L.	ELIFE	2017	6		41	10,25
miRNAs as novel biomarkers in the management of prostate cancer	Filella, Xavier; Foj, Laura	CLINICAL CHEMISTRY AND LABORATORY MEDICINE	2017	55	5	41	10,25

miR-375 induces docetaxel resistance in prostate cancer by targeting SEC23A and YAP1	Wang, Yuan; Lieberman, Rachel; Pan, Jing; Zhang, Qi; Du, Meijun; Zhang, Peng; Nevalainen, Marja; Kohli, Manish; Shenoy, Niraj K.; Meng, Hui; You, Ming; Wang, Liang	MOLECULAR CANCER	2016	15		41	8,2
Resistance to docetaxel in prostate cancer is associated with androgen receptor activation and loss of KDM5D expression	Komura, Kazumasa; Jeong, Seong Ho; Hinohara, Kunihiko; Qu, Fangfang; Wang, Xiaodong; Hiraki, Masayuki; Azuma, Haruhito; Lee, Gwo-Shu Mary; Kantoff, Philip W.; Sweeney, Christopher J.	PROCEEDINGS OF THE NATIONAL ACADEMY OF SCIENCES OF THE UNITED STATES OF AMERICA	2016	113	22	41	8,2
Challenges for CTC-based liquid biopsies: low CTC frequency and diagnostic leukapheresis as a potential solution	Stoecklein, Nikolas H.; Fischer, Johannes C.; Niederacher, Dieter; Terstappen, Leon W. M. M.	EXPERT REVIEW OF MOLECULAR DIAGNOSTICS	2016	16	2	41	8,2
A Somatically Acquired Enhancer of the Androgen Receptor Is a Noncoding Driver in Advanced Prostate Cancer	Takeda, David Y.; Spisak, Sandor; Seo, Ji-Heui; Bell, Connor; O'Connor, Edward; Korthauer, Keegan; Ribli, Dezso; Csabai, Istvan; Solymosi, Norbert; Szallasi, Zoltan; Stillman, David R.; Cejas, Paloma; Qiu, Xintao; Long, Henry W.; Tisza, Viktoria; Nuzzo, Pier Vitale; Rohanizadegan, Mersedeh; Pomerantz, Mark M.; Hahn, William C.; Freedman, Matthew L.	CELL	2018	174	2	40	13,33
The Metabolic Phenotype of Prostate Cancer	Eidelman, Eric; Twum-Ampofo, Jeffrey; Ansari, Jamal; Siddiqui, Mohummad Minhaj	FRONTIERS IN ONCOLOGY	2017	7		40	10
Subgroups of Castration-resistant Prostate Cancer Bone Metastases Defined Through an Inverse Relationship Between Androgen Receptor Activity and Immune Response	Ylitalo, Erik Bovinder; Thysell, Elin; Jernberg, Emma; Lundholm, Marie; Crnalic, Sead; Egevad, Lars; Stattin, Par; Widmark, Anders; Bergh, Anders; Wikstrom, Pernilla	EUROPEAN UROLOGY	2017	71	5	40	10

Integrative analyses of transcriptome sequencing identify novel functional lncRNAs in esophageal squamous cell carcinoma	Li, C-Q; Huang, G-W; Wu, Z-Y; Xu, Y-J; Li, X-C; Xue, Y-J; Zhu, Y.; Zhao, J-M; Li, M.; Zhang, J.; Wu, J-Y; Lei, F.; Wang, Q-Y; Li, S.; Zheng, C-P; Ai, B.; Tang, Z-D; Feng, C-C; Liao, L-D; Wang, S-H; Shen, J-H; Liu, Y-J; Bai, X-F; He, J-Z; Cao, H-H; Wu, B-L; Wang, M-R; Lin, D-C; Koeffler, H. P.; Wang, L-D; Li, X.; Li, E-M; Xu, L-Y	ONCOGENESIS	2017	6		40	10
Lysine acetylation and cancer: A proteomics perspective	Gil, Jeovanis; Ramirez-Torres, Alberto; Encarnacion-Guevara, Sergio	JOURNAL OF PROTEOMICS	2017	150		40	10
Monitoring cancer prognosis, diagnosis and treatment efficacy using metabolomics and lipidomics	Armitage, Emily G.; Southam, Andrew D.	METABOLOMICS	2016	12	9	40	8
Expression Profile Analysis of microRNAs in Prostate Cancer by Next-Generation Sequencing	Song, Chunjiao; Chen, Huan; Wang, Tingzhang; Zhang, Weiguang; Ru, Guomei; Lang, Juan	PROSTATE	2015	75	5	40	6,67
Genomic Classifier Augments the Role of Pathological Features in Identifying Optimal Candidates for Adjuvant Radiation Therapy in Patients With Prostate Cancer: Development and Internal Validation of a Multivariable Prognostic Model	Dalela, Deepansh; Santiago-Jimenez, Maria; Yousefi, Kasra; Karnes, R. Jeffrey; Ross, Ashley E.; Den, Robert B.; Freedland, Stephen J.; Schaeffer, Edward M.; Dicker, Adam P.; Menon, Mani; Briganti, Alberto; Davicioni, Elai; Abdollah, Firas	JOURNAL OF CLINICAL ONCOLOGY	2017	35	18	39	9,75
Proteomic analysis of urinary extracellular vesicles from high Gleason score prostate cancer	Fujita, Kazutoshi; Kume, Hideaki; Matsuzaki, Kyosuke; Kawashima, Atsunari; Ujike, Takeshi; Nagahara, Akira; Uemura, Motohide; Miyagawa, Yasushi; Tomonaga, Takeshi; Nonomura, Norio	SCIENTIFIC REPORTS	2017	7		39	9,75
The biology of DHX9 and its potential as a therapeutic target	Lee, Teresa; Pelletier, Jerry	ONCOTARGET	2016	7	27	39	7,8
Novel diagnostic and prognostic classifiers for prostate cancer identified by genome-wide microRNA profiling	Kristensen, Helle; Thomsen, Anni R.; Haldrup, Christa; Dyrskjot, Lars; Hoyer, Soren; Borre, Michael; Mouritzen, Peter; Orntoft, Torben F.; Sorensen, Karina Dalsgaard	ONCOTARGET	2016	7	21	39	7,8

Exosomes confer pro-survival signals to alter the phenotype of prostate cells in their surrounding environment	Hosseini-Beheshti, Elham; Choi, Wendy; Weiswald, Louis-Bastien; Kharmate, Geetanjali; Ghaffari, Mazyar; Roshan-Moniri, Mani; Hassona, Mohamed D.; Chan, Leslie; Chin, Mei Yieng; Tai, Isabella T.; Rennie, Paul S.; Fazli, Ladan; Guns, Emma S. Tomlinson	ONCOTARGET	2016	7	12	39	7,8
JARID1D Is a Suppressor and Prognostic Marker of Prostate Cancer Invasion and Metastasis	Li, Na; Dhar, Shilpa S.; Chen, Tsai-Yu; Kan, Pu-Yeh; Wei, Yongkun; Kim, Jae-Hwan; Chan, Chia-Hsin; Lin, Hui-Kuan; Hung, Mien-Chie; Lee, Min Gyu	CANCER RESEARCH	2016	76	4	39	7,8
Genome-wide analysis of microRNA and mRNA expression signatures in cancer	Li, Ming-hui; Fu, Sheng-bo; Xiao, Hua-sheng	ACTA PHARMACOLOGICA SINICA	2015	36	10	39	6,5
Biomarkers in prostate cancer - Current clinical utility and future perspectives	Kretschmer, Alexander; Tilki, Derya	CRITICAL REVIEWS IN ONCOLOGY HEMATOLOGY	2017	120		38	9,5
Targeting genomic rearrangements in tumor cells through Cas9-mediated insertion of a suicide gene	Chen, Zhang-Hui; Yu, Yan P.; Zuo, Ze-Hua; Nelson, Joel B.; Michalopoulos, George K.; Monga, Satdatshan; Liu, Silvia; Tseng, George; Luo, Jian-Hua	NATURE BIOTECHNOLOGY	2017	35	6	38	9,5
Metformin alters DNA methylation genome-wide via the H19/SAHH axis	Zhong, T.; Men, Y.; Lu, L.; Geng, T.; Zhou, J.; Mitsuhashi, A.; Shozu, M.; Maihle, N. J.; Carmichael, G. G.; Taylor, H. S.; Huang, Y.	ONCOGENE	2017	36	17	38	9,5
Prostate cancer biomarkers: Are we hitting the mark?	McGrath, Shannon; Christidis, Daniel; Perera, Marlon; Hong, Sung Kyu; Manning, Todd; Vela, Ian; Lawrentschuk, Nathan	PROSTATE INTERNATIONAL	2016	4	4	38	7,6
A long noncoding RNA signature for ulcerative colitis identifies IFNG-AS1 as an enhancer of inflammation	Padua, David; Mahurkar-Joshi, Swapna; Law, Ivy Ka Man; Polytarchou, Christos; Vu, John P.; Pisegna, Joseph R.; Shih, David; Iliopoulos, Dimitrios; Pothoulakis, Charalabos	AMERICAN JOURNAL OF PHYSIOLOGY-GASTROINTESTINAL AND LIVER PHYSIOLOGY	2016	311	3	38	7,6

Association of multiparametric MRI quantitative imaging features with prostate cancer gene expression in MRI-targeted prostate biopsies	Stoyanova, Radka; Pollack, Alan; Takhar, Mandeep; Lynne, Charles; Parra, Nestor; Lam, Lucia L. C.; Alshalalfa, Mohammed; Buerki, Christine; Castillo, Rosa; Jorda, Merce; Ashab, Hussam Al-deen; Kryvenko, Oleksandr N.; Punnen, Sanoj; Parekh, Dipen J.; Abramowitz, Matthew C.; Gillies, Robert J.; Davicioni, Elai; Erho, Nicholas; Ishkanian, Adrian	ONCOTARGET	2016	7	33	38	7,6
Prostate cancer radiomics and the promise of radiogenomics	Stoyanova, Radka; Takhar, Mandeep; Tschudi, Yohann; Ford, John C.; Solorzano, Gabriel; Erho, Nicholas; Balagurunathan, Yoganand; Punnen, Sanoj; Davicioni, Elai; Gillies, Robert J.; Pollack, Alan	TRANSLATIONAL CANCER RESEARCH	2016	5	4	38	7,6
Metabolic Reprogramming and Dependencies Associated with Epithelial Cancer Stem Cells Independent of the Epithelial-Mesenchymal Transition Program	Aguilar, Esther; Marin de Mas, Igor; Zodda, Erika; Marin, Silvia; Morrish, Fionnuala; Selivanov, Vitaly; Meca-Cortes, Oscar; Delowar, Hossain; Pons, Monica; Izquierdo, Ines; Celia-Terrassa, Toni; de Atauri, Pedro; Centelles, Josep J.; Hockenbery, David; Thomson, Timothy M.; Cascante, Marta	STEM CELLS	2016	34	5	38	7,6
Transcriptional profiling analysis and functional prediction of long noncoding RNAs in cancer	Yuan, Jiao; Yue, Haiyan; Zhang, Meiying; Luo, Jianjun; Liu, Lihui; Wu, Wei; Xiao, Tengfei; Chen, Xiaowei; Chen, Xiaomin; Zhang, Dongdong; Xing, Rui; Tong, Xin; Wu, Nan; Zhao, Jian; Lu, Youyong; Guo, Mingzhou; Chen, Runsheng	ONCOTARGET	2016	7	7	38	7,6
Metabolic markers in blood can separate prostate cancer from benign prostatic hyperplasia	Giskeodegard, Guro F.; Hansen, Ailin Falkmo; Bertilsson, Helena; Gonzalez, Susana Villa; Kristiansen, Kare Andre; Bruheim, Per; Mjos, Svein A.; Angelsen, Anders; Bathen, Tone Frost; Tessem, May-Britt	BRITISH JOURNAL OF CANCER	2015	113	12	38	6,33

TLR9 signaling through NF-kappa B/RELA and STAT3 promotes tumor-propagating potential of prostate cancer cells	Moreira, Dayson; Zhang, Qifang; Hossain, Dewan Md S.; Nechaev, Sergey; Li, Haiqing; Kowolik, Claudia M.; D'Apuzzo, Massimo; Forman, Stephen; Jones, Jeremy; Pal, Sumanta K.; Kortylewski, Marcin	ONCOTARGET	2015	6	19	38	6,33
Flux balance analysis predicts essential genes in clear cell renal cell carcinoma metabolism	Gatto, Francesco; Miess, Heike; Schulze, Almut; Nielsen, Jens	SCIENTIFIC REPORTS	2015	5		38	6,33
Background, Current Role, and Potential Applications of Radiogenomics	Pinker, Katja; Shitano, Fuki; Sala, Evis; Do, Richard K.; Young, Robert J.; Wibmer, Andreas G.; Hricak, Hedvig; Sutton, Elizabeth J.; Morris, Elizabeth A.	JOURNAL OF MAGNETIC RESONANCE IMAGING	2018	47	3	37	12,33
Clinical variability and molecular heterogeneity in prostate cancer	Shoag, Jonathan; Barbieri, Christopher E.	ASIAN JOURNAL OF ANDROLOGY	2016	18	4	37	7,4
Molecular evidence that invasive adenocarcinoma can mimic prostatic intraepithelial neoplasia (PIN) and intraductal carcinoma through retrograde glandular colonization	Haffner, Michael C.; Weier, Christopher; Xu, Meng Meng; Vaghasia, Ajay; Guerel, Bora; Guemueskaya, Berrak; Esopi, David M.; Fedor, Helen; Tan, Hsueh-Li; Kulac, Ibrahim; Hicks, Jessica; Isaacs, William B.; Lotan, Tamara L.; Nelson, William G.; Yegnasubramanian, Srinivasan; De Marzo, Angelo M.	JOURNAL OF PATHOLOGY	2016	238	1	37	7,4
Expression of androgen receptor splice variants in clinical breast cancers	Hickey, Theresa E.; Irvine, Connie M.; Dvinge, Heidi; Tarulli, Gerard A.; Hanson, Adrienne R.; Ryan, Natalie K.; Pickering, Marie A.; Birrell, Stephen N.; Hu, Dong Gui; Mackenzie, Peter I.; Russell, Roslin; Caldas, Carlos; Raj, Ganesh V.; Dehm, Scott M.; Plymate, Stephen R.; Bradley, Robert K.; Tilley, Wayne D.; Selth, Luke A.	ONCOTARGET	2015	6	42	37	6,17

The GALNT9, BNC1 and CCDC8 genes are frequently epigenetically dysregulated in breast tumours that metastasise to the brain	Pangeni, Rajendra P.; Channathodiyil, Prasanna; Huen, David S.; Eagles, Lawrence W.; Johal, Balraj K.; Pasha, Dawar; Hadjistephanou, Natasa; Nevell, Oliver; Davies, Claire L.; Adewumi, Ayobami I.; Khanom, Hamida; Samra, Ikroop S.; Buzatto, Vanessa C.; Chandrasekaran, Preethi; Shinawi, Thoraia; Dawson, Timothy P.; Ashton, Katherine M.; Davis, Charles; Brodbelt, Andrew R.; Jenkinson, Michael D.; Bieche, Ivan; Latif, Farida; Darling, John L.; Warr, Tracy J.; Morris, Mark R.	CLINICAL EPIGENETICS	2015	7		37	6,17
Unraveling the molecular repertoire of tears as a source of biomarkers: Beyond ocular diseases	Pieragostino, Damiana; D'Alessandro, Michele; di Ioia, Maria; Di Ilio, Carmine; Sacchetta, Paolo; Del Boccio, Piero	PROTEOMICS CLINICAL APPLICATIONS	2015	9	1-2	37	6,17
Clinical and molecular features of treatment-related neuroendocrine prostate cancer	Akamatsu, Shusuke; Inoue, Takahiro; Ogawa, Osamu; Gleave, Martin E.	INTERNATIONAL JOURNAL OF UROLOGY	2018	25	4	36	12
Cell-Cycle-Targeting MicroRNAs as Therapeutic Tools against Refractory Cancers	Hydbring, Per; Wang, Yinan; Fassl, Anne; Li, Xiaoting; Matia, Veronica; Otto, Tobias; Choi, Yoon Jong; Sweeney, Katharine E.; Suski, Jan M.; Yin, Hao; Bogorad, Roman L.; Goel, Shom; Yuzugullu, Haluk; Kauffman, Kevin J.; Yang, Junghoon; Jin, Chong; Li, Yingxiang; Floris, Davide; Swanson, Richard; Ng, Kimmie; Sicinska, Ewa; Anders, Lars; Zhao, Jean J.; Polyak, Kornelia; Anderson, Daniel G.; Li, Cheng; Sicinski, Piotr	CANCER CELL	2017	31	4	36	9
Targeting mutant p53 in cancer: a long road to precision therapy	Mantovani, Fiamma; Walerych, Dawid; Del Sal, Giannino	FEBS JOURNAL	2017	284	6	36	9
Targeting the SR-B1 Receptor as a Gateway for Cancer Therapy and Imaging	Mooberry, Linda K.; Sabnis, Nirupama A.; Panchoo, Marlyn; Nagarajan, Bhavani; Lacko, Andras G.	FRONTIERS IN PHARMACOLOGY	2016	7		36	7,2
When Genome Maintenance Goes Badly Awry	Kass, Elizabeth M.; Moynahan, Mary Ellen; Jasin, Maria	MOLECULAR CELL	2016	62	5	36	7,2

The marine triterpene glycoside frondoside A exhibits activity in vitro and in vivo in prostate cancer	Dyshlovoy, Sergey A.; Menchinskaya, Ekaterina S.; Venz, Simone; Rast, Stefanie; Amann, Kerstin; Hauschild, Jessica; Otte, Katharina; Kalinin, Vladimir I.; Silchenko, Alexandra S.; Avilov, Sergey A.; Alsdorf, Winfried; Madanchi, Ramin; Bokemeyer, Carsten; Schumacher, Udo; Walther, Reinhard; Aminin, Dmitry L.; Fedorov, Sergey N.; Shubina, Larisa K.; Stonik, Valentin A.; Balabanov, Stefan; Honecker, Friedemann; von Amsberg, Gunhild	INTERNATIONAL JOURNAL OF CANCER	2016	138	10	36	7,2
Integration of Metabolomics and Transcriptomics Reveals Major Metabolic Pathways and Potential Biomarker Involved in Prostate Cancer	Ren, Shancheng; Shao, Yaping; Zhao, Xinjie; Hong, Christopher S.; Wang, Fubo; Lu, Xin; Li, Jia; Ye, Guozhu; Yan, Min; Zhuang, Zhengping; Xu, Chuanliang; Xu, Guowang; Sun, Yinghao	MOLECULAR & CELLULAR PROTEOMICS	2016	15	1	36	7,2
Androgen receptor profiling predicts prostate cancer outcome	Stelloo, Suzan; Nevedomskaya, Ekaterina; van der Poel, Henk G.; de Jong, Jeroen; van Leenders, Geert J. L. H.; Jenster, Guido; Wessels, Lodewyk F. A.; Bergman, Andries M.; Zwart, Wilbert	EMBO MOLECULAR MEDICINE	2015	7	11	36	6
Differential regulation of metabolic pathways by androgen receptor (AR) and its constitutively active splice variant, AR-V7, in prostate cancer cells	Shafi, Ayesha A.; Putluri, Vasanta; Arnold, James M.; Tsouko, Efrosini; Maity, Suman; Roberts, Justin M.; Coarfa, Cristian; Frigo, Daniel E.; Putluri, Nagireddy; Sreekumar, Arun; Weigel, Nancy L.	ONCOTARGET	2015	6	31	36	6
C/D-box snoRNA-derived RNA production is associated with malignant transformation and metastatic progression in prostate cancer	Martens-Uzunova, Elena S.; Hoogstrate, Youri; Kalsbeek, Anton; Pigmans, Bas; Vredenbregt-van den Berg, Mirella; Dits, Natasja; Nielsen, Soren Jensby; Baker, Adam; Visakorpi, Tapio; Bangma, Chris; Jenster, Guido	ONCOTARGET	2015	6	19	36	6
Top2a identifies and provides epigenetic rationale for novel combination therapeutic strategies for aggressive prostate cancer	Kirk, Jason S.; Schaarschuch, Kevin; Dalimov, Zafardjan; Lasorsa, Elena; Ku, ShengYu; Ramakrishnan, Swathi; Hu, Qiang; Azabdaftari, Gissou; Wang, Jianmin; Pili, Roberto; Ellis, Leigh	ONCOTARGET	2015	6	5	36	6

CHD1 loss sensitizes prostate cancer to DNA damaging therapy by promoting error-prone double-strand break repair	Shenoy, T. R.; Boysen, G.; Wang, M. Y.; Xu, Q. Z.; Guo, W.; Koh, F. M.; Wang, C.; Zhang, L. Z.; Wang, Y.; Gil, V.; Aziz, S.; Christova, R.; Rodrigues, D. N.; Crespo, M.; Rescigno, P.; Tunariu, N.; Riisnaes, R.; Zafeiriou, Z.; Flohr, P.; Yuan, W.; Knight, E.; Swain, A.; Ramalho-Santos, M.; Xu, D. Y.; de Bono, J.; Wu, H.	ANNALS OF ONCOLOGY	2017	28	7	35	8,75
Genomic Insight into the Role of lncRNAs in Cancer Susceptibility	Gao, Ping; Wei, Gong-Hong	INTERNATIONAL JOURNAL OF MOLECULAR SCIENCES	2017	18	6	35	8,75
Prostate cancer heterogeneity: Discovering novel molecular targets for therapy	Ciccarese, Chiara; Massari, Francesco; Iacovelli, Roberto; Fiorentino, Michelangelo; Montironi, Rodolfo; Di Nunno, Vincenzo; Giunchi, Francesca; Brunelli, Matteo; Tortora, Giampaolo	CANCER TREATMENT REVIEWS	2017	54		35	8,75
Long non-coding RNA urothelial carcinoma associated 1 (UCA1) mediates radiation response in prostate cancer	Ghiam, Alireza Fotouhi; Taeb, Samira; Huang, Xiaoyong; Huang, Vincent; Ray, Jessica; Scarcello, Seville; Hoey, Christianne; Jahangiri, Sahar; Fokas, Emmanouil; Loblaw, Andrew; Bristow, Robert G.; Vesprini, Danny; Boutros, Paul; Liu, Stanley K.	ONCOTARGET	2017	8	3	35	8,75
Upregulation of long non-coding RNA PRNCR1 in colorectal cancer promotes cell proliferation and cell cycle progression	Yang, Liu; Qiu, Mantang; Xu, Youtao; Wang, Jie; Zheng, Yanyan; Li, Ming; Xu, Lin; Yin, Rong	ONCOLOGY REPORTS	2016	35	1	35	7
Transcriptome Sequencing Reveals PCAT5 as a Novel ERG-Regulated Long Noncoding RNA in Prostate Cancer	Ylipaa, Antti; Kivinummi, Kati; Kohvakka, Annika; Annala, Matti; Latonen, Leena; Scaravilli, Mauro; Kartasalo, Kimmo; Leppanen, Simo-Pekka; Karakurt, Serdar; Seppala, Janne; Yli-Harja, Olli; Tammela, Teuvo L. J.; Zhang, Wei; Visakorpi, Tapio; Nykter, Matti	CANCER RESEARCH	2015	75	19	35	5,83

DNA Damage Response Assessments in Human Tumor Samples Provide Functional Biomarkers of Radiosensitivity	Willers, Henning; Gheorghiu, Liliana; Liu, Qi; Efstathiou, Jason A.; Wirth, Lori J.; Krause, Mechthild; von Neubeck, Claere	SEMINARS IN RADIATION ONCOLOGY	2015	25	4	35	5,83
Potent organo-osmium compound shifts metabolism in epithelial ovarian cancer cells	Hearn, Jessica M.; Romero-Canelon, Isolda; Munro, Alison F.; Fu, Ying; Pizarro, Ana M.; Garnett, Mathew J.; McDermott, Ultan; Carragher, Neil O.; Sadler, Peter J.	PROCEEDINGS OF THE NATIONAL ACADEMY OF SCIENCES OF THE UNITED STATES OF AMERICA	2015	112	29	35	5,83
Metabolomics-Derived Prostate Cancer Biomarkers: Fact or Fiction?	Kumar, Deepak; Gupta, Ashish; Mandhani, Anil; Sankhwar, Satya Narain	JOURNAL OF PROTEOME RESEARCH	2015	14	3	35	5,83
Comprehensive Evaluation of Programmed Death-Ligand 1 Expression in Primary and Metastatic Prostate Cancer	Haffner, Michael C.; Guner, Gunes; Taheri, Diana; Netto, George J.; Palsgrove, Doreen N.; Zheng, Qizhi; Guedes, Liana Benevides; Kim, Kunhwa; Tsai, Harrison; Esopi, David M.; Lotan, Tamara L.; Sharma, Rajni; Meeker, Alan K.; Chinnaiyan, Arul M.; Nelson, William G.; Yegnasubramania, Srinivasan; Luo, Jun; Mehra, Rohit; Antonarakis, Emmanuel S.; Drake, Charles G.; de Marzo, Angelo M.	AMERICAN JOURNAL OF PATHOLOGY	2018	188	6	34	11,33
Nutrigenomics in cancer: Revisiting the effects of natural compounds	Braicu, Cornelia; Mehterov, Nikolay; Vladimirov, Boyan; Sarafian, Victoria; Nabavi, Seyed Mohammad; Atanasov, Atanas G.; Berindan-Neagoe, Ioana	SEMINARS IN CANCER BIOLOGY	2017	46		34	8,5
miR-143-3p targeting LIM domain kinase 1 suppresses the progression of triple-negative breast cancer cells	Li, Dengfeng; Hu, Jiashu; Song, Hongming; Xu, Hui; Wu, Chengyang; Zhao, Bingkun; Xie, Dan; Wu, Tianqi; Zhao, Junyong; Fang, Lin	AMERICAN JOURNAL OF TRANSLATIONAL RESEARCH	2017	9	5	34	8,5

Individual patient data meta-analysis shows a significant association between the ATM rs1801516 SNP and toxicity after radiotherapy in 5456 breast and prostate cancer patients	Andreassen, Christian Nicolaj; Rosenstein, Barry S.; Kerns, Sarah L.; Ostrer, Harry; De Ruysscher, Dirk; Cesaretti, Jamie A.; Barnett, Gillian C.; Dunning, Alison M.; Dorling, Leila; West, Catharine M. L.; Burnet, Neil G.; Elliott, Rebecca; Coles, Charlotte; Hall, Emma; Fachal, Laura; Vega, Ana; Gomez-Caamano, Antonio; Talbot, Christopher J.; Symonds, R. Paul; De Ruyck, Kim; Thierens, Hubert; Ost, Piet; Chang-Claude, Jenny; Seibold, Petra; Popanda, Odilia; Overgaard, Marie; Dearnaley, David; Sydes, Matthew R.; Azria, David; Koch, Christine Anne; Parliament, Matthew; Blackshaw, Michael; Sia, Michael; Fuentes-Raspall, Maria J.; Ramon y Cajal, Teresa; Barnadas, Agustin; Vesprini, Danny; Gutierrez-Enriquez, Sara; Molla, Meritxell; Diez, Orland; Yarnold, John R.; Overgaard, Jens; Bentzen, Soren M.; Alsner, Jan	RADIOTHERAPY AND ONCOLOGY	2016	121	3	34	6,8
The long non-coding RNA EPB41L4A-AS2 inhibits tumor proliferation and is associated with favorable prognoses in breast cancer and other solid tumors	Xu, Shouping; Wang, Peiyuan; You, Zilong; Meng, Hongxue; Mu, Guannan; Bai, Xianan; Zhang, Guangwen; Zhang, Jinfeng; Pang, Da	ONCOTARGET	2016	7	15	34	6,8
Proteomic Profiling of Serum-Derived Exosomes from Ethnically Diverse Prostate Cancer Patients	Turay, David; Khan, Salma; Osterman, Carlos J. Diaz; Curtis, Matthew P.; Khaira, Balreet; Neidigh, Jonathan W.; Mirshahidi, Saied; Casiano, Carlos A.; Wall, Nathan R.	CANCER INVESTIGATION	2016	34	1	34	6,8
Adverse Pathologic Features at Radical Prostatectomy: Effect of Preoperative Risk on Oncologic Outcomes	Imnadze, Mariam; Sjoberg, Daniel D.; Vickers, Andrew J.	EUROPEAN UROLOGY	2016	69	1	34	6,8

Long Noncoding RNAs in Urine Are Detectable and May Enable Early Detection of Acute T Cell-Mediated Rejection of Renal Allografts	Lorenzen, Johan M.; Schauerte, Celina; Koelling, Melte; Huebner, Anika; Knapp, Monika; Haller, Hermann; Thuml, Thomas	CLINICAL CHEMISTRY	2015	61	12	34	5,67
A multicenter study shows PTEN deletion is strongly associated with seminal vesicle involvement and extracapsular extension in localized prostate cancer	Troyer, Dean A.; Jamaspishvili, Tamara; Wei, Wei; Feng, Ziding; Good, Jennifer; Hawley, Sarah; Fazli, Ladan; McKenney, Jesse K.; Simko, Jeff; Hurtado-Coll, Antonio; Carroll, Peter R.; Gleave, Martin; Lance, Raymond; Lin, Daniel W.; Nelson, Peter S.; Thompson, Ian M.; True, Lawrence D.; Brooks, James D.; Squire, Jeremy A.	PROSTATE	2015	75	11	34	5,67
Radiogenomics helps to achieve personalized therapy by evaluating patient responses to radiation treatment	Guo, Zhen; Shu, Yan; Zhou, Honghao; Zhang, Wei; Wang, Hui	CARCINOGENESIS	2015	36	3	34	5,67
Generalizability of established prostate cancer risk variants in men of African ancestry	Han, Ying; Signorello, Lisa B.; Strom, Sara S.; Kittles, Rick A.; Rybicki, Benjamin A.; Stanford, Janet L.; Goodman, Phyllis J.; Berndt, Sonja I.; Carpten, John; Casey, Graham; Chu, Lisa; Conti, David V.; Rand, Kristin A.; Diver, W. Ryan; Hennis, Anselm J. M.; John, Esther M.; Kibel, Adam S.; Klein, Eric A.; Kolb, Suzanne; Le Marchand, Loic; Leske, M. Cristina; Murphy, Adam B.; Neslund-Dudas, Christine; Park, Jong Y.; Pettaway, Curtis; Rebbeck, Timothy R.; Gapstur, Susan M.; Zheng, S. Lilly; Wu, Suh-Yuh; Witte, John S.; Xu, Jianfeng; Isaacs, William; Ingles, Sue A.; Hsing, Ann; Easton, Douglas F.; Eeles, Rosalind A.; Schumacher, Fredrick R.; Chanock, Stephen; Nemesure, Barbara; Blot, William J.; Stram, Daniel O.; Henderson, Brian E.; Haiman, Christopher A.	INTERNATIONAL JOURNAL OF CANCER	2015	136	5	34	5,67

Polyphenols: dietary assessment and role in the prevention of cancers	Rothwell, Joseph A.; Knaze, Viktoria; Zamora-Ros, Raul	CURRENT OPINION IN CLINICAL NUTRITION AND METABOLIC CARE	2017	20	6	33	8,25
Metabolic characterization and pathway analysis of berberine protects against prostate cancer	Li, Xianna; Zhang, Aihua; Sun, Hui; Liu, Zhidong; Zhang, Tianlei; Qiu, Shi; Liu, Liang; Wang, Xijun	ONCOTARGET	2017	8	39	33	8,25
Improving radiotherapy in cancer treatment: Promises and challenges	Chen, Helen H. W.; Kuo, Macus Tien	ONCOTARGET	2017	8	37	33	8,25
Exome Sequencing of African-American Prostate Cancer Reveals Loss-of-Function ERF Mutations	Huang, Franklin W.; Mosquera, Juan Miguel; Garofalo, Andrea; Oh, Coyin; Baco, Maria; Amin-Mansour, Ali; Rabasha, Bokang; Bahl, Samira; Mullane, Stephanie A.; Robinson, Brian D.; Aldubayan, Saud; Khani, Francesca; Karir, Beerinder; Kim, Eejung; Chimene-Weiss, Jeremy; Hofree, Matan; Romanel, Alessandro; Osborne, Joseph R.; Kim, Jong Wook; Azabdaftari, Gissou; Woloszynska-Read, Anna; Sfanos, Karen; De Marzo, Angelo M.; Demichelis, Francesca; Gabriel, Stacey; Van Allen, Eliezer M.; Mesirov, Jill; Tamayo, Pablo; Rubin, Mark A.; Powell, Isaac J.; Garraway, Levi A.	CANCER DISCOVERY	2017	7	9	33	8,25
Label-free isolation of prostate circulating tumor cells using Vortex microfluidic technology	Renier, Corinne; Pao, Edward; Che, James; Liu, Haiyan E.; Lemaire, Clementine A.; Matsumoto, Melissa; Triboulet, Melanie; Srivinas, Sandy; Jeffrey, Stefanie S.; Rettig, Matthew; Kulkarni, Rajan P.; Di Carlo, Dino; Sollier-Christen, Elodie	NPJ PRECISION ONCOLOGY	2017	1		33	8,25

LincRNa-p21: function and mechanism in cancer	Chen, Shaoyun; Liang, Hairong; Yang, Hui; Zhou, Kairu; Xu, Longmei; Liu, Jiaxian; Lai, Bei; Song, Li; Luo, Hao; Peng, Jianming; Liu, Zhidong; Xiao, Yongmei; Chen, Wen; Tang, Huanwen	MEDICAL ONCOLOGY	2017	34	5	33	8,25
Targeted proteomics in urinary extracellular vesicles identifies biomarkers for diagnosis and prognosis of prostate cancer	Sequeiros, Tamara; Rigau, Marina; Chiva, Cristina; Montes, Melania; Garcia-Grau, Iolanda; Garcia, Marta; Diaz, Sherley; Celma, Ana; Bijnsdorp, Irene; Campos, Alex; Di Mauro, Primiano; Borros, Salvador; Reventos, Jaume; Doll, Andreas; Paciucci, Rosanna; Pegtel, Michiel; de Torres, Ines; Sabido, Eduard; Morote, Juan; Olivan, Mireia	ONCOTARGET	2017	8	3	33	8,25
Liquid biopsy: ready to guide therapy in advanced prostate cancer?	Hegemann, Miriam; Stenzl, Arnulf; Bedke, Jens; Chi, Kim N.; Black, Peter C.; Todenhofer, Tilman	BJU INTERNATIONAL	2016	118	6	33	6,6
Identification of candidate anti-cancer molecular mechanisms of compound kushen injection using functional genomics	Qu, Zhipeng; Cui, Jian; Harata-Lee, Yuka; Aung, Thazin Nwe; Feng, Qianjin; Raison, Joy M.; Kortschak, Robert Daniel; Adelson, David L.	ONCOTARGET	2016	7	40	33	6,6
Proteomics analysis of malignant and benign prostate tissue by 2D DIGE/MS reveals new insights into proteins involved in prostate cancer	Davalieva, Katarina; Kostovska, Ivana Maleva; Kiprijanovska, Sanja; Markoska, Katerina; Kubelka-Sabit, Katerina; Filipovski, Vanja; Stavridis, Sotir; Stankov, Oliver; Komina, Selim; Petrusevska, Gordana; Polenakovic, Momir	PROSTATE	2015	75	14	33	5,5
Elevated expression of UBE2T exhibits oncogenic properties in human prostate cancer	Wen, Mingxin; Kwon, Yongwon; Wang, Yongsheng; Mao, Jian-Hua; Wei, Guangwei	ONCOTARGET	2015	6	28	33	5,5
Novel biomarkers and genomic tests in prostate cancer: a critical analysis	Falzarano, S. M.; Ferro, M.; Bollito, E.; Klein, E. A.; Carrieri, G.; Magi-Galluzzi, C.	MINERVA UROLOGICA E NEFROLOGICA	2015	67	3	33	5,5
Ligand-dependent genomic function of glucocorticoid receptor in triple-negative breast cancer	Chen, Zhong; Lan, Xun; Wu, Dayong; Sunkel, Benjamin; Ye, Zhenqing; Huang, Jiaoti; Liu, Zhihua; Clinton, Steven K.; Jin, Victor X.; Wang, Qianben	NATURE COMMUNICATIONS	2015	6		33	5,5

Transcriptome profiling of esophageal squamous cell carcinoma reveals a long noncoding RNA acting as a tumor suppressor	Wei, Guifeng; Luo, Huaxia; Sun, Yu; Li, Jiagen; Tian, Liqing; Liu, Wei; Liu, Lihui; Luo, Jianjun; He, Jie; Chen, Runsheng	ONCOTARGET	2015	6	19	33	5,5
Capillary zone electrophoresis on-line coupled to mass spectrometry: A perspective application for clinical proteomics	Pejchinovski, Martin; Hrnjez, Dajana; Ramirez-Torres, Adela; Bitsika, Vasiliki; Mermelekas, George; Vlahou, Antonia; Zuerbig, Petra; Mischak, Harald; Metzger, Jochen; Koeck, Thomas	PROTEOMICS CLINICAL APPLICATIONS	2015	9	5-6	33	5,5
Quantitative Nuclear Proteomics Identifies that miR-137-mediated EZH2 Reduction Regulates Resveratrol-induced Apoptosis of Neuroblastoma Cells	Ren, Xiaoqing; Bai, Xue; Zhang, Xuefei; Li, Zheyi; Tang, Lingfang; Zhao, Xuyang; Li, Zeyang; Ren, Yanfei; Wei, Shicheng; Wang, Qingsong; Liu, Cong; Ji, Jianguo	MOLECULAR & CELLULAR PROTEOMICS	2015	14	2	33	5,5
Proteomics analysis of urine reveals acute phase response proteins as candidate diagnostic biomarkers for prostate cancer	Davalieva, Katarina; Kiprijanovska, Sanja; Komina, Selim; Petrusevska, Gordana; Zografska, Natasha Chokrevska; Polenakovic, Momir	PROTEOME SCIENCE	2015	13		33	5,5
Genetics and biology of prostate cancer	Wang, Guocan; Zhao, Di; Spring, Denise J.; DePinho, Ronald A.	GENES & DEVELOPMENT	2018	32	17-18	32	10,67
Clinical Outcome of Prostate Cancer Patients with Germline DNA Repair Mutations: Retrospective Analysis from an International Study	Mateo, Joaquin; Cheng, Heather H.; Beltran, Himisha; Dolling, David; Xu, Wen; Pritchard, Colin C.; Mossop, Helen; Rescigno, Pasquale; Perez-Lopez, Raquel; Sailer, Verena; Kolinsky, Michael; Balasopoulou, Ada; Bertan, Claudia; Nanus, David M.; Tagawa, Scott T.; Thorne, Heather; Montgomery, Bruce; Carreira, Suzanne; Sandhu, Shahneen; Rubin, Mark A.; Nelson, Peter S.; de Bono, Johann S.	EUROPEAN UROLOGY	2018	73	5	32	10,67

Ability of a Genomic Classifier to Predict Metastasis and Prostate Cancer-specific Mortality after Radiation or Surgery based on Needle Biopsy Specimens	Nguyen, Paul L.; Haddad, Zaid; Ross, Ashley E.; Martin, Neil E.; Deheshi, Samineh; Lam, Lucia L. C.; Chelliserry, Jijumon; Tosoian, Jeffrey J.; Lotan, Tamara L.; Spratt, Daniel E.; Stoyanova, Radka S.; Punnen, Sanoj; Ong, Kaye; Buerki, Christine; Aranes, Maria; Kolisnik, Tyler; Margrave, Jennifer; Yousefi, Kasra; Choeurng, Voleak; Davicioni, Elai; Trock, Bruce J.; Kane, Christopher J.; Pollack, Alan; Davis, John W.; Feng, Felix Y.; Klein, Eric A.	EUROPEAN UROLOGY	2017	72	5	32	8
Bioorthogonal Labeling of Human Prostate Cancer Tissue Slice Cultures for Glycoproteomics	Spiciarich, David R.; Nolley, Rosalie; Maund, Sophia L.; Purcell, Sean C.; Herschel, Jason; Iavarone, Anthony T.; Peehl, Donna M.; Bertozzi, Carolyn R.	ANGEWANDTE CHEMIE-INTERNATIONAL EDITION	2017	56	31	32	8
Alternative splicing promotes tumour aggressiveness and drug resistance in African American prostate cancer	Wang, Bi-Dar; Ceniccola, Kristin; Hwang, Sujin; Andrawis, Ramez; Horvath, Anelia; Freedman, Jennifer A.; Olender, Jacqueline; Knapp, Stefan; Ching, Travers; Garmire, Lana; Patel, Vyomesh; Garcia-Blanco, Mariano A.; Patierno, Steven R.; Lee, Norman H.	NATURE COMMUNICATIONS	2017	8		32	8
AKT1 and AKT2 isoforms play distinct roles during breast cancer progression through the regulation of specific downstream proteins	Riggio, Marina; Perrone, Maria C.; Polo, Maria L.; Rodriguez, Maria J.; May, Maria; Abba, Martin; Lanari, Claudia; Novaro, Virginia	SCIENTIFIC REPORTS	2017	7		32	8
Prostate Cancer Genetics: Variation by Race, Ethnicity, and Geography	Rebbeck, Timothy R.	SEMINARS IN RADIATION ONCOLOGY	2017	27	1	32	8

Meta-analysis of Genome Wide Association Studies Identifies Genetic Markers of Late Toxicity Following Radiotherapy for Prostate Cancer	Kerns, Sarah L.; Dorling, Leila; Fachal, Laura; Bentzen, Soren; Pharoah, Paul D. P.; Barnes, Daniel R.; Gomez-Caamano, Antonio; Carballo, Ana M.; Dearnaley, David P.; Peleteiro, Paula; Gulliford, Sarah L.; Hall, Emma; Michailidou, Kyriaki; Carracedo, Angel; Sia, Michael; Stock, Richard; Stone, Nelson N.; Sydes, Matthew R.; Tyrer, Jonathan P.; Ahmed, Shahana; Parliament, Matthew; Ostrer, Harry; Rosenstein, Barry S.; Vega, Ana; Burnet, Neil G.; Dunning, Alison M.; Barnett, Gillian C.; West, Catharine M. L.	EBIOMEDICINE	2016	10		32	6,4
Application of a Clinical Whole-Transcriptome Assay for Staging and Prognosis of Prostate Cancer Diagnosed in Needle Core Biopsy Specimens	Knudsen, Beatrice S.; Kim, Hyung L.; Erho, Nicholas; Shin, Heesun; Alshatalfa, Mohammed; Lam, Lucia L. C.; Tenggara, Imelda; Chadwich, Karen; Van Der Kwast, Theo; Fleshner, Neil; Davicioni, Elai; Carroll, Peter R.; Cooperberg, Matthew R.; Chan, June M.; Simko, Jeffry P.	JOURNAL OF MOLECULAR DIAGNOSTICS	2016	18	3	32	6,4
Incorporation of tissue-based genomic biomarkers into localized prostate cancer clinics	Moschini, Marco; Spahn, Martin; Mattei, Agostino; Cheville, John; Karnes, R. Jeffrey	BMC MEDICINE	2016	14		32	6,4
Beyond proliferation: KLF5 promotes angiogenesis of bladder cancer through directly regulating VEGFA transcription	Gao, Yang; Wu, Kaijie; Chen, Yule; Zhou, Jiancheng; Du, Chong; Shi, Qi; Xu, Shan; Jia, Jing; Tang, Xiaoshuang; Li, Feng; Hui, Ke; He, Dalin; Guo, Peng	ONCOTARGET	2015	6	41	32	5,33
Evaluation of Antioxidative and Cytotoxic Activities of Streptomyces pluripotens MUSC 137 Isolated from Mangrove Soil in Malaysia	Ser, Hooi-Leng; Ab Mutalib, Nurul-Syakima; Yin, Wai-Fong; Chan, Kok-Gan; Goh, Bey-Hing; Lee, Learn-Han	FRONTIERS IN MICROBIOLOGY	2015	6		32	5,33
Molecular landscape of prostate cancer: Implications for current clinical trials	Khemlina, Galina; Ikeda, Sadakatsu; Kurzrock, Razelle	CANCER TREATMENT REVIEWS	2015	41	9	32	5,33

Prediction of Individual Genetic Risk to Prostate Cancer Using a Polygenic Score	Szulkin, Robert; Whitington, Thomas; Eklund, Martin; Aly, Markus; Eeles, Rosalind A.; Easton, Douglas; Kote-Jarai, ZSofia; Al Olama, Ali Amin; Benlloch, Sara; Muir, Kenneth; Giles, Graham G.; Southey, Melissa C.; Fitzgerald, Liesel M.; Henderson, Brian E.; Schumacher, Fredrick; Haiman, Christopher A.; Schleutker, Johanna; Wahlfors, Tiina; Tammela, Teuvo L. J.; Nordestgaard, Borge G.; Key, Tim J.; Travis, Ruth C.; Neal, David E.; Donovan, Jenny L.; Hamdy, Freddie C.; Pharoah, Paul; Pashayan, Nora; Khaw, Kay-Tee; Stanford, Janet L.; Thibodeau, Stephen N.; McDonnell, Shannon K.; Schaid, Daniel J.; Maier, Christiane; Vogel, Walther; Luedeke, Manuel; Herkommer, Kathleen; Kibel, Adam S.; Cybulski, Cezary; Lubinski, Jan; Kluzniak, Wojciech; Cannon-Albright, Lisa; Brenner, Hermann; Butterbach, Katja; Stegmaier, Christa; Park, Jong Y.; Sellers, Thomas; Lim, Hui-Yi; Slavov, Chavdar; Kaneva, Radka; Mitev, Vanio; Batra, Jyotsna; Clements, Judith A.; Spurdle, Amanda; Teixeira, Manuel R.; Paulo, Paula; Maia, Sofia; Pandha, Hardev; Michael, Agnieszka; Kierzek, Andrzej; Gronberg, Henrik; Wiklund, Fredrik	PROSTATE	2015	75	13	32	5,33
TRPM8 channel as a novel molecular target in androgen-regulated prostate cancer cells	Asuthkar, Swapna; Velpula, Kiran Kumar; Elustondo, Pia A.; Demirkhanyan, Lusine; Zakharian, Eleonora	ONCOTARGET	2015	6	19	32	5,33
The Androgen Receptor in Breast Cancer	Giovannelli, Pia; Di Donato, Marzia; Galasso, Giovanni; Di Zazzo, Erika; Bilancio, Antonio; Migliaccio, Antimo	FRONTIERS IN ENDOCRINOLOGY	2018	9		31	10,33

MiR-25-3p promotes the proliferation of triple negative breast cancer by targeting BTG2	Chen, Hua; Pan, Hong; Qian, Yi; Zhou, Wenbin; Liu, Xiaoan	MOLECULAR CANCER	2018	17		31	10,33
The Role of Gut Microbiome in the Pathogenesis of Prostate Cancer: A Prospective, Pilot Study	Golombos, David M.; Ayangbesan, Abimbola; O'Malley, Padraic; Lewicki, Patrick; Barlow, LaMont; Barbieri, Christopher E.; Chan, Chrystal; DuLong, Casey; Abu-Ali, Galeb; Huttenhower, Curtis; Scherr, Douglas S.	UROLOGY	2018	111		31	10,33
Nucleic Acid Aptamers: Emerging Applications in Medical Imaging, Nanotechnology, Neurosciences, and Drug Delivery	Rothlisberger, Pascal; Gasse, Cecile; Hollenstein, Marcel	INTERNATIONAL JOURNAL OF MOLECULAR SCIENCES	2017	18	11	31	7,75
Circulating mRNAs and miRNAs as candidate markers for the diagnosis and prognosis of prostate cancer	de Souza, Marilesia Ferreira; Kuasne, Hellen; Barros-Filho, Mateus de Camargo; Ciliao, Heloisa Lizotti; Marchi, Fabio Albuquerque; Fuganti, Paulo Emilio; Paschoal, Alexandre Rossi; Rogatto, Silvia Regina; de Syllos Colus, Ilce Mara	PLOS ONE	2017	12	9	31	7,75
Comprehensive proteomics analysis of exosomes derived from human seminal plasma	Yang, C.; Guo, W. -b.; Zhang, W. -s.; Bian, J.; Yang, J. -k.; Zhou, Q. -z.; Chen, M. -k.; Peng, W.; Qi, T.; Wang, C. -y.; Liu, C. -d.	ANDROLOGY	2017	5	5	31	7,75
Patient-derived xenografts as in vivo models for research in urological malignancies	Inoue, Takahiro; Terada, Naoki; Kobayashi, Takashi; Ogawa, Osamu	NATURE REVIEWS UROLOGY	2017	14	5	31	7,75
Comprehensive Drug Testing of Patient-derived Conditionally Reprogrammed Cells from Castration-resistant Prostate Cancer	Saeed, Khalid; Rahkama, Vesa; Eldfors, Samuli; Bychkov, Dmitry; Mpindi, John Patrick; Yadav, Bhagwan; Paavolainen, Lassi; Aittokallio, Tero; Heckman, Caroline; Wennerberg, Krister; Peehl, Donna M.; Horvath, Peter; Mirtti, Tuomas; Rannikko, Antti; Kallioniemi, Olli; Ostling, Paivi; af Hallstrom, Taija M.	EUROPEAN UROLOGY	2017	71	3	31	7,75

The Emergence of Precision Urologic Oncology: A Collaborative Review on Biomarker-driven Therapeutics	Barbieri, Christopher E.; Chinnaiyan, Arul M.; Lerner, Seth P.; Swanton, Charles; Rubin, Mark A.	EUROPEAN UROLOGY	2017	71	2	31	7,75
Multi-institutional Analysis Shows that Low PCAT-14 Expression Associates with Poor Outcomes in Prostate Cancer	White, Nicole M.; Zhao, Shuang G.; Zhang, Jin; Rozycki, Emily B.; Dang, Ha X.; McFadden, Sandra D.; Eteleeb, Abdallah M.; Alshalalfa, Mohammed; Vergara, Ismael A.; Erho, Nicholas; Arbeit, Jeffrey M.; Karnes, Robert Jeffrey; Den, Robert B.; Davicioni, Elai; Maher, Christopher A.	EUROPEAN UROLOGY	2017	71	2	31	7,75
Dysregulation of miR-212 Promotes Castration Resistance through hnRNPH1-Mediated Regulation of AR and AR-V7: Implications for Racial Disparity of Prostate Cancer	Yang, Yijun; Jia, Dingwu; Kim, Hogyoung; Elmageed, Zakaria Y. Abd; Datta, Amrita; Davis, Rodney; Srivastav, Sudesh; Moroz, Krzysztof; Crawford, Byron E.; Moparty, Krishnarao; Thomas, Raju; Hudson, Robert S.; Ambs, Stefan; Abdel-Mageed, Asim B.	CLINICAL CANCER RESEARCH	2016	22	7	31	6,2
Prostasomes as a source of diagnostic biomarkers for prostate cancer	Zijlstra, Carla; Stoorvogel, Willem	JOURNAL OF CLINICAL INVESTIGATION	2016	126	4	31	6,2
The Thr300Ala variant in ATG16L1 is associated with improved survival in human colorectal cancer and enhanced production of type I interferon	Grimm, Wesley A.; Messer, Jeannette S.; Murphy, Stephen F.; Nero, Thomas; Lodolce, James P.; Weber, Christopher R.; Logsdon, Mark F.; Bartulis, Sarah; Sylvester, Brooke E.; Springer, Amanda; Dougherty, Urszula; Niewold, Timothy B.; Kupfer, Sonia S.; Ellis, Nathan; Huo, Dezheng; Bissonnette, Marc; Boone, David L.	GUT	2016	65	3	31	6,2
Tumor-suppressive microRNAs (miR-26a/b, miR-29a/b/c and miR-218) concertedly suppressed metastasis-promoting LOXL2 in head and neck squamous cell carcinoma	Fukumoto, Ichiro; Kikkawa, Naoko; Matsushita, Ryosuke; Kato, Mayuko; Kurozumi, Akira; Nishikawa, Rika; Goto, Yusuke; Koshizuka, Keiichi; Hanazawa, Toyoyuki; Enokida, Hideki; Nakagawa, Masayuki; Okamoto, Yoshitaka; Seki, Naohiko	JOURNAL OF HUMAN GENETICS	2016	61	2	31	6,2

Virtues and Weaknesses of DNA Methylation as a Test for Cervical Cancer Prevention	Lorincz, Attila T.	ACTA CYTOLOGICA	2016	60	6	31	6,2
Targeting ion channels for cancer therapy by repurposing the approved drugs	Kale, Vijay Pralhad; Amin, Shantu G.; Pandey, Manoj K.	BIOCHIMICA ET BIOPHYSICA ACTA-BIOMEMBRANES	2015	1848	10	31	5,17
The Prediction of Radiotherapy Toxicity Using Single Nucleotide Polymorphism-Based Models: A Step Toward Prevention	Kerns, Sarah L.; Kundu, Suman; Oh, Jung Hun; Singhal, Sandeep K.; Janelsins, Michelle; Travis, Lois B.; Deasy, Joseph O.; Janssens, A. Cecile J. E.; Ostrer, Harry; Parliament, Matthew; Usmani, Nawaid; Rosenstein, Barry S.	SEMINARS IN RADIATION ONCOLOGY	2015	25	4	31	5,17
The genomic evolution of human prostate cancer	Mitchell, T.; Neal, D. E.	BRITISH JOURNAL OF CANCER	2015	113	2	31	5,17
Prostate cancer epigenetic biomarkers: next-generation technologies	Valdes-Mora, F.; Clark, S. J.	ONCOGENE	2015	34	13	31	5,17

Fine-mapping of the HNF1B multicancer locus identifies candidate variants that mediate endometrial cancer risk	Painter, Jodie N.; O'Mara, Tracy A.; Batra, Jyotsna; Cheng, Timothy; Lose, Felicity A.; Dennis, Joe; Michailidou, Kyriaki; Tyrer, Jonathan P.; Ahmed, Shahana; Ferguson, Kaltin; Healey, Catherine S.; Kaufmann, Susanne; Hillman, Kristine M.; Walpole, Carina; Moya, Leire; Pollock, Pamela; Jones, Angela; Howarth, Kimberley; Martin, Lynn; Gorman, Maggie; Hodgson, Shirley; Magdalena Echeverry De Polanco, Ma.; Sans, Monica; Carracedo, Angel; Castellvi-Bel, Sergi; Rojas-Martinez, Augusto; Santos, Erika; Teixeira, Manuel R.; Carvajal-Carmona, Luis; Shu, Xiao-Ou; Long, Jirong; Zheng, Wei; Xiang, Yong-Bing; Montgomery, Grant W.; Webb, Penelope M.; Scott, Rodney J.; McEvoy, Mark; Attia, John; Holliday, Elizabeth; Martin, Nicholas G.; Nyholt, Dale R.; Henders, Anjali K.; Fasching, Peter A.; Hein, Alexander; Beckmann, Matthias W.; Renner, Stefan P.; Doerk, Thilo; Hillemanns, Peter; Duerst, Matthias; Runnebaum, Ingo; Lambrechts, Diether; Coenegrachts, Lieve; Schrauwen, Stefanie; Amant, Frederic; Winterhoff, Boris; Dowdy, Sean C.; Goode, Ellen L.; Teoman, Attila; Salvesen, Helga B.; Trovik, Jone; Njolstad, Tormund S.; Werner, Henrica M. J.; Ashton, Katie; Proietto, Tony; Otton, Geoffrey; Tzortzatos, Gerasimos; Mints, Miriam; Tham, Emma; Hall, Per; Czene, Kamila; Liu, Jianjun; Li, Jingmei; Hopper,	HUMAN MOLECULAR GENETICS	2015	24	5	31	5,17
ER beta-Mediated Alteration of circATP2B1 and miR-204-3p Signaling Promotes Invasion of Clear Cell Renal Cell Carcinoma	Han, Zhenwei; Zhang, Yong; Sun, Yin; Chen, Jiaqi; Chang, Chawnshang; Wang, Xiaolu; Yeh, Shuyuan	CANCER RESEARCH	2018	78	10	30	10
Long non-coding RNAs on the stage of cervical cancer (review)	Dong, Junxue; Su, Manman; Chang, Weiqin; Zhang, Kun; Wu, Shuying; Xu, Tianmin	ONCOLOGY REPORTS	2017	38	4	30	7,5

Correlation of B7-H3 with androgen receptor, immune pathways and poor outcome in prostate cancer: an expression-based analysis	Benzon, B.; Zhao, S. G.; Haffner, M. C.; Takhar, M.; Erho, N.; Yousefi, K.; Hurley, P.; Bishop, J. L.; Tosoian, J.; Ghabili, K.; Alshalalfa, M.; Glavaris, S.; Simons, B. W.; Tran, P.; Davicioni, E.; Karnes, R. J.; Boudadi, K.; Antonarakis, E. S.; Schaeffer, E. M.; Drake, C. G.; Feng, F.; Ross, A. E.	PROSTATE CANCER AND PROSTATIC DISEASES	2017	20	1	30	7,5
Identifying fusion transcripts using next generation sequencing	Kumar, Shailesh; Razzaq, Sundus Khalid; Vo, Angie Duy; Gautam, Mamta; Li, Hui	WILEY INTERDISCIPLINARY REVIEWS-RNA	2016	7	6	30	6
Liquid Biopsy in Lung Cancer A Perspective From Members of the Pulmonary Pathology Society	Sholl, Lynette M.; Aisner, Dara L.; Allen, Timothy Craig; Beasley, Mary Beth; Cagle, Philip T.; Capelozzi, Vera L.; Dacic, Sanja; Hariri, Lida P.; Kerr, Keith M.; Lantuejoul, Sylvie; Mino-Kenudson, Mari; Raparia, Kirtee; Rekhtman, Natasha; Roy-Chowdhuri, Sinchita; Thunnissen, Eric; Tsao, Ming; Vivero, Marina; Yatabe, Yasushi	ARCHIVES OF PATHOLOGY & LABORATORY MEDICINE	2016	140	8	30	6
Six Novel Loci Associated with Circulating VEGF Levels Identified by a Meta-analysis of Genome-Wide Association Studies	Choi, Seung Hoan; Ruggiero, Daniela; Sorice, Rossella; Song, Ci; Nutile, Teresa; Smith, Albert Vernon; Concas, Maria Pina; Traglia, Michela; Barbieri, Caterina; Ndiaye, Ndeye Coumba; Stathopoulou, Maria G.; Lagou, Vasiliki; Maestrale, Giovanni Battista; Sala, Cinzia; Debette, Stephanie; Kovacs, Peter; Lind, Lars; Lamont, John; Fitzgerald, Peter; Toenjes, Anke; Gudnason, Vilmundur; Toniolo, Daniela; Pirastu, Mario; Bellenguez, Celine; Vasan, Ramachandran S.; Ingelsson, Erik; Leutenegger, Anne-Louise; Johnson, Andrew D.; DeStefano, Anita L.; Visvikis-Siest, Sophie; Seshadri, Sudha; Ciullo, Marina	PLOS GENETICS	2016	12	2	30	6

Getting to know the extracellular vesicle glycome	Gerlach, Jared Q.; Griffin, Matthew D.	MOLECULAR BIOSYSTEMS	2016	12	4	30	6
Therapeutic targeting of replicative immortality	Yaswen, Paul; MacKenzie, Karen L.; Keith, W. Nicol; Hentosh, Patricia; Rodier, Francis; Zhu, Jiyue; Firestone, Gary L.; Matheu, Ander; Carnero, Amancio; Bilsland, Alan; Sundin, Tabetha; Honoki, Kanya; Fujii, Hiromasa; Georgakilas, Alexandros G.; Amedei, Amedeo; Amin, Amr; Helferich, Bill; Boosani, Chandra S.; Guha, Gunjan; Ciriolo, Maria Rosa; Chen, Sophie; Mohammed, Sulma I.; Azmi, Asfar S.; Bhakta, Dipita; Halicka, Dorota; Niccolai, Elena; Aquilano, Katia; Ashraf, S. Salman; Nowsheen, Somaira; Yang, Xujuan	SEMINARS IN CANCER BIOLOGY	2015	35		30	5
Aberrant GLI1 Activation in DNA Damage Response, Carcinogenesis and Chemoresistance	Palle, Komaraiah; Mani, Chinnadurai; Tripathi, Kaushlendra; Athar, Mohammad	CANCERS	2015	7	4	30	5
TMPRSS2: ERG blocks neuroendocrine and luminal cell differentiation to maintain prostate cancer proliferation	Mounir, Z.; Lin, F.; Lin, V. G.; Korn, J. M.; Yu, Y.; Valdez, R.; Aina, O. H.; Buchwalter, G.; Jaffe, A. B.; Korpal, M.; Zhu, P.; Brown, M.; Cardiff, R. D.; Rocnik, J. L.; Yang, Y.; Pagliarini, R.	ONCOGENE	2015	34	29	30	5
A genetic variant of MDM4 influences regulation by multiple microRNAs in prostate cancer	Stegeman, Shane; Moya, Leire; Selth, Luke A.; Spurdle, Amanda B.; Clements, Judith A.; Batra, Jyotsna	ENDOCRINE-RELATED CANCER	2015	22	2	30	5
Secretome Analysis of an Osteogenic Prostate Tumor Identifies Complex Signaling Networks Mediating Cross-talk of Cancer and Stromal Cells Within the Tumor Microenvironment	Lee, Yu-Chen; Gajdosik, Martina Srajer; Josic, Djuro; Clifton, James G.; Logothetis, Christopher; Yu-Lee, Li-Yuan; Gallick, Gary E.; Maity, Sankar N.; Lin, Sue-Hwa	MOLECULAR & CELLULAR PROTEOMICS	2015	14	3	30	5

Chromosomal Instability in Cell-Free DNA Is a Serum Biomarker for Prostate Cancer	Schuetz, Ekkehard; Akbari, Mohammad R.; Beck, Julia; Urnovitz, Howard; Zhang, William W.; Bornemann-Kolatzki, Kirsten; Mitchell, William M.; Nam, Robert K.; Narod, Steven A.	CLINICAL CHEMISTRY	2015	61	1	30	5
Genetic insights into the morass of metastatic heterogeneity	Hunter, Kent W.; Amin, Ruhul; Deasy, Sarah; Ngoc-Han Ha; Wakefield, Lalage	NATURE REVIEWS CANCER	2018	18	4	29	9,67
The post-translational modification, SUMOylation, and cancer (Review)	Han, Zhi-Jian; Feng, Yan-Hu; Gu, Bao-Hong; Li, Yu-Min; Chen, Hao	INTERNATIONAL JOURNAL OF ONCOLOGY	2018	52	4	29	9,67
Genome-scale analysis to identify prognostic markers in patients with early-stage pancreatic ductal adenocarcinoma after pancreaticoduodenectomy	Liao, Xiwen; Huang, Ketuan; Huang, Rui; Liu, Xiaoguang; Han, Chuangye; Yu, Long; Yu, Tingdong; Yang, Chengkun; Wang, Xiangkun; Peng, Tao	ONCOTARGETS AND THERAPY	2017	10		29	7,25
Characters, functions and clinical perspectives of long non-coding RNAs	Wu, Ruifang; Su, Yuwen; Wu, Haijing; Dai, Yong; Zhao, Ming; Lu, Qianjin	MOLECULAR GENETICS AND GENOMICS	2016	291	3	29	5,8
Transcriptomic profiling of urine extracellular vesicles reveals alterations of CDH3 in prostate cancer	Royo, Felix; Zuniga-Garcia, Patricia; Torrano, Veronica; Loizaga, Ana; Sanchez-Mosquera, Pilar; Ugalde-Olano, Aitziber; Gonzalez, Esperanza; Cortazar, Ana R.; Palomo, Laura; Fernandez-Ruiz, Sonia; Lacasa-Viscasillas, Isabel; Berdasco, Maria; Sutherland, James D.; Barrio, Rosa; Zabala-Letona, Amaia; Martin-Martin, Natalia; Arruabarrena-Aristorena, Amaia; Valcarcel-Jimenez, Lorea; Caro-Maldonado, Alfredo; Gonzalez-Tampan, Jorge; Cachi-Fuentes, Guido; Esteller, Manel; Aransay, Ana M.; Unda, Miguel; Falcon-Perez, Juan M.; Carracedo, Arkaitz	ONCOTARGET	2016	7	6	29	5,8
The use of the NIS reporter gene for optimizing oncolytic virotherapy	Miller, Amber; Russell, Stephen J.	EXPERT OPINION ON BIOLOGICAL THERAPY	2016	16	1	29	5,8

A comprehensive analysis of genome-wide association studies to identify prostate cancer susceptibility loci for the Romanian population	Radavoi, George Daniel; Pricop, Catalin; Jinga, Viorel; Mates, Dana; Radoi, Viorica Elena; Jinga, Mariana; Ursu, Radu Roan; Bratu, Ovidiu Gabriel; Mischianu, Dan-Liviu Dorel; Iordache, Paul	ROMANIAN JOURNAL OF MORPHOLOGY AND EMBRYOLOGY	2016	57	2	29	5,8
METABOLOMICS IN MEDICAL SCIENCES - TRENDS, CHALLENGES AND PERSPECTIVES	Klupczynska, Agnieszka; Derezinski, Pawel; Kokot, Zenon J.	ACTA POLONIAE PHARMACEUTICA	2015	72	4	29	4,83
Theranos phenomenon: promises and fallacies	Diamandis, Eleftherios P.	CLINICAL CHEMISTRY AND LABORATORY MEDICINE	2015	53	7	29	4,83
Heterogeneity of PTEN and ERG expression in prostate cancer on core needle biopsies: implications for cancer risk stratification and biomarker sampling	Shah, Rajal B.; Bentley, James; Jeffery, Zach; DeMarzo, Angelo M.	HUMAN PATHOLOGY	2015	46	5	29	4,83
Novel long non-coding RNAs are specific diagnostic and prognostic markers for prostate cancer	Bottcher, Rene; Hoogland, A. Marije; Dits, Natasja; Verhoef, Esther I.; Kweldam, Charlotte; Waranecki, Piotr; Bangma, Chris H.; van Leenders, Geert J. L. H.; Jenster, Guido	ONCOTARGET	2015	6	6	29	4,83
Calcium calmodulin dependent kinase kinase 2-a novel therapeutic target for gastric adenocarcinoma	Subbannayya, Yashwanth; Syed, Nazia; Barbhuiya, Mustafa A.; Raja, Remya; Marimuthu, Arivusudar; Sahasrabuddhe, Nandini; Pinto, Sneha M.; Manda, Srikanth Srinivas; Renuse, Santosh; Manju, H. C.; Zameer, Mohammed Abdul Lateef; Sharma, Jyoti; Brait, Mariana; Srikumar, Kotteazeth; Carlos Roa, Juan; Kumar, M. Vijaya; Kumar, K. V. Veerendra; Prasad, T. S. Keshava; Ramaswamy, Girija; Kumar, Rekha Vijay; Pandey, Akhilesh; Gowda, Harsha; Chatterjee, Aditi	CANCER BIOLOGY & THERAPY	2015	16	2	29	4,83

Metabolomic profiling for the identification of novel diagnostic markers in prostate cancer	Lucarelli, Giuseppe; Rutigliano, Monica; Galleggiante, Vanessa; Giglio, Andrea; Palazzo, Silvano; Ferro, Matteo; Simone, Cristiano; Bettocchi, Carlo; Battaglia, Michele; Ditonno, Pasquale	EXPERT REVIEW OF MOLECULAR DIAGNOSTICS	2015	15	9	29	4,83
Proteomic signatures of extracellular vesicles secreted by nonmineralizing and mineralizing human osteoblasts and stimulation of tumor cell growth	Morhayim, Jess; van de Peppel, Jeroen; Demmers, Jeroen A. A.; Kocer, Gulistan; Nigg, Alex L.; van Driel, Marjolein; Chiba, Hideki; van Leeuwen, Johannes P.	FASEB JOURNAL	2015	29	1	29	4,83
Innovation in metabolomics to improve personalized healthcare	Cacciatore, Stefano; Loda, Massimo	COMPANION DIAGNOSTICS: FROM BIOMARKER IDENTIFICATION TO MARKET ENTRY	2015	1346		29	4,83
Progress in epigenetic histone modification analysis by mass spectrometry for clinical investigations	Onder, Ozlem; Sidoli, Simone; Carroll, Martin; Garcia, Benjamin A.	EXPERT REVIEW OF PROTEOMICS	2015	12	5	29	4,83
Phosphatidylserine: A cancer cell targeting biomarker	Sharma, Bhupender; Kanwar, Shamsher S.	SEMINARS IN CANCER BIOLOGY	2018	52		28	9,33
Comparison Between Adjuvant and Early-Salvage Postprostatectomy Radiotherapy for Prostate Cancer With Adverse Pathological Features	Hwang, William L.; Tendulkar, Rahul D.; Niemierko, Andrzej; Agrawal, Shree; Stephans, Kevin L.; Spratt, Daniel E.; Hearn, Jason W.; Koontz, Bridget F.; Lee, W. Robert; Michalski, Jeff M.; Pisansky, Thomas M.; Liauw, Stanley L.; Abramowitz, Matthew C.; Pollack, Alan; Moghanaki, Drew; Anscher, Mitchell S.; Den, Robert B.; Zietman, Anthony L.; Stephenson, Andrew J.; Efstathiou, Jason A.	JAMA ONCOLOGY	2018	4	5	28	9,33

Resistance to BET Inhibitor Leads to Alternative Therapeutic Vulnerabilities in Castration-Resistant Prostate Cancer	Pawar, Aishwarya; Gollavilli, Paradesi Naidu; Wang, Shaomeng; Asangani, Irfan A.	CELL REPORTS	2018	22	9	28	9,33
TOP2A and EZH2 Provide Early Detection of an Aggressive Prostate Cancer Subgroup	Labbe, David P.; Sweeney, Christopher J.; Brown, Myles; Galbo, Phillip; Rosario, Spencer; Wadosky, Kristine M.; Ku, Sheng-Yu; Sjostrom, Martin; Alshalalfa, Mohammed; Erho, Nicholas; Davicioni, Elai; Karnes, R. Jeffrey; Schaeffer, Edward M.; Jenkins, Robert B.; Den, Robert B.; Ross, Ashley E.; Bowden, Michaela; Huang, Ying; Gray, Kathryn P.; Feng, Felix Y.; Spratt, Daniel E.; Goodrich, David W.; Eng, Kevin H.; Ellis, Leigh	CLINICAL CANCER RESEARCH	2017	23	22	28	7
Impact of Therapy on Genomics and Transcriptomics in High-Risk Prostate Cancer Treated with Neoadjuvant Docetaxel and Androgen Deprivation Therapy	Beltran, Himisha; Wyatt, Alexander W.; Chedgy, Edmund C.; Donoghue, Adam; Annala, Matti; Warner, Evan W.; Beja, Kevin; Sigouros, Michael; Mo, Fan; Fazli, Ladan; Collins, Colin C.; Eastham, James; Morris, Michael; Taplin, Mary-Ellen; Sboner, Andrea; Halabi, Susan; Gleave, Martin E.	CLINICAL CANCER RESEARCH	2017	23	22	28	7
DNA methylation variations are required for epithelial-to-mesenchymal transition induced by cancer-associated fibroblasts in prostate cancer cells	Pistore, C.; Giannoni, E.; Colangelo, T.; Rizzo, F.; Magnani, E.; Muccillo, L.; Giurato, G.; Mancini, M.; Rizzo, S.; Riccardi, M.; Sahnane, N.; Del Vescovo, V.; Kishore, K.; Mandruzzato, M.; Macchi, F.; Pelizzola, M.; Denti, M. A.; Furlan, D.; Weisz, A.; Colantuoni, V.; Chiarugi, P.; Bonapace, I. M.	ONCOGENE	2017	36	40	28	7
Targeting multiple pro-apoptotic signaling pathways with curcumin in prostate cancer cells	Rivera, Mariela; Ramos, Yanilda; Rodriguez-Valentin, Madeline; Lopez-Acevedo, Sheila; Cubano, Luis A.; Zou, Jin; Zhang, Qiang; Wang, Guangdi; Boukli, Nawal M.	PLOS ONE	2017	12	6	28	7

Phosphorylation-induced conformational dynamics in an intrinsically disordered protein and potential role in phenotypic heterogeneity	Kulkarni, Prakash; Jolly, Mohit Kumar; Jia, Dongya; Mooney, Steven M.; Bhargava, Ajay; Kagohara, Luciane T.; Chen, Yihong; Hao, Pengyu; He, Yanan; Veltri, Robert W.; Grishaev, Alexander; Weninger, Keith; Levine, Herbert; Orban, John	PROCEEDINGS OF THE NATIONAL ACADEMY OF SCIENCES OF THE UNITED STATES OF AMERICA	2017	114	13	28	7
Defining RNA Small Molecule Affinity Landscapes Enables Design of a Small Molecule Inhibitor of an Oncogenic Noncoding RNA	Velagapudi, Sai Pradeep; Luo, Yiling; Tran, Tuan; Haniff, Hafeez S.; Nakai, Yoshio; Fallahi, Mohammad; Martinez, Gustavo J.; Childs-Disney, Jessica L.; Disney, Matthew D.	ACS CENTRAL SCIENCE	2017	3	3	28	7
Exosomal proteins as prostate cancer biomarkers in urine: From mass spectrometry discovery to immunoassay-based validation	Wang, Ling; Skotland, Tore; Berge, Viktor; Sandvig, Kirsten; Llorente, Alicia	EUROPEAN JOURNAL OF PHARMACEUTICAL SCIENCES	2017	98		28	7
PD-L1 promoter methylation is a prognostic biomarker for biochemical recurrence-free survival in prostate cancer patients following radical prostatectomy	Gevensleben, Heidrun; Holmes, Emily Eva; Goltz, Diane; Dietrich, Joern; Sailer, Verena; Ellinger, Joerg; Dietrich, Dimo; Kristiansen, Glen	ONCOTARGET	2016	7	48	28	5,6
Translational and clinical implications of the genetic landscape of prostate cancer	Spratt, Daniel E.; Zumsteg, Zachary S.; Feng, Felix Y.; Tomlins, Scott A.	NATURE REVIEWS CLINICAL ONCOLOGY	2016	13	10	28	5,6
Proteomics and peptidomics: moving toward precision medicine in urological malignancies	Di Meo, Ashley; Pasic, Maria D.; Yousef, George M.	ONCOTARGET	2016	7	32	28	5,6
Novel drugs that target the metabolic reprogramming in renal cell cancer	van der Mijn, Johannes C.; Panka, David J.; Geissler, Andrew K.; Verheul, Henk. M.; Mier, James W.	CANCER & METABOLISM	2016	4		28	5,6

Direct regulation of LAMP1 by tumor-suppressive microRNA-320a in prostate cancer	Okato, Atsushi; Goto, Yusuke; Kurozumi, Akira; Kato, Mayuko; Kojima, Satoko; Matsushita, Ryosuke; Yonemori, Masaya; Miyamoto, Kazutaka; Ichikawa, Tomohiko; Seki, Naohiko	INTERNATIONAL JOURNAL OF ONCOLOGY	2016	49	1	28	5,6
MiR-301a Regulates E-Cadherin Expression and Is Predictive of Prostate Cancer Recurrence	Nam, Robert K.; Benatar, Tania; Wallis, Christopher J. D.; Amemiya, Yutaka; Yang, Wenyi; Garbens, Alaina; Naeim, Magda; Sherman, Christopher; Sugar, Linda; Seth, Arun	PROSTATE	2016	76	10	28	5,6
Promoter methylation of the immune checkpoint receptor PD-1 (PDCD1) is an independent prognostic biomarker for biochemical recurrence-free survival in prostate cancer patients following radical prostatectomy	Goltz, Diane; Gevensleben, Heidrun; Dietrich, Joern; Ellinger, Joerg; Landsberg, Jennifer; Kristiansen, Glen; Dietrich, Dimo	ONCOIMMUNOLOGY	2016	5	10	28	5,6
Cancer stem cells: a potential target for cancer therapy	Qiu, Hong; Fang, Xiaoguang; Luo, Qi; Ouyang, Gaoliang	CELLULAR AND MOLECULAR LIFE SCIENCES	2015	72	18	28	4,67
Metabolomic profiling of hormone-dependent cancers: a bird's eye view	Lloyd, Stacy M.; Arnold, James; Sreekumar, Arun	TRENDS IN ENDOCRINOLOGY AND METABOLISM	2015	26	9	28	4,67
The Deubiquitinating Enzyme USP7 Regulates Androgen Receptor Activity by Modulating Its Binding to Chromatin	Chen, Shu-Ting; Okada, Maiko; Nakato, Ryuichiro; Izumi, Kosuke; Bando, Masashige; Shirahige, Katsuhiko	JOURNAL OF BIOLOGICAL CHEMISTRY	2015	290	35	28	4,67
Toward an integrated pipeline for protein biomarker development	Drabovich, Andrei P.; Martinez-Morillo, Eduardo; Diamandis, Eleftherios P.	BIOCHIMICA ET BIOPHYSICA ACTA-PROTEINS AND PROTEOMICS	2015	1854	6	28	4,67
MiR-187 Targets the Androgen-Regulated Gene ALDH1A3 in Prostate Cancer	Casanova-Salas, Irene; Masia, Esther; Arminan, Ana; Calatrava, Ana; Mancarella, Caterina; Rubio-Briones, Jose; Scotlandi, Katia; Vicent, Maria Jesus; Lopez-Guerrero, Jose Antonio	PLOS ONE	2015	10	5	28	4,67

A Novel Urinary Long Non-Coding RNA Transcript Improves Diagnostic Accuracy in Patients Undergoing Prostate Biopsy	Zhang, Wei; Ren, Shan-Cheng; Shi, Xiao-Lei; Liu, Ya-wei; Zhu, Ya-Sheng; Jing, Tai-Le; Wang, Fu-Bo; Chen, Rui; Xu, Chuan-Liang; Wang, Hui-Qing; Wang, Hai-Feng; Wang, Yan; Liu, Bing; Li, Yao-Ming; Fang, Zi-Yu; Guo, Fei; Lu, Xin; Shen, Dan; Gao, Xu; Hou, Jian-Guo; Sun, Ying-Hao	PROSTATE	2015	75	6	28	4,67
Label-free quantitative proteomic analysis reveals potential biomarkers and pathways in renal cell carcinoma	Zhao, Zuohui; Wu, Fei; Ding, Sentai; Sun, Liang; Liu, Zhao; Ding, Kejia; Lu, Jiaju	TUMOR BIOLOGY	2015	36	2	28	4,67
IRE1 alpha-XBP1s pathway promotes prostate cancer by activating c-MYC signaling	Sheng, Xia; Nenseth, Hatice Zeynep; Qu, Su; Kuzu, Omer F.; Frahnow, Turid; Simon, Lukas; Greene, Stephanie; Zeng, Qingping; Fazli, Ladan; Rennie, Paul S.; Mills, Ian G.; Danielsen, Havard; Theis, Fabian; Patterson, John B.; Jin, Yang; Saatcioglu, Fahri	NATURE COMMUNICATIONS	2019	10		27	13,5

Immunogenomic analyses associate immunological alterations with mismatch repair defects in prostate cancer	Rodrigues, Daniel Nava; Rescigno, Pasquale; Liu, David; Yuan, Wei; Carreira, Suzanne; Lambros, Maryou B.; Seed, George; Mateo, Joaquin; Riisnaes, Ruth; Mullane, Stephanie; Margolis, Claire; Miao, Diana; Miranda, Susana; Dolling, David; Clarke, Matthew; Bertan, Claudia; Crespo, Mateus; Boysen, Gunther; Ferreira, Ana; Sharp, Adam; Figueiredo, Ines; Keliher, Daniel; Aldubayan, Saud; Burke, Kelly P.; Sumanasuriya, Semini; Fontes, Mariane Sousa; Bianchini, Diletta; Zafeiriou, Zafeiris; Mendes, Larissa Sena Teixeira; Mouw, Kent; Schweizer, Michael T.; Pritchard, Colin C.; Salipante, Stephen; Taplin, Mary-Ellen; Beltran, Himisha; Rubin, Mark A.; Cieslik, Marcin; Robinson, Dan; Heath, Elizabeth; Schultz, Nikolaus; Armenia, Joshua; Abida, Wassim; Scher, Howard; Lord, Christopher; D'Andrea, Alan; Sawyers, Charles L.; Chinnaiyan, Arul M.; Alimonti, Andrea; Nelson, Peter S.; Drake, Charles G.; Van Allen, Eliezer M.; de Bono, Johann S.	JOURNAL OF CLINICAL INVESTIGATION	2018	128	10	27	9
Aptamer-based targeted therapy	Zhu, Guizhi; Chen, Xiaoyuan	ADVANCED DRUG DELIVERY REVIEWS	2018	134		27	9
Multi-layered prevention and treatment of chronic inflammation, organ fibrosis and cancer associated with canonical WNT/beta-catenin signaling activation (Review)	Katoh, Masaru	INTERNATIONAL JOURNAL OF MOLECULAR MEDICINE	2018	42	2	27	9
Dancing with the DNA damage response: next-generation anti-cancer therapeutic strategies	Minchom, Anna; Aversa, Caterina; Lopez, Juanita	THERAPEUTIC ADVANCES IN MEDICAL ONCOLOGY	2018	10		27	9

Revisiting the role of Wnt/beta-catenin signaling in prostate cancer	Schneider, Jeffrey A.; Logan, Susan K.	MOLECULAR AND CELLULAR ENDOCRINOLOGY	2018	462		27	9
Identifying DNA methylation biomarkers for non-endoscopic detection of Barrett's esophagus	Moinova, Helen R.; LaFramboise, Thomas; Lutterbaugh, James D.; Chandar, Apoorva Krishna; Dumot, John; Faulx, Ashley; Brock, Wendy; Cabrera, Omar De la Cruz; Guda, Kishore; Barnholtz-Sloan, Jill S.; Iyer, Prasad G.; Canto, Marcia I.; Wang, Jean S.; Shaheen, Nicholas J.; Thota, Prashanti N.; Willis, Joseph E.; Chak, Amitabh; Markowitz, Sanford D.	SCIENCE TRANSLATIONAL MEDICINE	2018	10	424	27	9
Somatic Superenhancer Duplications and Hotspot Mutations Lead to Oncogenic Activation of the KLF5 Transcription Factor	Zhang, Xiaoyang; Choi, Peter S.; Francis, Joshua M.; Gao, Galen F.; Campbell, Joshua D.; Ramachandran, Aruna; Mitsuishi, Yoichiro; Ha, Gavin; Shih, Juliann; Vazquez, Francisca; Tsherniak, Aviad; Taylor, Alison M.; Zhou, Jin; Wu, Zhong; Berger, Ashton C.; Giannakis, Marios; Hahn, William C.; Cherniack, Andrew D.; Meyerson, Matthew	CANCER DISCOVERY	2018	8	1	27	9
Precision medicine based on epigenomics: the paradigm of carcinoma of unknown primary	Moran, Sebastian; Martinez-Cardus, Anna; Boussios, Stergios; Esteller, Manel	NATURE REVIEWS CLINICAL ONCOLOGY	2017	14	11	27	6,75
A Pyrrole-Imidazole Polyamide Is Active against Enzalutamide-Resistant Prostate Cancer	Kurmis, Alexis A.; Yang, Fei; Welch, Timothy R.; Nickols, Nicholas G.; Dervan, Peter B.	CANCER RESEARCH	2017	77	9	27	6,75
Prostate cancer proteomics: Current trends and future perspectives for biomarker discovery	Tanase, Cristiana Pistol; Codrici, Elena; Popescu, Ionela Daniela; Mihai, Simona; Enciu, Ana-Maria; Necula, Laura Georgiana; Preda, Adrian; Ismail, Gener; Albulescu, Radu	ONCOTARGET	2017	8	11	27	6,75

Oxidative Stress Gene Expression Profile Correlates with Cancer Patient Poor Prognosis: Identification of Crucial Pathways Might Select Novel Therapeutic Approaches	Leone, Alessandra; Roca, Maria Serena; Ciardiello, Chiara; Costantini, Susan; Budillon, Alfredo	OXIDATIVE MEDICINE AND CELLULAR LONGEVITY	2017	2017		27	6,75
Extracellular Vesicles in Renal Pathophysiology	Pomatto, Margherita A. C.; Gai, Chiara; Bussolati, Benedetta; Camussi, Giovanni	FRONTIERS IN MOLECULAR BIOSCIENCES	2017	4		27	6,75
Regulation of TPD52 by antitumor microRNA-218 suppresses cancer cell migration and invasion in lung squamous cell carcinoma	Kumamoto, Tomohiro; Seki, Naohiko; Mataki, Hiroko; Mizuno, Keiko; Kamikawaji, Kazuto; Samukawa, Takuya; Koshizuka, Keiichi; Goto, Yusuke; Inoue, Hiromasa	INTERNATIONAL JOURNAL OF ONCOLOGY	2016	49	5	27	5,4
Methylation analyses in liquid biopsy	Lissa, Delphine; Robles, Ana I.	TRANSLATIONAL LUNG CANCER RESEARCH	2016	5	5	27	5,4
A cancer specific hypermethylation signature of the TERT promoter predicts biochemical relapse in prostate cancer: A retrospective cohort study	Castelo-Branco, Pedro; Leao, Ricardo; Lipman, Tatiana; Campbell, Brittany; Lee, Donghyun; Price, Aryeh; Zhang, Cindy; Heidari, Abolfazl; Stephens, Derek; Boerno, Stefan; Coelho, Hugo; Gomes, Ana; Domingos, Celia; Apolonio, Joana D.; Schaefer, Georg; Bristow, Robert G.; Schweiger, Michal R.; Hamilton, Robert; Zlotta, Alexandre; Figueiredo, Arnaldo; Klocker, Helmut; Sueltmann, Holger; Tabori, Uri	ONCOTARGET	2016	7	36	27	5,4
The role of glycans in the development and progression of prostate cancer	Munkley, Jennifer; Mills, Ian G.; Elliott, David J.	NATURE REVIEWS UROLOGY	2016	13	6	27	5,4
The importance of non-nuclear AR signaling in prostate cancer progression and therapeutic resistance	Zarif, Jelani C.; Miranti, Cindy K.	CELLULAR SIGNALLING	2016	28	5	27	5,4

Regulation of androgen receptor splice variant AR3 by PCGEM1	Zhang, Ziqiang; Zhou, Nanjiang; Huang, Jianguo; Ho, Tsui-Ting; Zhu, Zhuxian; Qiu, Zhongmin; Zhou, Xinchun; Bai, Chunxue; Wu, Fangting; Xu, Min; Mo, Yin-Yuan	ONCOTARGET	2016	7	13	27	5,4
Long Noncoding RNA RGMB-AS1 Indicates a Poor Prognosis and Modulates Cell Proliferation, Migration and Invasion in Lung Adenocarcinoma	Li, Ping; Zhang, Guojun; Li, Juan; Yang, Rui; Chen, Shanshan; Wu, Shujun; Zhang, Furui; Bai, Yong; Zhao, Huasi; Wang, Yuanyuan; Dun, Shaozhi; Chen, Xiaonan; Sun, Qianqian; Zhao, Guoqiang	PLOS ONE	2016	11	3	27	5,4
DNA methylome changes by estradiol benzoate and bisphenol A links early-life environmental exposures to prostate cancer risk	Cheong, Ana; Zhang, Xiang; Cheung, Yuk-Yin; Tang, Wan-yee; Chen, Jing; Ye, Shu-Hua; Medvedovic, Mario; Leung, Yuet-Kin; Prins, Gail S.; Ho, Shuk-Mei	EPIGENETICS	2016	11	9	27	5,4
Proteomics analysis of vesicles isolated from plasma and urine of prostate cancer patients using a multiplex, aptamer-based protein array	Welton, Joanne Louise; Brennan, Paul; Gurney, Mark; Webber, Jason Paul; Spary, Lisa Kate; Carton, David Gil; Falcon-Perez, Juan Manuel; Walton, Sean Peter; Mason, Malcolm David; Tabi, Zsuzsanna; Clayton, Aled	JOURNAL OF EXTRACELLULAR VESICLES	2016	5		27	5,4
Epigenetic dysregulation of K(Ca)3.1 channels induces poor prognosis in lung cancer	Bulk, Etmar; Ay, Anne-Sophie; Hammadi, Mehdi; Ouadid-Ahidouch, Halima; Schelhaas, Sonja; Hascher, Antje; Rohde, Christian; Thoennissen, Nils H.; Wiewrodt, Rainer; Schmidt, Eva; Marra, Alessandro; Hillejan, Ludger; Jacobs, Andreas H.; Klein, Hans-Ulrich; Dugas, Martin; Berdel, Wolfgang E.; Mueller-Tidow, Carsten; Schwab, Albrecht	INTERNATIONAL JOURNAL OF CANCER	2015	137	6	27	4,5

Plasma genetic and genomic abnormalities predict treatment response and clinical outcome in advanced prostate cancer	Xia, Shu; Kohli, Manish; Du, Meijun; Dittmar, Rachel L.; Lee, Adam; Nandy, Debashis; Yuan, Tiezheng; Guo, Yongchen; Wang, Yuan; Tschannen, Michael R.; Worthey, Elizabeth; Jacob, Howard; See, William; Kilari, Deepak; Wang, Xuexia; Hovey, Raymond L.; Huang, Chiang-Ching; Wang, Liang	ONCOTARGET	2015	6	18	27	4,5
Precise quantitation of 136 urinary proteins by LC/MRM-MS using stable isotope labeled peptides as internal standards for biomarker discovery and/or verification studies	Percy, Andrew J.; Yang, Juncong; Hardie, Darryl B.; Chambers, Andrew G.; Tamura-Wells, Jessica; Borchers, Christoph H.	METHODS	2015	81		27	4,5
Discovery and characterization of long intergenic non-coding RNAs (lincRNA) module biomarkers in prostate cancer: an integrative analysis of RNA-Seq data	Cui, Weirong; Qian, Yulan; Zhou, Xiaoke; Lin, Yuxin; Jiang, Junfeng; Chen, Jiajia; Zhao, Zhongming; Shen, Bairong	BMC GENOMICS	2015	16		27	4,5
Circulating Tumor DNA Abundance and Potential Utility in De Novo Metastatic Prostate Cancer	Vandekerkhove, Gillian; Struss, Werner J.; Annala, Matti; Kallio, Heini M. L.; Khalaf, Daniel; Warner, Evan W.; Herberts, Cameron; Ritch, Elie; Beja, Kevin; Loktionova, Yulia; Hurtado-Coll, Antonio; Fazli, Ladan; So, Alan; Black, Peter C.; Nykter, Matti; Tammela, Teuvo; Chi, Kim N.; Gleave, Martin E.; Wyatt, Alexander W.	EUROPEAN UROLOGY	2019	75	4	26	13

TP53 Outperforms Other Androgen Receptor Biomarkers to Predict Abiraterone or Enzalutamide Outcome in Metastatic Castration-Resistant Prostate Cancer	De Laere, Bram; Oeyen, Steffi; Mayrhofer, Markus; Whitington, Tom; van Dam, Pieter-Jan; Van Oyen, Peter; Ghysel, Christophe; Ampe, Jozef; Ost, Piet; Demey, Wim; Hoekx, Lucien; Schrijvers, Dirk; Brouwers, Barbara; Lybaert, Willem; Everaert, Els G.; De Maeseneer, Daan; Strijbos, Michiel; Bols, Alain; Fransis, Karen; Beije, Nick; de Kruijff, Inge E.; van Dam, Valerie; Brouwer, Anja; Goossens, Dirk; Heyrman, Lien; Van den Eynden, Gert G.; Rutten, Annemie; Del Favero, Jurgen; Rantalainen, Mattias; Rajan, Prabhakar; Sleijfer, Stefan; Ullen, Anders; Yachnin, Jeffrey; Gronberg, Henrik; Van Laere, Steven J.; Lindberg, Johan; Dirix, Luc Y.	CLINICAL CANCER RESEARCH	2019	25	6	26	13
Cell metabolomics identify regulatory pathways and targets of magnoline against prostate cancer	Sun, Hui; Zhang, Ai-hua; Liu, Shao-bo; Qiu, Shi; Li, Xian-na; Zhang, Tian-lei; Liu, Liang; Wang, Xi-jun	JOURNAL OF CHROMATOGRAPHY B-ANALYTICAL TECHNOLOGIES IN THE BIOMEDICAL AND LIFE SCIENCES	2018	1102		26	8,67
Aberrant RNA Splicing in Cancer and Drug Resistance	Wang, Bi-Dar; Lee, Norman H.	CANCERS	2018	10	11	26	8,67
Clinical and Genomic Characterization of Low-Prostate-specific Antigen, High-grade Prostate Cancer	Mahal, Brandon A.; Yang, David D.; Wang, Natalie Q.; Alshalalfa, Mohammed; Davicioni, Elai; Choeurng, Voleak; Schaeffer, Edward M.; Ross, Ashley E.; Spratt, Daniel E.; Den, Robert B.; Martin, Neil E.; Mouw, Kent W.; Orio, Peter F., III; Choueiri, Toni K.; Taplin, Mary-Ellen; Quoc-Dien Trinh; Feng, Felix Y.; Nguyen, Paul L.	EUROPEAN UROLOGY	2018	74	2	26	8,67

Enduring epigenetic landmarks define the cancer microenvironment	Pidsley, Ruth; Lawrence, Mitchell G.; Zotenko, Elena; Niranjan, Birunthi; Statham, Aaron; Song, Jenny; Chabanon, Roman M.; Qu, Wenjia; Wang, Hong; Richards, Michelle; Nair, Shalima S.; Armstrong, Nicola J.; Nim, Hieu T.; Papargiris, Melissa; Balanathan, Preetika; French, Hugh; Peters, Timothy; Norden, Sam; Ryan, Andrew; Pedersen, John; Kench, James; Daly, Roger J.; Horvath, Lisa G.; Stricker, Phillip; Frydenberg, Mark; Taylor, Renea A.; Stirzaker, Clare; Risbridger, Gail P.; Clark, Susan J.	GENOME RESEARCH	2018	28	5	26	8,67
A RNA-Sequencing approach for the identification of novel long non-coding RNA biomarkers in colorectal cancer	Yamada, Atsushi; Yu, Pingjian; Lin, Wei; Okugawa, Yoshinaga; Boland, C. Richard; Goel, Ajay	SCIENTIFIC REPORTS	2018	8		26	8,67
Prognostic and predictive biomarkers in prostate cancer: latest evidence and clinical implications	Terada, Naoki; Akamatsu, Shusuke; Kobayashi, Takashi; Inoue, Takahiro; Ogawa, Osamu; Antonarakis, Emmanuel S.	THERAPEUTIC ADVANCES IN MEDICAL ONCOLOGY	2017	9	8	26	6,5
Development and Validation of a Three-gene Prognostic Signature for Patients with Hepatocellular Carcinoma	Li, Binghua; Feng, Wendu; Luo, Ouyang; Xu, Tiancheng; Cao, Yajuan; Wu, Hongyan; Yu, Decai; Ding, Yitao	SCIENTIFIC REPORTS	2017	7		26	6,5
The ovarian cancer oncobiome	Banerjee, Sagarika; Tian, Tian; Wei, Zhi; Shih, Natalie; Feldman, Michael D.; Alwine, James C.; Coukos, George; Robertson, Erle S.	ONCOTARGET	2017	8	22	26	6,5
Extracellular vesicles as a source for non-invasive biomarkers in bladder cancer progression	Andreu, Zoraida; Otta Oshiro, Renan; Redruello, Alberto; Lopez-Martin, Soraya; Gutierrez-Vazquez, Cristina; Morato, Esperanza; Isabel Marina, Ana; Olivier Gomez, Carlos; Yanez-Mo, Maria	EUROPEAN JOURNAL OF PHARMACEUTICAL SCIENCES	2017	98		26	6,5

Sequential pathogenesis of metastatic VHL mutant clear cell renal cell carcinoma: putting it together with a translational perspective	Shenoy, N.; Pagliaro, L.	ANNALS OF ONCOLOGY	2016	27	9	26	5,2
Evaluation and consequences of heterogeneity in the circulating tumor cell compartment	Brouwer, Anja; De laere, Bram; Peeters, Dieter; Peeters, Marc; Salgado, Roberto; Dirix, Luc; Van Laere, Steven	ONCOTARGET	2016	7	30	26	5,2
BET bromodomain-mediated interaction between ERG and BRD4 promotes prostate cancer cell invasion	Blee, Alexandra M.; Liu, Shujun; Wang, Liguo; Huang, Haojie	ONCOTARGET	2016	7	25	26	5,2

Integration of multiethnic fine-mapping and genomic annotation to prioritize candidate functional SNPs at prostate cancer susceptibility regions	Han, Ying; Hazelett, Dennis J.; Wiklund, Fredrik; Schumacher, Fredrick R.; Stram, Daniel O.; Berndt, Sonja I.; Wang, Zhaoming; Rand, Kristin A.; Hoover, Robert N.; Machiela, Mitchell J.; Yeager, Merideth; Burdette, Laurie; Chung, Charles C.; Hutchinson, Amy; Yu, Kai; Xu, Jianfeng; Travis, Ruth C.; Key, Timothy J.; Siddiq, Afshan; Canzian, Federico; Takahashi, Atsushi; Kubo, Michiaki; Stanford, Janet L.; Kolb, Suzanne; Gapstur, Susan M.; Diver, W. Ryan; Stevens, Victoria L.; Strom, Sara S.; Pettaway, Curtis A.; Al Olama, Ali Amin; Kote-Jarai, Zsofia; Eeles, Rosalind A.; Yeboah, Edward D.; Tettey, Yao; Biritwum, Richard B.; Adjei, Andrew A.; Tay, Evelyn; Truelove, Ann; Niwa, Shelley; Chokkalingam, Anand P.; Isaacs, William B.; Chen, Constance; Lindstrom, Sara; Le Marchand, Loic; Giovannucci, Edward L.; Pomerantz, Mark; Long, Henry; Li, Fugen; Ma, Jing; Stampfer, Meir; John, Esther M.; Ingles, Sue A.; Kittles, Rick A.; Murphy, Adam B.; Blot, William J.; Signorello, Lisa B.; Zheng, Wei; Albanes, Demetrius; Virtamo, Jarmo; Weinstein, Stephanie; Nemesure, Barbara; Carpten, John; Leske, M. Cristina; Wu, Suh-Yuh; Hennis, Anselm J. M.; Rybicki, Benjamin A.; Neslund-Dudas, Christine; Hsing, Ann W.; Chu, Lisa; Goodman, Phyllis J.; Klein, Eric A.; Zheng, S. Lilly; Witte, John S.; Casey, Graham;	HUMAN MOLECULAR GENETICS	2015	24	19	26	4,33
An integrated genome-wide approach to discover deregulated microRNAs in non-small cell lung cancer: Clinical significance of miR-23b-3p deregulation	Begum, Shahnaz; Hayashi, Masamichi; Ogawa, Takenori; Jabboure, Fayez J.; Brait, Mariana; Izumchenko, Evgeny; Tabak, Sarit; Ahrendt, Steven A.; Westra, William H.; Koch, Wayne; Sidransky, David; Hoque, Mohammad O.	SCIENTIFIC REPORTS	2015	5		26	4,33

Androgen receptor non-nuclear regulation of prostate cancer cell invasion mediated by Src and matriptase	Zarif, Jelani C.; Lamb, Laura E.; Schulz, Veronique V.; Nollet, Eric A.; Miranti, Cindy K.	ONCOTARGET	2015	6	9	26	4,33
The histone chaperone HJURP is a new independent prognostic marker for luminal A breast carcinoma	de Oca, Rocio Montes; Gurard-Levin, Zachary A.; Berger, Frederique; Rehman, Haniya; Martel, Elise; Corpet, Armelle; de Koning, Leanne; Vassias, Isabelle; Wilson, Laurence O. W.; Meseure, Didier; Reyal, Fabien; Savignoni, Alexia; Asselain, Bernard; Sastre-Garauk, Xavier; Almouzni, Genevieve	MOLECULAR ONCOLOGY	2015	9	3	26	4,33
Cracking the Code of Human Diseases Using Next-Generation Sequencing: Applications, Challenges, and Perspectives	Precone, Vincenza; Del Monaco, Valentina; Esposito, Maria Valeria; De Palma, Fatima Domenica Elisa; Ruocco, Anna; Salvatore, Francesco; D'Argenio, Valeria	BIOMED RESEARCH INTERNATIONAL	2015	2015		26	4,33
A Phase II Trial of the Aurora Kinase A Inhibitor Alisertib for Patients with Castration-resistant and Neuroendocrine Prostate Cancer: Efficacy and Biomarkers	Beltran, Himisha; Oromendia, Clara; Danila, Daniel C.; Montgomery, Bruce; Hoimes, Christopher; Szmulewitz, Russell Z.; Vaishampayan, Ulka; Armstrong, Andrew J.; Stein, Mark; Pinski, Jacek; Mosquera, Juan M.; Sailer, Verena; Bareja, Rohan; Romanel, Alessandro; Gumpeni, Naveen; Sboner, Andrea; Dardenne, Etienne; Puca, Loredana; Prandi, Davide; Rubin, Mark A.; Scher, Howard I.; Rickman, David S.; Demichelis, Francesca; Nanus, David M.; Ballman, Karla V.; Tagawa, Scott T.	CLINICAL CANCER RESEARCH	2019	25	1	25	12,5

Molecular Evolution of Early-Onset Prostate Cancer Identifies Molecular Risk Markers and Clinical Trajectories	Gerhauser, Clarissa; Favero, Francesco; Risch, Thomas; Simon, Ronald; Feuerbach, Lars; Assenov, Yassen; Heckmann, Doreen; Sidiropoulos, Nikos; Waszak, Sebastian M.; Huebschmann, Daniel; Urbanucci, Alfonso; Girma, Etsehiwot G.; Kuryshev, Vladimir; Klimczak, Leszek J.; Saini, Natalie; Stuetz, Adrian M.; Weichenhan, Dieter; Boettcher, Lisa-Marie; Toth, Reka; Hendriksen, Josephine D.; Koop, Christina; Lutsik, Pavlo; Matzk, Soeren; Warnatz, Hans-Joerg; Amstislayskiy, Vyacheslav; Feuerstein, Clarissa; Raeder, Benjamin; Bogatyrova, Olga; Schmitz, Eva-Maria; Hube-Magg, Claudia; Kluth, Martina; Huland, Hartwig; Graefen, Markus; Lawerenz, Chris; Henry, Gervaise H.; Yamaguchi, Takafumi N.; Malewska, Alicia; Meiners, Jan; Schilling, Daniela; Reisinger, Eva; Eils, Roland; Schlesner, Matthias; Strand, Douglas W.; Bristow, Robert G.; Boutros, Paul C.; von Kalle, Christof; Gordenin, Dmitry; Sueltmann, Holger; Brors, Benedikt; Sauter, Guido; Plass, Christoph; Yaspo, Marie-Laure; Korbel, Jan O.; Schlomm, Thorsten; Weischenfeldt, Joachim	CANCER CELL	2018	34	6	25	8,33
A PDX/Organoid Biobank of Advanced Prostate Cancers Captures Genomic and Phenotypic Heterogeneity for Disease Modeling and Therapeutic Screening	Beshiri, Michael L.; Tice, Caitlin M.; Tran, Crystal; Nguyen, Holly M.; Sowalsky, Adam G.; Agarwal, Supreet; Jansson, Keith H.; Yang, Qi; McGowen, Kerry M.; Yin, JuanJuan; Alilin, Aian Neil; Karzai, Fatima H.; Dahut, William L.; Corey, Eva; Kelly, Kathleen	CLINICAL CANCER RESEARCH	2018	24	17	25	8,33

Diverse AR-V7 cistromes in castration-resistant prostate cancer are governed by HoxB13	Chen, Zhong; Wu, Dayong; Thomas-Ahner, Jennifer M.; Lu, Changxue; Zhao, Pei; Zhang, Qingfu; Geraghty, Connor; Yan, Pearlly S.; Hankey, William; Sunkel, Benjamin; Cheng, Xiaolong; Antonarakis, Emmanuel S.; Wang, Qi-En; Liu, Zhihua; Huang, Tim H-M; Jin, Victor X.; Clinton, Steven K.; Luo, Jun; Huang, Jiaoti; Wang, Qianben	PROCEEDINGS OF THE NATIONAL ACADEMY OF SCIENCES OF THE UNITED STATES OF AMERICA	2018	115	26	25	8,33
Treatment of Advanced Prostate Cancer-A Review of Current Therapies and Future Promise	Sumanasuriya, Semini; De Bono, Johann	COLD SPRING HARBOR PERSPECTIVES IN MEDICINE	2018	8	6	25	8,33
GC-MS based metabolomics used for the identification of cancer volatile organic compounds as biomarkers	Lubes, Giuseppe; Goodarzi, Mohammad	JOURNAL OF PHARMACEUTICAL AND BIOMEDICAL ANALYSIS	2018	147		25	8,33
Intratumoural evolutionary landscape of high-risk prostate cancer: the PROGENY study of genomic and immune parameters	Linch, M.; Goh, G.; Hiley, C.; Shanmugabavan, Y.; McGranahan, N.; Rowan, A.; Wong, Y. N. S.; King, H.; Furness, A.; Freeman, A.; Linares, J.; Akarca, A.; Herrero, J.; Rosenthal, R.; Harder, N.; Schmidt, G.; Wilson, G. A.; Birkbak, N. J.; Mitter, R.; Dentro, S.; Cathcart, P.; Arya, M.; Johnston, E.; Scott, R.; Hung, M.; Emberton, M.; Attard, G.; Szallasi, Z.; Punwani, S.; Quezada, S. A.; Marafioti, T.; Gerlinger, M.; Ahmed, H. U.; Swanton, C.	ANNALS OF ONCOLOGY	2017	28	10	25	6,25
Cell Cycle Control by PTEN	Brandmaier, Andrew; Hou, Sheng-Qi; Shen, Wen H.	JOURNAL OF MOLECULAR BIOLOGY	2017	429	15	25	6,25

Genomic landscape of high-grade meningiomas	Bi, Wenya Linda; Greenwald, Noah F.; Abedalthagafi, Malak; Wala, Jeremiah; Gibson, Will J.; Agarwalla, Pankaj K.; Horowitz, Peleg; Schumacher, Steven E.; Esaulova, Ekaterina; Mei, Yu; Chevalier, Aaron; Ducar, Matthew A.; Thorner, Aaron R.; van Hummelen, Paul; Stemmer-Rachamimov, Anat O.; Artyomov, Maksym; Al-Mefty, Ossama; Dunn, Gavin P.; Santagata, Sandro; Dunn, Ian F.; Beroukhim, Rameen	NPJ GENOMIC MEDICINE	2017	2		25	6,25
Mitochondrial mutations and metabolic adaptation in pancreatic cancer	Hardie, Rae-Anne; van Dam, Ellen; Cowley, Mark; Han, Ting-Li; Balaban, Seher; Pajic, Marina; Pinese, Mark; Iconomou, Mary; Shearer, Robert F.; McKenna, Jessie; Miller, David; Waddell, Nicola; Pearson, John V.; Grimmond, Sean M.; Sazanov, Leonid; Biankin, Andrew V.; Villas-Boas, Silas; Hoy, Andrew J.; Turner, Nigel; Saunders, Darren N.	CANCER & METABOLISM	2017	5		25	6,25
Dissecting the Heterogeneity of Circulating Tumor Cells in Metastatic Breast Cancer: Going Far Beyond the Needle in the Haystack	Bulfoni, Michela; Turetta, Matteo; Del Ben, Fabio; Di Loreto, Carla; Beltrami, Antonio Paolo; Cesselli, Daniela	INTERNATIONAL JOURNAL OF MOLECULAR SCIENCES	2016	17	10	25	5
Systems Glycobiology: Integrating Glycogenomics, Glycoproteomics, Glycomics, and Other 'Omics Data Sets to Characterize Cellular Glycosylation Processes	Bennun, Sandra V.; Hizal, Deniz Baycin; Heffner, Kelley; Can, Ozge; Zhang, Hui; Betenbaugh, Michael J.	JOURNAL OF MOLECULAR BIOLOGY	2016	428	16	25	5
Copy number variations in urine cell free DNA as biomarkers in advanced prostate cancer	Xia, Yun; Huang, Chiang-Ching; Dittmar, Rachel; Du, Meijun; Wang, Yuan; Liu, Hongyan; Shenoy, Niraj; Wang, Liang; Kohli, Manish	ONCOTARGET	2016	7	24	25	5

Validation of a Genomic Classifier for Predicting Post-Prostatectomy Recurrence in a Community Based Health Care Setting	Glass, Andrew G.; Leo, Michael C.; Haddad, Zaid; Yousefi, Kasra; du Plessis, Marguerite; Chen, Chuhe; Choeurng, Voleak; Abdollah, Firas; Robbins, Bruce; Ra, Seong; Richert-Boe, Kathryn E.; Buerki, Christine; Pearson, Kathy; Davicioni, Elai; Weinmann, Sheila	JOURNAL OF UROLOGY	2016	195	6	25	5
Targeted proteomics identifies liquid-biopsy signatures for extracapsular prostate cancer	Kim, Yunee; Jeon, Jouhyun; Mejia, Salvador; Yao, Cindy Q.; Ignatchenko, Vladimir; Nyalwidhe, Julius O.; Gramolini, Anthony O.; Lance, Raymond S.; Troyer, Dean A.; Drake, Richard R.; Boutros, Paul C.; Semmes, O. John; Kislinger, Thomas	NATURE COMMUNICATION S	2016	7		25	5
Regulation of E3 ubiquitin ligase-1 (WWP1) by microRNA-452 inhibits cancer cell migration and invasion in prostate cancer	Goto, Yusuke; Kojima, Satoko; Kurozumi, Akira; Kato, Mayuko; Okato, Atsushi; Matsushita, Ryosuke; Ichikawa, Tomohiko; Seki, Naohiko	BRITISH JOURNAL OF CANCER	2016	114	10	25	5
The Childhood Solid Tumor Network: A new resource for the developmental biology and oncology research communities	Stewart, Elizabeth; Federico, Sara; Karlstrom, Asa; Shelat, Anang; Sablauer, Andras; Pappo, Alberto; Dyer, Michael A.	DEVELOPMENTAL BIOLOGY	2016	411	2	25	5
Distinct lymphocyte antigens 6 (Ly6) family members Ly6D, Ly6E, Ly6K and Ly6H drive tumorigenesis and clinical outcome	Luo, Linlin; McGarvey, Peter; Madhavan, Subha; Kumar, Rakesh; Gusev, Yuriy; Upadhyay, Geeta	ONCOTARGET	2016	7	10	25	5
Unscrambling the genomic chaos of osteosarcoma reveals extensive transcript fusion, recurrent rearrangements and frequent novel TP53 aberrations	Lorenz, Susanne; Baroy, Tale; Sun, Jinchang; Nome, Torfinn; Vodak, Daniel; Bryne, Jan-Christian; Hakelien, Anne-Mari; Fernandez-Cuesta, Lynnette; Moehlendick, Birte; Rieder, Harald; Szuhai, Karoly; Zaikova, Olga; Ahlquist, Terje C.; Thomassen, Gard O. S.; Skotheim, Rolf I.; Lothe, Ragnhild A.; Tarpey, Patrick S.; Campbell, Peter; Flanagan, Adrienne; Myklebost, Ola; Meza-Zepeda, Leonardo A.	ONCOTARGET	2016	7	5	25	5

New blood markers detection technology: A leap in the diagnosis of gastric cancer	Beeharry, Maneesh K.; Liu, Wen-Tao; Yan, Min; Zhu, Zheng-Gang	WORLD JOURNAL OF GASTROENTEROLOGY	2016	22	3	25	5
Integration of tissue metabolomics, transcriptomics and immunohistochemistry reveals ERG- and gleason score-specific metabolomic alterations in prostate cancer	Meller, Sebastian; Meyer, Hellmuth-A; Bethan, Bianca; Dietrich, Dimo; Maldonado, Sandra Gonzalez; Lein, Michael; Montani, Matteo; Reszka, Regina; Schatz, Philipp; Peter, Erik; Stephan, Carsten; Jung, Klaus; Kamlage, Beate; Kristiansen, Glen	ONCOTARGET	2016	7	2	25	5
Urine metabolic phenotypes analysis of extrahepatic cholangiocarcinoma disease using ultra-high performance liquid chromatography-mass spectrometry	Wang, Xinxin; Li, Jun; Zhang, Ai-Hua	RSC ADVANCES	2016	6	67	25	5
High-throughput metabolomics to identify metabolites to serve as diagnostic biomarkers of prostate cancer	Li, Yuanfeng; Qiu, Shi; Zhang, Ai Hua	ANALYTICAL METHODS	2016	8	16	25	5
MAPK/ERK signaling pathway-induced hyper-O-GlcNAcylation enhances cancer malignancy	Zhang, Xinling; Ma, Leina; Qi, Jieqiong; Shan, Hui; Yu, Wengong; Gu, Yuchao	MOLECULAR AND CELLULAR BIOCHEMISTRY	2015	410	1-2	25	4,17
Synergistic action of image-guided radiotherapy and androgen deprivation therapy	Locke, Jennifer A.; Dal Pra, Alan; Supiot, Stephane; Warde, Padraig; Bristow, Robert G.	NATURE REVIEWS UROLOGY	2015	12	4	25	4,17
Circulating Tumor Cell Analysis in Metastatic Triple-Negative Breast Cancers	Magbanua, Mark Jesus M.; Carey, Lisa A.; DeLuca, Amy; Hwang, Jimmy; Scott, Janet H.; Rimawi, Mothaffar F.; Mayer, Erica L.; Marcom, P. Kelly; Liu, Minetta C.; Esteva, Francisco J.; Park, John W.; Rugo, Hope S.	CLINICAL CANCER RESEARCH	2015	21	5	25	4,17
OTUB1 de-ubiquitinating enzyme promotes prostate cancer cell invasion in vitro and tumorigenesis in vivo	Iglesias-Gato, Diego; Chuan, Yin-Choy; Jiang, Ning; Svensson, Charlotte; Bao, Jing; Paul, Indranil; Egevad, Lars; Kessler, Benedikt M.; Wikstorm, Pernilla; Niu, Yuanjie; Flores-Morales, Amilcar	MOLECULAR CANCER	2015	14		25	4,17

Prevalence of Germline Variants in Prostate Cancer and Implications for Current Genetic Testing Guidelines	Nicolosi, Piper; Ledet, Elisa; Yang, Shan; Michalski, Scott; Freschi, Brandy; O'Leary, Erin; Esplin, Edward D.; Nussbaum, Robert L.; Sartor, Oliver	JAMA ONCOLOGY	2019	5	4	24	12
Polycomb- and Methylation-Independent Roles of EZH2 as a Transcription Activator	Kim, Jung; Lee, Yongik; Lu, Xiaodong; Song, Bing; Fong, Ka-Wing; Cao, Qi; Licht, Jonathan D.; Zhao, Jonathan C.; Yu, Jindan	CELL REPORTS	2018	25	10	24	8
Cancer stem cells: Regulation programs, immunological properties and immunotherapy	Zhang, Dingxiao; Tang, Dean G.; Rycaj, Kiera	SEMINARS IN CANCER BIOLOGY	2018	52		24	8
Mass Spectrometry-Based Chemical and Enzymatic Methods for Global Analysis of Protein Glycosylation	Xiao, Haopeng; Suttapitugsakul, Suttipong; Sun, Fangxu; Wu, Ronghu	ACCOUNTS OF CHEMICAL RESEARCH	2018	51	8	24	8
FAM35A associates with REV7 and modulates DNAdamage responses of normal and BRCA1-defective cells	Tomida, Junya; Takata, Kei-ichi; Bhetawal, Sarita; Person, Maria D.; Chao, Hsueh-Ping; Tang, Dean G.; Wood, Richard D.	EMBO JOURNAL	2018	37	12	24	8
Microsatellite instability in prostate cancer by PCR or next-generation sequencing	Hempelmann, Jennifer A.; Lockwood, Christina M.; Konnick, Eric Q.; Schweizer, Michael T.; Antonarakis, Emmanuel S.; Lotan, Tamara L.; Montgomery, Bruce; Nelson, Peter S.; Klemfuss, Nola; Salipante, Stephen J.; Pritchard, Colin C.	JOURNAL FOR IMMUNOTHERAPY OF CANCER	2018	6		24	8
AKR1C1 Activates STAT3 to Promote the Metastasis of Non-Small Cell Lung Cancer	Zhu, Hong; Chang, Lin-Lin; Yan, Fang-Jie; Hu, Yan; Zeng, Chen-Ming; Zhou, Tian-Yi; Yuan, Tao; Ying, Mei-Dan; Cao, Ji; He, Qiao-Jun; Yang, Bo	THERANOSTICS	2018	8	3	24	8
Urinary biomarkers in prostate cancer detection and monitoring progression	Wu, Duojia; Ni, Jie; Beretov, Julia; Cozzi, Paul; Willcox, Mark; Wasinger, Valerie; Walsh, Bradley; Graham, Peter; Li, Yong	CRITICAL REVIEWS IN ONCOLOGY HEMATOLOGY	2017	118		24	6
Telomeres and telomerase in prostate cancer development and therapy	Graham, Mindy Kim; Meeker, Alan	NATURE REVIEWS UROLOGY	2017	14	10	24	6

Tunicamycin induced endoplasmic reticulum stress promotes apoptosis of prostate cancer cells by activating mTORC1	Guha, Prasun; Kaptan, Engin; Gade, Padmaja; Kalvakolanu, Dhananjaya V.; Ahmed, Hafiz	ONCOTARGET	2017	8	40	24	6
Decipher Test Impacts Decision Making Among Patients Considering Adjuvant and Salvage Treatment After Radical Prostatectomy: Interim Results From the Multicenter Prospective PRO-IMPACT Study	Gore, John L.; du Plessis, Marguerite G.; Santiago-Jimenez, Maria K.; Yousefi, Kasra K.; Thompson, Darby K.; Karsh, Lawrence P.; Lane, Brian R.; Franks, Michael T.; Chen, David S.; Bandyk, Mark P.; Bianco, Fernando J.; Brown, Gordon T.; Clark, William R.; Kibel, Adam S.; Kim, Hyung A.; Lowrance, William M.; Manoharan, Murugesan T.; Maroni, Paul U.; Perrapato, Scott, V; Sieber, Paul R.; Trabulsi, Edouard J.; Waterhouse, Robert C.; Davicioni, Elai T.; Lotan, Yair A.; Lin, Daniel W.	CANCER	2017	123	15	24	6
Autoantibody biomarkers for the detection of serous ovarian cancer	Katchman, Benjamin A.; Chowell, Diego; Wallstrom, Garrick; Vitonis, Allison F.; LaBaer, Joshua; Cramer, Daniel W.; Anderson, Karen S.	GYNECOLOGIC ONCOLOGY	2017	146	1	24	6
Quantitative proteomics by SWATH-MS reveals sophisticated metabolic reprogramming in hepatocellular carcinoma tissues	Gao, Yanyan; Wang, Xinzheng; Sang, Zhihong; Li, Zongcheng; Liu, Feng; Mao, Jie; Yan, Dan; Zhao, Yongqiang; Wang, Hongli; Li, Ping; Ying, Xiaomin; Zhang, Xuemin; He, Kun; Wang, Hongxia	SCIENTIFIC REPORTS	2017	7		24	6
Pan-Cancer Analysis of the Mediator Complex Transcriptome Identifies CDK19 and CDK8 as Therapeutic Targets in Advanced Prostate Cancer	Braegelmann, Johannes; Kluemper, Niklas; Offermann, Anne; Von Maessenhausen, Anne; Boehm, Diana; Deng, Mario; Queisser, Angela; Sanders, Christine; Syring, Isabella; Merseburger, Axel S.; Vogel, Wenzel; Sievers, Elisabeth; Vlasic, Ignacija; Carlsson, Jessica; Andren, Ove; Brossart, Peter; Duensing, Stefan; Svensson, Maria A.; Shaikhibrahim, Zaki; Kirfel, Jutta; Perner, Sven	CLINICAL CANCER RESEARCH	2017	23	7	24	6

Non-Genomic Actions of the Androgen Receptor in Prostate Cancer	Leung, Jacky K.; Sadar, Marianne D.	FRONTIERS IN ENDOCRINOLOGY	2017	8		24	6
Amino Acid Profiles of Serum and Urine in Search for Prostate Cancer Biomarkers: a Pilot Study	Derezinski, Pawel; Klupczynska, Agnieszka; Sawicki, Wojciech; Palka, Jerzy A.; Kokot, Zenon J.	INTERNATIONAL JOURNAL OF MEDICAL SCIENCES	2017	14	1	24	6
Antibody-drug conjugate targeting CD46 eliminates multiple myeloma cells	Sherbenou, Daniel W.; Aftab, Blake T.; Su, Yang; Behrens, Christopher R.; Wiita, Arun; Logan, Aaron C.; Acosta-Alvear, Diego; Hann, Byron C.; Walter, Peter; Shuman, Marc A.; Wu, Xiaobo; Atkinson, John P.; Wolf, Jeffrey L.; Martin, Thomas G.; Liu, Bin	JOURNAL OF CLINICAL INVESTIGATION	2016	126	12	24	4,8
OCT-4: a novel estrogen receptor-alpha collaborator that promotes tamoxifen resistance in breast cancer cells	Bhatt, S.; Stender, J. D.; Joshi, S.; Wu, G.; Katzenellenbogen, B. S.	ONCOGENE	2016	35	44	24	4,8
Effects of 8-Year Treatment of Long-Acting Testosterone Undecanoate on Metabolic Parameters, Urinary Symptoms, Bone Mineral Density, and Sexual Function in Men With Late-Onset Hypogonadism	Permpongkosol, Sompol; Khupulsup, Kalayanee; Leelaphiwat, Supatra; Pavavattananusorn, Sarawan; Thongpradit, Supranee; Petchthong, Thanom	JOURNAL OF SEXUAL MEDICINE	2016	13	8	24	4,8
Protein Counting in Single Cancer Cells	Schubert, Stephanie M.; Walter, Stephanie R.; Manesse, Mael; Walt, David R.	ANALYTICAL CHEMISTRY	2016	88	5	24	4,8
Differentially methylated genes and androgen receptor re-expression in small cell prostate carcinomas	Kleb, Brittany; Estecio, Marcos R. H.; Zhang, Jiexin; Tzelepid, Vassiliki; Chung, Woonbok; Jelinek, Jaroslav; Navone, Nora M.; Tahir, Salahaldin; Marquez, Victor E.; Issa, Jean-Pierre; Maity, Sankar; Aparicio, Ana	EPIGENETICS	2016	11	3	24	4,8
Interplay among Drosophila transcription factors Ets21c, Fos and Ftz-F1 drives JNK-mediated tumor malignancy	Kuelshammer, Eva; Mundorf, Juliane; Kilinc, Merve; Frommolt, Peter; Wagle, Prerana; Uhlirova, Mirka	DISEASE MODELS & MECHANISMS	2015	8	10	24	4

Two new loci and gene sets related to sex determination and cancer progression are associated with susceptibility to testicular germ cell tumor	Kristiansen, Wenche; Karlsson, Robert; Rounge, Trine B.; Whitington, Thomas; Andreassen, Bettina K.; Magnusson, Patrik K.; Fossa, Sophie D.; Adami, Hans-Olov; Turnbull, Clare; Haugen, Trine B.; Grotmol, Tom; Wiklund, Fredrik	HUMAN MOLECULAR GENETICS	2015	24	14	24	4
Colorimetric detection of both total genomic and loci-specific DNA methylation from limited DNA inputs	Wee, Eugene J. H.; Ngo, Thu Ha; Trau, Matt	CLINICAL EPIGENETICS	2015	7		24	4
Effect of a genomic classifier test on clinical practice decisions for patients with high-risk prostate cancer after surgery	Badani, Ketan K.; Thompson, Darby J.; Brown, Gordon; Holmes, Daniel; Kella, Naveen; Albala, David; Singh, Amar; Buerki, Christine; Davicioni, Elai; Hornberger, John	BJU INTERNATIONAL	2015	115	3	24	4
Molecular Foundations for Personalized Therapy in Prostate Cancer	Fisher, Kurt W.; Montironi, Rodolfo; Lopez Beltran, Antonio; Moch, Holger; Wang, Lisha; Scarpelli, Marina; Williamson, Sean R.; Koch, Michael O.; Cheng, Liang	CURRENT DRUG TARGETS	2015	16	2	24	4
ARv7 Represses Tumor-Suppressor Genes in Castration-Resistant Prostate Cancer	Cato, Laura; de Tribolet-Hardy, Jonas; Lee, Irene; Rottenberg, Jaice T.; Coleman, Ilsa; Melchers, Diana; Houtman, Rene; Xiao, Tengfei; Li, Wei; Uo, Takuma; Sun, Shihua; Kuznik, Nane C.; Goeppert, Bettina; Ozgun, Fatma; van Royen, Martin E.; Houtsmuller, Adriaan B.; Vadhi, Raga; Rao, Prakash K.; Li, Lewyn; Balk, Steven P.; Den, Robert B.; Trock, Bruce J.; Karnes, R. Jeffrey; Jenkins, Robert B.; Klein, Eric A.; Davicioni, Elai; Gruhl, Friederike J.; Long, Henry W.; Liu, X. Shirley; Cato, Andrew C. B.; Lack, Nathan A.; Nelson, Peter S.; Plymate, Stephen R.; Groner, Anna C.; Brown, Myles	CANCER CELL	2019	35	3	23	11,5

Autoantibody biomarkers for the detection of serous ovarian cancer	Katchman, Benjamin A.; Chowell, Diego; Wallstrom, Garrick; Vitonis, Allison F.; LaBaer, Joshua; Cramer, Daniel W.; Anderson, Karen S.	GYNECOLOGIC ONCOLOGY	2017	146	1	24	6
Database-augmented Mass Spectrometry Analysis of Exosomes Identifies Claudin 3 as a Putative Prostate Cancer Biomarker	Worst, Thomas Stefan; von Hardenberg, Jost; Gross, Julia Christina; Erben, Philipp; Schnoelzer, Martina; Hausser, Ingrid; Bugert, Peter; Michel, Maurice Stephan; Boutros, Michael	MOLECULAR & CELLULAR PROTEOMICS	2017	16	6	24	6
Quantitative proteomics by SWATH-MS reveals sophisticated metabolic reprogramming in hepatocellular carcinoma tissues	Gao, Yanyan; Wang, Xinzheng; Sang, Zhihong; Li, Zongcheng; Liu, Feng; Mao, Jie; Yan, Dan; Zhao, Yongqiang; Wang, Hongli; Li, Ping; Ying, Xiaomin; Zhang, Xuemin; He, Kun; Wang, Hongxia	SCIENTIFIC REPORTS	2017	7		24	6
Cancer metastasis: issues and 'challenges	Qian, Chao-Nan; Mei, Yan; Zhang, Jian	CHINESE JOURNAL OF CANCER	2017	36		24	6
Pan-Cancer Analysis of the Mediator Complex Transcriptome Identifies CDK19 and CDK8 as Therapeutic Targets in Advanced Prostate Cancer	Braegelmann, Johannes; Kluemper, Niklas; Offermann, Anne; Von Maessenhausen, Anne; Boehm, Diana; Deng, Mario; Queisser, Angela; Sanders, Christine; Syring, Isabella; Merseburger, Axel S.; Vogel, Wenzel; Sievers, Elisabeth; Vlasic, Ignacija; Carlsson, Jessica; Andren, Ove; Brossart, Peter; Duensing, Stefan; Svensson, Maria A.; Shaikhibrahim, Zaki; Kirfel, Jutta; Perner, Sven	CLINICAL CANCER RESEARCH	2017	23	7	24	6
Amino Acid Profiles of Serum and Urine in Search for Prostate Cancer Biomarkers: a Pilot Study	Derezinski, Pawel; Klupczynska, Agnieszka; Sawicki, Wojciech; Palka, Jerzy A.; Kokot, Zenon J.	INTERNATIONAL JOURNAL OF MEDICAL SCIENCES	2017	14	1	24	6
OCT-4: a novel estrogen receptor-alpha collaborator that promotes tamoxifen resistance in breast cancer cells	Bhatt, S.; Stender, J. D.; Joshi, S.; Wu, G.; Katzenellenbogen, B. S.	ONCOGENE	2016	35	44	24	4,8
Sequencing Structural Variants in Cancer for Precision Therapeutics	Macintyre, Geoff; Ylstra, Bauke; Brenton, James D.	TRENDS IN GENETICS	2016	32	9	24	4,8

Effects of 8-Year Treatment of Long-Acting Testosterone Undecanoate on Metabolic Parameters, Urinary Symptoms, Bone Mineral Density, and Sexual Function in Men With Late-Onset Hypogonadism	Permpongkosol, Sompol; Khupulsup, Kalayanee; Leelaphiwat, Supatra; Pavavattananusorn, Sarawan; Thongpradit, Supranee; Petchthong, Thanom	JOURNAL OF SEXUAL MEDICINE	2016	13	8	24	4,8
CAPE suppresses migration and invasion of prostate cancer cells via activation of non-canonical Wnt signaling	Tseng, Jen-Chih; Lin, Ching-Yu; Su, Liang-Chen; Fu, Hsiao-Hui; Yang, Shiaw-Der; Chuu, Chih-Pin	ONCOTARGET	2016	7	25	24	4,8
Protein Counting in Single Cancer Cells	Schubert, Stephanie M.; Walter, Stephanie R.; Manesse, Mael; Walt, David R.	ANALYTICAL CHEMISTRY	2016	88	5	24	4,8
Tissue Metabonomic Phenotyping for Diagnosis and Prognosis of Human Colorectal Cancer	Tian, Yuan; Xu, Tangpeng; Huang, Jia; Zhang, Limin; Xu, Shan; Xiong, Bin; Wang, Yulan; Tang, Huiru	SCIENTIFIC REPORTS	2016	6		24	4,8
Differentially methylated genes and androgen receptor re-expression in small cell prostate carcinomas	Kleb, Brittany; Estecio, Marcos R. H.; Zhang, Jiexin; Tzelepid, Vassiliki; Chung, Woonbok; Jelinek, Jaroslav; Navone, Nora M.; Tahir, Salahaldin; Marquez, Victor E.; Issa, Jean-Pierre; Maity, Sankar; Aparicio, Ana	EPIGENETICS	2016	11	3	24	4,8
Two new loci and gene sets related to sex determination and cancer progression are associated with susceptibility to testicular germ cell tumor	Kristiansen, Wenche; Karlsson, Robert; Rounge, Trine B.; Whitington, Thomas; Andreassen, Bettina K.; Magnusson, Patrik K.; Fossa, Sophie D.; Adami, Hans-Olov; Turnbull, Clare; Haugen, Trine B.; Grotmol, Tom; Wiklund, Fredrik	HUMAN MOLECULAR GENETICS	2015	24	14	24	4
Colorimetric detection of both total genomic and loci-specific DNA methylation from limited DNA inputs	Wee, Eugene J. H.; Ngo, Thu Ha; Trau, Matt	CLINICAL EPIGENETICS	2015	7		24	4
Targeting EZH2 for Cancer Therapy: Progress and Perspective	Li, Chi Han; Chen, Yangchao	CURRENT PROTEIN & PEPTIDE SCIENCE	2015	16	6	24	4

Molecular Foundations for Personalized Therapy in Prostate Cancer	Fisher, Kurt W.; Montironi, Rodolfo; Lopez Beltran, Antonio; Moch, Holger; Wang, Lisha; Scarpelli, Marina; Williamson, Sean R.; Koch, Michael O.; Cheng, Liang	CURRENT DRUG TARGETS	2015	16	2	24	4
Cell-free DNA profiling of metastatic prostate cancer reveals microsatellite instability, structural rearrangements and clonal hematopoiesis	Mayrhofer, Markus; De Laere, Bram; Whitington, Tom; Van Oyen, Peter; Ghysel, Christophe; Ampe, Jozef; Ost, Piet; Demey, Wim; Hoekx, Lucien; Schrijvers, Dirk; Brouwers, Barbara; Lybaert, Willem; Everaert, Els; De Maeseneer, Daan; Strijbos, Michiel; Bols, Alain; Fransis, Karen; Oeyen, Steffi; van Dam, Pieter-Jan; Van den Eynden, Gert; Rutten, Annemie; Aly, Markus; Nordstrom, Tobias; Van Laere, Steven; Rantalainen, Mattias; Rajan, Prabhakar; Egevad, Lars; Ullen, Anders; Yachnin, Jeffrey; Dirix, Luc; Gronberg, Henrik; Lindberg, Johan	GENOME MEDICINE	2018	10		23	7,67
Integrated Pharmacodynamic Analysis Identifies Two Metabolic Adaption Pathways to Metformin in Breast Cancer	Lord, Simon R.; Cheng, Wei-Chen; Liu, Dan; Gaude, Edoardo; Haider, Syed; Metcalf, Tom; Patel, Neel; Teoh, Eugene J.; Gleeson, Fergus; Bradley, Kevin; Wigfield, Simon; Zois, Christos; McGowan, Daniel R.; Ah-See, Mei-Lin; Thompson, Alastair M.; Sharma, Anand; Bidaut, Luc; Pollak, Michael; Roy, Pankaj G.; Karpe, Fredrik; James, Tim; English, Ruth; Adams, Rosie F.; Campo, Leticia; Ayers, Lisa; Snell, Cameron; Roxanis, Ioannis; Frezza, Christian; Fenwick, John D.; Buffa, Francesca M.; Harris, Adrian L.	CELL METABOLISM	2018	28	5	23	7,67
LncRNA MIR100HG promotes cell proliferation in triple- negative breast cancer through triplex formation with p27 loci	Wang, Shaowei; Ke, Hao; Zhang, Honglei; Ma, Yujie; Ao, Lei; Zou, Li; Yang, Qin; Zhu, Hao; Nie, Jianyun; Wu, Chunlian; Jiao, Baowei	CELL DEATH & DISEASE	2018	9		23	7,67

Designer Oncolytic Adenovirus: Coming of Age	Baker, Alexander T.; Aguirre-Hernandez, Carmen; Hallden, Gunnel; Parker, Alan L.	CANCERS	2018	10	6	23	7,67
BRD4 Promotes DNA Repair and Mediates the Formation of TMPRSS2-ERG Gene Rearrangements in Prostate Cancer	Li, Xiangyi; Baek, GuemHee; Ramanand, Susmita G.; Sharp, Adam; Gao, Yunpeng; Yuan, Wei; Welti, Jon; Rodrigues, Daniel N.; Dolling, David; Figueiredo, Ines; Sumanasuriya, Semini; Crespo, Mateus; Aslam, Adam; Li, Rui; Yin, Yi; Mukherjee, Bipasha; Kanchwala, Mohammed; Hughes, Ashley M.; Halsey, Wendy S.; Chiang, Cheng-Ming; Xing, Chao; Raj, Ganesh V.; Burma, Sandeep; de Bono, Johann; Mani, Ram S.	CELL REPORTS	2018	22	3	23	7,67
CRAF gene fusions in pediatric low-grade gliomas define a distinct drug response based on dimerization profiles	Jain, P.; Fierst, T. M.; Han, H. J.; Smith, T. E.; Vakil, A.; Storm, P. B.; Resnick, A. C.; Waanders, A. J.	ONCOGENE	2017	36	45	23	5,75
Mitochondrial mutations drive prostate cancer aggression	Hopkins, Julia F.; Sabelnykova, Veronica Y.; Weischenfeldt, Joachim; Simon, Ronald; Aguiar, Jennifer A.; Alkallas, Rached; Heisler, Lawrence E.; Zhang, Junyan; Watson, John D.; Chua, Melvin L. K.; Fraser, Michael; Favero, Francesco; Lawerenz, Chris; Plass, Christoph; Sauter, Guido; McPherson, John D.; van der Kwast, Theodorus; Korbel, Jan; Schlomm, Thorsten; Bristow, Robert G.; Boutros, Paul C.	NATURE COMMUNICATION S	2017	8		23	5,75
PARP Inhibitors in Prostate Cancer	Geethakumari, Praveen Ramakrishnan; Schiewer, Matthew J.; Knudsen, Karen E.; Kelly, Wm. Kevin	CURRENT TREATMENT OPTIONS IN ONCOLOGY	2017	18	6	23	5,75
FEN1 promotes tumor progression and confers cisplatin resistance in non-small-cell lung cancer	He, Lingfeng; Luo, Libo; Zhu, Hong; Yang, Huan; Zhang, Yilan; Wu, Huan; Sun, Hongfang; Jiang, Feng; Kathera, Chandra S.; Liu, Lingjie; Zhuang, Ziheng; Chen, Haoyan; Pan, Feiyan; Hu, Zhigang; Zhang, Jing; Guo, Zhigang	MOLECULAR ONCOLOGY	2017	11	6	23	5,75

Global analysis of H3K27me3 as an epigenetic marker in prostate cancer progression	Ngollo, Marjolaine; Lebert, Andre; Daures, Marine; Judes, Gaelle; Rifai, Khaldoun; Dubois, Lucas; Kemeny, Jean-Louis; Penault-Llorca, Frederique; Bignon, Yves-Jean; Guy, Laurent; Bernard-Gallon, Dominique	BMC CANCER	2017	17		23	5,75
Resveratrol induces mitochondria-mediated, caspase-independent apoptosis in murine prostate cancer cells	Kumar, Sanjay; Eroglu, Erdal; Stokes, James A., III; Scissum-Gunn, Karyn; Saldanha, Sabita N.; Singh, Udai P.; Manne, Upender; Ponnazhagan, Selvarangan; Mishra, Manoj K.	ONCOTARGET	2017	8	13	23	5,75
Metabolomics Applications in Precision Medicine: An Oncological Perspective	Puchades-Carrasco, Leonor; Pineda-Lucena, Antonio	CURRENT TOPICS IN MEDICINAL CHEMISTRY	2017	17	24	23	5,75
Adding genetic risk score to family history identifies twice as many high-risk men for prostate cancer: Results from the prostate cancer prevention trial	Chen, Haitao; Liu, Xu; Brendler, Charles B.; Ankerst, Donna P.; Leach, Robin J.; Goodman, Phyllis J.; Lucia, M. Scott; Tangen, Catherine M.; Wang, Li; Hsu, Fang-Chi; Sun, Jielin; Kader, A. Karim; Isaacs, William B.; Helfand, Brian T.; Zheng, S. Lilly; Thompson, Ian M.; Platz, Elizabeth A.; Xu, Jianfeng	PROSTATE	2016	76	12	23	4,6
Quantification of Somatic Chromosomal Rearrangements in Circulating Cell-Free DNA from Ovarian Cancers	Harris, Faye R.; Kovtun, Irina V.; Smadbeck, James; Multinu, Francesco; Jatoi, Aminah; Kosari, Farhad; Kalli, Kimberly R.; Murphy, Stephen J.; Halling, Geoffrey C.; Johnson, Sarah H.; Liu, Minetta C.; Mariani, Andrea; Vasmatzis, George	SCIENTIFIC REPORTS	2016	6		23	4,6
Epigenomic profiling of DNA methylation in paired prostate cancer versus adjacent benign tissue	Geybels, Milan S.; Zhao, Shanshan; Wong, Chao-Jen; Bibikova, Marina; Klotzle, Brandy; Wu, Michael; Ostrander, Elaine A.; Fan, Jian-Bing; Feng, Ziding; Stanford, Janet L.	PROSTATE	2015	75	16	23	3,83
Nuclear Protein Sam68 Interacts with the Enterovirus 71 Internal Ribosome Entry Site and Positively Regulates Viral Protein Translation	Zhang, Hua; Song, Lei; Cong, Haolong; Tien, Po	JOURNAL OF VIROLOGY	2015	89	19	23	3,83

Role and regulation of coordinately expressed de novo purine biosynthetic enzymes PPAT and PAICS in lung cancer	Goswami, Moloy T.; Chen, Guoan; Chakravarthi, Balabhadrapatruni V. S. K.; Pathi, Satya S.; Anand, Sharath K.; Carskadon, Shannon L.; Giordano, Thomas J.; Chinnaiyan, Arul M.; Thomas, Dafydd G.; Palanisamy, Nallasivam; Beer, David G.; Varambally, Sooryanarayana	ONCOTARGET	2015	6	27	23	3,83
Systematic enrichment analysis of potentially functional regions for 103 prostate cancer risk-associated loci	Chen, Haitao; Yu, Hongjie; Wang, Jianqing; Zhang, Zheng; Gao, Zhengrong; Chen, Zhuo; Lu, Yulan; Liu, Wennuan; Jiang, Deke; Zheng, S. Lilly; Wei, Gong-hong; Issacs, William B.; Feng, Junjie; Xu, Jianfeng	PROSTATE	2015	75	12	23	3,83
Bone metastasis in prostate cancer: Recurring mitochondrial DNA mutation reveals selective pressure exerted by the bone microenvironment	Arnold, Rebecca S.; Fedewa, Stacey A.; Goodman, Michael; Osunkoya, Adeboye O.; Kissick, Haydn T.; Morrissey, Colm; True, Lawrence D.; Petros, John A.	BONE	2015	78		23	3,83
Breast cancer risk associated with gene expression and genotype polymorphisms of the folate-metabolizing MTHFR gene: a case-control study in a high altitude Ecuadorian mestizo population	Lopez-Cortes, Andres; Echeverria, Carolina; Ona-Cisneros, Fabian; Eugenia Sanchez, Maria; Herrera, Camilo; Cabrera-Andrade, Alejandro; Rosales, Felipe; Ortiz, Malena; Paz-y-Mino, Cesar	TUMOR BIOLOGY	2015	36	8	23	3,83
Capillary nano-immunoassays: advancing quantitative proteomics analysis, biomarker assessment, and molecular diagnostics	Chen, Jin-Qiu; Wakefield, Lalage M.; Goldstein, David J.	JOURNAL OF TRANSLATIONAL MEDICINE	2015	13		23	3,83
Functional proteomics of the epigenetic regulators ASXL1, ASXL2 and ASXL3: a convergence of proteomics and epigenetics for translational medicine	Katoh, Masaru	EXPERT REVIEW OF PROTEOMICS	2015	12	3	23	3,83

Proteomics of Microparticles with SILAC Quantification (PROMIS-Quan): A Novel Proteomic Method for Plasma Biomarker Quantification	Harel, Michal; Oren-Giladi, Pazit; Kaidar-Person, Orit; Shaked, Yuval; Geiger, Tamar	MOLECULAR & CELLULAR PROTEOMICS	2015	14	4	23	3,83
The G-quadruplex-stabilising agent RHPS4 induces telomeric dysfunction and enhances radiosensitivity in glioblastoma cells	Berardinelli, F.; Siteni, S.; Tanzarella, C.; Stevens, M. F.; Sgura, A.; Antoccia, A.	DNA REPAIR	2015	25		23	3,83
TrkB inhibition by GNF-4256 slows growth and enhances chemotherapeutic efficacy in neuroblastoma xenografts	Croucher, Jamie L.; Iyer, Radhika; Li, Nanxin; Molteni, Valentina; Loren, Jon; Gordon, W. Perry; Tuntland, Tove; Liu, Bo; Brodeur, Garrett M.	CANCER CHEMOTHERAPY AND PHARMACOLOGY	2015	75	1	23	3,83
Olaparib in patients with metastatic castration-resistant prostate cancer with DNA repair gene aberrations (TOPARP-B): a multicentre, open-label, randomised, phase 2 trial	Mateo, Joaquin; Porta, Nuria; Bianchini, Diletta; McGovern, Ursula; Elliott, Tony; Jones, Robert; Syndikus, Isabel; Ralph, Christy; Jain, Suneil; Varughese, Mohini; Parikh, Omi; Crabb, Simon; Robinson, Angus; McLaren, Duncan; Birtle, Alison; Tanguay, Jacob; Miranda, Susana; Figueiredo, Ines; Seed, George; Bertan, Claudia; Flohr, Penny; Ebbs, Berni; Rescigno, Pasquale; Fowler, Gemma; Ferreira, Ana; Riisnaes, Ruth; Pereira, Rita; Curcean, Andra; Chandler, Robert; Clarke, Matthew; Gurel, Bora; Crespo, Mateus; Rodrigues, Daniel Nava; Sandhu, Shahneen; Espinasse, Aude; Chatfield, Peter; Tunariu, Nina; Yuan, Wei; Hall, Emma; Carreira, Suzanne; de Bono, Johann S.	LANCET ONCOLOGY	2020	21	1	22	22

A precision oncology approach to the pharmacological targeting of mechanistic dependencies in neuroendocrine tumors	Alvarez, Mariano J.; Subramaniam, Prem S.; Tang, Laura H.; Grunn, Adina; Aburi, Mahalaxmi; Rieckhof, Gabrielle; Komissarova, Elena V.; Hagan, Elizabeth A.; Bodei, Lisa; Clemons, Paul A.; Dela Cruz, Filemon S.; Dhall, Deepti; Diolaiti, Daniel; Fraker, Douglas A.; Ghavami, Afshin; Kaemmerer, Daniel; Karan, Charles; Kidd, Mark; Kim, Kyoung M.; Kim, Hee C.; Kunju, Lakshmi P.; Langel, Ulo; Li, Zhong; Lee, Jeeyun; Li, Hai; LiVolsi, Virginia; Pfragner, Roswitha; Rainey, Allison R.; Realubit, Ronald B.; Remotti, Helen; Regberg, Jakob; Roses, Robert; Rustgi, Anil; Sepulveda, Antonia R.; Serra, Stefano; Shi, Chanjuan; Yuan, Xiaopu; Barberis, Massimo; Bergamaschi, Roberto; Chinnaiyan, Arul M.; Detre, Tony; Ezzat, Shereen; Frilling, Andrea; Hommann, Merten; Jaeger, Dirk; Kim, Michelle K.; Knudsen, Beatrice S.; Kung, Andrew L.; Leahy, Emer; Metz, David C.; Milsom, Jeffrey W.; Park, Young S.; Reidy-Lagunes, Diane; Schreiber, Stuart; Washington, Kay; Wiedenmann, Bertram; Modlin, Irvin; Califano, Andrea	NATURE GENETICS	2018	50	7	22	7,33
From biomarkers to therapeutic targets-the promises and perils of long non-coding RNAs in cancer	Gutschner, Tony; Richtig, Georg; Haemmerle, Monika; Pichler, Martin	CANCER AND METASTASIS REVIEWS	2018	37	1	22	7,33
Long noncoding RNA: multiple players in gene expression	Chen, Xiaochang; Sun, Yunmei; Cai, Rui; Wang, Guoqiang; Shu, Xiaoyan; Pang, Weijun	BMB REPORTS	2018	51	6	22	7,33
Smart Combinations of Bioactive Compounds in Fruits and Vegetables May Guide New Strategies for Personalized Prevention of Chronic Diseases	van Breda, Simone G. J.; de Kok, Theo M. C. M.	MOLECULAR NUTRITION & FOOD RESEARCH	2018	62	1	22	7,33

Comparative Transcriptome Profiling Reveals Coding and Noncoding RNA Differences in NSCLC from African Americans and European Americans	Mitchell, Khadijah A.; Zingone, Adriana; Toulabi, Leila; Boeckelman, Jacob; Ryan, Brid M.	CLINICAL CANCER RESEARCH	2017	23	23	22	5,5
MALDI imaging mass spectrometry - From bench to bedside	Schwamborn, Kristina; Kriegsmann, Mark; Weichert, Wilko	BIOCHIMICA ET BIOPHYSICA ACTA-PROTEINS AND PROTEOMICS	2017	1865	7	22	5,5
Translating a Prognostic DNA Genomic Classifier into the Clinic: Retrospective Validation in 563 Localized Prostate Tumors	Lalonde, Emilie; Alkallas, Rached; Chua, Melvin Lee Kiang; Fraser, Michael; Haider, Syed; Meng, Alice; Zheng, Junyan; Yao, Cindy Q.; Picard, Valerie; Orain, Michele; Hovington, Helene; Murgic, Jure; Berlin, Alejandro; Lacombe, Louis; Bergeron, Alain; Fradet, Yves; Tetu, Bernard; Lindberg, Johan; Egevad, Lars; Gronberg, Henrik; Ross-Adams, Helen; Lamb, Alastair D.; Halim, Silvia; Dunning, Mark J.; Neal, David E.; Pintilie, Melania; van der Kwast, Theodorus; Bristow, Robert G.; Boutros, Paul C.	EUROPEAN UROLOGY	2017	72	1	22	5,5
Streptomyces colonosanans sp nov., A Novel Actinobacterium Isolated Exhibiting Antioxidative Activity and Cytotoxic Potential against Human Colon Cancer Cell Lines	Law, Jodi Woan-Fei; Ser, Hooi-Leng; Duangjai, Acharaporn; Saokaew, Surasak; Bukhari, Sarah I.; Khan, Tahir M.; Ab Mutalib, Nurul-Syakima; Chan, Kok-Gan; Goh, Bey-Hing; Lee, Learn-Han	FRONTIERS IN MICROBIOLOGY	2017	8		22	5,5
Metabolomics approaches in pancreatic adenocarcinoma: tumor metabolism profiling predicts clinical outcome of patients	Battini, S.; Faitot, F.; Imperiale, A.; Cicek, A. E.; Heimburger, C.; Averous, G.; Bachellier, P.; Namer, I. J.	BMC MEDICINE	2017	15		22	5,5

Genome-wide association study of prostate-specific antigen levels identifies novel loci independent of prostate cancer	Hoffmann, Thomas J.; Passarelli, Michael N.; Graff, Rebecca E.; Emami, Nima C.; Sakoda, Lori C.; Jorgenson, Eric; Habel, Laurel A.; Shan, Jun; Ranatunga, Dilrini K.; Quesenberry, Charles P.; Chao, Chun R.; Ghai, Nirupa R.; Aaronson, David; Presti, Joseph; Nordstrom, Tobias; Wang, Zhaoming; Berndt, Sonja I.; Chanock, Stephen J.; Mosley, Jonathan D.; Klein, Robert J.; Middha, Mridu; Lilja, Hans; Melander, Olle; Kvale, Mark N.; Kwok, Pui-Yan; Schaefer, Catherine; Risch, Neil; Van Den Eeden, Stephen K.; Witte, John S.	NATURE COMMUNICATIONS	2017	8		22	5,5
SiNVICT: ultra-sensitive detection of single nucleotide variants and indels in circulating tumour DNA	Kockan, Can; Hach, Faraz; Sarrafi, Iman; Bell, Robert H.; McConeghy, Brian; Beja, Kevin; Haegert, Anne; Wyatt, Alexander W.; Volik, Stanislav V.; Chi, Kim N.; Collins, Colin C.; Sahinalp, Cenk	BIOINFORMATICS	2017	33	1	22	5,5
Phenotypic plasticity in prostate cancer: role of intrinsically disordered proteins	Mooney, Steven M.; Jolly, Mohit Kumar; Levine, Herbert; Kulkarni, Prakash	ASIAN JOURNAL OF ANDROLOGY	2016	18	5	22	4,4
Insulin-like Growth Factor 1 Signaling Axis Meets p53 Genome Protection Pathways	Werner, Haim; Sarfstein, Rive; LeRoith, Derek; Bruchim, Ilan	FRONTIERS IN ONCOLOGY	2016	6		22	4,4
Co-targeting hexokinase 2-mediated Warburg effect and ULK1-dependent autophagy suppresses tumor growth of PTEN- and TP53-deficiency-driven castration-resistant prostate cancer	Wang, Lei; Wang, Ji; Xiong, Hua; Wu, Fengxia; Lan, Tian; Zhang, Yingjie; Guo, Xiaolan; Wang, Huanan; Saleem, Mohammad; Jiang, Cheng; Lu, Junxuan; Deng, Yibin	EBIOMEDICINE	2016	7		22	4,4
Oncolytic vaccinia virus as a vector for therapeutic sodium iodide symporter gene therapy in prostate cancer	Mansfield, D. C.; Kyula, J. N.; Rosenfelder, N.; Chao-Chu, J.; Kramer-Marek, G.; Khan, A. A.; Roulstone, V.; McLaughlin, M.; Melcher, A. A.; Vile, R. G.; Pandha, H. S.; Khoo, V.; Harrington, K. J.	GENE THERAPY	2016	23	4	22	4,4

CDO1 promoter methylation is associated with gene silencing and is a prognostic biomarker for biochemical recurrence-free survival in prostate cancer patients	Meller, Sebastian; Zipfel, Lisa; Gevensleben, Heidrun; Dietrich, Joern; Ellinger, Joerg; Majores, Michael; Stein, Johannes; Sailer, Verena; Jung, Maria; Kristiansen, Glen; Dietrich, Dimo	EPIGENETICS	2016	11	12	22	4,4
The emerging role of the androgen receptor in bladder cancer	Lombard, Alan P.; Mudryj, Maria	ENDOCRINE-RELATED CANCER	2015	22	5	22	3,67
Epigenetic regulation of drug metabolism and transport	Peng, Lai; Zhong, Xiaobo	ACTA PHARMACEUTICA SINICA B	2015	5	2	22	3,67
Organoid development in cancer genome discovery	Gao, Dong; Chen, Yu	CURRENT OPINION IN GENETICS & DEVELOPMENT	2015	30		22	3,67
Targeting Transcription Factor Binding to DNA by Competing with DNA Binders as an Approach for Controlling Gene Expression	Bouhlel, Mohamed Amine; Lambert, Melanie; David-Cordonnier, Marie-Helene	CURRENT TOPICS IN MEDICINAL CHEMISTRY	2015	15	14	22	3,67
Diagnostic associations of gene expression signatures in prostate cancer tissue	Nguyen, Hao G.; Welty, Christopher J.; Cooperberg, Matthew R.	CURRENT OPINION IN UROLOGY	2015	25	1	22	3,67
Linking prostate cancer cell AR heterogeneity to distinct castration and enzalutamide responses	Li, Qiuhui; Deng, Qu; Chao, Hsueh-Ping; Liu, Xin; Lu, Yue; Lin, Kevin; Liu, Bigang; Tang, Gregory W.; Zhang, Dingxiao; Tracz, Amanda; Jeter, Collene; Rycaj, Kiera; Calhoun-Davis, Tammy; Huang, Jiaoti; Rubin, Mark A.; Beltran, Himisha; Shen, Jianjun; Chatta, Gurkamal; Puzanov, Igor; Mohler, James L.; Wang, Jianmin; Zhao, Ruizhe; Kirk, Jason; Chen, Xin; Tang, Dean G.	NATURE COMMUNICATIONS	2018	9		21	7

Systemic surfaceome profiling identifies target antigens for immune-based therapy in subtypes of advanced prostate cancer	Lee, John K.; Bangayan, Nathanael J.; Chai, Timothy; Smith, Bryan A.; Pariva, Tiffany E.; Yun, Sangwon; Vashisht, Ajay; Zhang, Qingfu; Park, Jung Wook; Corey, Eva; Huang, Jiaoti; Graeber, Thomas G.; Wohlschlegel, James; Witte, Owen N.	PROCEEDINGS OF THE NATIONAL ACADEMY OF SCIENCES OF THE UNITED STATES OF AMERICA	2018	115	19	21	7
Stromal Gene Expression is Predictive for Metastatic Primary Prostate Cancer	Mo, Fan; Lin, Dong; Takhar, Mandeep; Ramnarine, Varune Rohan; Dong, Xin; Bell, Robert H.; Volik, Stanislav V.; Wang, Kendric; Xue, Hui; Wang, Yuwei; Haegert, Anne; Anderson, Shawn; Brahmbhatt, Sonal; Erho, Nicholas; Wang, Xinya; Gout, Peter W.; Morris, James; Karnes, R. Jeffrey; Den, Robert B.; Klein, Eric A.; Schaeffer, Edward M.; Ross, Ashley; Ren, Shancheng; Sahinalp, S. Cenk; Li, Yingrui; Xu, Xun; Wang, Jun; Wang, Jian; Gleave, Martin E.; Davicioni, Elai; Sun, Yinghao; Wang, Yuzhuo; Collin, Colin C.	EUROPEAN UROLOGY	2018	73	4	21	7
Intraductal/ductal histology and lymphovascular invasion are associated with germline DNA-repair gene mutations in prostate cancer	Velho, Pedro Isaacsson; Silberstein, John L.; Markowski, Mark C.; Luo, Jun; Lotan, Tamara L.; Isaacs, William B.; Antonarakis, Emmanuel S.	PROSTATE	2018	78	5	21	7
Principal Component Analysis with Linear and Quadratic Discriminant Analysis for Identification of Cancer Samples Based on Mass Spectrometry	Morais, Camilo L. M.; Lima, Kassio M. G.	JOURNAL OF THE BRAZILIAN CHEMICAL SOCIETY	2018	29	3	21	7
Androgen receptor splice variants bind to constitutively open chromatin and promote abiraterone-resistant growth of prostate cancer	He, Yundong; Lu, Ji; Ye, Zhenqing; Hao, Siyuan; Wang, Liewei; Kohli, Manish; Tindall, Donald J.; Li, Benyi; Zhu, Runzhi; Wang, Liguo; Huang, Haojie	NUCLEIC ACIDS RESEARCH	2018	46	4	21	7

Single-Cell RNA-seq Reveals a Subpopulation of Prostate Cancer Cells with Enhanced Cell-Cycle-Related Transcription and Attenuated Androgen Response	Horning, Aaron M.; Wang, Yao; Lin, Che-Kuang; Louie, Anna D.; Jadhav, Rohit R.; Hung, Chia-Nung; Wang, Chiou-Miin; Lin, Chun-Lin; Kirma, Nameer B.; Liss, Michael A.; Kumar, Addanki P.; Sun, Luzhe; Liu, Zhijie; Chao, Wei-Ting; Wang, Qianben; Jin, Victor X.; Chen, Chun-Liang; Huang, Tim H.-M.	CANCER RESEARCH	2018	78	4	21	7
Liquid Biopsy-Analysis of Circulating Tumor DNA (ctDNA) in Bladder Cancer	Todenhoefer, Tilman; Struss, Werner J.; Seiler, Roland; Wyatt, Alexander William; Black, Peter C.	BLADDER CANCER	2018	4	1	21	7
Rapid, ultra low coverage copy number profiling of cell-free DNA as a precision oncology screening strategy	Hovelson, Daniel H.; Liu, Chia-Jen; Wang, Yugang; Kang, Qing; Henderson, James; Gursky, Amy; Brockman, Scott; Ramnath, Nithya; Krauss, John C.; Talpaz, Moshe; Kandarpa, Malathi; Chugh, Rashmi; Tuck, Missy; Herman, Kirk; Grasso, Catherine S.; Quist, Michael J.; Feng, Felix Y.; Haakenson, Christine; Langmore, John; Kamberov, Emmanuel; Tesmer, Tim; Husain, Hatim; Lonigro, Robert J.; Robinson, Dan; Smith, David C.; Alva, Ajjai S.; Hussain, Maha H.; Chinnaiyan, Arul M.; Tewari, Muneesh; Mills, Ryan E.; Morgan, Todd M.; Tomlins, Scott A.	ONCOTARGET	2017	8	52	21	5,25
Up-regulation of Biglycan is Associated with Poor Prognosis and PTEN Deletion in Patients with Prostate Cancer	Jacobsen, Frank; Kraft, Juliane; Schroeder, Cornelia; Hube-Magg, Claudia; Kluth, Martina; Lang, Dagmar S.; Simon, Ronald; Sauter, Guido; Izbicki, Jakob R.; Clauditz, Till S.; Luebke, Andreas M.; Hinsch, Andrea; Wilczak, Waldemar; Wittmer, Corinna; Buescheck, Franziska; Hoeflmayer, Doris; Minner, Sarah; Tsourlakis, Maria Christina; Huland, Hartwig; Graefen, Markus; Budaeus, Lars; Thederan, Imke; Salomon, Georg; Schlomm, Thorsten; Melling, Nathaniel	NEOPLASIA	2017	19	9	21	5,25

Non-invasive urinary metabolomic profiling discriminates prostate cancer from benign prostatic hyperplasia	Perez-Rambla, Clara; Puchades-Carrasco, Leonor; Garcia-Flores, Maria; Rubio-Briones, Jose; Antonio Lopez-Guerrero, Jose; Pineda-Lucena, Antonio	METABOLOMICS	2017	13	5	21	5,25
Acquired CYP19A1 amplification is an early specific mechanism of aromatase inhibitor resistance in ER alpha metastatic breast cancer	Magnani, Luca; Frige, Gianmaria; Gadaleta, Raffaella Maria; Corleone, Giacomo; Fabris, Sonia; Kempe, Hermannus; Verschure, Pernette J.; Barozzi, Iros; Vircillo, Valentina; Hong, Sung-Pil; Perone, Ylenia; Saini, Massimo; Trumpp, Andreas; Viale, Giuseppe; Neri, Antonino; Ali, Simak; Colleoni, Marco Angelo; Pruneri, Giancarlo; Minucci, Saverio	NATURE GENETICS	2017	49	3	21	5,25
A novel non-canonical Wnt signature for prostate cancer aggressiveness	Sandsmark, Elise; Hansen, Ailin Falkmo; Selnaes, Kirsten M.; Bertilsson, Helena; Bofin, Anna M.; Wright, Alan J.; Viset, Trond; Richardsen, Elin; Drablos, Finn; Bathen, Tone F.; Tessem, May-Britt; Rye, Morten B.	ONCOTARGET	2017	8	6	21	5,25
The molecular underpinnings of prostate cancer: impacts on management and pathology practice	Rodrigues, Daniel Nava; Boysen, Gunther; Sumanasuriya, Semini; Seed, George; De Marzo, Angelo M.; de Bono, Johann	JOURNAL OF PATHOLOGY	2017	241	2	21	5,25
Identification and validation of potential prognostic gene biomarkers for predicting survival in patients with acute myeloid leukemia	Huang, Rui; Liao, Xiwen; Li, Qiaochuan	ONCOTARGETS AND THERAPY	2017	10		21	5,25
MicroRNA and Transcription Factor Gene Regulatory Network Analysis Reveals Key Regulatory Elements Associated with Prostate Cancer Progression	Sadeghi, Mehdi; Ranjbar, Bijan; Ganjalikhany, Mohamad Reza; Khan, Faiz M.; Schmitz, Ulf; Wolkenhauer, Olaf; Gupta, Shailendra K.	PLOS ONE	2016	11	12	21	4,2

Emergence of the Noncoding Cancer Genome: A Target of Genetic and Epigenetic Alterations	Zhou, Stanley; Treloar, Aislinn E.; Lupien, Mathieu	CANCER DISCOVERY	2016	6	11	21	4,2
Pharmacogenetic Discovery in CALGB (Alliance) 90401 and Mechanistic Validation of a VAC14 Polymorphism that Increases Risk of Docetaxel-Induced Neuropathy	Hertz, Daniel L.; Owzar, Kouros; Lessans, Sherrie; Wing, Claudia; Jiang, Chen; Kelly, William Kevin; Patel, Jai; Halabi, Susan; Furukawa, Yoichi; Wheeler, Heather E.; Sibley, Alexander B.; Lassiter, Cameron; Weisman, Lois; Watson, Dorothy; Krens, Stefanie D.; Mulkey, Flora; Renn, Cynthia L.; Small, Eric J.; Febbo, Phillip G.; Shterev, Ivo; Kroetz, Deanna L.; Friedman, Paula N.; Mahoney, John F.; Carducci, Michael A.; Kelley, Michael J.; Nakamura, Yusuke; Kubo, Michiaki; Dorsey, Susan G.; Dolan, M. Eileen; Morris, Michael J.; Ratain, Mark J.; McLeod, Howard L.	CLINICAL CANCER RESEARCH	2016	22	19	21	4,2
Importance of Estrogenic Signaling and Its Mediated Receptors in Prostate Cancer	Lau, Kin-Mang; To, Ka-Fai	INTERNATIONAL JOURNAL OF MOLECULAR SCIENCES	2016	17	9	21	4,2
The Early Effects of Rapid Androgen Deprivation on Human Prostate Cancer	Shaw, Greg L.; Whitaker, Hayley; Corcoran, Marie; Dunning, Mark J.; Luxton, Hayley; Kay, Jonathan; Massie, Charlie E.; Miller, Jodi L.; Lamb, Alastair D.; Ross-Adams, Helen; Russell, Roslin; Nelson, Adam W.; Eldridge, Matthew D.; Lynch, Andrew G.; Ramos-Montoya, Antonio; Mills, Ian G.; Taylor, Angela E.; Arlt, Wiebke; Shah, Nimish; Warren, Anne Y.; Neal, David E.	EUROPEAN UROLOGY	2016	70	2	21	4,2
Whole-genome DNA methylation and hydroxymethylation profiling for HBV-related hepatocellular carcinoma	Ye, Chao; Tao, Ran; Cao, Qingyi; Zhu, Danhua; Wang, Yini; Wang, Jie; Lu, Juan; Chen, Ermei; Li, Lanjuan	INTERNATIONAL JOURNAL OF ONCOLOGY	2016	49	2	21	4,2

Complex-I Alteration and Enhanced Mitochondrial Fusion Are Associated With Prostate Cancer Progression	Philley, Julie V.; Kannan, Anbarasu; Qin, Wenyi; Sauter, Edward R.; Ikebe, Mitsuo; Hertweck, Kate L.; Troyer, Dean A.; Semmes, Oliver J.; Dasgupta, Santanu	JOURNAL OF CELLULAR PHYSIOLOGY	2016	231	6	21	4,2
Integrated analysis of the prostate cancer small-nucleolar transcriptome reveals SNORA55 as a driver of prostate cancer progression	Crea, Francesco; Quagliata, Luca; Michael, Agnieszlza; Liu, Hui Hsuan; Frumento, Paolo; Azad, Arun A.; Xue, Hui; Pikor, Larissa; Watahiki, Akira; Morant, Rudolf; Eppenberger-Castori, Serenella; Wang, Yuwei; Parolia, Abhijit; Lennox, Kim A.; Lam, Wan L.; Gleave, Martin; Chi, Kim N.; Pandha, Hardev; Wang, Yuzhuo; Helgason, Cheryl D.	MOLECULAR ONCOLOGY	2016	10	5	21	4,2
Hunting the genes in male-pattern alopecia: how important are they, how close are we and what will they tell us?	Heilmann-Heimbach, Stefanie; Hochfeld, Lara M.; Paus, Ralf; Noethen, Markus M.	EXPERIMENTAL DERMATOLOGY	2016	25	4	21	4,2
The UGT2B28 Sex-steroid Inactivation Pathway Is a Regulator of Steroidogenesis and Modifies the Risk of Prostate Cancer Progression	Belledant, Anais; Hovington, Helene; Garcia, Luciana; Caron, Patrick; Brisson, Herve; Villeneuve, Lyne; Simonyan, David; Tetu, Bernard; Fradet, Yves; Lacombe, Louis; Guillemette, Chantal; Levesque, Eric	EUROPEAN UROLOGY	2016	69	4	21	4,2
SRM/MRM targeted proteomics as a tool for biomarker validation and absolute quantification in human urine	Mermelekas, George; Vlahou, Antonia; Zoidakis, Jerome	EXPERT REVIEW OF MOLECULAR DIAGNOSTICS	2015	15	11	21	3,5
Incorporating Genetic Biomarkers into Predictive Models of Normal Tissue Toxicity	Barnett, G. C.; Kerns, S. L.; Noble, D. J.; Dunning, A. M.; West, C. M. L.; Burnet, N. G.	CLINICAL ONCOLOGY	2015	27	10	21	3,5
MicroRNA-205 inhibits cancer cell migration and invasion via modulation of centromere protein F regulating pathways in prostate cancer	Nishikawa, Rika; Goto, Yusuke; Kurozumi, Akira; Matsushita, Ryosuke; Enokida, Hideki; Kojima, Satoko; Naya, Yukio; Nakagawa, Masayuki; Ichikawa, Tomohiko; Seki, Naohiko	INTERNATIONAL JOURNAL OF UROLOGY	2015	22	9	21	3,5
Annexin A4 and cancer	Wei, Bin; Guo, Chunmei; Liu, Shuqing; Sun, Ming-Zhong	CLINICA CHIMICA ACTA	2015	447		21	3,5

Downregulation of EphA5 by promoter methylation in human prostate cancer	Li, Shibao; Zhu, Yingfeng; Ma, Chunguang; Qiu, Zhenhua; Zhang, Xinju; Kang, Zhihua; Wu, Zhiyuan; Wang, Hua; Xu, Xiao; Zhang, Hu; Ren, Guoqiang; Tang, Jianmin; Li, Xiangyu; Guan, Ming	BMC CANCER	2015	15		21	3,5
Plumbagin elicits differential proteomic responses mainly involving cell cycle, apoptosis, autophagy, and epithelial-to-mesenchymal transition pathways in human prostate cancer PC-3 and DU145 cells	Qiu, Jia-Xuan; Zhou, Zhi-Wei; He, Zhi-Xu; Zhao, Ruan Jin; Zhang, Xueji; Yang, Lun; Zhou, Shu-Feng; Mao, Zong-Fu	DRUG DESIGN DEVELOPMENT AND THERAPY	2015	9		21	3,5
Targeting FOXA1-mediated repression of TGF-beta signaling suppresses castration-resistant prostate cancer progression	Song, Bing; Park, Su-Hong; Zhao, Jonathan C.; Fong, Ka-Wing; Li, Shangze; Lee, Yongik; Yang, Yeqing A.; Sridhar, Subhasree; Lu, Xiaodong; Abdulkadir, Sarki A.; Vessella, Robert L.; Morrissey, Colm; Kuzel, Timothy M.; Catalona, William; Yang, Ximing; Yu, Jindan	JOURNAL OF CLINICAL INVESTIGATION	2019	129	2	20	10

Patient-derived Models of Abiraterone- and Enzalutamide-resistant Prostate Cancer Reveal Sensitivity to Ribosome-directed Therapy	Lawrence, Mitchell G.; Obinata, Daisuke; Sandhu, Shahneen; Selth, Luke A.; Wong, Stephen Q.; Porter, Laura H.; Lister, Natalie; Pook, David; Pezaro, Carmel J.; Goode, David L.; Rebello, Richard J.; Clark, Ashlee K.; Papargiris, Melissa; Van Gramberg, Jenna; Hanson, Adrienne R.; Banks, Patricia; Wang, Hong; Niranjan, Birunthi; Keerthikumar, Shivakumar; Hedwards, Shelley; Huglo, Alisee; Yang, Rendong; Henzler, Christine; Li, Yingming; Lopez-Campos, Fernando; Castro, Elena; Toivanen, Roxanne; Azad, Arun; Bolton, Damien; Goad, Jeremy; Grummet, Jeremy; Harewood, Laurence; Kourambas, John; Lawrentschuk, Nathan; Moon, Daniel; Murphy, Declan G.; Sengupta, Shomik; Snow, Ross; Thorne, Heather; Mitchell, Catherine; Pedersen, John; Clouston, David; Norden, Sam; Ryan, Andrew; Dehm, Scott M.; Tilley, Wayne D.; Pearson, Richard B.; Hannan, Ross D.; Frydenberg, Mark; Furic, Luc; Taylor, Renea A.; Risbridger, Gail P.	EUROPEAN UROLOGY	2018	74	5	20	6,67
A Plasma Biomarker Panel of Four MicroRNAs for the Diagnosis of Prostate Cancer	Matin, Farhana; Jeet, Varinder; Moya, Leire; Selth, Luke A.; Chambers, Suzanne; Clements, Judith A.; Batra, Jyotsna	SCIENTIFIC REPORTS	2018	8		20	6,67
Tamoxifen Resistance in Breast Cancer Is Regulated by the EZH2-ER alpha-GREB1 Transcriptional Axis	Wu, Yanming; Zhang, Zhao; Cenciarini, Mauro E.; Proietti, Cecilia J.; Amasino, Matias; Hong, Tao; Yang, Mei; Liao, Yiji; Chiang, Huai-Chin; Kaklamani, Virginia G.; Jeselsohn, Rinath; Vadlamudi, Ratna K.; Huang, Tim Hui-Ming; Li, Rong; De Angelis, Carmine; Fu, Xiaoyong; Elizalde, Patricia V.; Schiff, Rachel; Brown, Myles; Xu, Kexin	CANCER RESEARCH	2018	78	3	20	6,67

Validation of a Genomic Risk Classifier to Predict Prostate Cancer-specific Mortality in Men with Adverse Pathologic Features	Karnes, R. Jeffrey; Choeurng, Voleak; Ross, Ashley E.; Schaeffer, Edward M.; Klein, Eric A.; Freedland, Stephen J.; Erho, Nicholas; Yousefi, Kasra; Takhar, Mandeep; Davicioni, Elai; Cooperberg, Matthew R.; Trock, Bruce J.	EUROPEAN UROLOGY	2018	73	2	20	6,67
Metabolomic estimation of the diagnosis of hepatocellular carcinoma based on ultrahigh performance liquid chromatography coupled with time-of-flight mass spectrometry	Li, Yuan-Feng; Qiu, Shi; Gao, Li-Juan; Zhang, Ai-Hua	RSC ADVANCES	2018	8	17	20	6,67
Aberrant Activation of a Gastrointestinal Transcriptional Circuit in Prostate Cancer Mediates Castration Resistance	Shukla, Shipra; Cyrta, Joanna; Murphy, Devan A.; Walczak, Edward G.; Ran, Leili; Agrawal, Praveen; Xie, Yuanyuan; Chen, Yuedan; Wang, Shangqian; Zhan, Yu; Li, Dan; Wong, Elissa W. P.; Sboner, Andrea; Beltran, Himisha; Mosquera, Juan Miguel; Sher, Jessica; Cao, Zhen; Wongvipat, John; Koche, Richard P.; Gopalan, Anuradha; Zheng, Deyou; Rubin, Mark A.; Scher, Howard I.; Chi, Ping; Chen, Yu	CANCER CELL	2017	32	6	20	5
Biomarker significance of plasma and tumor miR-21, miR-221, and miR-106a in osteosarcoma	Nakka, Manjula; Allen-Rhoades, Wendy; Li, Yiting; Kelly, Aaron J.; Shen, Jianhe; Taylor, Aaron M.; Barkauskas, Donald A.; Yustein, Jason T.; Andrulis, Irene L.; Wunder, Jay S.; Gorlick, Richard; Meltzer, Paul S.; Lau, Ching C.; Man, Tsz-Kwong	ONCOTARGET	2017	8	57	20	5
The Novel Association of Circulating Tumor Cells and Circulating Megakaryocytes with Prostate Cancer Prognosis	Xu, Lei; Mao, Xueying; Guo, Tianyu; Chan, Pui Ying; Shaw, Greg; Hines, John; Stankiewicz, Elzbieta; Wang, Yuqin; Oliver, R. Tim D.; Ahmad, Amar Sabri; Berney, Daniel; Shamash, Jonathan; Lu, Yong-Jie	CLINICAL CANCER RESEARCH	2017	23	17	20	5

Novel Androgen Receptor Coregulator GRHL2 Exerts Both Oncogenic and Antimetastatic Functions in Prostate Cancer	Paltoglou, Steve; Das, Rajdeep; Townley, Scott L.; Hickey, Theresa E.; Tarulli, Gerard A.; Coutinho, Isabel; Fernandes, Rayzel; Hanson, Adrienne R.; Denis, Iza; Carroll, Jason S.; Dehm, Scott M.; Raj, Ganesh V.; Plymate, Stephen R.; Tilley, Wayne D.; Selth, Luke A.	CANCER RESEARCH	2017	77	13	20	5
Demographic, lifestyle, and genetic determinants of circulating concentrations of 25-hydroxyvitamin D and vitamin D-binding protein in African American and European American women	Yao, Song; Hong, Chi-Chen; Bandera, Elisa V.; Zhu, Qianqian; Liu, Song; Cheng, Ting-Yuan David; Zirpoli, Gary; Haddad, Stephen A.; Lunetta, Kathryn L.; Ruiz-Narvaez, Edward A.; McCann, Susan E.; Troester, Melissa A.; Rosenberg, Lynn; Palmer, Julie R.; Olshan, Andrew F.; Ambrosone, Christine B.	AMERICAN JOURNAL OF CLINICAL NUTRITION	2017	105	6	20	5
Chromosomal Instability in Cell-Free DNA as a Highly Specific Biomarker for Detection of Ovarian Cancer in Women with Adnexal Masses	Vanderstichele, Adriaan; Busschaert, Pieter; Smeets, Dominiek; Landolfo, Chiara; Van Nieuwenhuysen, Els; Leunen, Karin; Neven, Patrick; Amant, Frederic; Mahner, Sven; Braicu, Elena Ioana; Zeilinger, Robert; Coosemans, An; Timmerman, Dirk; Lambrechts, Diether; Vergote, Ignace	CLINICAL CANCER RESEARCH	2017	23	9	20	5
Genome-wide DNA methylation measurements in prostate tissues uncovers novel prostate cancer diagnostic biomarkers and transcription factor binding patterns	Kirby, Marie K.; Ramaker, Ryne C.; Roberts, Brian S.; Lasseigne, Brittany N.; Gunther, David S.; Burwell, Todd C.; Davis, Nicholas S.; Gulzar, Zulficiar G.; Absher, Devin M.; Cooper, Sara J.; Brooks, James D.; Myers, Richard M.	BMC CANCER	2017	17		20	5
Role of steroid receptor and coregulator mutations in hormone-dependent cancers	Groner, Anna C.; Brown, Myles	JOURNAL OF CLINICAL INVESTIGATION	2017	127	4	20	5
Circulating tumor cells capture disease evolution in advanced prostate cancer	Lack, Justin; Gillard, Marc; Cam, Maggie; Paner, Gladell P.; VanderWeele, David J.	JOURNAL OF TRANSLATIONAL MEDICINE	2017	15		20	5

Integration of VDR genome wide binding and GWAS genetic variation data reveals co-occurrence of VDR and NF-kappa B binding that is linked to immune phenotypes	Singh, Prashant K.; van den Berg, Patrick R.; Long, Mark D.; Vreugdenhil, Angie; Grieshober, Laurie; Ochs-Balcom, Heather M.; Wang, Jianmin; Delcambre, Sylvie; Heikkinen, Sami; Carlberg, Carsten; Campbell, Moray J.; Sucheston-Campbell, Lara E.	BMC GENOMICS	2017	18		20	5
Therapeutic Targeting of Epithelial Plasticity Programs: Focus on the Epithelial-Mesenchymal Transition	Malek, Reem; Wang, Hailun; Kekoa, Taparra; Tran, Phuoc T.	CELLS TISSUES ORGANS	2017	203	2	20	5
Detection of aggressive prostate cancer associated glycoproteins in urine using glycoproteomics and mass spectrometry	Jia, Xingwang; Chen, Jing; Sun, Shisheng; Yang, Weiming; Yang, Shuang; Shah, Punit; Hoti, Naseruddin; Veltri, Bob; Zhang, Hui	PROTEOMICS	2016	16	23	20	4
Antibody-Array-Based Proteomic Screening of Serum Markers in Systemic Lupus Erythematosus: A Discovery Study	Wu, Tianfu; Ding, Huihua; Han, Jie; Arriens, Cristina; Wei, Chungwen; Han, Weilu; Pedroza, Claudia; Jiang, Shan; Anolik, Jennifer; Petri, Michelle; Sanz, Ignacio; Saxena, Ramesh; Mohan, Chandra	JOURNAL OF PROTEOME RESEARCH	2016	15	7	20	4
The Landscape of Prognostic Outlier Genes in High-Risk Prostate Cancer	Zhao, Shuang G.; Evans, Joseph R.; Kothari, Vishal; Sun, Grace; Larm, Ashley; Mondine, Victor; Schaeffer, Edward M.; Ross, Ashley E.; Klein, Eric A.; Den, Robert B.; Dicker, Adam P.; Karnes, R. Jeffrey; Erho, Nicholas; Nguyen, Paul L.; Davicioni, Elai; Feng, Felix Y.	CLINICAL CANCER RESEARCH	2016	22	7	20	4
Development of Castration Resistant Prostate Cancer can be Predicted by a DNA Hypermethylation Profile	Angulo, Javier C.; Andres, Guillermo; Ashour, Nadia; Sanchez-Chapado, Manuel; Lopez, Jose I.; Ropero, Santiago	JOURNAL OF UROLOGY	2016	195	3	20	4
Unravelling the transcriptomic landscape of the major phase II UDP-glucuronosyltransferase drug metabolizing pathway using targeted RNA sequencing	Tourancheau, A.; Margaillan, G.; Rouleau, M.; Gilbert, I.; Villeneuve, L.; Levesque, E.; Droit, A.; Guillemette, C.	PHARMACOGENO MICS JOURNAL	2016	16	1	20	4

Targeting Androgen/Estrogen Receptors Crosstalk in Cancer	Karamouzis, Michalis V.; Papavassiliou, Kostas A.; Adamopoulos, Christos; Papavassiliou, Athanasios G.	TRENDS IN CANCER	2016	2	1	20	4
Differentially Expressed Genes and Signature Pathways of Human Prostate Cancer	Myers, Jennifer S.; von Lersner, Ariana K.; Robbins, Charles J.; Sang, Qing-Xiang Amy	PLOS ONE	2015	10	12	20	3,33
Methylseleninic acid promotes antitumour effects via nuclear FOXO3a translocation through Akt inhibition	Tarrado-Castellarnau, Miriam; Cortes, Roldan; Zanuy, Miriam; Tarrago-Celada, Josep; Polat, Ibrahim H.; Hill, Richard; Fan, Teresa W. M.; Link, Wolfgang; Cascante, Marta	PHARMACOLOGICAL RESEARCH	2015	102		20	3,33
Hormone-related pathways and risk of breast cancer subtypes in African American women	Haddad, Stephen A.; Lunetta, Kathryn L.; Ruiz-Narvaez, Edward A.; Bensen, Jeannette T.; Hong, Chi-Chen; Sucheston-Campbell, Lara E.; Yao, Song; Bandera, Elisa V.; Rosenberg, Lynn; Haiman, Christopher A.; Troester, Melissa A.; Ambrosone, Christine B.; Palmer, Julie R.	BREAST CANCER RESEARCH AND TREATMENT	2015	154	1	20	3,33
FOXA1 regulates androgen receptor variant activity in models of castrate-resistant prostate cancer	Jones, Dominic; Wade, Mark; Nakjang, Sirintra; Chaytor, Lewis; Grey, James; Robson, Craig N.; Gaughan, Luke	ONCOTARGET	2015	6	30	20	3,33
Genomic deletion of chromosome 12p is an independent prognostic marker in prostate cancer	Kluth, Martina; Ahrary, Ramin; Hube-Magg, Claudia; Ahmed, Malik; Volta, Heinke; Schwemin, Catina; Steurer, Stefan; Wittmer, Corinna; Wilczak, Waldemar; Krech, Eike Burandt Till; Adam, Meike; Michl, Uwe; Heinzer, Hans; Salomon, Georg; Graefen, Markus; Koop, Christina; Minner, Sarah; Simon, Ronald; Sauter, Guido; Schlomm, Thorsten	ONCOTARGET	2015	6	29	20	3,33
gamma H2AX assay in ex vivo irradiated tumour specimens: A novel Method to determine tumour radiation sensitivity in patient-derived material	Menegakis, Apostolos; von Neubeck, Claere; Yaromina, Ala; Thames, Howard; Hering, Sandra; Hennenlotter, Joerg; Scharpf, Marcus; Noell, Susan; Krause, Mechthild; Zips, Daniel; Baumann, Michael	RADIOTHERAPY AND ONCOLOGY	2015	116	3	20	3,33

Estrogen receptor alpha drives proliferation in PTEN-deficient prostate carcinoma by stimulating survival signaling, MYC expression and altering glucose sensitivity	Takizawa, Itsuhiro; Lawrence, Mitchell G.; Balanathan, Preetika; Rebello, Richard; Pearson, Helen B.; Garg, Elika; Pedersen, John; Pouliot, Normand; Nadon, Robert; Watt, Matthew J.; Taylor, Renea A.; Humbert, Patrick; Topisirovic, Ivan; Larsson, Ola; Risbridger, Gail P.; Furic, Luc	ONCOTARGET	2015	6	2	20	3,33
Identifying Actionable Targets through Integrative Analyses of GEM Model and Human Prostate Cancer Genomic Profiling	Wanjala, Jackie; Taylor, Barry S.; Chapinski, Caren; Hieronymus, Haley; Wongvipat, John; Chen, Yu; Nanjangud, Gouri J.; Schultz, Nikolaus; Xie, Yingqiu; Liu, Shenji; Lu, Wenfu; Yang, Qing; Sander, Chris; Chen, Zhenbang; Sawyers, Charles L.; Carver, Brett S.	MOLECULAR CANCER THERAPEUTICS	2015	14	1	20	3,33
Long non-coding RNAs as monitoring tools and therapeutic targets in breast cancer	Luisa Pecero, Ma; Salvador-Bofill, Javier; Molina-Pinelo, Sonia	CELLULAR ONCOLOGY	2019	42	1	19	9,5
ONECUT2 is a driver of neuroendocrine prostate cancer	Guo, Haiyang; Ci, Xinpei; Ahmed, Musaddeque; Hua, Junjie Tony; Soares, Fraser; Lin, Dong; Puca, Loredana; Vosoughi, Aram; Xue, Hui; Li, Estelle; Su, Peiran; Chen, Sujun; Tran Nguyen; Liang, Yi; Zhang, Yuzhe; Xu, Xin; Xu, Jing; Sheahan, Anjali V.; Ba-Alawi, Wail; Zhang, Si; Mahamud, Osman; Vellanki, Ravi N.; Gleave, Martin; Bristow, Robert G.; Haibe-Kains, Benjamin; Poirier, John T.; Rudin, Charles M.; Tsao, Ming-Sound; Wouters, Bradly G.; Fazli, Ladan; Feng, Felix Y.; Ellis, Leigh; van der Kwast, Theo; Berlin, Alejandro; Koritzinsky, Marianne; Boutros, Paul C.; Zoubeidi, Amina; Beltran, Himisha; Wang, Yuzhuo; He, Housheng Hansen	NATURE COMMUNICATION S	2019	10		19	9,5
Therapeutic Inhibition of Myc in Cancer. Structural Bases and Computer-Aided Drug Discovery Approaches	Carabet, Lavinia A.; Rennie, Paul S.; Cherkasov, Artem	INTERNATIONAL JOURNAL OF MOLECULAR SCIENCES	2019	20	1	19	9,5

Merging new-age biomarkers and nanodiagnostics for precision prostate cancer management	Koo, Kevin M.; Mainwaring, Paul N.; Tomlins, Scott A.; Trau, Matt	NATURE REVIEWS UROLOGY	2019	16	5	18	9
PTEN as a Prognostic/Predictive Biomarker in Cancer: An Unfulfilled Promise?	Bazzichetto, Chiara; Conciatori, Fabiana; Pallocca, Matteo; Falcone, Italia; Fanciulli, Maurizio; Cognetti, Francesco; Milella, Michele; Ciuffreda, Ludovica	CANCERS	2019	11	4	18	9
High miR-454-3p expression predicts poor prognosis in hepatocellular carcinoma	Li, Yanqing; Jiao, Yan; Fu, Zhuo; Luo, Zhangping; Su, Jing; Li, Yang	CANCER MANAGEMENT AND RESEARCH	2019	11		18	9
Prostate-specific Membrane Antigen Heterogeneity and DNA Repair Defects in Prostate Cancer	Paschalis, Alec; Sheehan, Beshara; Riisnaes, Ruth; Rodrigues, Daniel Nava; Gurel, Bora; Bertan, Claudia; Ferreira, Ana; Lambros, Maryou B. K.; Seed, George; Yuan, Wei; Dolling, David; Welti, Jon C.; Neeb, Antje; Sumanasuriya, Semini; Rescigno, Pasquale; Bianchini, Diletta; Tunariu, Nina; Carreira, Suzanne; Sharp, Adam; Oyen, Wim; de Bono, Johann S.	EUROPEAN UROLOGY	2019	76	4	17	8,5
Exosomes, new biomarkers in early cancer detection	Jalalian, Seyed Hamid; Ramezani, Mohammad; Jalalian, Seyed Ali; Abnous, Khalil; Taghdisi, Seyed Mohammad	ANALYTICAL BIOCHEMISTRY	2019	571		17	8,5
The Proteogenomic Landscape of Curable Prostate Cancer	Sinha, Ankit; Huang, Vincent; Livingstone, Julie; Wang, Jenny; Fox, Natalie S.; Kurganovs, Natalie; Ignatchenko, Vladimir; Fritsch, Katharina; Donmez, Nilgun; Heisler, Lawrence E.; Shiah, Yu-Jia; Yao, Cindy Q.; Alfaro, Javier A.; Volik, Stas; Lapuk, Anna; Fraser, Michael; Kron, Ken; Murison, Alex; Lupien, Mathieu; Sahinalp, Cenk; Collins, Colin C.; Tetu, Bernard; Masoomian, Mehdi; Berman, David M.; van der Kwast, Theodorus; Bristow, Robert G.; Kislinger, Thomas; Boutros, Paul C.	CANCER CELL	2019	35	3	17	8,5

The Long Noncoding RNA TTTY15, Which Is Located on the Y Chromosome, Promotes Prostate Cancer Progression by Sponging let-7	Xiao, Guang'an; Yao, Jingjing; Kong, Depei; Ye, Chen; Chen, Rui; Li, Li; Zeng, Tao; Wang, Liujun; Zhang, Wei; Shi, Xiaolei; Zhou, Tie; Li, Jing; Wang, Yue; Xu, Chuan Liang; Jiang, Junfeng; Sun, Yinghao	EUROPEAN UROLOGY	2019	76	3	16	8
Identification of the perturbed metabolic pathways associating with prostate cancer cells and anticancer affects of obacunone	Xie, Jing; Zhang, Ai-hua; Qiu, Shi; Zhang, Tian-lei; Li, Xian-na; Yan, Guang-li; Sun, Hui; Liu, Liang; Wang, Xi-jun	JOURNAL OF PROTEOMICS	2019	206		16	8
Genome-wide CRISPR screens reveal synthetic lethality of RNASEH2 deficiency and ATR inhibition	Wang, Chao; Wang, Gang; Feng, Xu; Shepherd, Peter; Zhang, Jie; Tang, Mengfan; Chen, Zhen; Srivastava, Mrinal; McLaughlin, Megan E.; Navone, Nora M.; Hart, Glen Traver; Chen, Junjie	ONCOGENE	2019	38	14	16	8
The Microbiome and Genitourinary Cancer: A Collaborative Review	Markowski, Mark C.; Boorjian, Stephen A.; Burton, Jeremy P.; Hahn, Noah M.; Ingersoll, Molly A.; Vareki, Saman Maleki; Pal, Sumanta K.; Sfanos, Karen S.	EUROPEAN UROLOGY	2019	75	4	16	8
Advancing Personalized Medicine Through the Application of Whole Exome Sequencing and Big Data Analytics	Suwinski, Pawel; Ong, ChuangKee; Ling, Maurice H. T.; Poh, Yang Ming; Khan, Asif M.; Ong, Hui San	FRONTIERS IN GENETICS	2019	10		16	8
N-Myc mediated epigenetic reprogramming drives lineage plasticity in advanced prostate cancer	Berger, Adeline; Brady, Nicholas J.; Bareja, Rohan; Robinson, Brian; Conteduca, Vincenza; Augello, Michael A.; Ruca, Loredana; Ahmed, Adnan; Dardenne, Etienne; Lu, Xiaodong; Hwang, Inah; Bagadion, Alyssa M.; Sboner, Andrea; Elemento, Olivier; Paik, Jihye; Yu, Jindan; Barbieri, Christopher E.; Dephoure, Noah; Beltran, Himisha; Rickman, David S.	JOURNAL OF CLINICAL INVESTIGATION	2019	129	9	15	7,5

Long noncoding RNA PCAT6 inhibits colon cancer cell apoptosis by regulating anti-apoptotic protein ARC expression via EZH2	Huang, Weimei; Su, Geng; Huang, Xiaoxian; Zou, Angru; Wu, Jingfang; Yang, Yunchu; Zhu, Yaru; Liang, Shumei; Li, Deyu; Ma, Feng; Guo, Linlang	CELL CYCLE	2019	18	1	15	7,5
Current Trends in Cancer Biottiarker Discovery Using Urinary Metabolomics: Achievements and New Challenges	Burton, Casey; Ma, Yinfa	CURRENT MEDICINAL CHEMISTRY	2019	26	1	15	7,5
Exosomes for Non-Invasive Cancer Monitoring	Kalishwaralal, Kalimuthu; Kwon, Woo Young; Park, Ki Soo	BIOTECHNOLOGY JOURNAL	2019	14	1	15	7,5
The role of microRNAs in prostate cancer migration, invasion, and metastasis	Aghdam, Shirin Golabi; Ebrazeh, Mehrdad; Hemmatzadeh, Maryam; Seyfizadeh, Narges; Shabgah, Arezoo Gowhari; Azizi, Gholamreza; Ebrahimi, Negin; Babaie, Farhad; Mohammadi, Hamed	JOURNAL OF CELLULAR PHYSIOLOGY	2019	234	7	14	7
A Rich Array of Prostate Cancer Molecular Biomarkers: Opportunities and Challenges	Kohaar, Indu; Petrovics, Gyorgy; Srivastava, Shiv	INTERNATIONAL JOURNAL OF MOLECULAR SCIENCES	2019	20	8	14	7
Growth Hormone's Links to Cancer	Boguszewski, Cesar Luiz; da Silva Boguszewski, Margaret Cristina	ENDOCRINE REVIEWS	2019	40	2	14	7
The evolution of long noncoding RNA acceptance in prostate cancer initiation, progression, and its clinical utility in disease management	Ramnarine, Varune Rohan; Kobelev, Maxim; Gibb, Ewan A.; Nouri, Mannan; Lin, Dong; Wang, Yuzhuo; Buttyan, Ralph; Davicioni, Elai; Zoubeidi, Amina; Collins, Colin C.	EUROPEAN UROLOGY	2019	76	5	13	6,5
Distinct structural classes of activating FOXA1 alterations in advanced prostate cancer	Parolia, Abhijit; Cieslik, Marcin; Chu, Shih-Chun; Xiao, Lanbo; Ouchi, Takahiro; Zhang, Yuping; Wang, Xiaoju; Vats, Pankaj; Cao, Xuhong; Pitchiaya, Sethuramasundaram; Su, Fengyun; Wang, Rui; Feng, Felix Y.; Wu, Yi-Mi; Lonigro, Robert J.; Robinson, Dan R.; Chinnaiyan, Arul M.	NATURE	2019	571	7765	13	6,5

Review and perspectives on the applications of mass spectrometry imaging under ambient conditions	Perez, Consuelo J.; Bagga, Aafreen K.; Prova, Shamina S.; Taemeh, Maryam Yousefi; Ifa, Demian R.	RAPID COMMUNICATIONS IN MASS SPECTROMETRY	2019	33		13	6,5
Next-Generation Hedgehog/GLI Pathway Inhibitors for Cancer Therapy	Peer, Elisabeth; Tesanovic, Suzana; Aberger, Fritz	CANCERS	2019	11	4	13	6,5
Biological Evolution of Castration-resistant Prostate Cancer	Davies, Alastair; Conteduca, Vincenza; Zoubeidi, Amina; Beltran, Himisha	EUROPEAN UROLOGY FOCUS	2019	5	2	13	6,5
Pirin: a potential novel therapeutic target for castration-resistant prostate cancer regulated by miR-455-5p	Arai, Takayuki; Kojima, Satoko; Yamada, Yasutaka; Sugawara, Sho; Kato, Mayuko; Yamazaki, Kazuto; Naya, Yukio; Ichikawa, Tomohiko; Seki, Naohiko	MOLECULAR ONCOLOGY	2019	13	2	13	6,5
Large oncosomes overexpressing integrin alpha-V promote prostate cancer adhesion and invasion via AKT activation	Ciardiello, Chiara; Leone, Alessandra; Lanuti, Paola; Roca, Maria S.; Moccia, Tania; Minciacchi, Valentina R.; Minopoli, Michele; Gigantino, Vincenzo; De Cecio, Rossella; Rippa, Massimo; Petti, Lucia; Capone, Francesca; Vitagliano, Carlo; Milone, Maria R.; Pucci, Biagio; Lombardi, Rita; Iannelli, Federica; Di Gennaro, Elena; Bruzzese, Francesca; Marchisio, Marco; Carriero, Maria, V; Di Vizio, Dolores; Budillon, Alfredo	JOURNAL OF EXPERIMENTAL & CLINICAL CANCER RESEARCH	2019	38		12	6
Long noncoding RNA PCAT1, a novel serum-based biomarker, enhances cell growth by sponging miR-326 in oesophageal squamous cell carcinoma	Huang, Lijie; Wang, Yan; Chen, Jiao; Wang, Yu; Zhao, Yabing; Wang, Yali; Ma, Yunping; Chen, Xin; Liu, Wenzhong; Li, Zhengzheng; Zhao, Lianmei; Shan, Baoen; Dong, Xin; Li, Dan; Shao, Shujuan; Song, Yongmei; Zhan, Qimin; Liu, Xuefeng	CELL DEATH & DISEASE	2019	10		12	6

DNA-Methylation-Based Detection of Urological Cancer in Urine: Overview of Biomarkers and Considerations on Biomarker Design, Source of DNA, and Detection Technologies	Larsen, Louise Katrine; Lind, Guro Elisabeth; Guldberg, Per; Dahl, Christina	INTERNATIONAL JOURNAL OF MOLECULAR SCIENCES	2019	20	11	12	6
Overexpression of the ASPM gene is associated with aggressiveness and poor outcome in bladder cancer	Xu, Zhenglin; Zhang, Qi; Luh, Frank; Jin, Baiye; Liu, Xiyong	ONCOLOGY LETTERS	2019	17	2	12	6
Neuroendocrine Differentiation in Prostate Cancer: Emerging Biology, Models, and Therapies	Puca, Loredana; Vlachostergios, Panagiotis J.; Beltran, Himisha	COLD SPRING HARBOR PERSPECTIVES IN MEDICINE	2019	9	2	12	6
Post-GWAS in prostate cancer: from genetic association to biological contribution	Farashi, Samaneh; Kryza, Thomas; Clements, Judith; Batra, Jyotsna	NATURE REVIEWS CANCER	2019	19	1	12	6
Metabolic Regulation of Redox Balance in Cancer	Purohit, Vinee; Simeone, Diane M.; Lyssiotis, Costas A.	CANCERS	2019	11	7	11	5,5
The influence of BRCA2 mutation on localized prostate cancer	Taylor, Renea A.; Fraser, Michael; Rebello, Richard J.; Boutros, Paul C.; Murphy, Declan G.; Bristow, Robert G.; Risbridger, Gail P.	NATURE REVIEWS UROLOGY	2019	16	5	11	5,5
An AR-ERG transcriptional signature defined by long-range chromatin interactomes in prostate cancer cells	Zhang, Zhizhuo; Chng, Kern Rei; Lingadahalli, Shreyas; Chen, Zikai; Liu, Mei Hui; Do, Huy Hoang; Cai, Shaojiang; Rinaldi, Nicola; Poh, Huay Mei; Li, Guoliang; Sung, Ying Ying; Heng, Charlie L.; Core, Leighton J.; Tan, Si Kee; Ruan, Xiaoan; Lis, John T.; Kellis, Manolis; Ruan, Yijun; Sung, Wing-Kin; Cheung, Edwin	GENOME RESEARCH	2019	29	2	11	5,5

Genomic Classifier for Guiding Treatment of Intermediate-Risk Prostate Cancers to Dose-Escalated Image Guided Radiation Therapy Without Hormone Therapy	Berlin, Alejandro; Murgic, Jure; Hosni, Ali; Pintilie, Melania; Salcedo, Adriana; Fraser, Michael; Kamel-Reid, Suzanne; Zhang, Jingbin; Wang, Qiqi; Ch'ng, Carolyn; Deheshi, Samineh; Davicioni, Elai; van der Kwast, Theodorus; Boutros, Paul C.; Bristow, Robert G.; Chua, Melvin L. K.	INTERNATIONAL JOURNAL OF RADIATION ONCOLOGY BIOLOGY PHYSICS	2019	103	1	11	5,5
SRRM4 gene expression correlates with neuroendocrine prostate cancer	Li, Yinan; Zhang, Qingfu; Lovnicki, Jessica; Chen, Ruiqi; Fazli, Ladan; Wang, Yuzhuo; Gleave, Martin; Huang, Jiaoti; Dong, Xuesen	PROSTATE	2019	79	1	11	5,5

Management of Patients with Advanced Prostate Cancer: Report of the Advanced Prostate Cancer Consensus Conference 2019	Gillessena, Silke; Attard, Gerhardt; Beer, Tomasz M.; Beltran, Himisha; Bjartell, Anders; Bossi, Alberto; Briganti, Alberto; Bristow, Rob G.; Chi, Kim N.; Clarke, Noel; Davis, Ian D.; de Bono, Johann; Drake, Charles G.; Duran, Ignacio; Eeles, Ros; Efstathiou, Eleni; Evans, Christopher P.; Fanti, Stefano; Feng, Felix Y.; Fizazi, Karim; Frydenberg, Mark; Gleave, Martin; Halabi, Susan; Heidenreich, Axel; Heinrich, Daniel; Higano, Celestia (Tia) S.; Hofman, Michael S.; Hussain, Maha; James, Nicolas; Kanesvaran, Ravindran; Kantoff, Philip; Khauli, Raja B.; Leibowitz, Raya; Logothetis, Chris; Maluf, Fernando; Millman, Robin; Morgans, Alicia K.; Morris, Michael J.; Mottet, Nicolas; Mrabti, Hind; Murphy, Declan G.; Murthy, Vedang; Oh, William K.; Ost, Piet; OSullivan, Joe M.; Padhani, Anwar R.; Parker, Chris; Poon, Darren M. C.; Pritchard, Colin C.; Reiter, Robert E.; Roach, Mack; Rubin, Mark; Ryan, Charles J.; Saad, Fred; Sade, Juan Pablo; Sartor, Oliver; Scher, Howard, I; Shore, Neal; Small, Eric; Smith, Matthew; Soule, Howard; Sternberg, Cora N.; Steuber, Thomas; Suzuki, Hiroyoshi; Sweeneyh, Christopher; Sydes, Matthew R.; Taplinh, Mary-Ellen; Tombal, Bertrand; Turkeri, Levent; van Oort, Inge; Zapatero, Almudena; Omlind, Aurelius	EUROPEAN UROLOGY	2020	77	4	10	10
The potential and controversy of targeting STAT family members in cancer	Verhoeven, Yannick; Tilborghs, Sam; Jacobs, Julie; De Waele, Jorrit; Quatannens, Delphine; Deben, Christophe; Prenen, Hans; Pauwels, Patrick; Xuan Bich Trinh; Wouters, An; Smits, Evelien L. J.; Lardon, Filip; van Dam, Peter A.	SEMINARS IN CANCER BIOLOGY	2020	60		10	10

Clinical features of neuroendocrine prostate cancer	Conteduca, Vincenza; Oromendia, Clara; Eng, Kenneth W.; Bareja, Rohan; Sigouros, Michael; Molina, Ana; Faltas, Bishoy M.; Sboner, Andrea; Mosquera, Juan Miguel; Elemento, Olivier; Nanus, David M.; Tagawa, Scott T.; Ballman, Karla V.; Beltran, Himisha	EUROPEAN JOURNAL OF CANCER	2019	121		10	5
A positive role of c-Myc in regulating androgen receptor and its splice variants in prostate cancer	Bai, Shanshan; Cao, Subing; Jin, Lianjin; Kobelski, Margaret; Schouest, Blake; Wang, Xiaojie; Ungerleider, Nathan; Baddoo, Melody; Zhang, Wensheng; Corey, Eva; Vessella, Robert L.; Dong, Xuesen; Zhang, Kun; Yu, Xianghui; Flemington, Erik K.; Dong, Yan	ONCOGENE	2019	38	25	10	5
The Metabolic Landscape of Prostate Cancer	Giunchi, Francesca; Fiorentino, Michelangelo; Loda, Massimo	EUROPEAN UROLOGY ONCOLOGY	2019	2	1	10	5
Non-BRCA DNA Damage Repair Gene Alterations and Response to the PARP Inhibitor Rucaparib in Metastatic Castration-Resistant Prostate Cancer: Analysis From the Phase II TRITON2 Study	Abida, Wassim; Campbell, David; Patnaik, Akash; Shapiro, Jeremy D.; Sautois, Brieuc; Vogelzang, Nicholas J.; Voog, Eric G.; Bryce, Alan H.; McDermott, Ray; Ricci, Francesco; Rowe, Julie; Zhang, Jingsong; Piulats, Josep Maria; Fizazi, Karim; Merseburger, Axel S.; Higano, Celestia S.; Krieger, Laurence E.; Ryan, Charles J.; Feng, Felix Y.; Simmons, Andrew D.; Loehr, Andrea; Despain, Darrin; Dowson, Melanie; Green, Foad; Watkins, Simon P.; Golsorkhi, Tony; Chowdhury, Simon	CLINICAL CANCER RESEARCH	2020	26	11	8	8
Hyperpolarized MRI of Human Prostate Cancer Reveals Increased Lactate with Tumor Grade Driven by Monocarboxylate Transporter 1	Granlund, Kristin L.; Tee, Sui-Seng; Vargas, Hebert A.; Lyashchenko, Serge K.; Reznik, Ed; Fine, Samson; Laudone, Vincent; Eastham, James A.; Touijer, Karim A.; Reuter, Victor E.; Gonen, Mithat; Sosa, Ramon E.; Nicholson, Duane; Guo, YanWei W.; Chen, Albert P.; Tropp, James; Robb, Fraser; Hricak, Hedvig; Keshari, Kayvan R.	CELL METABOLISM	2020	31	1	7	7

Wnt-pathway Activating Mutations Are Associated with Resistance to First-line Abiraterone and Enzalutamide in Castration-resistant Prostate Cancer	Velho, Pedro Isaacsson; Fu, Wei; Wang, Hao; Mirkheshti, Nooshin; Qazi, Fahad; Lima, Fabiola A. S.; Shaukat, Farah; Carducci, Michael A.; Denmeade, Samuel R.; Paller, Channing J.; Markowski, Mark C.; Marshall, Catherine H.; Eisenberger, Mario A.; Antonarakis, Emmanuel S.	EUROPEAN UROLOGY	2020	77	1	7	7
Establishment and characterization of stable red, far-red (fR) and near infra-red (NIR) transfected canine prostate cancer cell lines	Liu, Wen; Sender, Sina; Kong, Weibo; Beck, Julia; Sekora, Anett; Bornemann-Kolatzki, Kirsten; Schuetz, Ekkehart; Junghanss, Christian; Brenig, Bertram; Nolte, Ingo; Escobar, Hugo Murua	CANCER CELL INTERNATIONAL	2020	20	1	6	6
Long noncoding RNA SNHG12 indicates the prognosis of prostate cancer and accelerates tumorigenesis via sponging miR-133b	Cheng, Gong; Song, Zhengshuai; Liu, Yuenan; Xiao, Haibing; Ruan, Hailong; Cao, Qi; Wang, Keshan; Xiao, Wen; Xiong, Zhiyong; Liu, Di; Chen, Ke; Zhang, Xiaoping	JOURNAL OF CELLULAR PHYSIOLOGY	2020	235	2	6	6
The Novel ATR Inhibitor BAY 1895344 Is Efficacious as Monotherapy and Combined with DNA Damage-Inducing or Repair-Compromising Therapies in Preclinical Cancer Models	Wengner, Antje M.; Siemeister, Gerhard; Luecking, Ulrich; Lefranc, Julien; Wortmann, Lars; Lienau, Philip; Bader, Benjamin; Boemer, Ulf; Moosmayer, Dieter; Eberspaecher, Uwe; Golfier, Sven; Schatz, Christoph A.; Baumgart, Simon J.; Haendler, Bernard; Lejeune, Pascale; Schlicker, Andreas; von Nussbaum, Franz; Brands, Michael; Ziegelbauer, Karl; Mumberg, Dominik	MOLECULAR CANCER THERAPEUTICS	2020	19	1	6	6
A critical appraisal of biomarkers in prostate cancer	Narayan, Vikram M.	WORLD JOURNAL OF UROLOGY	2020	38	3	5	5
Clinical Outcomes in Cyclin-dependent Kinase 12 Mutant Advanced Prostate Cancer	Reimers, Melissa A.; Yip, Steven M.; Zhang, Li; Cieslik, Marcin; Dhawan, Mallika; Montgomery, Bruce; Wyatt, Alexander W.; Chi, Kim N.; Small, Eric J.; Chinnaiyan, Arul M.; Alva, Ajjai S.; Feng, Felix Y.; Chou, Jonathan	EUROPEAN UROLOGY	2020	77	3	5	5

Durable Response of Enzalutamide-resistant Prostate Cancer to Supraphysiological Testosterone Is Associated with a Multifaceted Growth Suppression and Impaired DNA Damage Response Transcriptomic Program in Patient-derived Xenografts	Lam, Hung-Ming; Nguyen, Holly M.; Labrecque, Mark P.; Brown, Lisha G.; Coleman, Ilsa M.; Gulati, Roman; Lakely, Bryce; Sondheim, Daniel; Chatterjee, Payel; Marck, Brett T.; Matsumoto, Alvin M.; Mostaghel, Elahe A.; Schweizer, Michael T.; Nelson, Peter S.; Corey, Eva	EUROPEAN UROLOGY	2020	77	2	5	5
Potential screening assays for individual radiation sensitivity and susceptibility and their current validation state	Gomolka, Maria; Blyth, Benjamin; Bourguignon, Michel; Badie, Christophe; Schmitz, Annette; Talbot, Christopher; Hoeschen, Christoph; Salomaa, Sisko	INTERNATIONAL JOURNAL OF RADIATION BIOLOGY	2020	96	3	5	2,5
Exosomes secreted by prostate cancer cells under hypoxia promote matrix metalloproteinases activity at pre-metastatic niches	Deep, Gagan; Jain, Anil; Kumar, Ashish; Agarwal, Chapla; Kim, Susy; Leevy, W. Matthew; Agarwal, Rajesh	MOLECULAR CARCINOGENESIS	2020	59	3	4	4
A low DNA methylation epigenotype in lung squamous cell carcinoma and its association with idiopathic pulmonary fibrosis and poorer prognosis	Hata, Atsushi; Nakajima, Takahiro; Matsusaka, Keisuke; Fukuyo, Masaki; Morimoto, Junichi; Yamamoto, Takayoshi; Sakairi, Yuichi; Rahmutulla, Bahityar; Ota, Satoshi; Wada, Hironobu; Suzuki, Hidemi; Matsubara, Hisahiro; Yoshino, Ichiro; Kaneda, Atsushi	INTERNATIONAL JOURNAL OF CANCER	2020	146	2	4	2
Prospective study to define the clinical utility and benefit of Decipher testing in men following prostatectomy	Marascio, Joseph; Spratt, Daniel E.; Zhang, Jingbin; Trabulsi, Edouard J.; Le, Tiffany; Sedzorme, Worlanyo Sosu; Beeler, Whitney H.; Davicioni, Elai; Dabbas, Bashar; Lin, Daniel W.; Gore, John L.; Bloom, Matthew; Mann, Mark; Mark, J. Ryan; Calvaresi, Anne; Godwin, James L.; McCue, Peter; Hurwitz, Mark D.; Kelly, W. Kevin; Lallas, Costas D.; Knudsen, Karen E.; Gomella, Leonard G.; Dicker, Adam P.; Den, Robert B.	PROSTATE CANCER AND PROSTATIC DISEASES	2020	23	2	3	3

Patients Resistant Against PSMA-Targeting alpha-Radiation Therapy Often Harbor Mutations in DNA Damage-Repair-Associated Genes	Kratochwil, Clemens; Giesel, Frederik L.; Heussel, Claus-Peter; Kazdal, Daniel; Endris, Volker; Nientiedt, Cathleen; Bruchertseifer, Frank; Kippenberger, Maximilian; Rathke, Hendrik; Leichsenring, Jonas; Hohenfellner, Markus; Morgenstern, Alfred; Haberkorn, Uwe; Duensing, Stefan; Stenzinger, Albrecht	JOURNAL OF NUCLEAR MEDICINE	2020	61	5	3	3
Exosomal microRNAs as liquid biopsy biomarkers in prostate cancer	Wang, Jingpu; Ni, Jie; Beretov, Julia; Thompson, James; Graham, Peter; Li, Yong	CRITICAL REVIEWS IN ONCOLOGY HEMATOLOGY	2020	145		3	3
LncRNA SNHG15: A new budding star in human cancers	Shuai, You; Ma, Zhonghua; Lu, Jianwei; Feng, Jifeng	CELL PROLIFERATION	2020	53	1	3	1,5
Long noncoding RNA FER1L4 acts as an oncogenic driver in human pan-cancer	You, Zilong; Ge, Anqi; Pang, Da; Zhao, Yashuang; Xu, Shouping	JOURNAL OF CELLULAR PHYSIOLOGY	2020	235	2	3	1,5
Prostate cancer cell-intrinsic interferon signaling regulates dormancy and metastatic outgrowth in bone	Owen, Katie L.; Gearing, Linden J.; Zanker, Damien J.; Brockwell, Natasha K.; Khoo, Weng Hua; Roden, Daniel L.; Cmero, Marek; Mangiola, Stefano; Hong, Matthew K.; Spurling, Alex J.; McDonald, Michelle; Chan, Chia-Ling; Pasam, Anupama; Lyons, Ruth J.; Duivenvoorden, Hendrika M.; Ryan, Andrew; Butler, Lisa M.; Mariadason, John M.; Phan, Tri Giang; Hayes, Vanessa M.; Sandhu, Shahneen; Swarbrick, Alexander; Corcoran, Niall M.; Hertzog, Paul J.; Croucher, Peter, I; Hovens, Chris; Parker, Belinda S.	EMBO REPORTS	2020	21	6	2	2
Molecular Biomarkers in Localized Prostate Cancer: ASCO Guideline	Eggener, Scott E.; Rumble, R. Bryan; Armstrong, Andrew J.; Morgan, Todd M.; Crispino, Tony; Cornford, Philip; van der Kwast, Theodorus; Grignon, David J.; Rai, Alex J.; Agarwal, Neeraj; Klein, Eric A.; Den, Robert B.; Beltran, Himisha	JOURNAL OF CLINICAL ONCOLOGY	2020	38	13	2	2

Intron retention is a hallmark and spliceosome represents a therapeutic vulnerability in aggressive prostate cancer	Zhang, Dingxiao; Hu, Qiang; Liu, Xiaozhuo; Ji, Yibing; Chao, Hsueh-Ping; Liu, Yan; Tracz, Amanda; Kirk, Jason; Buonamici, Silvia; Zhu, Ping; Wang, Jianmin; Liu, Song; Tang, Dean G.	NATURE COMMUNICATIONS	2020	11	1	2	2
Prostate cancer cell-intrinsic interferon signaling regulates dormancy and metastatic outgrowth in bone	Owen, Katie L.; Gearing, Linden J.; Zanker, Damien J.; Brockwell, Natasha K.; Khoo, Weng Hua; Roden, Daniel L.; Cmero, Marek; Mangiola, Stefano; Hong, Matthew K.; Spurling, Alex J.; McDonald, Michelle; Chan, Chia-Ling; Pasam, Anupama; Lyons, Ruth J.; Duivenvoorden, Hendrika M.; Ryan, Andrew; Butler, Lisa M.; Mariadason, John M.; Phan, Tri; Hayes, Vanessa M.; Sandhu, Shahneen; Swarbrick, Alexander; Corcoran, Niall M.; Hertzog, Paul J.; Croucher, Peter, I; Hovens, Chris; Parker, Belinda S.	EMBO REPORTS	2020			2	2
Appraising causal relationships of dietary, nutritional and physical-activity exposures with overall and aggressive prostate cancer: two-sample Mendelian-randomization study based on 79148 prostate-cancer cases and 61106 controls	Kazmi, Nabila; Haycock, Philip; Tsilidis, Konstantinos; Lynch, Brigid M.; Truong, Therese; Martin, Richard M.; Lewis, Sarah J.	INTERNATIONAL JOURNAL OF EPIDEMIOLOGY	2020	49	2	2	2
Single-sample landscape entropy reveals the imminent phase transition during disease progression	Liu, Rui; Chen, Pei; Chen, Luonan	BIOINFORMATICS	2020	36	5	2	2
Cell Stress Induced Stressome Release Including Damaged Membrane Vesicles and Extracellular HSP90 by Prostate Cancer Cells	Eguchi, Takanori; Sogawa, Chiharu; Ono, Kisho; Matsumoto, Masaki; Manh Tien Tran; Okusha, Yuka; Lang, Benjamin J.; Okamoto, Kuniaki; Calderwood, Stuart K.	CELLS	2020	9	3	2	2
Integrative molecular characterization of Chinese prostate cancer specimens	Lv, Shi-Dong; Wang, Hong-Yi; Yu, Xin-Pei; Zhai, Qi-Liang; Wu, Yao-Bin; Wei, Qiang; Huang, Wen-Hua	ASIAN JOURNAL OF ANDROLOGY	2020	22	2	2	2

Understanding aberrant RNA splicing to facilitate cancer diagnosis and therapy	Dong, Xuesen; Chen, Ruiqi	ONCOGENE	2020	39	11	2	2
Allergy, asthma, and the risk of breast and prostate cancer: a Mendelian randomization study	Jiang, Xia; Dimou, Niki L.; Zhu, Zhaozhong; Bonilla, Carolina; Lewis, Sarah J.; Lindstrom, Sara; Kraft, Peter; Tsilidis, Konstantinos K.; Martin, Richard M.	CANCER CAUSES & CONTROL	2020	31	3	2	2
Data Mining and Expression Analysis of Differential lncRNA ADAMTS9-AS1 in Prostate Cancer	Wan, Jiahui; Jiang, Shijun; Jiang, Ying; Ma, Wei; Wang, Xiuli; He, Zikang; Wang, Xiaojin; Cui, Rongjun	FRONTIERS IN GENETICS	2020	10		2	2
Divergent mutational processes distinguish hypoxic and normoxic tumours	Bhandari, Vinayak; Li, Constance H.; Bristow, Robert G.; Boutros, Paul C.	NATURE COMMUNICATION S	2020	11	1	2	2
HNF1B-mediated repression of SLUG is suppressed by EZH2 in aggressive prostate cancer	Wang, Jianqing; He, Chenxi; Gao, Peng; Wang, Siqing; Lv, Ruitu; Zhou, Huihui; Zhou, Qidong; Zhang, Ke; Sun, Jian; Fan, Caibin; Ding, Guanxiong; Lan, Fei	ONCOGENE	2020	39	6	2	2
Phenethyl Isothiocyanate Suppresses Stemness in the Chemo- and Radio-Resistant Triple-Negative Breast Cancer Cell Line MDA-MB-231/IR Via Downregulation of Metadherin	Nguyen, Yen Thi-Kim; Moon, Jeong Yong; Ediriweera, Meran Keshawa; Cho, Somi Kim	CANCERS	2020	12	2	2	2
Identification of a Circulating Amino Acid Signature in Frail Older Persons with Type 2 Diabetes Mellitus: Results from the Metabofrail Study	Calvani, Riccardo; Rodriguez-Manas, Leocadio; Picca, Anna; Marini, Federico; Biancolillo, Alessandra; Laosa, Olga; Pedraza, Laura; Gervasoni, Jacopo; Primiano, Aniello; Conta, Giorgia; Bourdel-Marchasson, Isabelle; Regueme, Sophie C.; Bernabei, Roberto; Marzetti, Emanuele; Sinclair, Alan J.; Gambassi, Giovanni	NUTRIENTS	2020	12	1	2	2

Development of Treatments for Localized Prostate Cancer in Patients Eligible for Active Surveillance: US Food and Drug Administration Oncology Center of Excellence Public Workshop	Weinstock, Chana; Suzman, Daniel; Kluetz, Paul; Baxley, John; Viviano, Charles; Ibrahim, Amna; Jarow, Jonathan; Sridhara, Raejshwari; Liu, Ke; Carroll, Peter; Eggener, Scott; Hu, Jim C.; Hussain, Maha; King, Martin; Klein, Eric; Kungel, Terry; Makarov, Danil; Pinto, Peter A.; Rini, Brian; Roach, Mack; Sandler, Howard; Schlegel, Peter N.; Song, Daniel; Goldberg, Kirsten; Pazdur, Richard; Beaver, Julia A.	JOURNAL OF UROLOGY	2020	203	1	2	2
HDR Prostate Brachytherapy	Crook, Juanita; Marban, Marina; Batchelar, Deidre	SEMINARS IN RADIATION ONCOLOGY	2020	30	1	2	2
Sulforaphane Bioavailability and Chemopreventive Activity in Men Presenting for Biopsy of the Prostate Gland: A Randomized Controlled Trial	Zhang, Zhenzhen; Garzotto, Mark; Davis, Edward W.; Mori, Motomi; Stoller, Wesley A.; Farris, Paige E.; Wong, Carmen P.; Beaver, Laura M.; Thomas, George, V; Williams, David E.; Dashwood, Roderick H.; Hendrix, David A.; Ho, Emily; Shannon, Jackilen	NUTRITION AND CANCER-AN INTERNATIONAL JOURNAL	2020	72	1	2	1
ATM Loss Confers Greater Sensitivity to ATR Inhibition Than PARP Inhibition in Prostate Cancer	Rafiei, Shahrzad; Fitzpatrick, Kenyon; Liu, David; Cai, Mu-Yan; Elmarakeby, Haitham A.; Park, Jihye; Ricker, Cora; Kochupurakkal, Bose S.; Choudhury, Atish D.; Hahn, William C.; Balk, Steven P.; Hwang, Justin H.; Van Allen, Eliezer M.; Mouw, Kent W.	CANCER RESEARCH	2020	80	11	1	1
The influence of hypoxia on the prostate cancer proteome	Ross, James A.; Vissers, Johannes P. C.; Nanda, Jyoti; Stewart, Grant D.; Husi, Holger; Habib, Fouad K.; Hammond, Dean E.; Gethings, Lee A.	CLINICAL CHEMISTRY AND LABORATORY MEDICINE	2020	58	6	1	1
Detection of novel mitochondrial mutations in cytochrome C oxidase subunit 1 (COX1) in patients with familial adenomatous polyposis (FAP)	Afkhami, E.; Heidari, M. M.; Khatami, M.; Ghadamyari, F.; Dianatpour, S.	CLINICAL & TRANSLATIONAL ONCOLOGY	2020	22	6	1	1

Proteomic Tissue-Based Classifier for Early Prediction of Prostate Cancer Progression	Gao, Yuqian; Wang, Yi-Ting; Chen, Yongmei; Wang, Hui; Young, Denise; Shi, Tujin; Song, Yingjie; Schepmoes, Athena A.; Kuo, Claire; Fillmore, Thomas L.; Qian, Wei-Jun; Smith, Richard D.; Srivastava, Sudhir; Kagan, Jacob; Dobi, Albert; Sesterhenn, Isabell A.; Rosner, Inger L.; Petrovics, Gyorgy; Rodland, Karin D.; Srivastava, Shiv; Cullen, Jennifer; Liu, Tao	CANCERS	2020	12	5	1	1
Rapid Prostate Cancer Noninvasive Biomarker Screening Using Segmented Flow Mass Spectrometry-Based Untargeted Metabolomics	Pinto, Frederico G.; Mahmud, Iqbal; Harmon, Taylor A.; Rubio, Vanessa Y.; Garrett, Timothy J.	JOURNAL OF PROTEOME RESEARCH	2020	19	5	1	1
Benefits and Risks of Primary Treatments for High -risk Localized and Locally Advanced Prostate Cancer: An International Multidisciplinary Systematic Review	Moris, Lisa; Cumberbatch, Marcus G.; Van den Broeck, Thomas; Gandaglia, Giorgio; Fossati, Nicola; Kelly, Brian; Pal, Raj; Briers, Erik; Cornford, Philip; De Santis, Maria; Fanti, Stefano; Gillessen, Silke; Grummet, Jeremy P.; Henry, Ann M.; Lam, Thomas B. L.; Lardas, Michael; Liew, Matthew; Mason, Malcolm D.; Omar, Muhammad Imran; Rouviere, Olivier; Schoots, Ivo G.; Tilki, Derya; van den Bergh, Roderick C. N.; van Der Kwast, Theodorus H.; van Der Poel, Henk G.; Willemse, Peter-Paul M.; Yuan, Cathy Y.; Konety, Badrinath; Dorff, Tanya; Jain, Suneil; Mottet, Nicolas; Wiegel, Thomas	EUROPEAN UROLOGY	2020	77	5	1	1
Metabolic Fingerprinting on Synthetic Alloys for Medulloblastoma Diagnosis and Radiotherapy Evaluation	Cao, Jing; Shi, Xuejiao; Gurav, Deepanjali D.; Huang, Lin; Su, Haiyang; Li, Keke; Niu, Jingyang; Zhang, Mengji; Wang, Qian; Jiang, Mawei; Qian, Kun	ADVANCED MATERIALS	2020	32	23	1	1

Ligand-based pharmacophore filtering, atom based 3D-QSAR, virtual screening and ADME studies for the discovery of potential ck2 inhibitors	Mohan, A.; Kirubakaran, R.; Parray, J. A.; Sivakumar, R.; Murugesh, E.; Govarthanan, M.	JOURNAL OF MOLECULAR STRUCTURE	2020	1205		1	1
Fold-Change-Specific Enrichment Analysis (FSEA): Quantification of Transcriptional Response Magnitude for Functional Gene Groups	Wiebe, Daniil S.; Omelyanchuk, Nadezhda A.; Mukhin, Aleksei M.; Grosse, Ivo; Lashin, Sergey A.; Zemlyanskaya, Elena, V; Mironova, Victoria V.	GENES	2020	11	4	1	1
Clinical and genomic insights into circulating tumor DNA-based alterations across the spectrum of metastatic hormone-sensitive and castrate-resistant prostate cancer	Kohli, Manish; Tan, Winston; Zheng, Tiantian; Wang, Amy; Montesinos, Carlos; Wong, Calven; Du, Pan; Jia, Shidong; Yadav, Siddhartha; Horvath, Lisa G.; Mahon, Kate L.; Kwan, Edmond M.; Fettke, Heidi; Yu, Jianjun; Azad, Arun A.	EBIOMEDICINE	2020	54		1	1
Circulating tumor DNA profile recognizes transformation to castration-resistant neuroendocrine prostate cancer	Beltran, Himisha; Romanel, Alessandro; Conteduca, Vincenza; Casiraghi, Nicola; Sigouros, Michael; Franceschini, Gian Marco; Orlando, Francesco; Fedrizzi, Tarcisio; Ku, Sheng-Yu; Dann, Emma; Alonso, Alicia; Mosquera, Juan Miguel; Sboner, Andrea; Xiang, Jenny; Elemento, Olivier; Nanus, David M.; Tagawa, Scott T.; Benelli, Matteo; Demichelis, Francesca	JOURNAL OF CLINICAL INVESTIGATION	2020	130	4	1	1
Comprehensive Analysis of AR Alterations in Circulating Tumor DNA from Patients with Advanced Prostate Cancer	Ledet, Elisa M.; Lilly, Michael B.; Sonpavde, Guru; Lin, Edwin; Nussenzveig, Roberto H.; Barata, Pedro C.; Yandell, Mark; Nagy, Rebecca J.; Kiedrowski, Lesli; Agarwal, Neeraj; Sartor, Oliver	ONCOLOGIST	2020	25	4	1	1

Significance of BRCA2 and RB1 Co-loss in Aggressive Prostate Cancer Progression	Chakraborty, Goutam; Armenia, Joshua; Mazzu, Ying Z.; Nandakumar, Subhiksha; Stopsack, Konrad H.; Atiq, Mohammad O.; Komura, Kazumasa; Jehane, Lina; Hirani, Rahim; Chadalavada, Kalyani; Yoshikawa, Yuki; Khan, Nabeela A.; Chen, Yu; Abida, Wassim; Mucci, Lorelei A.; Lee, Gwo-Shu Mary; Nanjangud, Gouri J.; Kantoff, Philip W.	CLINICAL CANCER RESEARCH	2020	26	8	1	1
Comprehensive analysis of biomarkers for prostate cancer based on weighted gene co-expression network analysis	Chen, Xuan; Wang, Jingyao; Peng, Xiqi; Liu, Kaihao; Zhang, Chunduo; Zeng, Xingzhen; Lai, Yongqing	MEDICINE	2020	99	14	1	1
Identification of Therapeutic Vulnerabilities in Small-cell Neuroendocrine Prostate Cancer	Corella, Alexandra N.; Ordonio, Ma Victoria Andrea Cabiliza; Coleman, Ilsa; Lucas, Jared M.; Kaipainen, Arja; Nguyen, Holly M.; Sondheim, Daniel; Brown, Lisha G.; True, Lawrence D.; Lee, John K.; MacPherson, David; Nghiem, Paul; Gulati, Roman; Morrissey, Colm; Corey, Eva; Nelson, Peter S.	CLINICAL CANCER RESEARCH	2020	26	7	1	1
Transcription levels and prognostic significance of the NFI family members in human cancers	Li, Yuexian; Sun, Cheng; Tan, Yonggang; Li, Lin; Zhang, Heying; Liang, Yusi; Zeng, Juan; Zou, Huawei	PEERJ	2020	8		1	1
Plasma cell-free DNA-based predictors of response to abiraterone acetate/prednisone and prognostic factors in metastatic castration-resistant prostate cancer	Du, Meijun; Tian, Yijun; Tan, Winston; Wang, Liewei; Wang, Liguo; Kilari, Deepak; Huang, Chiang-Ching; Wang, Liang; Kohli, Manish	PROSTATE CANCER AND PROSTATIC DISEASES	2020			1	1
Cys(2)His(2) Zinc Finger Methyl-CpG Binding Proteins: Getting a Handle on Methylated DNA	Hodges, Amelia J.; Hudson, Nicholas O.; Buck-Koehntop, Bethany A.	JOURNAL OF MOLECULAR BIOLOGY	2020	432	6	1	1
Prediction of tumor location in prostate cancer tissue using a machine learning system on gene expression data	Hamzeh, Osama; Alkhateeb, Abedalrhman; Zheng, Julia; Kandalam, Srinath; Rueda, Luis	BMC BIOINFORMATICS	2020	21		1	1

miR-221-3p Regulates VEGFR2 Expression in High-Risk Prostate Cancer and Represents an Escape Mechanism from Sunitinib In Vitro	Krebs, Markus; Solimando, Antonio Giovanni; Kalogirou, Charis; Marquardt, Andre; Frank, Torsten; Sokolakis, Ioannis; Hatzichristodoulou, Georgios; Kneitz, Susanne; Bargou, Ralf; Kuebler, Hubert; Schilling, Bastian; Spahn, Martin; Kneitz, Burkhard	JOURNAL OF CLINICAL MEDICINE	2020	9	3	1	1
Preanalytical Variables for the Genomic Assessment of the Cellular and Acellular Fractions of the Liquid Biopsy in a Cohort of Breast Cancer Patients	Shishido, Stephanie N.; Welter, Lisa; Rodriguez-Lee, Mariam; Kolatkar, Anand; Xu, Liya; Ruiz, Carmen; Gerdtsson, Anna S.; Restrepo-Vassalli, Sara; Carlsson, Anders; Larsen, Joe; Greenspan, Emily J.; Hwang, E. Shelley; Waitman, Kathryn R.; Nieva, Jorge; Bethel, Kelly; Hicks, James; Kuhn, Peter	JOURNAL OF MOLECULAR DIAGNOSTICS	2020	22	3	1	1
Identification of PTPRR and JAG1 as key genes in castration-resistant prostate cancer by integrated bioinformatics methods	Wang, Ji-li; Wang, Yan; Ren, Guo-ping	JOURNAL OF ZHEJIANG UNIVERSITY-SCIENCE B	2020	21	3	1	1
Clinical Utility of a Genomic Classifier in Men Undergoing Radical Prostatectomy: The PRO-IMPACT Trial	Gore, John L.; du Plessis, Marguerite; Zhang, Jingbin; Dai, Darlene; Thompson, Darby J. S.; Karsh, Lawrence; Lane, Brian; Franks, Michael; Chen, David Y. T.; Bianco, Fernando J., Jr.; Brown, Gordon; Clark, William; Kibel, Adam S.; Kim, Hyung; Lowrance, William; Manoharan, Murugesan; Maroni, Paul; Perrapato, Scott; Sieber, Paul; Trabulsi, Edouard J.; Waterhouse, Robert; Spratt, Daniel E.; Davicioni, Elai; Lotan, Yair; Lin, Daniel W.	PRACTICAL RADIATION ONCOLOGY	2020	10	2	1	1
Ethnic disparities among men with prostate cancer undergoing germline testing	Kwon, Daniel Hyuck-Min; Borno, Hala T.; Cheng, Heather H.; Zhou, Alicia Yiran; Small, Eric Jay	UROLOGIC ONCOLOGY-SEMINARS AND ORIGINAL INVESTIGATIONS	2020	38	3	1	1

Why the UK Should Consider Gene Expression Testing in Prostate Cancer	Dess, R. T.; Spratt, D. E.	CLINICAL ONCOLOGY	2020	32	3	1	1
Cancer transcriptomic profiling from rapidly enriched circulating tumor cells	Morrison, Gareth J.; Cunha, Alexander T.; Jojo, Nita; Xu, Yucheng; Xu, Yili; Kwok, Eric; Robinson, Peggy; Dorff, Tanya; Quinn, David; Carpten, John; Manojlovic, Zarko; Goldkorn, Amir	INTERNATIONAL JOURNAL OF CANCER	2020	146	10	1	1
Can epigenetic and inflammatory biomarkers identify clinically aggressive prostate cancer?	Santos, Pedro Bargao; Patel, Hitendra; Henrique, Rui; Felix, Ana	WORLD JOURNAL OF CLINICAL ONCOLOGY	2020	11	2	1	1
TMEFF2 is a novel prognosis signature and target for endometrial carcinoma	Gao, Lingling; Nie, Xin; Zheng, Mingjun; Li, Xiao; Guo, Qian; Liu, Juanjuan; Liu, Qing; Hao, Yingying; Lin, Bei	LIFE SCIENCES	2020	243		1	1
Establishment and Analysis of Three-Dimensional (3D) Organoids Derived from Patient Prostate Cancer Bone Metastasis Specimens and their Xenografts	Lee, Sanghee; Burner, Danielle N.; Mendoza, Theresa R.; Muldong, Michelle T.; Arreola, Catalina; Wu, Christina N.; Cacalano, Nicholas A.; Kulidjian, Anna A.; Kane, Christopher J.; Jamieson, Christina A. M.	JOVE-JOURNAL OF VISUALIZED EXPERIMENTS	2020		156	1	1
A NF-kappa B-Activin A signaling axis enhances prostate cancer metastasis	Chen, Lanpeng; De Menna, Marta; Groenewoud, Arwin; Thalmann, George N.; Kruithof-de Julio, Marianna; Snaar-Jagalska, B. Ewa	ONCOGENE	2020	39	8	1	1
DeepCOP: deep learning-based approach to predict gene regulating effects of small molecules	Woo, Godwin; Fernandez, Michael; Hsing, Michael; Lack, Nathan A.; Cavga, Ayse Derya; Cherkasov, Artem	BIOINFORMATICS	2020	36	3	1	1
Activation of beta-Catenin Cooperates with Loss of Pten to Drive AR-Independent Castration-Resistant Prostate Cancer	Patel, Rachana; Brzezinska, Elspeth A.; Repiscak, Peter; Ahmad, Imran; Mui, Ernest; Gao, Meiling; Blomme, Arnaud; Harle, Victoria; Tan, Ee Hong; Malviya, Gaurav; Mrowinska, Agata; Loveridge, Carolyn J.; Rushworth, Linda K.; Edwards, Joanne; Ntala, Chara; Nixon, Colin; Hedley, Ann; Mackay, Gillian; Tardito, Saverio; Sansom, Owen J.; Leung, Hing Y.	CANCER RESEARCH	2020	80	3	1	1

Advancing Biomarker Development Through Convergent Engagement: Summary Report of the 2nd International Danube Symposium on Biomarker Development, Molecular Imaging and Applied Diagnostics; March 14-16, 2018; Vienna, Austria	Lim, M. S.; Beyer, Thomas; Babayan, A.; Bergmann, M.; Brehme, M.; Buyx, A.; Czernin, J.; Egger, G.; Elenitoba-Johnson, K. S. J.; Gueckel, B.; Jacan, A.; Haslacher, H.; Hicks, R. J.; Kenner, L.; Langanke, M.; Mitterhauser, M.; Pichler, B. J.; Salih, H. R.; Schibli, R.; Schulz, S.; Simecek, J.; Simon, J.; Soares, M. O.; Stelzl, U.; Wadsak, W.; Zatloukal, K.; Zeitlinger, M.; Hacker, M.	MOLECULAR IMAGING AND BIOLOGY	2020	22	1	1	1
NMR-based metabolomics analysis identifies discriminatory metabolic disturbances in tissue and biofluid samples for progressive prostate cancer	Zheng, Hong; Dong, Baijun; Ning, Jie; Shao, Xiaoguang; Zhao, Liangcai; Jiang, Qiaoying; Ji, Hui; Cai, Aimin; Xue, Wei; Gao, Hongchang	CLINICA CHIMICA ACTA	2020	501		1	1
Improving diagnosis of genitourinary cancers: Biomarker discovery strategies through mass spectrometry-based metabolomics	Manzi, Malena; Riquelme, Gabriel; Zabalegui, Nicolas; Eugenia Monge, Maria	JOURNAL OF PHARMACEUTICAL AND BIOMEDICAL ANALYSIS	2020	178		1	1
Genetic Testing in Prostate Cancer	Sokolova, Alexandra O.; Cheng, Heather H.	CURRENT ONCOLOGY REPORTS	2020	22	1	1	1
Multi-omic serum biomarkers for prognosis of disease progression in prostate cancer	Kiebish, Michael A.; Cullen, Jennifer; Mishra, Prachi; Ali, Amina; Milliman, Eric; Rodrigues, Leonardo O.; Chen, Emily Y.; Tolstikov, Vladimir; Zhang, Lixia; Panagopoulos, Kiki; Shah, Punit; Chen, Yongmei; Petrovics, Gyorgy; Rosner, Inger L.; Sesterhenn, Isabell A.; McLeod, David G.; Granger, Elder; Sarangarajan, Rangaprasad; Akmaev, Viatcheslav; Srinivasan, Alagarsamy; Srivastava, Shiv; Narain, Niven R.; Dobi, Albert	JOURNAL OF TRANSLATIONAL MEDICINE	2020	18	1	1	1

Cyclic Multiplexed-Immunofluorescence (cmIF), a Highly Multiplexed Method for Single-Cell Analysis	Eng, Jennifer; Thibault, Guillaume; Luoh, Shiuh-Wen; Gray, Joe W.; Chang, Young Hwan; Chin, Koei	BIOMARKERS FOR IMMUNOTHERAPY OF CANCER: METHODS AND PROTOCOLS	2020	2055		1	1
Differential Characteristics of HMGB2 Versus HMGB1 and their Perspectives in Ovary and Prostate Cancer	Camara-Quilez, Maria; Barreiro-Alonso, Aida; Rodriguez-Bemonte, Esther; Quindos-Varela, Maria; Esperanza Cerdan, M.; Lamas-Maceiras, Monica	CURRENT MEDICINAL CHEMISTRY	2020	27	20	1	1
150 years of the periodic table: New medicines and diagnostic agents	Imberti, Cinzia; Sadler, Peter J.	MEDICINAL CHEMISTRY	2020	75		1	1
Urinary prostate-specific antigen and microseminoprotein-beta levels in men with and without prostate cancer: A prospective cohort study	Shrivastava, Prashant; Garg, Harshit; Bhat, Madhusudan; Dinda, Amit; Kumar, Rajeev	INDIAN JOURNAL OF UROLOGY	2020	36	1	1	1
Urothelial Carcinoma Detection Based on Copy Number Profiles of Urinary Cell-Free DNA by Shallow Whole-Genome Sequencing	Ge, Guangzhe; Peng, Ding; Guan, Bao; Zhou, Yuanyuan; Gong, Yanqing; Shi, Yue; Hao, Xueyu; Xu, Zhengzheng; Qi, Jie; Lu, Huan; Zhang, Xiaoyun; Zhan, Yonghao; Li, Yifan; Wu, Yucai; Ding, Guangpu; Shen, Qi; He, Qun; Li, Xuesong; Zhou, Liqun; Ci, Weimin	CLINICAL CHEMISTRY	2020	66	1	1	1
Unravelling the proteomic landscape of extracellular vesicles in prostate cancer by density-based fractionation of urine	Dhondt, Bert; Geeurickx, Edward; Tulkens, Joeri; Van Deun, Jan; Vergauwen, Glenn; Lippens, Lien; Miinalainen, Ilkka; Rappu, Pekka; Heino, Jyrki; Ost, Piet; Lumen, Nicolaas; De Wever, Olivier; Hendrix, An	JOURNAL OF EXTRACELLULAR VESICLES	2020	9	1	1	1
The Panel of 12 Cell-Free MicroRNAs as Potential Biomarkers in Prostate Neoplasms	Konoshenko, Maria Yu; Lekchnov, Evgeniy A.; Bryzgunova, Olga E.; Zaporozhchenko, Ivan A.; Yarmoschuk, Sergey, V; Pashkovskaya, Oksana A.; Pak, Svetlana, V; Laktionov, Pavel P.	DIAGNOSTICS	2020	10	1	1	1
Genome-wide analysis reveals the emerging roles of long non-coding RNAs in cancer (Review)	Ren, Xiaoxia	ONCOLOGY LETTERS	2020	19	1	1	1

Network Pharmacology-Based Study on the Molecular Biological Mechanism of Action for Compound Kushen Injection in Anti-Cancer Effect	He, Ruirong; Ou, Shuya; Chen, Shichun; Ding, Shaobo	MEDICAL SCIENCE MONITOR	2020	26		1	1
The Genomic and Molecular Pathology of Prostate Cancer: Clinical Implications for Diagnosis, Prognosis, and Therapy	Faisal, Farzana A.; Lotan, Tamara L.	ADVANCES IN ANATOMIC PATHOLOGY	2020	27	1	1	1
Weighted gene co-expression network analysis identifies CCNA2 as a treatment target of prostate cancer through inhibiting cell cycle	Yang, Rui; Du, Yang; Wang, Lei; Chen, Zhiyuan; Liu, Xiuheng	JOURNAL OF CANCER	2020	11	5	1	1
Evaluation of statistical techniques to normalize mass spectrometry-based urinary metabolomics data	Cook, Tyler; Ma, Yinfa; Gamagedara, Sanjeewa	JOURNAL OF PHARMACEUTICAL AND BIOMEDICAL ANALYSIS	2020	177		1	1
Noncanonical Wnt as a prognostic marker in prostate cancer: you can't always get what you Wnt	Fisher, Rebecca R.; Pleskow, Haley M.; Bedingfield, Kathleen; Miyamoto, David T.	EXPERT REVIEW OF MOLECULAR DIAGNOSTICS	2020	20	2	1	0,5
Prostate cancer research: The next generation; report from the 2019 Coffey-Holden Prostate Cancer Academy Meeting	Miyahira, Andrea K.; Sharp, Adam; Ellis, Leigh; Jones, Jennifer; Kaochar, Salma; Larman, H. Benjamin; Quigley, David A.; Ye, Huihui; Simons, Jonathan W.; Pienta, Kenneth J.; Soule, Howard R.	PROSTATE	2020	80	2	1	0,5

Expression of CCCTC-binding factor (CTCF) is linked to poor prognosis in prostate cancer	Hoeflmayer, Doris; Steinhoff, Amelie; Hube-Magg, Claudia; Kluth, Martina; Simon, Ronald; Burandt, Eike; Tsourlakis, Maria Christina; Minner, Sarah; Sauter, Guido; Buescheck, Franziska; Wilczak, Waldemar; Steurer, Stefan; Huland, Hartwig; Graefen, Markus; Haese, Alexander; Heinzer, Hans; Schlomm, Thorsten; Jacobsen, Frank; Hinsch, Andrea; Poos, Alexandra M.; Oswald, Marcus; Rippe, Karsten; Koenig, Rainer; Schroeder, Cornelia	MOLECULAR ONCOLOGY	2020	14	1	1	0,5
Discordant and heterogeneous clinically relevant genomic alterations in circulating tumor cells vs plasma DNA from men with metastatic castration resistant prostate cancer	Gupta, Santosh; Hovelson, Daniel H.; Kemeny, Gabor; Halabi, Susan; Foo, Wen-Chi; Anand, Monika; Somarelli, Jason A.; Tomlins, Scott A.; Antonarakis, Emmanuel S.; Luo, Jun; Dittamore, Ryan, V; George, Daniel J.; Rothwell, Colin; Nanus, David M.; Armstrong, Andrew J.; Gregory, Simon G.	GENES CHROMOSOMES & CANCER	2020	59	4	1	0,5
The molecular function of kallikrein-related peptidase 14 demonstrates a key modulatory role in advanced prostate cancer	Kryza, Thomas; Bock, Nathalie; Lovell, Scott; Rockstroh, Anja; Lehman, Melanie L.; Lesner, Adam; Panchadsaram, Janaththani; Silva, Lakmali Munasinghage; Srinivasan, Srilakshmi; Snell, Cameron E.; Williams, Elizabeth D.; Fazli, Ladan; Gleave, Martin; Batra, Jyotsna; Nelson, Colleen; Tate, Edward W.; Harris, Jonathan; Hooper, John D.; Clements, Judith A.	MOLECULAR ONCOLOGY	2020	14	1	1	0,5
Correlation between cribriform/intraductal prostatic adenocarcinoma and percent Gleason pattern 4 to a 22-gene genomic classifier	Taylor, Alexander S.; Morgan, Todd M.; Wallington, David G.; Chinnaiyan, Arul M.; Spratt, Daniel E.; Mehra, Rohit	PROSTATE	2020	80	2	1	0,5

Mithramycin suppresses DNA damage repair via targeting androgen receptor in prostate cancer	Wang, Shan; Gilbreath, Collin; Kollipara, Rahul K.; Sonavane, Rajni; Huo, Xiaofang; Yenerall, Paul; Das, Amit; Ma, Shihong; Raj, Ganesh, V; Kittler, Ralf	CANCER LETTERS	2020	488		0	0
Paper-based ITP technology: An application to specific cancer-derived exosome detection and analysis	Guo, Shuang; Xu, Jie; Estell, Alexander Pettit; Ivory, Cornelius F.; Du, Dan; Lin, Yuehe; Dong, Wen-Ji	BIOSENSORS & BIOELECTRONICS	2020	164		0	0
Deregulated PTEN/PI3K/AKT/mTOR signaling in prostate cancer: Still a potential druggable target?	Braglia, Luca; Zavatti, Manuela; Vinceti, Marco; Martelli, Alberto M.; Marmiroli, Sandra	BIOCHIMICA ET BIOPHYSICA ACTA-MOLECULAR CELL RESEARCH	2020	1867	9	0	0
Identification of a natural compound, sesamin, as a novel TRPM8 antagonist with inhibitory effects on prostate adenocarcinoma	Sui, Yutong; Li, Shiyou; Zhao, Yahui; Liu, Qing; Qiao, Yanjiang; Feng, Li; Li, Sheng	FITOTERAPIA	2020	145		0	0
Quantitative and qualitative impairments in dendritic cell subsets of patients with ovarian or prostate cancer	Mastelic-Gavillet, Beatris; Sarivalasis, Apostolos; Lozano, Leyder Elena; Wyss, Tania; Inoges, Susana; de Vries, Ingrid Jolanda Monique; Dartiguenave, Florence; Jichlinski, Patrice; Derre, Laurent; Coukos, George; Melero, Ignacio; Harari, Alexandre; Romero, Pedro; Vigano, Selena; Kandalaft, Lana Elias	EUROPEAN JOURNAL OF CANCER	2020	135		0	0
An in vivo functional genomics screen of nuclear receptors and their co-regulators identifies FOXA1 as an essential gene in lung tumorigenesis	Hight, Suzie K.; Mootz, Allison; Kollipara, Rahul K.; McMillan, Elizabeth; Yenerall, Paul; Otaki, Yoichi; Li, Long-Shan; Avila, Kimberley; Peyton, Michael; Rodriguez-Canales, Jaime; Mino, Barbara; Villalobos, Pamela; Girard, Luc; Dospoy, Patrick; Larsen, Jill; White, Michael A.; Heymach, John V.; Wistuba, Ignacio I.; Kittler, Ralf; Minna, John D.	NEOPLASIA	2020	22	8	0	0

Ampholine immobilized polymer microspheres for increasing coverage of human urinary proteome	Deng, Nan; Chen, Yuanbo; Liang, Zhen; Bian, Yangyang; Wang, Bing; Sui, Zhigang; Zhang, Xiaodan; Yang, Kaiguang; Zhang, Lihua; Zhang, Yukui	TALANTA	2020	215		0	0
Identification of six novel alternative transcripts of the human kallikrein-related peptidase 15 (KLK15), using 3 ' RACE and high-throughput sequencing	Adamopoulos, Panagiotis G.; Koukouzeli, Fotini E.; Kontos, Christos K.; Scorilas, Andreas	GENE	2020	749		0	0
Melatonin, an ubiquitous metabolic regulator: functions, mechanisms and effects on circadian disruption and degenerative diseases	Socaciu, Andreea Iulia; Ionut, Razvan; Socaciu, Mihai Adrian; Ungur, Andreea Petra; Barsan, Maria; Chiorean, Angelica; Socaciu, Carmen; Rajnoveanu, Armand Gabriel	REVIEWS IN ENDOCRINE & METABOLIC DISORDERS	2020			0	0
Zonal regulation of collagen-type proteins and posttranslational modifications in prostatic benign and cancer tissues by imaging mass spectrometry	Angel, Peggi M.; Spruill, Laura; Jefferson, Melanie; Bethard, Jennifer R.; Ball, Lauren E.; Hughes-Halbert, Chanita; Drake, Richard R.	PROSTATE	2020			0	0
Identifying and treatingROBO1(-ve)/DOCK1(+ve)prostate cancer: An aggressive cancer subtype prevalent in African American patients	Ferrari, Marina G.; Ganaie, Arsheed A.; Shabenah, Ashraf; Mansini, Adrian P.; Wang, Li; Murugan, Paari; Davicioni, Elai; Wang, Jinhua; Deng, Yibin; Hoeppner, Luke H.; Warlick, Christopher A.; Konety, Badrinath R.; Saleem, Mohammad	PROSTATE	2020			0	0

Secreted Frizzled-Related Protein 4 (SFRP4) Is an Independent Prognostic Marker in Prostate Cancers LackingTMPRSS2: ERGFusions	Bernreuther, Christian; Daghigh, Ferdous; Moeller, Katharina; Hube-Magg, Claudia; Lennartz, Maximilian; Lutz, Florian; Rico, Sebastian Dwertmann; Fraune, Christoph; Dum, David; Luebke, Andreas M.; Eichenauer, Till; Moeller-Koop, Christina; Schlomm, Thorsten; Wittmer, Corinna; Huland, Hartwig; Heinzer, Hans; Graefen, Markus; Haese, Alexander; Burandt, Eike; Tsourlakis, Maria Christina; Clauditz, Till S.; Hoeflmayer, Doris; Izbicki, Jakob R.; Simon, Ronald; Sauter, Guido; Minner, Sarah; Steurer, Stefan; Meiners, Jan	PATHOLOGY & ONCOLOGY RESEARCH	2020			0	0
The CHK1 inhibitor MU380 significantly increases the sensitivity of human docetaxel-resistant prostate cancer cells to gemcitabine through the induction of mitotic catastrophe	Drapela, Stanislav; Khirsariya, Prashant; van Weerden, Wytske M.; Fedr, Radek; Suchankova, Tereza; Buzova, Diana; Cerveny, Jan; Hampl, Ales; Puhr, Martin; Watson, William R.; Culig, Zoran; Krejci, Lumir; Paruch, Kamil; Soucek, Karel	MOLECULAR ONCOLOGY	2020			0	0
Microtubule Engagement with Taxane Is Altered in Taxane-Resistant Gastric Cancer	Galletti, Giuseppe; Zhang, Chao; Gjyrezi, Ada; Cleveland, Kyle; Zhang, Jiaren; Powell, Sarah; Thakkar, Prashant, V; Betel, Doron; Shah, Manish A.; Giannakakou, Paraskevi	CLINICAL CANCER RESEARCH	2020	26	14	0	0
Associations of IL6 rs1800795, BLK rs13277113, TIMP3 rs9621532, IL1RL1 rs1041973 and IL1RAP rs4624606 single gene polymorphisms with laryngeal squamous cell carcinoma	Pasvenskaite, Agne; Vilkeviciute, Alvita; Liutkeviciene, Rasa; Gedvilaite, Greta; Liutkevicius, Vykintas; Uloza, Virgilijus	GENE	2020	747		0	0
Targeting CPT1B as a potential therapeutic strategy in castration-resistant and enzalutamide-resistant prostate cancer	Abudurexiti, Mierxiati; Zhu, Wenkai; Wang, Yuchen; Wang, Jun; Xu, Wenhao; Huang, Yongqiang; Zhu, Yao; Shi, Guohai; Zhang, Hailiang; Zhu, Yiping; Shen, Yijun; Dai, Bo; Wan, Fangning; Lin, Guowen; Ye, Dingwei	PROSTATE	2020	80	12	0	0

Prostate cancer-derived holoclones: a novel and effective model for evaluating cancer stemness	Flynn, Louise; Barr, Martin P.; Baird, Anne-Marie; Smyth, Paul; Casey, Orla M.; Blackshields, Gordon; Greene, John; Pennington, Stephen R.; Hams, Emily; Fallon, Padraic G.; O'Leary, John; Sheils, Orla; Finn, Stephen P.	SCIENTIFIC REPORTS	2020	10	1	0	0
Acute Promyelocytic Leukemia After Radium-223 Exposure for Prostate Cancer in a Chemotherapy-Naive Patient	Perrone, Salvatore; Ortu La Barbera, Elettra; Ottone, Tiziana; Capriata, Marcello; Passucci, Mauro; Filippi, Luca; Bagni, Oreste; Voso, Maria Teresa; Cimino, Giuseppe	NUCLEAR MEDICINE AND MOLECULAR IMAGING	2020			0	0
Systematic profiling of alternative splicing signature reveals prognostic predictor for prostate cancer	Zhao, Jiyu; Chang, Luchen; Gu, Xianen; Liu, Jia; Sun, Bei; Wei, Xi	CANCER SCIENCE	2020			0	0
In silicodocking of phytocompounds to identify potent inhibitors of signaling pathways involved in prostate cancer	Jonnalagadda, Bhavana; Arockiasamy, Sumathy; Vetrivel, Umashankar; Abhinand, P. A.	JOURNAL OF BIOMOLECULAR STRUCTURE & DYNAMICS	2020			0	0
An organoid-based drug screening identified a menin-MLL inhibitor for endometrial cancer through regulating the HIF pathway	Chen, Jingyao; Zhao, Lei; Peng, Hongling; Dai, Siqi; Quan, Yuan; Wang, Manli; Wang, Jian; Bi, Zhanying; Zheng, Ying; Zhou, Shengtao; Liu, Yu; Chen, Chong; Na, Feifei	CANCER GENE THERAPY	2020			0	0
Decoding the evolutionary response to prostate cancer therapy by plasma genome sequencing	Ramesh, Naveen; Sei, Emi; Tsai, Pei Ching; Bai, Shanshan; Zhao, Yuehui; Troncoso, Patricia; Corn, Paul G.; Logothetis, Christopher; Zurita, Amado J.; Navin, Nicholas E.	GENOME BIOLOGY	2020	21	1	0	0
Cancer-driven IgG promotes the development of prostate cancer though the SOX2-CIgG pathway	Qin, Caipeng; Sheng, Zhengzuo; Huang, Xinmei; Tang, Jingshu; Liu, Yang; Xu, Tao; Qiu, Xiaoyan	PROSTATE	2020			0	0

Reduced KLK2 expression is a strong and independent predictor of poor prognosis in ERG-negative prostate cancer	Bonk, Sarah; Kluth, Martina; Jansen, Kristina; Hube-Magg, Claudia; Makrypidi-Fraune, Georgia; Hoeflmayer, Doris; Weidemann, Soeren; Moeller, Katharina; Uhlig, Ria; Buescheck, Franziska; Luebke, Andreas M.; Burandt, Eike; Clauditz, Till S.; Steurer, Stefan; Schlomm, Thorsten; Huland, Hartwig; Heinzer, Hans; Sauter, Guido; Simon, Ronald; Dum, David	PROSTATE	2020			0	0
Patterns of stemness-associated markers in the development of castration-resistant prostate cancer	Federer-Gsponer, Joel R.; Mueller, David C.; Zellweger, Tobias; Eggimann, Maurice; Marston, Katharina; Ruiz, Christian; Seifert, Hans-Helge; Rentsch, Cyrill A.; Bubendorf, Lukas; Le Magnen, Clementine	PROSTATE	2020			0	0
Proteomics Analysis of Formalin Fixed Paraffin Embedded Tissues in the Investigation of Prostate Cancer	Mantsiou, Anna; Makridakis, Manousos; Fasoulakis, Konstantinos; Katafigiotis, Ioannis; Constantinides, Constantinos A.; Zoidakis, Jerome; Roubelakis, Maria G.; Vlahou, Antonia; Lygirou, Vasiliki	JOURNAL OF PROTEOME RESEARCH	2020	19	7	0	0
HILIC-MRM-MS for Linkage-Specific Separation of Sialylated Glycopeptides to Quantify Prostate-Specific Antigen Proteoforms	van der Burgt, Yuri E. M.; Siliakus, Kasper M.; Cobbaert, Christa M.; Ruhaak, L. Renee	JOURNAL OF PROTEOME RESEARCH	2020	19	7	0	0

A Custom Genotyping Array Reveals Population-Level Heterogeneity for the Genetic Risks of Prostate Cancer and Other Cancers in Africa	Harlemon, Maxine; Ajayi, Olabode; Kachambwa, Paidamoyo; Kim, Michelle S.; Simonti, Corinne N.; Quiver, Melanie H.; Petersen, Desiree C.; Mittal, Anuradha; Fernandez, Pedro W.; Hsing, Ann W.; Baichoo, Shakuntala; Agalliu, Ilir; Jalloh, Mohamed; Gueye, Serigne M.; Snyper, Nana Yaa F.; Adusei, Ben; Mensah, James E.; Abrahams, Afua O. D.; Adebiyi, Akindele O.; Orunmuyi, Akin T.; Aisuodionoe-Shadrach, Oseremen I.; Nwegbu, Maxwell M.; Joffe, Maureen; Chen, Wenlong C.; Irusen, Hayley; Neugut, Alfred I.; Quintana, Yuri; Seutloali, Moleboheng; Fadipe, Mayowa B.; Warren, Christopher; Woehrmann, Marcos H.; Zhang, Peng; Ongaco, Chrissie M.; Mawhinney, Michelle; McBride, Jo; Andrews, Caroline V.; Adams, Marcia; Pugh, Elizabeth; Rebbeck, Timothy R.; Petersen, Lindsay N.; Lachance, Joseph	CANCER RESEARCH	2020	80	13	0	0
Oncogenic Genomic Alterations, Clinical Phenotypes, and Outcomes in Metastatic Castration-Sensitive for Prostate Cancer	Stopsack, Konrad H.; Nandakumar, Subhiksha; Wibmer, Andreas G.; Haywood, Samuel; Weg, Emily S.; Barnett, Ethan S.; Kim, Chloe J.; Carbone, Emily A.; Vasselman, Samantha E.; Nguyen, Bastien; Hullings, Melanie A.; Scher, Howard I.; Morris, Michael J.; Solit, David B.; Schultz, Nikolaus; Kantoff, Philip W.; Abida, Wassim	CLINICAL CANCER RESEARCH	2020	26	13	0	0
Transcriptomic analysis reveals that heat shock protein 90 alpha is a potential diagnostic and prognostic biomarker for cancer	Chen, Wei; Li, Guanghua; Peng, Jianjun; Dai, Weigang; Su, Qiao; He, Yulong	EUROPEAN JOURNAL OF CANCER PREVENTION	2020	29	4	0	0

A Network Pharmacology Approach to Explore the Potential Mechanisms of Huangqin-Baishao Herb Pair in Treatment of Cancer	Xu, Tian; Wang, Qingguo; Liu, Min	MEDICAL SCIENCE MONITOR	2020	26		0	0
p53/PGC-1 alpha-mediated mitochondrial dysfunction promotes PC3 prostate cancer cell apoptosis	Li, Jiuling; Li, Yany; Chen, Lanlan; Yu, Bingbing; Xue, Yanan; Guo, Rui; Su, Jing; Liu, Yanan; Sun, Liankun	MOLECULAR MEDICINE REPORTS	2020	22	1	0	0
Novel approach to therapeutic targeting of castration-resistant prostate cancer	Shankar, Eswar; Franco, Daniel; Iqbal, Omair; El-Hayek, Victoria; Gupta, Sanjay	MEDICAL HYPOTHESES	2020	140		0	0
Anti-prostate cancer activity of a nanoformulation of the spleen tyrosine kinase (SYK) inhibitor C61	Sahin, Taha Koray; Aktepe, Oktay Halit; Uckun, Fatih Mehmet; Yalcin, Suayib	ANTI-CANCER DRUGS	2020	31	6	0	0
Roles of lncRNAs in cancer: Focusing on angiogenesis	Jin, Ke-Tao; Yao, Jia-Yu; Fang, Xing-Liang; Di, Hua; Ma, Ying-Yu	LIFE SCIENCES	2020	252		0	0
An overview of current and emerging diagnostic, staging and prognostic markers for prostate cancer	Broenimann, Stephan; Pradere, Benjamin; Karakiewicz, Pierre; Abufaraj, Mohammad; Briganti, Alberto; Shariat, Shahrokh F.	EXPERT REVIEW OF MOLECULAR DIAGNOSTICS	2020			0	0
Prostate cancer management: long-term beliefs, epidemic developments in the early twenty-first century and 3PM dimensional solutions	Kucera, Radek; Pecen, Ladislav; Topolcan, Ondrej; Dahal, Anshu Raj; Costigliola, Vincenzo; Giordano, Frank A.; Golubnitschaja, Olga	EPMA JOURNAL	2020			0	0
Cytochrome P450 1B1 polymorphism drives cancer cell stemness and patient outcome in head-and-neck carcinoma	Le Morvan, Valerie; Richard, Elodie; Cadars, Maud; Fessart, Delphine; Broca-Brisson, Lea; Auzanneau, Celine; Pasquies, Alban; Modesto, Anouchka; Lusque, Amelie; Mathoulin-Pelissier, Simone; Lansiaux, Amelie; Robert, Jacques	BRITISH JOURNAL OF CANCER	2020			0	0
Blood-derived DNA methylation predictors of mortality discriminate tumor and healthy tissue in multiple organs	Zhang, Yan; Bewerunge-Hudler, Melanie; Schick, Matthias; Burwinkel, Barbara; Herpel, Esther; Hoffmeister, Michael; Brenner, Hermann	MOLECULAR ONCOLOGY	2020			0	0

PKMYT1 is associated with prostate cancer malignancy and may serve as a therapeutic target	Wang, Jianan; Wang, Lin; Chen, Saipeng; Peng, Huahong; Xiao, Longfei; Du, E.; Liu, Yan; Lin, Dong; Wang, Yuzhuo; Xu, Yong; Yang, Kuo	GENE	2020	744		0	0
The role of the histone H3 variant CENPA in prostate cancer	Saha, Anjan K.; Contreras-Galindo, Rafael; Niknafs, Yashar S.; Iyer, Matthew; Qin, Tingting; Padmanabhan, Karthik; Siddiqui, Javed; Palande, Monica; Wang, Claire; Qian, Brian; Ward, Elizabeth; Tang, Tara; Tomlins, Scott A.; Gitlin, Scott D.; Sartor, Maureen A.; Omenn, Gilbert S.; Chinnaiyan, Arul M.; Markovitz, David M.	JOURNAL OF BIOLOGICAL CHEMISTRY	2020	295	25	0	0
Therapy-induced lipid uptake and remodeling underpin ferroptosis hypersensitivity in prostate cancer	Tousignant, Kaylyn D.; Rockstroh, Anja; Poad, Berwyck L. J.; Talebi, Ali; Young, Reuben S. E.; Fard, Atefeh Taherian; Gupta, Rajesh; Zang, Tuo; Wang, Chenwei; Lehman, Melanie L.; Swinnen, Johan, V; Blanksby, Stephen J.; Nelson, Colleen C.; Sadowski, Martin C.	CANCER & METABOLISM	2020	8	1	0	0
Immune landscape of human prostate cancer: immune evasion mechanisms and biomarkers for personalized immunotherapy	Bou-Dargham, Mayassa J.; Sha, Linlin; Sang, Qing-Xiang Amy; Zhang, Jinfeng	BMC CANCER	2020	20	1	0	0
AR and ERG drive the expression of prostate cancer specific long noncoding RNAs	Kohvakka, Annika; Sattari, Mina; Shcherban, Anastasia; Annala, Matti; Urbanucci, Alfonso; Kesseli, Juha; Tammela, Teuvo L. J.; Kivinummi, Kati; Latonen, Leena; Nykter, Matti; Visakorpi, Tapio	ONCOGENE	2020	39	30	0	0
G9a regulates tumorigenicity and stemness through genome-wide DNA methylation reprogramming in non-small cell lung cancer	Pangeni, Rajendra P.; Yang, Lu; Zhang, Keqiang; Wang, Jinhui; Li, Wendong; Guo, Chao; Yun, Xinwei; Sun, Ting; Wang, Jami; Raz, Dan J.	CLINICAL EPIGENETICS	2020	12	1	0	0

Plasma metabolomic profile in prostatic intraepithelial neoplasia and prostate cancer and associations with the prostate-specific antigen and the Gleason score	Markin, Pavel A.; Brito, Alex; Moskaleva, Natalia; Lartsova, Ekaterina, V; Shpot, Yevgeny, V; Lerner, Yulia, V; Mikhajlov, Vasily Y.; Potoldykova, Natalia, V; Enikeev, Dimitry, V; La Frano, Michael R.; Appolonova, Svetlana A.	METABOLOMICS	2020	16	7	0	0
Coexpression Analysis of the EZH2 Gene Using The Cancer Genome Atlas and Oncomine Databases Identifies Coexpressed Genes Involved in Biological Networks in Breast Cancer, Glioblastoma, and Prostate Cancer	Zhu, Jin; Jin, Lu; Zhang, Aili; Gao, Peng; Dai, Guangcheng; Xu, Ming; Xu, Lijun; Yang, Dongrong	MEDICAL SCIENCE MONITOR	2020	26		0	0
Association ofKLK3,VAMP8andMDM4Genetic Variants within microRNA Binding Sites with Prostate Cancer: Evidence from Serbian Population	Kotarac, Nevena; Dobrijevic, Zorana; Matijasevic, Suzana; Savic-Pavicevic, Dusanka; Brajuskovic, Goran	PATHOLOGY & ONCOLOGY RESEARCH	2020			0	0
Sub-zeptomole Detection of Biomarker Proteins Using a Microfluidic Immunoarray with Nanostructured Sensors	Dhanapala, Lasangi; Jones, Abby L.; Czarnecki, Patricia; Rusling, James F.	ANALYTICAL CHEMISTRY	2020	92	12	0	0
Downregulation of CollagenCOL4A6Is Associated with Prostate Cancer Progression and Metastasis	Ma, Jian-Bin; Bai, Ji-Yu; Zhang, Hai-Bao; Gu, Lijiang; He, Dalin; Guo, Peng	GENETIC TESTING AND MOLECULAR BIOMARKERS	2020	24	7	0	0
Prediction of MiR-21-5p in Promoting the Development of Lung Adenocarcinoma via PDZD2 Regulation	Cui, Shengjin; Lou, Shuang; Guo, Weiquan; Jian, Shihui; Wu, Yunfeng; Liu, Xintong; Lan, Xi; Jia, Xingwang	MEDICAL SCIENCE MONITOR	2020	26		0	0

Enzyme-mediated depletion of serum L-Met abrogates prostate cancer growth via multiple mechanisms without evidence of systemic toxicity	Lu, Wei-Cheng; Saha, Achinto; Yan, Wupeng; Garrison, Kendra; Lamb, Candice; Pandey, Renu; Irani, Seema; Lodi, Alessia; Lu, Xiyuan; Tiziani, Stefano; Zhang, Yan Jessie; Georgiou, George; DiGiovanni, John; Stone, Everett	PROCEEDINGS OF THE NATIONAL ACADEMY OF SCIENCES OF THE UNITED STATES OF AMERICA	2020	117	23	0	0
Systemic modulation of stress and immune parameters in patients treated for prostate adenocarcinoma by intensity-modulated radiation therapy or stereotactic ablative body radiotherapy	Frey, B.; Mika, J.; Jelonek, K.; Cruz-Garcia, L.; Roelants, C.; Testard, I; Cherradi, N.; Lumniczky, K.; Polozov, S.; Napieralska, A.; Widlak, P.; Gaipl, U. S.; Badie, C.; Polanska, J.; Candelas, S. M.	STRAHLENTHERAPIE UND ONKOLOGIE	2020			0	0
Incarnatapeptins A and B, Nonribosomal Peptides Discovered Using Genome Mining and H-1/N-15 HSQC-TOCSY	Morgan, Kalindi D.; Williams, David E.; Patrick, Brian O.; Remigy, Marion; Banuelos, Carmen A.; Sadar, Marianne D.; Ryan, Katherine S.; Andersen, Raymond J.	ORGANIC LETTERS	2020	22	11	0	0
H-1 NMR-Based Urine Metabolomics Reveals Signs of Enhanced Carbon and Nitrogen Recycling in Prostate Cancer	Bruzzone, Chiara; Loizaga-Iriarte, Ana; Sanchez-Mosquera, Pilar; Gil-Redondo, Ruben; Astobiza, Ianire; Diercks, Tammo; Cortazar, Ana R.; Ugalde-Olano, Aitziber; Schaefer, Hartmut; Blanco, Francisco J.; Unda, Miguel; Cannet, Claire; Spraul, Manfred; Mato, Jose M.; Embade, Nieves; Carracedo, Arkaitz; Millet, Oscar	JOURNAL OF PROTEOME RESEARCH	2020	19	6	0	0
Darolutamide antagonizes androgen signaling by blocking enhancer and super-enhancer activation	Baumgart, Simon J.; Nevedomskaya, Ekaterina; Lesche, Ralf; Newman, Richard; Mumberg, Dominik; Haendler, Bernard	MOLECULAR ONCOLOGY	2020			0	0
New findings on urinary prostate cancer metabolome through combined GC-MS and(1)H NMR analytical platforms	Lima, Ana Rita; Pinto, Joana; Barros-Silva, Daniela; Jeronimo, Carmen; Henrique, Rui; Bastos, Maria de Lourdes; Carvalho, Marcia; Pinho, Paula Guedes	METABOLOMICS	2020	16	6	0	0

MARCH5 mediates NOXA-dependent MCL1 degradation driven by kinase inhibitors and integrated stress response activation	Arai, Seiji; Varkaris, Andreas; Nouri, Mannan; Chen, Sen; Xie, Lisha; Balk, Steven P.	ELIFE	2020	9		0	0
Conditionally Reprogrammed Cells from Patient-Derived Xenograft to Model Neuroendocrine Prostate Cancer Development	Ci, Xinpei; Hao, Jun; Dong, Xin; Xue, Hui; Wu, Rebecca; Choi, Stephen Yiu Chuen; Haegert, Anne M.; Collins, Colin C.; Liu, Xuefeng; Lin, Dong; Wang, Yuzhuo	CELLS	2020	9	6	0	0
Clinical Applications of Molecular Biomarkers in Prostate Cancer	Counago, Felipe; Lopez-Campos, Fernando; Diaz-Gavela, Ana Aurora; Almagro, Elena; Fenandez-Pascual, Esau; Henriquez, Ivan; Lozano, Rebeca; Espinos, Estefania Linares; Gomez-Iturriaga, Alfonso; de Velasco, Guillermo; Franco, Luis Miguel Quintana; Rodriguez-Melcon, Ignacio; Lopez-Torrecilla, Jose; Spratt, Daniel E.; Guerrero, Luis Leonardo; Martinez-Salamanca, Juan Ignacio; del Cerro, Elia	CANCERS	2020	12	6	0	0
Liquid Biopsy in Colorectal Carcinoma: Clinical Applications and Challenges	Kolencik, Drahomir; Shishido, Stephanie N.; Pitule, Pavel; Mason, Jeremy; Hicks, James; Kuhn, Peter	CANCERS	2020	12	6	0	0
Low Molecular Weight Fucoidan Prevents Radiation-Induced Fibrosis and Secondary Tumors in a Zebrafish Model	Wu, Szu-Yuan; Yang, Wan-Yu; Cheng, Chun-Chia; Hsiao, Ming-Chen; Tsai, Shin-Lin; Lin, Hua-Kuo; Lin, Kuan-Hao; Yuh, Chiou-Hwa	CANCERS	2020	12	6	0	0
New Frontiers in Prostate Cancer Treatment: Are We Ready for Drug Combinations with Novel Agents?	Aurilio, Gaetano; Cimadamore, Alessia; Santoni, Matteo; Nole, Franco; Scarpelli, Marina; Massari, Francesco; Lopez-Beltran, Antonio; Cheng, Liang; Montironi, Rodolfo	CELLS	2020	9	6	0	0
DNA Methylation Changes in Human Papillomavirus-Driven Head and Neck Cancers	Weeramange, Chameera Ekanayake; Tang, Kai Dun; Vasani, Sarju; Langton-Lockton, Julian; Kenny, Liz; Punyadeera, Chamindie	CELLS	2020	9	6	0	0

Prostate cancer biology & genomics	Whitaker, Hayley; Tam, Joseph O.; Connor, Martin J.; Grey, Alistair	TRANSLATIONAL ANDROLOGY AND UROLOGY	2020	9	3	0	0
Triple Selection Strategy for In Situ Labeling of Circulating Tumor Cells with High Purity and Viability toward Preclinical Personalized Drug Sensitivity Analysis	Mu, Hsuan-Yo; Ou, Yen-Chuan; Chuang, Han-Ni; Lu, Tsai-Jung; Jhan, Pei-Pei; Hsiao, Tzu-Hung; Huang, Jen-Huang	ADVANCED BIOSYSTEMS	2020	4	6	0	0
Identification of Potential Key Genes for Pathogenesis and Prognosis in Prostate Cancer by Integrated Analysis of Gene Expression Profiles and the Cancer Genome Atlas	Liu, Shuang; Wang, Wenxin; Zhao, Yan; Liang, Kaige; Huang, Yaojiang	FRONTIERS IN ONCOLOGY	2020	10		0	0
Detection of Molecular Signatures of Homologous Recombination Deficiency in Prostate Cancer with or without BRCA1/2 Mutations	Sztupinszki, Zsofia; Diossy, Miklos; Krzystanek, Marcin; Borcsok, Judit; Pomerantz, Mark M.; Tisza, Viktoria; Spisak, Sandor; Rusz, Orsolya; Csabai, Istvan; Freedman, Matthew L.; Szallasi, Zoltan	CLINICAL CANCER RESEARCH	2020	26	11	0	0
An Emerging Landscape for Canonical and Actionable Molecular Alterations in Primary and Metastatic Prostate Cancer	Dawson, Nancy A.; Zibelman, Matthew; Lindsay, Timothy; Feldman, Rebecca A.; Saul, Michelle; Gatalica, Zoran; Korn, W. Michael; Heath, Elisabeth, I	MOLECULAR CANCER THERAPEUTICS	2020	19	6	0	0
Extracellular vesicles as biomarkers in malignant pleural mesothelioma: A review	Ahmadzada, Tamkin; Kao, Steven; Reid, Glen; Clarke, Stephen; Grau, Georges E.; Hosseini-Beheshti, Elham	CRITICAL REVIEWS IN ONCOLOGY HEMATOLOGY	2020	150		0	0
AR-dependent phosphorylation and phospho-proteome targets in prostate cancer	Venkadakrishnan, Varadha Balaji; Ben-Salem, Salma; Heemers, Hannelore, V	ENDOCRINE-RELATED CANCER	2020	27	6	0	0
Germline polymorphisms associated with impaired survival outcomes and somatic tumor alterations in advanced prostate cancer	Chen, William S.; Feng, Eric L.; Aggarwal, Rahul; Foye, Adam; Beer, Tomasz M.; Alumkal, Joshi J.; Gleave, Martin; Chi, Kim N.; Reiter, Robert E.; Rettig, Matthew B.; Evans, Christopher P.; Small, Eric J.; Sharifi, Nima; Zhao, Shuang G.	PROSTATE CANCER AND PROSTATIC DISEASES	2020	23	2	0	0

Genomics models in radiotherapy: From mechanistic to machine learning	Kang, John; Coates, James T.; Strawderman, Robert L.; Rosenstein, Barry S.; Kerns, Sarah L.	MEDICAL PHYSICS	2020	47	5	0	0
PCAT1: An oncogenic lncRNA in diverse cancers and a putative therapeutic target	Ghafouri-Fard, Soudeh; Dashti, Sepideh; Taheri, Mohammad	EXPERIMENTAL AND MOLECULAR PATHOLOGY	2020	114		0	0
Challenges, applications and future directions of precision medicine in prostate cancer - the role of organoids and patient-derived xenografts	Joshi, Andre; Roberts, Matthew J.; Alinezhad, Saeid; Williams, Elizabeth D.; Vela, Ian	BJU INTERNATIONAL	2020	126	1	0	0
Reverse transcriptase inhibitors promote the remodelling of nuclear architecture and induce autophagy in prostate cancer cells	Bellisai, Cristina; Sciamanna, Ilaria; Rovella, Paola; Giovannini, Daniela; Baranzini, Mirko; Pugliese, Giusj Monia; Ansari, Mohammad Salik Zeya; Milite, Ciro; Sinibaldi-Vallebona, Paola; Cirilli, Roberto; Sbardella, Gianluca; Pichierri, Pietro; Trisciuoglio, Daniela; Lavia, Patrizia; Serafino, Annalucia; Spadafora, Corrado	CANCER LETTERS	2020	478		0	0
Detecting Prognosis Risk Biomarkers for Colon Cancer Through Multi-Omics-Based Prognostic Analysis and Target Regulation Simulation Modeling	Yin, Zuojing; Yan, Xinmiao; Wang, Qiming; Deng, Zeliang; Tang, Kailin; Cao, Zhiwei; Qiu, Tianyi	FRONTIERS IN GENETICS	2020	11		0	0
Combined TP53 and RB1 Loss Promotes Prostate Cancer Resistance to a Spectrum of Therapeutics and Confers Vulnerability to Replication Stress	Nyquist, Michael D.; Corella, Alexandra; Coleman, Ilsa; De Sarkar, Navonil; Kaipainen, Arja; Ha, Gavin; Gulati, Roman; Ang, Lisa; Chatterjee, Payel; Lucas, Jared; Pritchard, Colin; Risbridger, Gail; Isaacs, John; Montgomery, Bruce; Morrissey, Colm; Corey, Eva; Nelson, Peter S.	CELL REPORTS	2020	31	8	0	0

Independence of HIF1a and androgen signaling pathways in prostate cancer	Tran, Maxine G. B.; Bibby, Becky A. S.; Yang, Lingjian; Lo, Franklin; Warren, Anne Y.; Shukla, Deepa; Osborne, Michelle; Hadfield, James; Carroll, Thomas; Stark, Rory; Scott, Helen; Ramos-Montoya, Antonio; Massie, Charlie; Maxwell, Patrick; West, Catharine M. L.; Mills, Ian G.; Neal, David E.	BMC CANCER	2020	20	1	0	0
The genetic landscapes of urological cancers and their clinical implications in the era of high-throughput genome analysis	Light, Alexander; Ahmed, Aamir; Dasgupta, Prokar; Elhage, Oussama	BJU INTERNATIONAL	2020	126	1	0	0
CDCA2 Inhibits Apoptosis and Promotes Cell Proliferation in Prostate Cancer and Is Directly Regulated by HIF-1 alpha Pathway	Zhang, Yixiang; Cheng, Yingduan; Zhang, Zhaoxia; Bai, Zhongyuan; Jin, Hongtao; Guo, Xiaojing; Huang, Xiaoyan; Li, Meiqi; Wang, Maolin; Shu, Xing-sheng; Yuan, Yeqing; Ying, Ying	FRONTIERS IN ONCOLOGY	2020	10		0	0
2,4-dienoyl-CoA reductase regulates lipid homeostasis in treatment-resistant prostate cancer	Blomme, Arnaud; Ford, Catriona A.; Mui, Ernest; Patel, Rachana; Ntala, Chara; Jamieson, Lauren E.; Planque, Melanie; McGregor, Grace H.; Peixoto, Paul; Hervouet, Eric; Nixon, Colin; Salji, Mark; Gaughan, Luke; Markert, Elke; Repiscak, Peter; Sumpton, David; Blanco, Giovanny Rodriguez; Lilla, Sergio; Kamphorst, Jurre J.; Graham, Duncan; Faulds, Karen; MacKay, Gillian M.; Fendt, Sarah-Maria; Zanivan, Sara; Leung, Hing Y.	NATURE COMMUNICATIONS	2020	11	1	0	0
Long non-coding RNA FAM66C is associated with clinical progression and promotes cell proliferation by inhibiting proteasome pathway in prostate cancer	Xie, Yimin; Gu, Jie; Qin, Zhenqian; Ren, Zhen; Wang, Yanwei; Shi, Haifeng; Chen, Binghai	CELL BIOCHEMISTRY AND FUNCTION	2020			0	0

Comprehensive metabolomics analysis of prostate cancer tissue in relation to tumor aggressiveness and TMPRSS2-ERG fusion status	Dudka, Ilona; Thysell, Elin; Lundquist, Kristina; Antti, Henrik; Iglesias-Gato, Diego; Flores-Morales, Amilcar; Bergh, Anders; Wikstrom, Pernilla; Grobner, Gerhard	BMC CANCER	2020	20	1	0	0
Identification and Validation of Prognostically Relevant Gene Signature in Melanoma	Gao, Yali; Li, Yaling; Niu, Xueli; Wu, Yutong; Guan, Xiuhao; Hong, Yuxiao; Chen, Hongduo; Song, Bing	BIOMED RESEARCH INTERNATIONAL	2020	2020		0	0
Aggressive prostate cancer phenotype and genome-wide association studies: where are we now?	Pinto, Ana R.; Silva, Jani; Pinto, Ricardo; Medeiros, Rui	PHARMACOGENOMICS	2020	21	7	0	0
Phospholipase C-like protein 2 (PLC-L2) is associated with cytolytic ability of CD8(+) T cells and prognosis of prostate cancer	Li, Kaiwen; Ma, Xiaoming; Wang, Qiong; Zhou, Qianghua; Chen, Xu; Guo, Zhenghui; Lai, Yiming; Tao, Yiran; Wu, Wanhua; Peng, Shirong; Cai, Wenli; Bai, Shoumin; Huang, Hai	MATERIALS EXPRESS	2020	10	5	0	0
SLFN11 Expression in Advanced Prostate Cancer and Response to Platinum-based Chemotherapy	Conteduca, Vincenza; Ku, Sheng-Yu; Puca, Loredana; Slade, Megan; Fernandez, Luisa; Hess, Judy; Bareja, Rohan; Vlachostergios, Panagiotis J.; Sigouros, Michael; Mosquera, Juan Miguel; Sboner, Andrea; Nanus, David M.; Elemento, Olivier; Dittamore, Ryan; Tagawa, Scott T.; Beltran, Himisha	MOLECULAR CANCER THERAPEUTICS	2020	19	5	0	0
Prognostic value of mitotic checkpoint protein BUB3, cyclin B1, and pituitary tumor-transforming 1 expression in prostate cancer	Ersvaer, Elin; Kildal, Wanja; Vlatkovic, Ljiljana; Cyll, Karolina; Pradhan, Manohar; Kleppe, Andreas; Hveem, Tarjei S.; Askautrud, Hanne A.; Novelli, Marco; Waehre, Hakon; Liestol, Knut; Danielsen, Havard E.	MODERN PATHOLOGY	2020	33	5	0	0
PLC epsilon knockdown enhances the radiosensitivity of castration-resistant prostate cancer via the AR/PARP1/DNA-PKcs axis	Pu, Jun; Li, Ting; Liu, Nanjing; Luo, Chunli; Quan, Zhen; Li, Luo; Wu, Xiaohou	ONCOLOGY REPORTS	2020	43	5	0	0
Proteomic and transcriptomic profiling of Pten gene-knockout mouse model of prostate cancer	Zhang, Jinhui; Kim, Sangyub; Li, Li; Kemp, Christopher J.; Jiang, Cheng; Lu, Junxuan	PROSTATE	2020	80	7	0	0

A comprehensive review of the role of long non-coding RNAs in organs with an endocrine function	Ghafouri-Fard, Soudeh; Esmaeili, Mohammadhosein; Shoorei, Hamed; Taheri, Mohammad	BIOMEDICINE & PHARMACOTHERAPY	2020	125		0	0
Epigenetic regulation in human cancer: the potential role of epi-drug in cancer therapy	Lu, Yuanjun; Chan, Yau-Tuen; Tan, Hor-Yue; Li, Sha; Wang, Ning; Feng, Yibin	MOLECULAR CANCER	2020	19	1	0	0
Circulating tumor cells as Trojan Horse for understanding, preventing, and treating cancer: a critical appraisal	Mentis, Alexios-Fotios A.; Grivas, Petros D.; Dardiotis, Efthimios; Romas, Nicholas A.; Papavassiliou, Athanasios G.	CELLULAR AND MOLECULAR LIFE SCIENCES	2020			0	0
Emerging role of PTEN loss in evasion of the immune response to tumours	Vidotto, Thiago; Melo, Camila Morais; Castelli, Erick; Koti, Madhuri; dos Reis, Rodolfo Borges; Squire, Jeremy A.	BRITISH JOURNAL OF CANCER	2020			0	0
ARNT-dependent CCR8 reprogrammed LDH isoform expression correlates with poor clinical outcomes of prostate cancer	Chen, Guo; Cai, Zhi-duan; Lin, Zhuo-yuan; Wang, Cong; Liang, Yu-xiang; Han, Zhao-dong; He, Hui-chan; Mo, Ru-jun; Lu, Jian-ming; Pan, Bin; Wu, Chin-lee; Wang, Fen; Zhong, Wei-de	MOLECULAR CARCINOGENESIS	2020	59	8	0	0
Intratumor delta-catenin heterogeneity driven by genomic rearrangement dictates growth factor dependent prostate cancer progression	Li, Mingchuan; Nopparat, Jongdee; Aguilar, Byron J.; Chen, Yan-hua; Zhang, Jiao; Du, Jie; Ai, Xin; Luo, Yong; Jiang, Yongguang; Boykin, Christi; Lu, Qun	ONCOGENE	2020	39	22	0	0
Upregulation of PTTG1 is associated with poor prognosis in prostate cancer	Fraune, Christoph; Yehorov, Serhiy; Luebke, Andreas M.; Steurer, Stefan; Hube-Magg, Claudia; Buescheck, Franziska; Hoeflmayer, Doris; Tsourlakis, Maria Christina; Clauditz, Till S.; Simon, Ronald; Sauter, Guido; Weidemann, Soeren; Dum, David; Kind, Simon; Minner, Sarah; Schlomm, Thorsten; Huland, Hartwig; Heinzer, Hans; Graefen, Markus; Burandt, Eike	PATHOLOGY INTERNATIONAL	2020	70	7	0	0

Functional and genomic characterization of three novel cell lines derived from a metastatic gallbladder cancer tumor	Garcia, Patricia; Bizama, Carolina; Rosa, Lorena; Espinoza, Jaime A.; Weber, Helga; Cerda-Infante, Javier; Sanchez, Marianela; Montecinos, Viviana P.; Lorenzo-Bermejo, Justo; Boekstegers, Felix; Davila-Lopez, Marcela; Alfaro, Francisca; Leiva-Acevedo, Claudia; Parra, Zasha; Romero, Diego; Kato, Sumie; Leal, Pamela; Lagos, Marcela; Carlos Roa, Juan	BIOLOGICAL RESEARCH	2020	53	1	0	0
Clinical and genomic characterization of Low PSA Secretors: a unique subset of metastatic castration resistant prostate cancer	Aggarwal, Rahul; Romero, Gustavo Rubio; Friedl, Verena; Weinstein, Alana; Foye, Adam; Huang Jiaoti; Feng, Felix; Stuart, Joshua M.; Small, Eric J.	PROSTATE CANCER AND PROSTATIC DISEASES	2020			0	0
Loss of CHD1 Promotes Heterogeneous Mechanisms of Resistance to AR-Targeted Therapy via Chromatin Dysregulation	Zhang, Zeda; Zhou, Chuanli; Li, Xiaoling; Barnes, Spencer D.; Deng, Su; Hoover, Elizabeth; Chen, Chi-Chao; Lee, Young Sun; Zhang, Yanxiao; Wang, Choushi; Metang, Lauren A.; Wu, Chao; Tirado, Carla Rodriguez; Johnson, Nickolas A.; Wongvipat, John; Navrazhina, Kristina; Cao, Zhen; Choi, Danielle; Huang, Chun-Hao; Linton, Eliot; Chen, Xiaoping; Liang, Yupu; Mason, Christopher E.; de Stanchina, Elisa; Abida, Wassim; Lujambio, Amaia; Li, Sheng; Lowe, Scott W.; Mendell, Joshua T.; Malladi, Venkat S.; Sawyers, Charles L.; Mu, Ping	CANCER CELL	2020	37	4	0	0
Quantitative SWATH-Based Proteomic Profiling for Identification of Mechanism-Driven Diagnostic Biomarkers Conferring in the Progression of Metastatic Prostate Cancer	Singh, Anshika N.; Sharma, Neeti	FRONTIERS IN ONCOLOGY	2020	10		0	0

Broad and thematic remodeling of the surfaceome and glycoproteome on isogenic cells transformed with driving proliferative oncogenes	Leung, Kevin K.; Wilson, Gary M.; Kirkemo, Lisa L.; Riley, Nicholas M.; Coon, Joshua J.; Wells, James A.	PROCEEDINGS OF THE NATIONAL ACADEMY OF SCIENCES OF THE UNITED STATES OF AMERICA	2020	117	14	0	0
Circulating cell-free DNA: Translating prostate cancer genomics into clinical care	Tandefelt, Delila Gasi; de Bono, Johann	MOLECULAR ASPECTS OF MEDICINE	2020	72		0	0
EGCG Mediated Targeting of Deregulated Signaling Pathways and Non-Coding RNAs in Different Cancers: Focus on JAK/STAT, Wnt/beta-Catenin, TGF/SMAD, NOTCH, SHH/GLI, and TRAIL Mediated Signaling Pathways	Farooqi, Ammad Ahmad; Pinheiro, Marina; Granja, Andreia; Farabegoli, Fulvia; Reis, Salette; Attar, Rukset; Sabitaliyevich, Uteuliyev Yerzhan; Xu, Baojun; Ahmad, Aamir	CANCERS	2020	12	4	0	0
The oncogenic roles of bacterial infections in development of cancer	Eyvazi, Shirin; Vostakolaei, Mehdi Asghari; Dilmaghani, Azita; Borumandi, Omid; Hejazi, Mohammad Saeid; Kahroba, Houman; Tarhriz, Vahideh	MICROBIAL PATHOGENESIS	2020	141		0	0
Identification of potential crucial genes associated with the pathogenesis and prognosis of prostate cancer	Mu, Hai-Qi; Liang, Zhi-Qiang; Xie, Qi-Peng; Han, Wei; Yang, Sen; Wang, Shuai-Bin; Zhao, Cheng; Cao, Ye-Min; He, You-Hua; Chen, Jian	BIOMARKERS IN MEDICINE	2020	14	5	0	0
Exome sequencing identified six copy number variations as a prediction model for recurrence of primary prostate cancers with distinctive prognosis	Liu, Jie; Yan, Jiajun; Mao, Ruifang; Ren, Guoping; Liu, Xiaoyan; Zhang, Yanling; Wang, Jili; Wang, Yan; Li, Meiling; Qiu, Qingchong; Wang, Lin; Liu, Guanfeng; Jin, Shanshan; Ma, Liang; Ma, Yingying; Zhao, Na; Zhang, Hongwei; Lin, Biaoyang	TRANSLATIONAL CANCER RESEARCH	2020	9	4	0	0

STAT3-dependent analysis reveals PDK4 as independent predictor of recurrence in prostate cancer	Oberhuber, Monika; Pecoraro, Matteo; Rusz, Mate; Oberhuber, Georg; Wieselberg, Maritta; Haslinger, Peter; Gurnhofer, Elisabeth; Schlederer, Michaela; Limberger, Tanja; Lagger, Sabine; Pencik, Jan; Kodajova, Petra; Hoegler, Sandra; Stockmaier, Georg; Grund-Groeschke, Sandra; Aberger, Fritz; Bolis, Marco; Theurillat, Jean-Philippe; Wiebringhaus, Robert; Weiss, Theresa; Haitel, Andrea; Brehme, Marc; Wadsak, Wolfgang; Griss, Johannes; Mohr, Thomas; Hofer, Alexandra; Jaeger, Anton; Pollheimer, Juergen; Egger, Gerda; Koellensperger, Gunda; Mann, Matthias; Hantusch, Brigitte; Kenner, Lukas	MOLECULAR SYSTEMS BIOLOGY	2020	16	4	0	0
MULTI-PARAMETRIC MAGNETIC RESONANCE IMAGING OF MULTI-FOCAL PROSTATE CANCER UNMASKS INTRA-PROSTATIC GENOMIC HETEROGENEITY AND NOVEL RADIO-GENOMIC CORRELATES: RESULTS OF THE SMARTER PROSTATE INTERVENTIONS AND THERAPEUTICS (SPIRIT) STUDY	Chin, Joseph; Correa, Rohann; Aref-Eshghi, Erfan; Alfano, Ryan; Sadikovic, Bekim; Ward, Aaron; Boutros, Paul; Bartlett, John; Kassam, Zahra; Pautler, Stephen; Gaed, Mena; GomezLemus, Jose; Moussa, Madeleine; Bauman, Glenn	JOURNAL OF UROLOGY	2020	203		0	0
Development and validation of hub genes for lymph node metastasis in patients with prostate cancer	Xu, Ning; Chen, Shao-Hao; Lin, Ting-Ting; Cai, Hai; Ke, Zhi-Bin; Dong, Ru-Nan; Huang, Peng; Li, Xiao-Dong; Chen, Ye-Hui; Zheng, Qing-Shui	JOURNAL OF CELLULAR AND MOLECULAR MEDICINE	2020	24	8	0	0

Diverse AR Gene Rearrangements Mediate Resistance to Androgen Receptor Inhibitors in Metastatic Prostate Cancer	Li, Yingming; Yang, Rendong; Henzler, Christine M.; Ho, Yeung; Passow, Courtney; Auch, Benjamin; Carreira, Suzanne; Rodrigues, Daniel Nava; Bertan, Claudia; Tae Hyun Hwang; Quigley, David A.; Dang, Ha X.; Morrissey, Colm; Fraser, Michael; Plymate, Stephen R.; Maher, Christopher A.; Feng, Felix Y.; de Bono, Johann S.; Dehm, Scott M.	CLINICAL CANCER RESEARCH	2020	26	8	0	0
TP53 Gain-of-Function Mutations in Circulating Tumor DNA in Men With Metastatic Castration-Resistant Prostate Cancer	Chapman, Lynne; Ledet, Elisa M.; Barata, Pedro C.; Cotogno, Patrick; Manogue, Charlotte; Moses, Marcus; Christensen, Bryce R.; Steinwald, Peter; Ranasinghe, Lahiru; Layton, Jodi L.; Lewis, Brian E.; Sartor, Oliver	CLINICAL GENITOURINARY CANCER	2020	18	2	0	0
A piece in prostate cancer puzzle: Future perspective of novel molecular signatures	Nassir, Anmar M.	SAUDI JOURNAL OF BIOLOGICAL SCIENCES	2020	27	4	0	0
Nuclear magnetic resonance spectroscopy of human body fluids and in vivo magnetic resonance spectroscopy: Potential role in the diagnosis and management of prostate cancer	Gholizadeh, Neda; Pundavela, Jay; Nagarajan, Rajakumar; Dona, Anthony; Quadrelli, Scott; Biswas, Tapan; Greer, Peter B.; Ramadan, Saadallah	UROLOGIC ONCOLOGY-SEMINARS AND ORIGINAL INVESTIGATIONS	2020	38	4	0	0
Current Perspectives on Circulating Tumor DNA, Precision Medicine, and Personalized Clinical Management of Cancer	Oliveira, Kelly C. S.; Ramos, Iago Barroso; Silva, Jessica M. C.; Barra, Williams Fernandes; Riggins, Gregory J.; Palande, Vikrant; Pinho, Catarina Torres; Frenkel-Morgenstern, Milana; Santos, Sidney E. B.; Assumpcao, Paulo P.; Burbano, Rommel R.; Calcagno, Danielle Queiroz	MOLECULAR CANCER RESEARCH	2020	18	4	0	0
Alternative Splicing in the Nuclear Receptor Superfamily Expands Gene Function to Refine Endo-Xenobiotic Metabolism	Annalora, Andrew J.; Marcus, Craig B.; Iversen, Patrick L.	DRUG METABOLISM AND DISPOSITION	2020	48	4	0	0

Metabolomic effects of androgen deprivation therapy treatment for prostate cancer	Chi, Jen-Tsan; Lin, Pao-Hwa; Tolstikov, Vladimir; Oyekunle, Taofik; Chen, Emily Y.; Bussberg, Valerie; Greenwood, Bennett; Sarangarajan, Rangaprasad; Narain, Niven R.; Kiebish, Michael A.; Freedland, Stephen J.	CANCER MEDICINE	2020	9	11	0	0
The Prospect of Identifying Resistance Mechanisms for Castrate-Resistant Prostate Cancer Using Circulating Tumor Cells: Is Epithelial-to-Mesenchymal Transition a Key Player?	Khan, Tanzila; Scott, Kieran F.; Becker, Therese M.; Lock, John; Nimir, Mohammed; Ma, Yafeng; de Souza, Paul	PROSTATE CANCER	2020	2020		0	0
How should radiologists incorporate non-imaging prostate cancer biomarkers into daily practice?	Rajwa, Pawel; Syed, Jamil; Leapman, Michael S.	ABDOMINAL RADIOLOGY	2020			0	0
Performance of clinicopathologic models in men with high risk localized prostate cancer: impact of a 22-gene genomic classifier	Tosoian, Jeffrey J.; Birer, Samuel R.; Karnes, R. Jeffrey; Zhang, Jingbin; Davicioni, Elai; Klein, Eric E.; Freedland, Stephen J.; Weinmann, Sheila; Trock, Bruce J.; Dess, Robert T.; Zhao, Shuang G.; Jackson, William C.; Yamoah, Kosj; Dal Pral, Alan; Mahal, Brandon A.; Morgan, Todd M.; Mehra, Rohit; Kaffenberger, Samuel; Salami, Simpa S.; Kane, Christopher; Pollack, Alan; Den, Robert B.; Berlin, Alejandro; Schaeffer, Edward M.; Nguyen, Paul L.; Feng, Felix Y.; Spratt, Daniel E.	PROSTATE CANCER AND PROSTATIC DISEASES	2020			0	0
Proteomic and genomic biomarkers for Non-Small Cell Lung Cancer: Peroxiredoxin, Haptoglobin, and Alpha-1 antitrypsin	Najafi, Zahra; Mohamadnia, Abdolreza; Ahmadi, Rahim; Mahmoudi, Minoo; Bahrami, Naghmeh; Khosravi, Adnan; Jamaati, Hamidreza; Tabarsi, Payam; Dizaji, Mehdi Kazem Pour; Shirian, Sadegh	CANCER MEDICINE	2020	9	11	0	0

Differential expression of circulating serum miR-1249-3p, miR-3195, and miR-3692-3p in non-small cell lung cancer	Kumar, Sachin; Sharawat, Surender K.; Ali, Ashraf; Gaur, Vikas; Malik, Prabhat Singh; Pandey, Monu; Kumar, Sunil; Mohan, Anant; Guleria, Randeep	HUMAN CELL	2020	33	3	0	0
Intratumoral heterogeneity and genetic characteristics of prostate cancer	Wu, Bo; Lu, Xin; Shen, Haibo; Yuan, Xiaobin; Wang, Xin; Yin, Nan; Sun, Libin; Shen, Pengliang; Hu, Caoyang; Jiang, Huanrong; Wang, Dongwen	INTERNATIONAL JOURNAL OF CANCER	2020	146	12	0	0
Type 2 diabetes induced microbiome dysbiosis is associated with therapy resistance in pancreatic adenocarcinoma	Kesh, Kousik; Mendez, Roberto; Abdelrahman, Leila; Banerjee, Santanu; Banerjee, Sulagna	MICROBIAL CELL FACTORIES	2020	19	1	0	0
A novel stratification framework for predicting outcome in patients with prostate cancer	Luca, Bogdan-Alexandru; Moulton, Vincent; Ellis, Christopher; Edwards, Dylan R.; Campbell, Colin; Cooper, Rosalin A.; Clark, Jeremy; Brewer, Daniel S.; Cooper, Colin S.	BRITISH JOURNAL OF CANCER	2020	122	10	0	0
Identification of the differentially expressed protein biomarkers in rat blood plasma in response to gamma irradiation	Sun, Jia-Li; Li, Shuang; Lu, Xue; Feng, Jiang-Bin; Cai, Tian-Jing; Tian, Mei; Liu, Qing-Jie	INTERNATIONAL JOURNAL OF RADIATION BIOLOGY	2020	96	6	0	0
Prostate cancer: more effective use of underutilized postoperative radiation therapy	Motterle, Giovanni; Morlacco, Alessandro; Zattoni, Fabio; Karnes, R. Jeffrey	EXPERT REVIEW OF ANTICANCER THERAPY	2020	20	4	0	0
Loss of the adhesion molecule CEACAM1 is associated with early biochemical recurrence in TMPRSS2:ERG fusion-positive prostate cancers	Luebke, Andreas M.; Ricken, Wiebke; Kluth, Martina; Hube-Magg, Claudia; Schroeder, Cornelia; Buescheck, Franziska; Moeller, Katharina; Dum, David; Hoeflmayer, Doris; Weidemann, Soeren; Fraune, Christoph; Hinsch, Andrea; Wittmer, Corinna; Schlomm, Thorsten; Huland, Hartwig; Heinzer, Hans; Graefen, Markus; Haese, Alexander; Minner, Sarah; Simon, Ronald; Sauter, Guido; Wilczak, Waldemar; Meiners, Jan	INTERNATIONAL JOURNAL OF CANCER	2020	147	2	0	0

Harnessing cell-free DNA: plasma circulating tumour DNA for liquid biopsy in genitourinary cancers	Maia, Manuel Caitano; Salgia, Meghan; Pal, Sumanta K.	NATURE REVIEWS UROLOGY	2020	17	5	0	0
Glandular orientation and shape determined by computational pathology could identify aggressive tumor for early colon carcinoma: a triple-center study	Ji, Meng-Yao; Yuan, Lei; Lu, Shi-Min; Gao, Meng-Ting; Zeng, Zhi; Zhan, Na; Ding, Yi-Juan; Liu, Zheng-Ru; Huang, Ping-Xiao; Lu, Cheng; Dong, Wei-Guo	JOURNAL OF TRANSLATIONAL MEDICINE	2020	18	1	0	0
Network-based multi-task learning models for biomarker selection and cancer outcome prediction	Wang, Zhibo; He, Zhezhi; Shah, Milan; Zhang, Teng; Fan, Deliang; Zhang, Wei	BIOINFORMATICS	2020	36	6	0	0
The role of actinin-4 (ACTN4) in exosomes as a potential novel therapeutic target in castration-resistant prostate cancer	Ishizuya, Yu; Uemura, Motohide; Narumi, Ryohei; Tomiyama, Eisuke; Koh, Yoko; Matsushita, Makoto; Nakano, Kosuke; Hayashi, Yujiro; Wang, Cong; Kato, Taigo; Hatano, Koji; Kawashima, Atsunari; Ujike, Takeshi; Fujita, Kazutoshi; Imamura, Ryoichi; Adachi, Jun; Tomonaga, Takeshi; Nonomura, Norio	BIOCHEMICAL AND BIOPHYSICAL RESEARCH COMMUNICATIONS	2020	523	3	0	0
Pathway-guided analysis identifies Myc-dependent alternative pre-mRNA splicing in aggressive prostate cancers	Phillips, John W.; Pan, Yang; Tsai, Brandon L.; Xie, Zhijie; Demirdjian, Levon; Xiao, Wen; Yang, Harry T.; Zhang, Yida; Lin, Chia Ho; Cheng, Donghui; Hu, Qiang; Liu, Song; Black, Douglas L.; Witte, Owen N.; Xing, Yi	PROCEEDINGS OF THE NATIONAL ACADEMY OF SCIENCES OF THE UNITED STATES OF AMERICA	2020	117	10	0	0
Construction of a replication-competent retroviral vector for expression of the VSV-G envelope glycoprotein for cancer gene therapy	Jin, Sae Young; Jung, Yong-Tae	ARCHIVES OF VIROLOGY	2020	165	5	0	0

CNV Radar: an improved method for somatic copy number alteration characterization in oncology	Soong, David; Stratford, Jeran; Avet-Loiseau, Herve; Bahlis, Nizar; Davies, Faith; Dispenzieri, Angela; Sasser, A. Kate; Schecter, Jordan M.; Qi, Ming; Brown, Chad; Jones, Wendell; Keats, Jonathan J.; Auclair, Daniel; Chiu, Christopher; Powers, Jason; Schaffer, Michael	BMC BIOINFORMATICS	2020	21	1	0	0
Immunotherapy for castration-resistant prostate cancer: has its time arrived?	Slovin, Susan F.	EXPERT OPINION ON BIOLOGICAL THERAPY	2020	20	5	0	0
Upregulation of the transcription factor TFAP2D is associated with aggressive tumor phenotype in prostate cancer lacking the TMPRSS2:ERG fusion	Fraune, Christoph; Harms, Luisa; Buescheck, Franziska; Hoeflmayer, Doris; Tsourlakis, Maria Christina; Clauditz, Till S.; Simon, Ronald; Moeller, Katharina; Luebke, Andreas M.; Moeller-Koop, Christina; Steurer, Stefan; Hube-Magg, Claudia; Sauter, Guido; Weidemann, Soeren; Lebok, Patrick; Dum, David; Kind, Simon; Minner, Sarah; Izbicki, Jakob R.; Schlomm, Thorsten; Huland, Hartwig; Heinzer, Hans; Burandt, Eike; Haese, Alexander; Graefen, Markus; Schroeder, Cornelia	MOLECULAR MEDICINE	2020	26	1	0	0
Androgen receptor variant-driven prostate cancer II: advances in laboratory investigations	Lu, Changxue; Brown, Landon C.; Antonarakis, Emmanuel S.; Armstrong, Andrew J.; Luo, Jun	PROSTATE CANCER AND PROSTATIC DISEASES	2020			0	0
Knowing what's growing: Why ductal and intraductal prostate cancer matter	Lawrence, Mitchell G.; Porter, Laura H.; Clouston, David; Murphy, Declan G.; Frydenberg, Mark; Taylor, Renea A.; Risbridger, Gail P.	SCIENCE TRANSLATIONAL MEDICINE	2020	12	533	0	0
Mutational and transcriptomic landscapes of a rare human prostate basal cell carcinoma	Su, Xianbin; Long, Qi; Bo, Juanjie; Shi, Yi; Zhao, Li-Nan; Lin, Yingxin; Luo, Qing; Ghazanfar, Shila; Zhang, Chao; Liu, Qiang; Wang, Lan; He, Kunyan; He, Jian; Cui, Xiaofang; Yang, Jean Y. H.; Han, Ze-Guang; Yang, Guoliang; Sha, Jian-jun	PROSTATE	2020	80	6	0	0

Exosomes are the Driving Force in Preparing the Soil for the Metastatic Seeds: Lessons from the Prostate Cancer	Saber, Saber H.; Ali, Hamdy E. A.; Gaballa, Rofaida; Gaballah, Mohamed; Ali, Hamed, I; Zerfaoui, Mourad; Abd Elmageed, Zakaria Y.	CELLS	2020	9	3	0	0
The Oncogenic Potential of the Centromeric Border Protein FAM84B of the 8q24.21 Gene Desert	Gu, Yan; Lin, Xiaozeng; Kapoor, Anil; Chow, Mathilda Jing; Jiang, Yanzhi; Zhao, Kuncheng; Tang, Damu	GENES	2020	11	3	0	0
TMPRSS2-Erg/AR-V7: Prognostic value of tests in urine and biopsy rince material in prostate cancer	Plante, G.; Bories, P-N; Denjean, L.; Pigat, N.; Sibony, M.; Goffin, V; Delongchamps, N. Barry	PROGRES EN UROLOGIE	2020	30	3	0	0
Isoquinoline thiosemicarbazone displays potent anticancer activity with in vivo efficacy against aggressive leukemias	Sun, Daniel L.; Poddar, Soumya; Pan, Roy D.; Rosser, Ethan W.; Abt, Evan R.; Van Valkenburgh, Juno; Le, Thuc M.; Lok, Vincent; Hernandez, Selena P.; Song, Janet; Li, Joanna; Turlik, Aneta; Chen, Xiaohong; Cheng, Chi-An; Chen, Wei; Mona, Christine E.; Stuparu, Andreea D.; Vergnes, Laurent; Reue, Karen; Damoiseaux, Robert; Zink, Jeffrey I.; Czernin, Johannes; Donahue, Timothy R.; Houk, Kendall N.; Jung, Michael E.; Radu, Caius G.	RSC MEDICINAL CHEMISTRY	2020	11	3	0	0
Human prostasomes an extracellular vesicle - Biomarkers for male infertility and prostrate cancer: The journey from identification to current knowledge	Vickram, A. S.; Samad, Hari Abdul; Latheef, Shyma K.; Chakraborty, Sandip; Dhama, Kuldeep; Sridharan, T. B.; Sundaram, Thanigaivel; Gulothungan, G.	INTERNATIONAL JOURNAL OF BIOLOGICAL MACROMOLECULES	2020	146		0	0
Radiation Biomarkers: Can Small Businesses Drive Accurate Radiation Precision Medicine?	Prasanna, Pataje G. S.; Narayanan, Deepa; Zhang, Kehui; Rahbar, Amir; Coleman, C. Norman; Vikram, Bhadrasain	RADIATION RESEARCH	2020	193	3	0	0
Systematic characterization of chromatin modifying enzymes identifies KDM3B as a critical regulator in castration resistant prostate cancer	Sarac, Hilal; Morova, Tunc; Pires, Elisabete; McCullagh, James; Kaplan, Anil; Cingoz, Ahmet; Bagci-Onder, Tugba; onder, Tamer; Kawamura, Akane; Lack, Nathan A.	ONCOGENE	2020	39	10	0	0

Targeted next-generation sequencing for locally advanced prostate cancer in the Korean population	Suh, Jungyo; Jeong, Chang Wook; Choi, Seongmin; Ku, Ja Hyeon; Kim, Hyeon Hoe; Kim, Kwang Soo; Kwak, Cheol	INVESTIGATIVE AND CLINICAL UROLOGY	2020	61	2	0	0
Updates in Histologic Grading of Urologic Neoplasms	Rice-Stitt, Travis; Valencia-Guerrero, Aida; Cornejo, Kristine M.; Wu, Chin-Lee	ARCHIVES OF PATHOLOGY & LABORATORY MEDICINE	2020	144	3	0	0
Identification of Novel Prognosis and Prediction Markers in Advanced Prostate Cancer Tissues Based on Quantitative Proteomics	Kwon, Oh Kwang; Ha, Yun-Sok; Na, Ann-Yae; Chun, So Young; Kwon, Tae Gyun; Lee, Jun Nyung; Lee, Sangkyu	CANCER GENOMICS & PROTEOMICS	2020	17	2	0	0
The influence of single-nucleotide polymorphisms on overall survival and toxicity in cabazitaxel-treated patients with metastatic castration-resistant prostate cancer	Belderbos, Bodine P. S.; De With, Mirjam; Singh, Rajbir K.; Agema, Bram C.; El Bouazzaoui, Samira; Oomen-de Hoop, Esther; De Wit, Ronald; Van Schaik, Ron H. N.; Mathijssen, Ron H. J.; Bins, Sander	CANCER CHEMOTHERAPY AND PHARMACOLOGY	2020	85	3	0	0
SIRPB1 promotes prostate cancer cell proliferation via Akt activation	Song, Qiong; Qin, Siyuan; Pascal, Laura E.; Zou, Chunlin; Wang, Wenchu; Tong, Haibo; Zhang, Jian; Catalona, William J.; Dhir, Rajiv; Morrell, Megan; Balasubramani, Goundappa K.; Lu, Yi; Wang, Zhou	PROSTATE	2020	80	4	0	0
Integrative Analysis of MicroRNA and Gene Interactions for Revealing Candidate Signatures in Prostate Cancer	Wei, Jingchao; Yin, Yinghao; Deng, Qiancheng; Zhou, Jun; Wang, Yong; Yin, Guangming; Yang, Jianfu; Tang, Yuxin	FRONTIERS IN GENETICS	2020	11		0	0
Androgen receptor variant-driven prostate cancer II: advances in clinical investigation	Brown, Landon C.; Lu, Changxue; Antonarakis, Emmanuel S.; Luo, Jun; Armstrong, Andrew J.	PROSTATE CANCER AND PROSTATIC DISEASES	2020			0	0
An Automatable Hydrogel Culture Platform for Evaluating Efficacy of Antibody-Based Therapeutics in Overcoming Chemoresistance	Kletzmayr, Anna; Clement Frey, Flurina; Zimmermann, Miriam; Eberli, Daniel; Millan, Christopher	BIOTECHNOLOGY JOURNAL	2020	15	5	0	0

Multiparametric magnetic resonance imaging of multifocal prostate cancer to reveal intra-prostatic genomic heterogeneity and novel radio-genomic correlates: Results of the Smarter Prostate Interventions and Therapeutics (SPIRIT) study	Bauman, Glenn; Correa, Rohann; Aref-Eshghi, Erfan; Alfano, Ryan; Sadikovic, Bekim; Bartlett, John; Boutros, Paul Christopher; Pautler, Stephen E.; Chin, Joseph; Gaed, Mena; Kassam, Zahra; Lemus, Jose Gomez; Moussa, Madeleine; Ward, Aaron	JOURNAL OF CLINICAL ONCOLOGY	2020	38	6	0	0
Circulating tumor cell (CTC) genomic signatures of hormone therapy resistance in men with metastatic castration-resistant prostate cancer (mCRPC)	Gupta, Santosh; Halabi, Susan; Kemeny, Gabor; Anand, Monika; Nanus, David M.; Giannakakou, Paraskevi; George, Daniel J.; Gregory, Simon; Armstrong, Andrew J.	JOURNAL OF CLINICAL ONCOLOGY	2020	38	6	0	0
Comprehensive genomic profiling (CGP) of metastatic castrate-sensitive prostate cancer (mCSPC) to reveal potential biomarkers and therapeutic targets	Jiang, Di Maria; Wong, Bryan; Hansen, Aaron Richard; Fallah-Rad, Nazanin; Sacher, Adrian G.; Zhang, Tong; Selvarajah, Shamini; Stockley, Tracy; Bedard, Philippe L.; Sridhar, Srikala S.	JOURNAL OF CLINICAL ONCOLOGY	2020	38	6	0	0
A tale of lineage plasticity: Intense neoadjuvant testosterone lowering therapy in localized prostate cancer (PCa) harboring high-risk genomic signatures	Karzai, Fatima; Madan, Ravi Amrit; Sowalsky, Adam G.; Bilusic, Marijo; Chun, Guinevere; Cordes, Lisa M.; Wilkinson, Scott C.; Terrigino, Nicholas; Harmon, Stephanie; Pinto, Peter A.; Choyke, Peter L.; Turkbey, Baris; Gulley, James L.; Dahut, William L.	JOURNAL OF CLINICAL ONCOLOGY	2020	38	6	0	0
Clinical-genomic sub-classification of high-risk prostate cancer: Implications for tailoring therapy and clinical trial design	Muralidhar, Vinayak; Alshalalfa, Mohammed; Spratt, Daniel Eidelberg; Liu, Yang; Karnes, R. Jeffrey; Schaeffer, Edward M.; Davicioni, Elai; Feng, Felix Y.; Klein, Eric A.; Tosoian, Jeffrey J.; Berlin, Alejandro; Den, Robert Benjamin; Nguyen, Paul L.	JOURNAL OF CLINICAL ONCOLOGY	2020	38	6	0	0
Recurrence-Associated Multi-RNA Signature to Predict Disease-Free Survival for Ovarian Cancer Patients	Zhang, Yu; Ye, Qingjian; He, Junxian; Chen, Peigen; Wan, Jing; Li, Jing; Yang, Yuebo; Li, Xiaomao	BIOMED RESEARCH INTERNATIONAL	2020	2020		0	0

Pan-cancer clinical and molecular analysis of racial disparities	Lara, Olivia D.; Wang, Ying; Asare, Amma; Xu, Tao; Chiu, Hua-Sheng; Liu, Yuexin; Hu, Wei; Sumazin, Pavel; Uppal, Shitanshu; Zhang, Lin; Rauh-Hain, J. Alejandro; Sood, Anil K.	CANCER	2020	126	4	0	0
The Rare Variant rs35356162 in UHRF1BP1 Increases Bladder Cancer Risk in Han Chinese Population	Wu, Junlong; Wang, Meilin; Chen, Haitao; Xu, Jianfeng; Zhang, Guiming; Gu, Chengyuan; Ding, Qiang; Wei, Qingyi; Zhu, Yao; Ye, Dingwei	FRONTIERS IN ONCOLOGY	2020	10		0	0
Characterising a human endogenous retrovirus(HERV)-derived tumour-associated antigen: enriched RNA-Seq analysis of HERV-K(HML-2) in mantle cell lymphoma cell lines	Tatkiewicz, Witold; Dickie, James; Bedford, Franchesca; Jones, Alexander; Atkin, Mark; Kiernan, Michele; Maze, Emmanuel Atangana; Agit, Bora; Farnham, Garry; Kanapin, Alexander; Belshaw, Robert	MOBILE DNA	2020	11	1	0	0
A Three-Gene Classifier Associated With MicroRNA-Mediated Regulation Predicts Prostate Cancer Recurrence After Radical Prostatectomy	Cheng, Bo; He, Qidan; Cheng, Yong; Yang, Haifan; Pei, Lijun; Deng, Qingfu; Long, Hao; Zhu, Likun; Jiang, Rui	FRONTIERS IN GENETICS	2020	10		0	0
Decreased glucose bioavailability and elevated aspartate metabolism in prostate cancer cells undergoing epithelial-mesenchymal transition	Chen, Yule; Wang, Ke; Liu, Tianjie; Chen, Jiaqi; Lv, Wei; Yang, Wenjie; Xu, Shan; Wang, Xinyang; Li, Lei	JOURNAL OF CELLULAR PHYSIOLOGY	2020	235	7-8	0	0
Genome-wide analysis of HOXC4 and HOXC6 regulated genes and binding sites in prostate cancer cells	Luo, Zhifei; Farnham, Peggy J.	PLOS ONE	2020	15	2	0	0
Leukocyte telomere length is associated with aggressive prostate cancer in localized prostate cancer patients	Xu, Junfeng; Chang, Wen-Shin; Tsai, Chia-Wen; Bau, Da-Tian; Xu, Yifan; Davis, John W.; Thompson, Timothy C.; Logothetis, Christopher J.; Gu, Jian	EBIOMEDICINE	2020	52		0	0
A risk prediction model of DNA methylation improves prognosis evaluation and indicates gene targets in prostate cancer	Zhang, Enchong; Hou, Xueying; Hou, Baoxian; Zhang, Mo; Song, Yongsheng	EPIGENOMICS	2020	12	4	0	0

Variants in the 8q24 region associated with risk of breast cancer Systematic research synopsis and meta-analysis	Wang Xuedong; He Xian; Guo Hui; Tong Yu	MEDICINE	2020	99	8	0	0
The miR-28-5p Targetome Discovery Identified SREBF2 as One of the Mediators of the miR-28-5p Tumor Suppressor Activity in Prostate Cancer Cells	Fazio, Sofia; Berti, Gabriele; Russo, Francesco; Evangelista, Monica; D'Aurizio, Romina; Mercatanti, Alberto; Pellegrini, Marco; Rizzo, Milena	CELLS	2020	9	2	0	0
A Comprehensive Analysis of FUT8 Overexpressing Prostate Cancer Cells Reveals the Role of EGFR in Castration Resistance	Hoti, Naseruddin; Lih, Tung-Shing; Pan, Jianbo; Zhou, Yangying; Yang, Ganglong; Deng, Ashely; Chen, Lijun; Dong, Mingmimg; Yang, Ruey-Bing; Tu, Cheng-Fen; Haffner, Michael C.; Li, Qing Kay; Zhang, Hui	CANCERS	2020	12	2	0	0
The Urinary Transcriptome as a Source of Biomarkers for Prostate Cancer	Sole, Carla; Goicoechea, Ibai; Goni, Alai; Schramm, Maike; Armesto, Maria; Arestin, Maria; Manterola, Lorea; Tellaetxe, Maitena; Alberdi, Aitor; Nogueira, Leonor; Roumiguie, Mathieu; Ignacio Lopez, Jose; Sanz Jaka, Juan Pablo; Urruticoechea, Ander; Vergara, Itziar; Loizaga-Iriarte, Ana; Unda, Miguel; Carracedo, Arkaitz; Malavaud, Bernard; Lawrie, Charles H.	CANCERS	2020	12	2	0	0
MiRNA-Based Inspired Approach in Diagnosis of Prostate Cancer	Munteanu, Vlad Cristian; Munteanu, Raluca Andrada; Onaciu, Anca; Berindan-Neagoe, Ioana; Petrut, Bogdan; Coman, Ioan	MEDICINA-LITHUANIA	2020	56	2	0	0
Screening key miRNAs and genes in prostate cancer by microarray analysis	Wu, Jianhui; Li, Xuemei; Luo, Fei; Yan, Jun; Yang, Kuo	TRANSLATIONAL CANCER RESEARCH	2020	9	2	0	0
Biomarker selection and imaging design in cancer: A link with biochemical pathways for imminent engineering	Ali, Joham Surfraz; ul Ain, Noor; Naz, Sania; Zia, Muhammad	HELIYON	2020	6	2	0	0

How and when to refer patients for oncogenetic counseling in the era of PARP inhibitors	Neviere, Zoe; Rouge, Thibault De La Motte; Floquet, Anne; Johnson, Alison; Berthet, Pascaline; Joly, Florence	THERAPEUTIC ADVANCES IN MEDICAL ONCOLOGY	2020	12		0	0
USP22 Functions as an Oncogenic Driver in Prostate Cancer by Regulating Cell Proliferation and DNA Repair	McCann, Jennifer J.; Vasilevskaya, Irina A.; Neupane, Neermala Poudel; Shafi, Ayesha A.; McNair, Christopher; Dylgjeri, Emanuela; Mandigo, Amy C.; Schiewer, Matthew; Schrecengost, Randy S.; Gallagher, Peter; Stanek, Timothy J.; Mcmahon, Steven B.; Berman-Booty, Lisa D.; Ostrander, William F., Jr.; Knudsen, Karen E.	CANCER RESEARCH	2020	80	3	0	0
Androgen Receptor Splice Variant, AR-V7, as a Biomarker of Resistance to Androgen Axis-Targeted Therapies in Advanced Prostate Cancer	Zhang, Tian; Karsh, Lawrence I.; Nissenblatt, Michael J.; Canfield, Steven E.	CLINICAL GENITOURINARY CANCER	2020	18	1	0	0
Enzalutamide in Combination with Abiraterone Acetate in Bone Metastatic Castration-resistant Prostate Cancer Patients	Efstathiou, Eleni; Titus, Mark; Wen, Sijin; Troncoso, Patricia; Hoang, Anh; Corn, Paul; Prokhorova, Ina; Araujo, John; Dmuchowski, Carl; Melhem-Bertrandt, Amal; Patil, Shiva; Logothetis, Christopher J.	EUROPEAN UROLOGY ONCOLOGY	2020	3	1	0	0
Correlation between mouse age and human age in anti-tumor research: Significance and method establishment	Wang, Shuo; Lai, Xiaoxue; Deng, Yihui; Song, Yanzhi	LIFE SCIENCES	2020	242		0	0
The ternary complex factor protein ELK1 is an independent prognosticator of disease recurrence in prostate cancer	Pardy, Luke; Rosati, Rayna; Soave, Claire; Huang, Yanfang; Kim, Seongho; Ratnam, Manohar	PROSTATE	2020	80	2	0	0

GC-MS-Based Metabolomic Profiles Combined with Chemometric Tools and Cytotoxic Activities of Non-Polar Leaf Extracts of Spondias mombin L. and Spondias tuberosa Arr. Cam.	Guedes, Jhonyson A. C.; Alves Filho, Elenilson G.; Silva, Maria F. S.; Rodrigues, Tigressa H. S.; Ramires, Christian M. C.; Lima, Maria A. C.; Silva, Gisele S.; Pessoa, Claudia O.; Canuto, Kirley M.; Brito, Edy S.; Alves, Ricardo E.; Nascimento, Ronaldo F.; Zocolo, Guilherme J.	JOURNAL OF THE BRAZILIAN CHEMICAL SOCIETY	2020	31	2	0	0
Evaluating liquid biopsies for methylomic profiling of prostate cancer	Silva, Romina; Moran, Bruce; Russell, Niamh M.; Fahey, Ciara; Vlajnic, Tatjana; Manecksha, Rustom P.; Finn, Stephen P.; Brennan, Donal J.; Gallagher, William M.; Perry, Antoinette S.	EPIGENETICS	2020			0	0
Pairing Microwell Arrays with an Affordable, Semiautomated Single-Cell Aspirator for the Interrogation of Circulating Tumor Cell Heterogeneity	Tokar, Jacob J.; Stahlfeld, Charlotte N.; Sperger, Jamie M.; Niles, David J.; Beebe, David J.; Lang, Joshua M.; Warrick, Jay W.	SLAS TECHNOLOGY	2020	25	2	0	0
miR-210 is induced by hypoxia and regulates neural cell adhesion molecule in prostate cells	Angel, Charlotte Zoe; Lynch, Seodhna M.; Nesbitt, Heather; McKenna, Michael M.; Walsh, Colum P.; McKenna, Declan J.	JOURNAL OF CELLULAR PHYSIOLOGY	2020	235	9	0	0
Sulfiredoxin as a Potential Therapeutic Target for Advanced and Metastatic Prostate Cancer	Barquilha, Caroline N.; Santos, Nilton J.; Moncao, Caio C. D.; Barbosa, Isabela C.; Lima, Flavio O.; Justulin, Luis A.; Pertega-Gomes, Nelma; Felisbino, Sergio L.	OXIDATIVE MEDICINE AND CELLULAR LONGEVITY	2020	2020		0	0
Unleashing the full potential of Hsp90 inhibitors as cancer therapeutics through simultaneous inactivation of Hsp90, Grp94, and TRAP1	Park, Hye-Kyung; Yoon, Nam Gu; Lee, Ji-Eun; Hu, Sung; Yoon, Sora; Kim, So Yeon; Hong, Jun-Hee; Nam, Dougu; Chae, Young Chan; Park, Jong Bae; Kang, Byoung Heon	EXPERIMENTAL AND MOLECULAR MEDICINE	2020			0	0
Active surveillance in intermediate-risk prostate cancer	Klotz, Laurence	BJU INTERNATIONAL	2020	125	3	0	0

The ETS transcription factor ETV5 is a target of activated ALK in neuroblastoma contributing to increased tumour aggressiveness	Mus, Liselot M.; Lambertz, Irina; Claeys, Shana; Kumps, Candy; Van Loocke, Wouter; Van Neste, Christophe; Umapathy, Ganesh; Vaapil, Marica; Bartenhagen, Christoph; Laureys, Genevieve; De Wever, Olivier; Bexell, Daniel; Fischer, Matthias; Hallberg, Bengt; Schulte, Johannes; De Wilde, Bram; Durinck, Kaat; Denecker, Geertrui; De Preter, Katleen; Speleman, Frank	SCIENTIFIC REPORTS	2020	10	1	0	0
Aggressive prostate cancer with somatic loss of the homologous recombination repair gene FANCA: a case report	Hongo, Hiroshi; Kosaka, Takeo; Aimono, Eriko; Nishihara, Hiroshi; Oya, Mototsugu	DIAGNOSTIC PATHOLOGY	2020	15	1	0	0
DNA hypermethylation associated with upregulated gene expression in prostate cancer demonstrates the diversity of epigenetic regulation	Rauluseviciute, Ieva; Drablos, Finn; Rye, Morten Beck	BMC MEDICAL GENOMICS	2020	13	1	0	0
Genomic and clinical characterization of stromal infiltration markers in prostate cancer	Mahal, Brandon A.; Alshalalfa, Mohammed; Zhao, Shuang G.; Beltran, Himisha; Chen, William S.; Chipidza, Fallon; Davicioni, Elai; Karnes, R. Jeffrey; Ku, Sheng-Yu; Lotan, Tamara L.; Muralidhar, Vinayak; Rebbeck, Timothy R.; Schaeffer, Edward M.; Spratt, Daniel E.; Feng, Felix Y.; Nguyen, Paul L.	CANCER	2020	126	7	0	0
Metabolic characteristics of large and small extracellular vesicles from pleural effusion reveal biomarker candidates for the diagnosis of tuberculosis and malignancy	Luo, Ping; Mao, Kaimin; Xu, Juanjuan; Wu, Feng; Wang, Xuan; Wang, Sufei; Zhou, Mei; Duan, Limin; Tan, Qi; Ma, Guangzhou; Yang, Guanghai; Du, Ronghui; Huang, Hai; Huang, Qi; Li, Yumei; Guo, Mengfei; Jin, Yang	JOURNAL OF EXTRACELLULAR VESICLES	2020	9	1	0	0
Comprehensive palmitoyl-proteomic analysis identifies distinct protein signatures for large and small cancer-derived extracellular vesicles	Mariscal, Javier; Vagner, Tatyana; Kim, Minhyung; Zhou, Bo; Chin, Andrew; Zandian, Mandana; Freeman, Michael R.; You, Sungyong; Zijlstra, Andries; Yang, Wei; Di Vizio, Dolores	JOURNAL OF EXTRACELLULAR VESICLES	2020	9	1	0	0

The Identification of Key Gene Expression Signature in Prostate Cancer	Yu, Huang; Qi, Cao; Song Zhengshuai; Ruan Hailong; Wang Keshan; Ke, Chen; Zhang Xiaoping	CRITICAL REVIEWS IN EUKARYOTIC GENE EXPRESSION	2020	30	2	0	0
Therapeutic Potential of Medicinal Plant Proteins: Present Status and Future Perspectives	Wani, Snober Shabeer; Dar, Parvaiz A.; Zargar, Sajad M.; Dar, Tanveer A.	CURRENT PROTEIN & PEPTIDE SCIENCE	2020	21	5	0	0
Upregulation of long non-coding RNA ATP6V0E2-AS1 predicts a poor prognosis in prostate cancer	Hu, Shengguo; Li, Youkong; Zhu, Min	INTERNATIONAL JOURNAL OF CLINICAL AND EXPERIMENTAL MEDICINE	2020	13	5	0	0
The interactome of the prostate-specific protein Anoctamin 7	Kaikkonen, Elina; Takala, Aliisa; Pursiheimo, Juha-Pekka; Wahlstrom, Gudrun; Schleutker, Johanna	CANCER BIOMARKERS	2020	28	1	0	0
The Power of Phytochemicals Combination in Cancer Chemoprevention	Rizeq, Balsam; Gupta, Ishita; Ilesanmi, Josephine; AlSafran, Mohammed; Rahman, Mizanur; Ouhtit, Allal	JOURNAL OF CANCER	2020	11	15	0	0
Circulating let-7f-5p improve risk prediction of prostate cancer in patients with benign prostatic hyperplasia	Ge, Yuqiu; Wang, Qiangdong; Shao, Wei; Zhao, You; Shi, Qianqian; Yuan, Qinbo; Cui, Li	JOURNAL OF CANCER	2020	11	15	0	0
BAP1 functions as a tumor promoter in prostate cancer cells through EMT regulation	Park, Chan Mi; Lee, Jac Eun; Kim, Jung Hwa	GENETICS AND MOLECULAR BIOLOGY	2020	43	2	0	0
Personalized Treatment Approach to Metastatic Castration-Resistant Prostate Cancer with BRCA2 and PTEN Mutations: A Case Report	Julka, Pramod Kumar; Verma, Amit; Gupta, Kush	CASE REPORTS IN ONCOLOGY	2020	13	1	0	0
Ubiquitin, SUMO, and Nedd8 as Therapeutic Targets in Cancer	Gatel, Pierre; Piechaczyk, Marc; Bossis, Guillaume	PROTEOSTASIS AND DISEASE: FROM BASIC MECHANISMS TO CLINICS	2020	1233		0	0

Combined signature of nine immune-related genes: a novel risk score for predicting prognosis in hepatocellular carcinoma	Tang, Yunliang; Zeng, Zhenguo; Wang, Jiao; Li, Guoyong; Huang, Chao; Dong, Xiaoyang; Feng, Zhen	AMERICAN JOURNAL OF TRANSLATIONAL RESEARCH	2020	12	4	0	0
Integrin-associated CD151 is a suppressor of prostate cancer progression	Han, Rongbo; Hensley, Patrick J.; Li, Jieming; Zhang, Yang; Stark, Timothy W.; Heller, Allie; Qian, Hai; Shi, Junfeng; Liu, Zeyi; Huang, Jian-An; Jin, Tengchuan; Wei, Xiaowei; Zhou, Binhua P.; Wu, Yadi; Kyprianou, Natasha; Chen, Jinfei; Yang, Xiuwei H.	AMERICAN JOURNAL OF TRANSLATIONAL RESEARCH	2020	12	4	0	0
Discovering Therapeutic Protein Targets for Bladder Cancer Using Proteomic Data Analysis	Bahrami, Samira; Kazemi, Bahram; Zali, Hakimeh; Black, Peter C.; Basiri, Abbas; Bandehpour, Mojgan; Hedayati, Mehdi; Sahebkar, Amirhossein	CURRENT MOLECULAR PHARMACOLOGY	2020	13	2	0	0
miR-489-3p Inhibits Prostate Cancer Progression by Targeting DLX1	Bai, Peide; Li, Wei; Wan, Zhenghua; Xiao, Yujuan; Xiao, Wen; Wang, Xuegang; Wu, Zhun; Zhang, Kaiyan; Wang, Yongfeng; Chen, Bin; Xing, Jinchun; Wang, Tao	CANCER MANAGEMENT AND RESEARCH	2020	12		0	0
Identification of key genes involved in the pathogenesis of cutaneous melanoma using bioinformatics analysis	Chen, Jianqin; Sun, Wen; Mo, Nian; Chen, Xiangjun; Yang, Lihong; Tu, Shaozhong; Zhang, Siwen; Liu, Jing	JOURNAL OF INTERNATIONAL MEDICAL RESEARCH	2020	48	1	0	0
Transcriptomic analysis reveals the oncogenic role of S6K1 in hepatocellular carcinoma	Lai, Keng Po; Cheung, Angela; Ho, Cheuk Hin; Tam, Nathan Yi-Kan; Li, Jing Woei; Lin, Xiao; Chan, Ting Fung; Lee, Nikki Pui-Yue; Li, Rong	JOURNAL OF CANCER	2020	11	9	0	0
Downregulation of PSCA promotes gastric cancer proliferation and is related to poor prognosis	Xu, Li-pu; Qiu, Hai-bo; Yuan, Shu-qiang; Chen, Yong-ming; Zhou, Zhi-wei; Chen, Ying-bo	JOURNAL OF CANCER	2020	11	9	0	0
Use of miRNA as a Biomarker in Prostate Cancer and New Approaches	Aslan, Elif Sibel; Cetinkaya, Sefa	TURK ONKOLOJI DERGISI-TURKISH JOURNAL OF ONCOLOGY	2020	35	1	0	0

Integrative Exome Sequencing Analysis in Castration-Resistant Prostate Cancer in Chinese Population	Hao, Lifang; Li, Hui; Zhang, Su; Yang, Yanlei; Xu, Zhenzhen; Zhang, Yanfen; Liu, Zhongcheng	CURRENT PHARMACEUTICAL BIOTECHNOLOGY	2020	21	2	0	0
Genomic Strategies to Personalize Use of Androgen Deprivation Therapy With Radiotherapy	Glicksman, Rachel M.; Berlin, Alejandro	CANCER JOURNAL	2020	26	1	0	0
Unleashing the full potential of Hsp90 inhibitors as cancer therapeutics through simultaneous inactivation of Hsp90, Grp94, and TRAP1	Park, Hye-Kyung; Yoon, Nam Gu; Lee, Ji-Eun; Hu, Sung; Yoon, Sora; Kim, So Yeon; Hong, Jun-Hee; Nam, Dougu; Chae, Young Chan; Park, Jong Bae; Kang, Byoung Heon	EXPERIMENTAL AND MOLECULAR MEDICINE	2020	52	1	0	0
PTEN deletion drives aberrations of DNA methylome and transcriptome in different stages of prostate cancer	Wang, Chao; Feng, Yaping; Zhang, Chengyue; Cheng, David; Wu, Renyi; Yang, Yuqing; Sargsyan, Davit; Kumar, Dibyendu; Kong, Ah-Ng	FASEB JOURNAL	2020	34	1	0	0
Germline variants and response to systemic therapy in advanced prostate cancer	Johnson, Eric; Nussenzveig, Roberto; Agarwal, Neeraj; Swami, Umang	PHARMACOGENO MICS	2020	21	1	0	0
Identification of novel alternative transcripts of the human Ribonuclease kappa (RNASEK) gene using 3 ' RACE and high-throughput sequencing approaches	Adamopoulos, Panagiotis G.; Kontos, Christos K.; Scorilas, Andreas; Sideris, Diamantis C.	GENOMICS	2020	112	1	0	0
Prognostic implication and functional annotations of Rad50 expression in patients with prostate cancer	Xu, Wen-Hao; Wang, Jun; Sheng, Hao-Yue; Qu, Yuan-Yuan; Wang, Hong-Kai; Zhu, Yu; Shi, Guo-Hai; Zhang, Hai-Liang; Ye, Ding-Wei	JOURNAL OF CELLULAR BIOCHEMISTRY	2020	121	5-6	0	0
Marked response to cabazitaxel in prostate cancer xenografts expressing androgen receptor variant 7 and reversion of acquired resistance by anti-androgens	Ylitalo, Erik Bovinder; Thysell, Elin; Thellenberg-Karlsson, Camilla; Lundholm, Marie; Widmark, Anders; Bergh, Anders; Josefsson, Andreas; Brattsand, Maria; Wikstrom, Pernilla	PROSTATE	2020	80	2	0	0

Integrative clinical transcriptome analysis reveals TMPRSS2-ERG dependency of prognostic biomarkers in prostate adenocarcinoma	Gerke, Julia S.; Orth, Martin F.; Tolkach, Yuri; Romero-Perez, Laura; Wehweck, Fabienne S.; Stein, Stefanie; Musa, Julian; Knott, Maximilian M. L.; Hoelting, Tilman L. B.; Li, Jing; Sannino, Giuseppina; Marchetto, Aruna; Ohmura, Shunya; Cidre-Aranaz, Florencia; Mueller-Nurasyid, Martina; Strauch, Konstantin; Stief, Christian; Kristiansen, Glen; Kirchner, Thomas; Buchner, Alexander; Gruenewald, Thomas G. P.	INTERNATIONAL JOURNAL OF CANCER	2020	146	7	0	0
Prostate cancer and the role of biomarkers	Nevo, Amihay; Navaratnam, Anojan; Andrews, Paul	ABDOMINAL RADIOLOGY	2020	45	7	0	0
High RSF1 protein expression is an independent prognostic feature in prostate cancer	Hoeflmayer, Doris; Hamuda, Moslim; Schroeder, Cornelia; Hube-Magg, Claudia; Simon, Ronald; Goebel, Cosima; Hinsch, Andrea; Weidemann, Soeren; Moeller, Katharina; Izbicki, Jacob R.; Jacobsen, Frank; Mandelkow, Tim; Blessin, Niclas C.; Lutz, Florian; Viehweger, Florian; Sauter, Guido; Burandt, Eike; Lebok, Patrick; Lennartz, Maximillian; Fraune, Christoph; Minner, Sarah; Bonk, Sarah; Huland, Hartwig; Graefen, Markus; Schlomm, Thorsten; Buescheck, Franziska	ACTA ONCOLOGICA	2020	59	3	0	0
Prostate cancer pathology: What has changed in the last 5 years	Cimadamore, Alessia; Scarpelli, Marina; Raspollini, Maria Rosaria; Doria, Andrea; Galosi, Andrea Benedetto; Massari, Francesco; Di Nunno, Vincenzo; Cheng, Liang; Lopez-Beltran, Antonio; Montironi, Rodolfo	UROLOGIA JOURNAL	2020	87	1	0	0
Anticancer Characteristics of Fomitopsis pinicola Extract in a Xenograft Mouse Model-a Preliminary Study	Kao, Chi H. J.; Greenwood, David R.; Jamieson, Stephen M. F.; Coe, Margaret E.; Murray, Pamela M.; Ferguson, Lynnette R.; Bishop, Karen S.	NUTRITION AND CANCER-AN INTERNATIONAL JOURNAL	2020	72	4	0	0
