# Supplementary material for: Omics Derived Biomarkers and Novel Drug Targets for Improved Intervention in Advanced Prostate Cancer
Source: Diagnostics (Basel). 2020 Aug 31;10(9):658. doi: 10.3390/diagnostics10090658 (PMC7555799; doi:10.3390/diagnostics10090658)
Supplement: Supplementary file 1 [file diagnostics-10-00658-s001.zip › Table S2. List of selected manuscripts .pdf]

Table S2. List of selected manuscripts

Title	Authors	Source Title	Publication Year	Volume	Issue	Total Citations	Average per Year
The landscape of long noncoding RNAs in the human transcriptome	Iyer, Matthew K.; Niknafs, Yashar S.; Malik, Rohit; Singhal, Udit; Sahu, Anirban; Hosono, Yasuyuki; Barrette, Terrence R.; Prensner, John R.; Evans, Joseph R.; Zhao, Shuang; Poliakov, Anton; Cao, Xuhong; Dhanasekaran, Saravana M.; Wu, Yi-Mi; Robinson, Dan R.; Beer, David G.; Feng, Felix Y.; Iyer, Hariharan K.; Chinnaiyan, Arul M.	NATURE GENETICS	2015	47	3	1100	183,33
Long Noncoding RNAs in Cancer Pathways	Schmitt, Adam M.; Chang, Howard Y.	CANCER CELL	2016	29	4	1076	215,2
DNA-Repair Defects and Olaparib in Metastatic Prostate Cancer	Mateo, J.; Carreira, S.; Sandhu, S.; Miranda, S.; Mossop, H.; Perez-Lopez, R.; Rodrigues, D. Nava; Robinson, D.; Omlin, A.; Tunariu, N.; Boysen, G.; Porta, N.; Flohr, P.; Gillman, A.; Figueiredo, I.; Paulding, C.; Seed, G.; Jain, S.; Ralph, C.; Protheroe, A.; Hussain, S.; Jones, R.; Elliott, T.; McGovern, U.; Bianchini, D.; Goodall, J.; Zafeiriou, Z.; Williamson, C. T.; Ferraldeschi, R.; Riisnaes, R.; Ebbs, B.; Fowler, G.; Roda, D.; Yuan, W.; Wu, Y. -M.; Cao, X.; Brough, R.; Pemberton, H.; A'Hern, R.; Swain, A.; Kunju, L. P.; Eeles, R.; Attard, G.; Lord, C. J.; Ashworth, A.; Rubin, M. A.; Knudsen, K. E.; Feng, F. Y.; Chinnaiyan, A. M.; Hall, E.; de Bono, J. S.	NEW ENGLAND JOURNAL OF MEDICINE	2015	373	18	935	155,83

The evolutionary history of lethal metastatic prostate cancer	Gundem, Gunes; Van Loo, Peter; Kremeyer, Barbara; Alexandrov, Ludmil B.; Tubio, Jose M. C.; Papaemmanuil, Elli; Brewer, Daniel S.; Kallio, Heini M. L.; Hoegnas, Gunilla; Annala, Matti; Kivinummi, Kati; Goody, Victoria; Latimer, Calli; O'Meara, Sarah; Dawson, Kevin J.; Isaacs, William; Emmert-Buck, Michael R.; Nykter, Matti; Foster, Christopher; Kote-Jarai, Zsofia; Easton, Douglas; Whitaker, Hayley C.; Neal, David E.; Cooper, Colin S.; Eeles, Rosalind A.; Visakorpi, Tapio; Campbell, Peter J.; McDermott, Ultan; Wedge, David C.; Bova, G. Steven	NATURE	2015	520	7547	604	100,67
Divergent clonal evolution of castration-resistant neuroendocrine prostate cancer	Beltran, Himisha; Prandi, Davide; Mosquera, Juan Miguel; Benelli, Matteo; Puca, Loredana; Cyrta, Joanna; Marotz, Clarisse; Giannopoulou, Eugenia; Chakravarthi, Balabhadrapatruni V. S. K.; Varambally, Sooryanarayana; Tomlins, Scott A.; Nanus, David M.; Tagawa, Scott T.; Van Allen, Eliezer M.; Elemento, Olivier; Sboner, Andrea; Garraway, Levi A.; Rubin, Mark A.; Demichelis, Francesca	NATURE MEDICINE	2016	22	3	485	97
Substantial interindividual and limited intraindividual genomic diversity among tumors from men with metastatic prostate cancer	Kumar, Akash; Coleman, Ilsa; Morrissey, Colm; Zhang, Xiaotun; True, Lawrence D.; Gulati, Roman; Etzioni, Ruth; Bolouri, Hamid; Montgomery, Bruce; White, Thomas; Lucas, Jared M.; Brown, Lisha G.; Dumpit, Ruth F.; DeSarkar, Navonil; Higano, Celestia; Yu, Evan Y.; Coleman, Roger; Schultz, Nikolaus; Fang, Min; Lange, Paul H.; Shendure, Jay; Vessella, Robert L.; Nelson, Peter S.	NATURE MEDICINE	2016	22	4	267	53,4

Integrative clinical genomics of metastatic cancer	Robinson, Dan R.; Wu, Yi-Mi; Lonigro, Robert J.; Vats, Pankaj; Cobain, Erin; Everett, Jessica; Cao, Xuhong; Rabban, Erica; Kumar-Sinha, Chandan; Raymond, Victoria; Schuetze, Scott; Alva, Ajjai; Siddiqui, Javed; Chugh, Rashmi; Worden, Francis; Zalupski, Mark M.; Innis, Jeffrey; Mody, Rajen J.; Tomlins, Scott A. .; Lucas, David; Baker, Laurence H.; Ramnath, Nithya; Schott, Ann F.; Hayes, Daniel F.; Vijai, Joseph; Offit, Kenneth; Stoffel, Elena M.; Roberts, J. Scott; Smith, David C.; Kunju, Lakshmi P.; Talpaz, Moshe; Cieslik, Marcin; Chinnaiyan, Arul M.	NATURE	2017	548	7667	202	50,5
Tracking the origins and drivers of subclonal metastatic expansion in prostate cancer	Hong, Matthew K. H.; Macintyre, Geoff; Wedge, David C.; Van Loo, Peter; Patel, Keval; Lunke, Sebastian; Alexandrov, Ludmil B.; Sloggett, Clare; Cmero, Marek; Marass, Francesco; Tsui, Dana; Mangiola, Stefano; Lonie, Andrew; Naeem, Haroon; Sapre, Nikhil; Phal, Pramit M.; Kurganovs, Natalie; Chin, Xiaowen; Kerger, Michael; Warren, Anne Y.; Neal, David; Gnanapragasam, Vincent; Rosenfeld, Nitzan; Pedersen, John S.; Ryan, Andrew; Haviv, Izhak; Costello, Anthony J.; Corcoran, Niall M.; Hovens, Christopher M.	NATURE COMMUNICATIONS	2015	6		189	31,5

A Biopsy-based 17-gene Genomic Prostate Score Predicts Recurrence After Radical Prostatectomy and Adverse Surgical Pathology in a Racially Diverse Population of Men with Clinically Low- and Intermediate-risk Prostate Cancer	Cullen, Jennifer; Rosner, Inger L.; Brand, Timothy C.; Zhang, Nan; Tsiatis, Athanasios C.; Moncur, Joel; Ali, Amina; Chen, Yongmei; Knezevic, Dejan; Maddala, Tara; Lawrence, H. Jeffrey; Febbo, Phillip G.; Srivastava, Shiv; Sesterhenn, Isabell A.; McLeod, David G.	EUROPEAN UROLOGY	2015	68	1	141	23,5
Genomic Classifier Identifies Men With Adverse Pathology After Radical Prostatectomy Who Benefit From Adjuvant Radiation Therapy	Den, Robert B.; Yousefi, Kasra; Trabulsi, Edouard J.; Abdollah, Firas; Choeurng, Voleak; Feng, Felix Y.; Dicker, Adam P.; Lallas, Costas D.; Gomella, Leonard G.; Davicioni, Elai; Karnes, R. Jeffrey	JOURNAL OF CLINICAL ONCOLOGY	2015	33	8	127	21,17
Genomic Predictors of Outcome in Prostate Cancer	Bostrom, Peter J.; Bjartell, Anders S.; Catto, James W. F.; Eggener, Scott E.; Lilja, Hans; Loeb, Stacy; Schalken, Jack; Schlomm, Thorsten; Cooperberg, Matthew R.	EUROPEAN UROLOGY	2015	68	6	103	17,17

Combined Value of Validated Clinical and Genomic Risk Stratification Tools for Predicting Prostate Cancer Mortality in a High-risk Prostatectomy Cohort	Cooperberg, Matthew R.; Davicioni, Elai; Crisan, Anamaria; Jenkins, Robert B.; Ghadessi, Mercedeh; Karnes, R. Jeffrey	EUROPEAN UROLOGY	2015	67	2	102	17
Tissue-based Genomics Augments Post-prostatectomy Risk Stratification in a Natural History Cohort of Intermediate- and High-Risk Men	Ross, Ashley E.; Johnson, Michael H.; Yousefi, Kasra; Davicioni, Elai; Netto, George J.; Marchionni, Luigi; Fedor, Helen L.; Glavaris, Stephanie; Choeurng, Voleak; Buerki, Christine; Erho, Nicholas; Lam, Lucia L.; Humphreys, Elizabeth B.; Faraj, Sheila; Bezerra, Stephania M.; Han, Misop; Partin, Alan W.; Trock, Bruce J.; Schaeffer, Edward M.	EUROPEAN UROLOGY	2016	69	1	102	20,4

A Genomic Classifier Improves Prediction of Metastatic Disease Within 5 Years After Surgery in Node-negative High-risk Prostate Cancer Patients Managed by Radical Prostatectomy Without Adjuvant Therapy	Klein, Eric A.; Yousefi, Kasra; Haddad, Zaid; Choeurng, Voleak; Buerki, Christine; Stephenson, Andrew J.; Li, Jianbo; Kattan, Michael W.; Magi-Galluzzi, Cristina; Davicioni, Elai	EUROPEAN UROLOGY	2015	67	4	96	16
Clinical and Genomic Characterization of Treatment-Emergent Small-Cell Neuroendocrine Prostate Cancer: A Multi-institutional Prospective Study	Aggarwal, Rahul; Huang, Jiaoti; Alumkal, Joshi J.; Zhang, Li; Feng, Felix Y.; Thomas, George V.; Weinstein, Alana S.; Friedl, Verena; Zhang, Can; Witte, Owen N.; Lloyd, Paul; Gleave, Martin; Evans, Christopher P.; Youngren, Jack; Beer, Tomasz M.; Rettig, Matthew; Wong, Christopher K.; True, Lawrence; Foye, Adam; Playdle, Denise; Ryan, Charles J.; Lara, Primo; Chi, Kim N.; Uzunangelov, Vlado; Sokolov, Artem; Newton, Yulia; Beltran, Himisha; Demichelis, Francesca; Rubin, Mark A.; Stuart, Joshua M.; Small, Eric J.	JOURNAL OF CLINICAL ONCOLOGY	2018	36	24	93	31
Decipher Genomic Classifier Measured on Prostate Biopsy Predicts Metastasis Risk	Klein, Eric A.; Haddad, Zaid; Yousefi, Kasra; Lam, Lucia L. C.; Wang, Qiqi; Choeurng, Voleak; Palmer-Aronsten, Beatrix; Buerki, Christine; Davicioni, Elai; Li, Jianbo; Kattan, Michael W.; Stephenson, Andrew J.; Magi-Galluzzi, Cristina	UROLOGY	2016	90		79	15,8

Combined Tumor Suppressor Defects Characterize Clinically Defined Aggressive Variant Prostate Cancers	Aparicio, Ana M.; Shen, Li; Tapia, Elsa Li Ning; Lu, Jing-Fang; Chen, Hsiang-Chun; Zhang, Jiexin; Wu, Guanglin; Wang, Xuemei; Troncoso, Patricia; Corn, Paul; Thompson, Timothy C.; Broom, Bradley; Baggerly, Keith; Maity, Sankar N.; Logothetis, Christopher J.	CLINICAL CANCER RESEARCH	2016	22	6	78	15,6
miR-195 Inhibits Tumor Progression by Targeting RPS6KB1 in Human Prostate Cancer	Cai, Chao; Chen, Qing-Biao; Han, Zhao-Dong; Zhang, Yan-Qiong; He, Hui-Chan; Chen, Jia-Hong; Chen, Yan-Ru; Yang, Sheng-Bang; Wu, Yong-Ding; Zeng, Yan-Ru; Qin, Guo-Qiang; Liang, Yu-Xiang; Dai, Qi-Shan; Jiang, Fu-Neng; Wu, Shu-lin; Zeng, Guo-Hua; Zhong, Wei-De; Wu, Chin-Lee	CLINICAL CANCER RESEARCH	2015	21	21	76	12,67
Phosphoproteome Integration Reveals Patient-Specific Networks in Prostate Cancer	Drake, Justin M.; Paull, Evan O.; Graham, Nicholas A.; Lee, John K.; Smith, Bryan A.; Titz, Bjoern; Stoyanova, Tanya; Faltermeier, Claire M.; Uzunangelov, Vladislav; Carlin, Daniel E.; Fleming, Daniel Teo; Wong, Christopher K.; Newton, Yulia; Sudha, Sud; Vashisht, Ajay A.; Huang, Jiaoti; Wohlschlegel, James A.; Graeber, Thomas G.; Witte, Owen N.; Stuart, Joshua M.	CELL	2016	166	4	72	14,4
Intrinsic BET inhibitor resistance in SPOP-mutated prostate cancer is mediated by BET protein stabilization and AKT-mTORC1 activation	Zhang, Pingzhao; Wang, Dejie; Zhao, Yu; Ren, Shancheng; Gao, Kun; Ye, Zhenqing; Wang, Shangqian; Pan, Chun-Wu; Zhu, Yasheng; Yan, Yuqian; Yang, Yinhui; Wu, Di; He, Yundong; Zhang, Jun; Lu, Daru; Liu, Xiuping; Yu, Long; Zhao, Shimin; Li, Yao; Lin, Dong; Wang, Yuzhuo; Wang, Liguo; Chen, Yu; Sun, Yinghao; Wang, Chenji; Huang, Haojie	NATURE MEDICINE	2017	23	9	72	18

Novel Biomarker Signature That May Predict Aggressive Disease in African American Men With Prostate Cancer	Yamoah, Kosj; Johnson, Michael H.; Choeurng, Voleak; Faisal, Farzana A.; Yousefi, Kasra; Haddad, Zaid; Ross, Ashley E.; Alshalafa, Mohammed; Den, Robert; Lal, Priti; Feldman, Michael; Dicker, Adam P.; Klein, Eric A.; Davicioni, Elai; Rebbeck, Timothy R.; Schaeffer, Edward M.	JOURNAL OF CLINICAL ONCOLOGY	2015	33	25	70	11,67
SRRM4 Drives Neuroendocrine Transdifferentiation of Prostate Adenocarcinoma Under Androgen Receptor Pathway Inhibition	Li, Yinan; Donmez, Nilgun; Sahinalp, Cenk; Xie, Ning; Wang, Yuwei; Xue, Hui; Mo, Fan; Beltran, Himisha; Gleave, Martin; Wang, Yuzhuo; Collins, Colin; Dong, Xuesen	EUROPEAN UROLOGY	2017	71	1	69	17,25
Development and Clinical Validation of an In Situ Biopsy-Based Multimarker Assay for Risk Stratification in Prostate Cancer	Blume-Jensen, Peter; Berman, David M.; Rimm, David L.; Shipitsin, Michail; Putzi, Mathew; Nifong, Thomas P.; Small, Clayton; Choudhury, Sibgat; Capela, Teresa; Coupal, Louis; Ernst, Christina; Hurley, Aeron; Kaprelyants, Alex; Chang, Hua; Giladi, Eldar; Nardone, Julie; Dunyak, James; Loda, Massimo; Klein, Eric A.; Magi-Galluzzi, Cristina; Latour, Mathieu; Epstein, Jonathan I.; Kantoff, Philip; Saad, Fred	CLINICAL CANCER RESEARCH	2015	21	11	69	11,5
Stem cell and neurogenic gene-expression profiles link prostate basal cells to aggressive prostate cancer	Zhang, Dingxiao; Park, Daechan; Zhong, Yi; Lu, Yue; Rycaj, Kiera; Gong, Shuai; Chen, Xin; Liu, Xin; Chao, Hsueh-Ping; Whitney, Pamela; Calhoun-Davis, Tammy; Takata, Yoko; Shen, Jianjun; Iyer, Vishwanath R.; Tang, Dean G.	NATURE COMMUNICATIONS	2016	7		65	13

MicroRNA expression signature of castration-resistant prostate cancer: the microRNA-221/222 cluster functions as a tumour suppressor and disease progression marker	Goto, Yusuke; Kojima, Satoko; Nishikawa, Rika; Kurozumi, Akira; Kato, Mayuko; Enokida, Hideki; Matsushita, Ryosuke; Yamazaki, Kazuto; Ishida, Yasuo; Nakagawa, Masayuki; Naya, Yukio; Ichikawa, Tomohiko; Seki, Naohiko	BRITISH JOURNAL OF CANCER	2015	113	7	65	10,83
ROR-gamma drives androgen receptor expression and represents a therapeutic target in castration-resistant prostate cancer	Wang, Junjian; Zou, June X.; Xue, Xiaoqian; Cai, Demin; Zhang, Yan; Duan, Zhijian; Xiang, Qiuping; Yang, Joy C.; Louie, Maggie C.; Borowsky, Alexander D.; Gao, Allen C.; Evans, Christopher P.; Lam, Kit S.; Xu, Jianzhen; Kung, Hsing-Jien; Evans, Ronald M.; Xu, Yong; Chen, Hong-Wu	NATURE MEDICINE	2016	22	5	65	13
Biology and evolution of poorly differentiated neuroendocrine tumors	Rickman, David S.; Beltran, Himisha; Demichelis, Francesca; Rubin, Mark A.	NATURE MEDICINE	2017	23	6	60	15
Minimal functional driver gene heterogeneity among untreated metastases	Reiter, Johannes G.; Makohon-Moore, Alvin P.; Gerold, Jeffrey M.; Heyde, Alexander; Attiyeh, Marc A.; Kohutek, Zachary A.; Tokheim, Collin J.; Brown, Alexia; DeBlasio, Rayne M.; Niyazov, Juliana; Zucker, Amanda; Karchin, Rachel; Kinzler, Kenneth W.; Iacobuzio-Donahue, Christine A.; Vogelstein, Bert; Nowak, Martin A.	SCIENCE	2018	361	6406	58	19,33

Development and Validation of a Novel Integrated Clinical-Genomic Risk Group Classification for Localized Prostate Cancer	Spratt, Daniel E.; Zhang, Jingbin; Santiago-Jimenez, Maria; Dess, Robert T.; Davis, John W.; Den, Robert B.; Dicker, Adam P.; Kane, Christopher J.; Pollack, Alan; Stoyanova, Radka; Abdollah, Firas; Ross, Ashley E.; Cole, Adam; Uchio, Edward; Randall, Josh M.; Hao Nguyen; Zhao, Shuang G.; Mehra, Rohit; Glass, Andrew G.; Lam, Lucia L. C.; Chelliserry, Jijumon; du Plessis, Marguerite; Choeurng, Voleak; Aranes, Maria; Kolisnik, Tyler; Margrave, Jennifer; Alter, Jason; Jordan, Jennifer; Buerki, Christine; Yousefi, Kasra; Haddad, Zaid; Davicioni, Elai; Trabulsi, Edouard J.; Loeb, Stacy; Tewari, Ashutosh; Carroll, Peter R.; Weinmann, Sheila; Schaeffer, Edward M.; Klein, Eric A.; Karnes, R. Jeffrey; Feng, Felix Y.; Nguyen, Paul L.	JOURNAL OF CLINICAL ONCOLOGY	2018	36	6	53	17,67
Meta-analysis of miRNA expression profiles for prostate cancer recurrence following radical prostatectomy	Pashaei, Elnaz; Pashaei, Elham; Ahmady, Maryam; Ozen, Mustafa; Aydin, Nizamettin	PLOS ONE	2017	12	6	50	12,5

Patient derived organoids to model rare prostate cancer phenotypes	Puca, Loredana; Bareja, Rohan; Prandi, Davide; Shaw, Reid; Benelli, Matteo; Karthaus, Wouter R.; Hess, Judy; Sigouros, Michael; Donoghue, Adam; Kossai, Myriam; Gao, Dong; Cyrta, Joanna; Sailer, Verena; Vosoughi, Aram; Pauli, Chantal; Churakova, Yelena; Cheung, Cynthia; Deonarine, Lesa Dayal; McNary, Terra J.; Rosati, Rachele; Tagawa, Scott T.; Nanus, David M.; Mosquera, Juan Miguel; Sawyers, Charles L.; Chen, Yu; Inghirami, Giorgio; Rao, Rema A.; Grandori, Carla; Elemento, Olivier; Sboner, Andrea; Demichelis, Francesca; Rubin, Mark A.; Beitrah, Himisha	NATURE COMMUNICATIONS	2018	9		49	16,33
The Proteome of Primary Prostate Cancer	Iglesias-Gato, Diego; Wikstrom, Pernilla; Tyanova, Stefka; Lavallee, Charlotte; Thysell, Elin; Carlsson, Jessica; Hagglof, Christina; Cox, Juergen; Andren, Ove; Stattin, Par; Egevad, Lars; Widmark, Anders; Bjartell, Anders; Collins, Colin C.; Bergh, Anders; Geiger, Tamar; Mann, Matthias; Flores-Morales, Amilcar	EUROPEAN UROLOGY	2016	69	5	49	9,8
Polycomb-mediated silencing in neuroendocrine prostate cancer	Clermont, Pier-Luc; Lin, Dong; Crea, Francesco; Wu, Rebecca; Xue, Hui; Wang, Yuwei; Thu, Kelsie L.; Lam, Wan L.; Collins, Colin C.; Wang, Yuzhuo; Helgason, Cheryl D.	CLINICAL EPIGENETICS	2015	7		44	7,33

Genomic Classifier Augments the Role of Pathological Features in Identifying Optimal Candidates for Adjuvant Radiation Therapy in Patients With Prostate Cancer: Development and Internal Validation of a Multivariable Prognostic Model	Dalela, Deepansh; Santiago-Jimenez, Maria; Yousefi, Kasra; Karnes, R. Jeffrey; Ross, Ashley E.; Den, Robert B.; Freedland, Stephen J.; Schaeffer, Edward M.; Dicker, Adam P.; Menon, Mani; Briganti, Alberto; Davicioni, Elai; Abdollah, Firas	JOURNAL OF CLINICAL ONCOLOGY	2017	35	18	39	9,75
Long non-coding RNA urothelial carcinoma associated 1 (UCA1) mediates radiation response in prostate cancer	Ghiam, Alireza Fotouhi; Taeb, Samira; Huang, Xiaoyong; Huang, Vincent; Ray, Jessica; Scarcello, Seville; Hoey, Christianne; Jahangiri, Sahar; Fokas, Emmanouil; Loblaw, Andrew; Bristow, Robert G.; Vesprini, Danny; Boutros, Paul; Liu, Stanley K.	ONCOTARGET	2017	8	3	35	8,75
Transcriptome Sequencing Reveals PCAT5 as a Novel ERG-Regulated Long Noncoding RNA in Prostate Cancer	Ylipaa, Antti; Kivinummi, Kati; Kohvakka, Annika; Annala, Matti; Latonen, Leena; Scaravilli, Mauro; Kartasalo, Kimmo; Leppanen, Simo-Pekka; Karakurt, Serdar; Seppala, Janne; Yli-Harja, Olli; Tammela, Teuvo L. J.; Zhang, Wei; Visakorpi, Tapio; Nykter, Matti	CANCER RESEARCH	2015	75	19	35	5,83

Validation of a Genomic Classifier for Predicting Post-Prostatectomy Recurrence in a Community Based Health Care Setting	Glass, Andrew G.; Leo, Michael C.; Haddad, Zaid; Yousefi, Kasra; du Plessis, Marguerite; Chen, Chuhe; Choeurng, Voleak; Abdollah, Firas; Robbins, Bruce; Ra, Seong; Richert-Boe, Kathryn E.; Buerki, Christine; Pearson, Kathy; Davicioni, Elai; Weinmann, Sheila	JOURNAL OF UROLOGY	2016	195	6	25	5
A Phase II Trial of the Aurora Kinase A Inhibitor Alisertib for Patients with Castration-resistant and Neuroendocrine Prostate Cancer: Efficacy and Biomarkers	Beltran, Himisha; Oromendia, Clara; Danila, Daniel C.; Montgomery, Bruce; Hoimes, Christopher; Szmulewitz, Russell Z.; Vaishampayan, Ulka; Armstrong, Andrew J.; Stein, Mark; Pinski, Jacek; Mosquera, Juan M.; Sailer, Verena; Bareja, Rohan; Romanel, Alessandro; Gumpeni, Naveen; Sboner, Andrea; Dardenne, Etienne; Puca, Loredana; Prandi, Davide; Rubin, Mark A.; Scher, Howard I.; Rickman, David S.; Demichelis, Francesca; Nanus, David M.; Ballman, Karla V.; Tagawa, Scott T.	CLINICAL CANCER RESEARCH	2019	25	1	25	12,5
Differentially methylated genes and androgen receptor re-expression in small cell prostate carcinomas	Kleb, Brittany; Estecio, Marcos R. H.; Zhang, Jiexin; Tzelepid, Vassiliki; Chung, Woonbok; Jelinek, Jaroslav; Navone, Nora M.; Tahir, Salahaldin; Marquez, Victor E.; Issa, Jean-Pierre; Maity, Sankar; Aparicio, Ana	EPIGENETICS	2016	11	3	24	4,8

Decipher Test Impacts Decision Making Among Patients Considering Adjuvant and Salvage Treatment After Radical Prostatectomy: Interim Results From the Multicenter Prospective PRO-IMPACT Study	Gore, John L.; du Plessis, Marguerite G.; Santiago-Jimenez, Maria K.; Yousefi, Kasra K.; Thompson, Darby K.; Karsh, Lawrence P.; Lane, Brian R.; Franks, Michael T.; Chen, David S.; Bandyk, Mark P.; Bianco, Fernando J.; Brown, Gordon T.; Clark, William R.; Kibel, Adam S.; Kim, Hyung A.; Lowrance, William M.; Manoharan, Murugesan T.; Maroni, Paul U.; Perrapato, Scott, V; Sieber, Paul R.; Trabulsi, Edouard J.; Waterhouse, Robert C.; Davicioni, Elai T.; Lotan, Yair A.; Lin, Daniel W.	CANCER	2017	123	15	24	6
Effect of a genomic classifier test on clinical practice decisions for patients with high-risk prostate cancer after surgery	Badani, Ketan K.; Thompson, Darby J.; Brown, Gordon; Holmes, Daniel; Kella, Naveen; Albala, David; Singh, Amar; Buerki, Christine; Davicioni, Elai; Hornberger, John	BJU INTERNATIONA L	2015	115	3	24	4
BRD4 Promotes DNA Repair and Mediates the Formation of TMPRSS2-ERG Gene Rearrangements in Prostate Cancer	Li, Xiangyi; Baek, GuemHee; Ramanand, Susmita G.; Sharp, Adam; Gao, Yunpeng; Yuan, Wei; Welti, Jon; Rodrigues, Daniel N.; Dolling, David; Figueiredo, Ines; Sumanasuriya, Semini; Crespo, Mateus; Aslam, Adam; Li, Rui; Yin, Yi; Mukherjee, Bipasha; Kanchwala, Mohammed; Hughes, Ashley M.; Halsey, Wendy S.; Chiang, Cheng-Ming; Xing, Chao; Raj, Ganesh V.; Burma, Sandeep; de Bono, Johann; Mani, Ram S.	CELL REPORTS	2018	22	3	23	7,67

Translating a Prognostic DNA Genomic Classifier into the Clinic: Retrospective Validation in 563 Localized Prostate Tumors	Lalonde, Emilie; Alkallas, Rached; Chua, Melvin Lee Kiang; Fraser, Michael; Haider, Syed; Meng, Alice; Zheng, Junyan; Yao, Cindy Q.; Picard, Valerie; Orain, Michele; Hovington, Helene; Murgic, Jure; Berlin, Alejandro; Lacombe, Louis; Bergeron, Alain; Fradet, Yves; Tetu, Bernard; Lindberg, Johan; Egevad, Lars; Gronberg, Henrik; Ross-Adams, Helen; Lamb, Alastair D.; Halim, Silvia; Dunning, Mark J.; Neal, David E.; Pintilie, Melania; van der Kwast, Theodorus; Bristow, Robert G.; Boutros, Paul C.	EUROPEAN UROLOGY	2017	72	1	22	5,5
Androgen receptor splice variants bind to constitutively open chromatin and promote abiraterone-resistant growth of prostate cancer	He, Yundong; Lu, Ji; Ye, Zhenqing; Hao, Siyuan; Wang, Liewei; Kohli, Manish; Tindall, Donald J.; Li, Benyi; Zhu, Runzhi; Wang, Liguo; Huang, Haojie	NUCLEIC ACIDS RESEARCH	2018	46	4	21	7
Genomic deletion of chromosome 12p is an independent prognostic marker in prostate cancer	Kluth, Martina; Ahrary, Ramin; Hube-Magg, Claudia; Ahmed, Malik; Volta, Heinke; Schwemin, Catina; Steurer, Stefan; Wittmer, Corinna; Wilczak, Waldemar; Krech, Eike Burandt Till; Adam, Meike; Michl, Uwe; Heinzer, Hans; Salomon, Georg; Graefen, Markus; Koop, Christina; Minner, Sarah; Simon, Ronald; Sauter, Guido; Schlomm, Thorsten	ONCOTARGET	2015	6	29	20	3,33

ONECUT2 is a driver of neuroendocrine prostate cancer	Guo, Haiyang; Ci, Xinpei; Ahmed, Musaddeque; Hua, Junjie Tony; Soares, Fraser; Lin, Dong; Puca, Loredana; Vosoughi, Aram; Xue, Hui; Li, Estelle; Su, Peiran; Chen, Sujun; Tran Nguyen; Liang, Yi; Zhang, Yuzhe; Xu, Xin; Xu, Jing; Sheahan, Anjali V.; Ba-Alawi, Wail; Zhang, Si; Mahamud, Osman; Vellanki, Ravi N.; Gleave, Martin; Bristow, Robert G.; Haibe-Kains, Benjamin; Poirier, John T.; Rudin, Charles M.; Tsao, Ming-Sound; Wouters, Bradly G.; Fazli, Ladan; Feng, Felix Y.; Ellis, Leigh; van der Kwast, Theo; Berlin, Alejandro; Koritzinsky, Marianne; Boutros, Paul C.; Zoubeidi, Amina; Beltran, Himisha; Wang, Yuzhuo; He, Housheng Hansen	NATURE COMMUNICATIONS	2019	10		19	9,5
Proteomic Tissue-Based Classifier for Early Prediction of Prostate Cancer Progression	Gao, Yuqian; Wang, Yi-Ting; Chen, Yongmei; Wang, Hui; Young, Denise; Shi, Tujin; Song, Yingjie; Schepmoes, Athena A.; Kuo, Claire; Fillmore, Thomas L.; Qian, Wei-Jun; Smith, Richard D.; Srivastava, Sudhir; Kagan, Jacob; Dobi, Albert; Sesterhenn, Isabell A.; Rosner, Inger L.; Petrovics, Gyorgy; Rodland, Karin D.; Srivastava, Shiv; Cullen, Jennifer; Liu, Tao	CANCERS	2020	12	5	1	1

Clinical and genomic insights into circulating tumor DNA-based alterations across the spectrum of metastatic hormone-sensitive and castrate-resistant prostate cancer	Kohli, Manish; Tan, Winston; Zheng, Tiantian; Wang, Amy; Montesinos, Carlos; Wong, Calven; Du, Pan; Jia, Shidong; Yadav, Siddhartha; Horvath, Lisa G.; Mahon, Kate L.; Kwan, Edmond M.; Fettke, Heidi; Yu, Jianjun; Azad, Arun A.	EBIOMEDICINE	2020	54		1	1
Multi-omic serum biomarkers for prognosis of disease progression in prostate cancer	Kiebish, Michael A.; Cullen, Jennifer; Mishra, Prachi; Ali, Amina; Milliman, Eric; Rodrigues, Leonardo O.; Chen, Emily Y.; Tolstikov, Vladimir; Zhang, Lixia; Panagopoulos, Kiki; Shah, Punit; Chen, Yongmei; Petrovics, Gyorgy; Rosner, Inger L.; Sesterhenn, Isabell A.; McLeod, David G.; Granger, Elder; Sarangarajan, Rangaprasad; Akmaev, Viatcheslav; Srinivasan, Alagarsamy; Srivastava, Shiv; Narain, Niven R.; Dobi, Albert	JOURNAL OF TRANSLATIONAL MEDICINE	2020	18	1	1	1
2,4-dienoyl-CoA reductase regulates lipid homeostasis in treatment-resistant prostate cancer	Blomme, Arnaud; Ford, Catriona A.; Mui, Ernest; Patel, Rachana; Ntala, Chara; Jamieson, Lauren E.; Planque, Melanie; McGregor, Grace H.; Peixoto, Paul; Hervouet, Eric; Nixon, Colin; Salji, Mark; Gaughan, Luke; Markert, Elke; Repiscak, Peter; Sumpton, David; Blanco, Giovanny Rodriguez; Lilla, Sergio; Kamphorst, Jurre J.; Graham, Duncan; Faulds, Karen; MacKay, Gillian M.; Fendt, Sarah-Maria; Zanivan, Sara; Leung, Hing Y.	NATURE COMMUNICATIONS	2020	11	1	0	0

SLFN11 Expression in Advanced Prostate Cancer and Response to Platinum-based Chemotherapy	Conteduca, Vincenza; Ku, Sheng-Yu; Puca, Loredana; Slade, Megan; Fernandez, Luisa; Hess, Judy; Bareja, Rohan; Vlachostergios, Panagiotis J.; Sigouros, Michael; Mosquera, Juan Miguel; Sboner, Andrea; Nanus, David M.; Elemento, Olivier; Dittamore, Ryan; Tagawa, Scott T.; Beltran, Himisha	MOLECULAR CANCER THERAPEUTICS	2020	19	5	0	0
Exome sequencing identified six copy number variations as a prediction model for recurrence of primary prostate cancers with distinctive prognosis	Liu, Jie; Yan, Jiajun; Mao, Ruifang; Ren, Guoping; Liu, Xiaoyan; Zhang, Yanling; Wang, Jili; Wang, Yan; Li, Meiling; Qiu, Qingchong; Wang, Lin; Liu, Guanfeng; Jin, Shanshan; Ma, Liang; Ma, Yingying; Zhao, Na; Zhang, Hongwei; Lin, Biaoyang	TRANSLATIONAL CANCER RESEARCH	2020	9	4	0	0
STAT3-dependent analysis reveals PDK4 as independent predictor of recurrence in prostate cancer	Oberhuber, Monika; Pecoraro, Matteo; Rusz, Mate; Oberhuber, Georg; Wieselberg, Maritta; Haslinger, Peter; Gurnhofer, Elisabeth; Schlederer, Michaela; Limberger, Tanja; Lagger, Sabine; Pencik, Jan; Kodajova, Petra; Hoegler, Sandra; Stockmaier, Georg; Grund-Groeschke, Sandra; Aberger, Fritz; Bolis, Marco; Theurillat, Jean-Philippe; Wiebringhaus, Robert; Weiss, Theresa; Haitel, Andrea; Brehme, Marc; Wadsak, Wolfgang; Griss, Johannes; Mohr, Thomas; Hofer, Alexandra; Jaeger, Anton; Pollheimer, Juergen; Egger, Gerda; Koellensperger, Gunda; Mann, Matthias; Hantusch, Brigitte; Kenner, Lukas	MOLECULAR SYSTEMS BIOLOGY	2020	16	4	0	0

Integrative Analysis of MicroRNA and Gene Interactions for Revealing Candidate Signatures in Prostate Cancer	Wei, Jingchao; Yin, Yinghao; Deng, Qiancheng; Zhou, Jun; Wang, Yong; Yin, Guangming; Yang, Jianfu; Tang, Yuxin	FRONTIERS IN GENETICS	2020	11		0	0
Integrative clinical transcriptome analysis reveals TMPRSS2-ERG dependency of prognostic biomarkers in prostate adenocarcinoma	Gerke, Julia S.; Orth, Martin F.; Tolkach, Yuri; Romero-Perez, Laura; Wehweck, Fabienne S.; Stein, Stefanie; Musa, Julian; Knott, Maximilian M. L.; Hoelting, Tilman L. B.; Li, Jing; Sannino, Giuseppina; Marchetto, Aruna; Ohmura, Shunya; Cidre-Aranaz, Florencia; Mueller-Nurasyid, Martina; Strauch, Konstantin; Stief, Christian; Kristiansen, Glen; Kirchner, Thomas; Buchner, Alexander; Gruenewald, Thomas G. P.	INTERNATIONAL JOURNAL OF CANCER	2020	146	7	0	0
